# Synthesis and Structural Study of Amidrazone Derived Pyrrole-2,5-Dione Derivatives: Potential Anti-Inflammatory Agents

**DOI:** 10.3390/molecules27092891

**Published:** 2022-04-30

**Authors:** Renata Paprocka, Leszek Pazderski, Liliana Mazur, Małgorzata Wiese-Szadkowska, Jolanta Kutkowska, Michalina Nowak, Anna Helmin-Basa

**Affiliations:** 1 Department of Organic Chemistry, Faculty of Pharmacy, Collegium Medicum in Bydgoszcz, Nicolaus Copernicus University in Toruń, Jurasza Str. 2, 85-089 Bydgoszcz, Poland; 2 Department of Analytical Chemistry and Applied Spectroscopy, Faculty of Chemistry, Nicolaus Copernicus University in Toruń, Gagarina Str. 7, 87-100 Torun, Poland; leszekp@chem.umk.pl; 3 Institute of Chemical Sciences, Faculty of Chemistry, Maria Curie-Skłodowska University, Pl. Marii Curie-Sklodowskiej 2, 20-031 Lublin, Poland; liliana.mazur@mail.umcs.pl; 4 Department of Immunology, Faculty of Pharmacy, Collegium Medicum in Bydgoszcz, Nicolaus Copernicus University in Toruń, M. Curie-Sklodowska Str. 9, 85-094 Bydgoszcz, Poland; mwiese@cm.umk.pl (M.W.-S.); michalina.nowak2442@gmail.com (M.N.); 5 Department of Genetics and Microbiology, Institute of Biological Sciences, Maria Curie-Skłodowska University, Akademicka Str. 19, 20-033 Lublin, Poland; jolanta.kutkowska@mail.umcs.pl

**Keywords:** amidrazone, pyrrole-2,5-dione, cyclic imide, anti-inflammatory activity, antiproliferative activity, antibacterial activity

## Abstract

1*H*-pyrrole-2,5-dione derivatives are known for their wide range of pharmacological properties, including anti-inflammatory and antimicrobial activities. This study aimed to synthesize new 3,4-dimethyl-*1H*-pyrrole-2,5-dione derivatives **2a**–**2f** in the reaction of *N*^3^-substituted amidrazones with 2,3-dimethylmaleic anhydride and evaluate their structural and biological properties. Compounds **2a**–**2f** were studied by the ^1^H-^13^C NMR two-dimensional techniques (HMQC, HMBC) and single-crystal X-ray diffraction (derivatives **2a** and **2d**). The anti-inflammatory activity of compounds **2a**–**2f** was examined by both an anti-proliferative study and a production study on the inhibition of pro-inflammatory cytokines (IL-6 and TNF-α) in anti-CD3 antibody- or lipopolysaccharide-stimulated human peripheral blood mononuclear cell (PBMC) cultures. The antibacterial activity of compounds **2a–2f** against *Staphylococcus aureus*, *Enterococcus faecalis*, *Micrococcus luteus*, *Esherichia coli*, *Pseudomonas aeruginosa*, *Yersinia enterocolitica*, *Mycobacterium smegmatis* and *Nocardia corralina* strains was determined using the broth microdilution method. Structural studies of **2a**–**2f** revealed the presence of distinct *Z* and *E* stereoisomers in the solid state and the solution. All compounds significantly inhibited the proliferation of PBMCs in anti-CD3-stimulated cultures. The strongest effect was observed for derivatives **2a**–**2d**. The strongest inhibition of pro-inflammatory cytokine production was observed for the most promising anti-inflammatory compound **2a**.

## 1. Introduction

Five-membered heterocyclic nitrogen-containing rings with two carbonyl groups adjacent to the N atom are present in many organic compounds exhibiting various biological activities, including antipsychotic (perospirone), anxiolytic and antidepressant (tandospirone), antiepileptic (ethosuximide), antiproliferative, immunomodulating and antineoplastic (thalidomide, pomalidomide) activities. Similar to many species studied recently, these well-known drugs contain variously substituted 1*H*-pyrrolidine-2,5-dione rings [1,2]. However, some interest has focused on 1*H*-pyrrole-2,5-dione derivatives during the last decade. In particular, the latter ring system is present in some anti-inflammatory compounds [3], e.g., those inhibiting *lipopolysaccharide (LPS)*-induced PGE2 production in RAW 264.7 macrophage cells [4,5] and cyclooxygenases (COX-1 and COX-2 enzymes) [5]. A similar inhibitory activity against COX-1 and COX-2 was exhibited for the N-Mannich bases derived from pyrrolo [3,4-c] pyrrol-1,3-dione [6]. Thereafter, some natural *1H*-pyrrole-2,5-dione derivatives, called aquabamycins, were described as antibacterial agents [7], while 3-bromo-*1H*-pyrrole-2,5-dione and 3,4-dibromo-*1H*-pyrrole-2,5-dione were described as antifungal and cytotoxic agents [8]. 1*H*-pyrrole-2,5-diones also play a role in cholesterol absorption as they are HMG-CoA reductase inhibitors [9]. Finally, many N(1)-substituted 1*H*-pyrrole-2,5-dione derivatives possess anti-fungal and insecticidal (larvicidal) [10], as well as anti-tumor [11] and antiviral [12] activities.

The simplest representatives of this class of chemicals are 1*H*-pyrrole-2,5-dione, also referred to as maleimide (i.e., maleic acid imide) [13,14,15], 3-methyl-1*H*-pyrrole-2,5-dione [16,17,18,19,20], 3,4-dimethyl-1*H*-pyrrole-2,5-dione [14,16,21,22], 3,4-diethyl-1*H*-pyrrole-2,5-dione [23], and 3,4-diphenyl-1*H*-pyrrole-2,5-dione [21,24,25]. Then, there are their N(1)-methyl derivatives (1-methyl-1*H*-pyrrole-2,5-dione, 1,3-dimethyl-1*H*-pyrrole-2,5-dione, 1,3,4-trimethyl-1*H*-pyrrole-2,5-dione and 1-methyl-3,4-diphenyl-1*H*-pyrrole-2,5-dione) [16,19,26,27,28,29,30,31,32,33], as well as various N(1)-amino derivatives (containing the moiety of –NH–: 1-phenylamino-, 1-(4-methylphenyl)amino-, 1-(4-methoxyphenyl)amino-, 1-(4-bromophenylamino)-, or of –N <: 1,1-dimethylamino-, 1,1-diphenylamino-, 1-(piperidyn-1-yl)-, 1-(morpholin-4-yl)-, 1-(4-methylpiperazin-1-yl)-, dimeric species consisting of two identical N(1),N(1′)-bonded 1*H*-pyrrole-2,5-dione ring systems) [34,35,36,37,38] and N(1)-amido (containing the moiety of –NH–CO–: 1-benzamido-, 1-(4-methoxybenzamido)-, 1-(4-bromobenzamido)-, and 1-(4-nitrobenzamido)-, or of –NH–CO–O–: 1-methoxycarbonylamino-) [39,40] analogues which were widely studied by ^1^H and ^13^C NMR, and by single crystal X-ray diffraction (see Table S1, Supplementary Materials, part A). In contrast, the analogous N(1)-imino species (containing the –N= moiety) are less known; the ^1^H and ^13^C NMR data are available only for two series of variously substituted (in the phenyl ring of the imino substituent) derivatives of 1-((*E*-4-phenylbut-3-en-2-ylidene)imino)-*1H*-pyrrole-2,5-dione and 1-((*E*-4-phenylbut-3-en-2-ylidene)imino)-3-methyl-*1H*-pyrrole-2,5-dione [10]. Their X-ray structures have never been reported.

**Figure 1:**
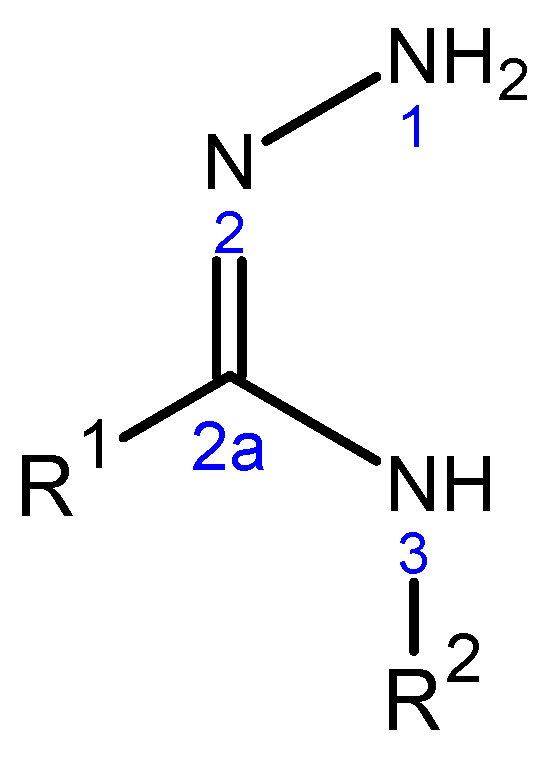
*N*^3^-substituted amidrazones **1a**–**1f. 1a**: R^1^ = phenyl, R^2^ = phenyl. **1b**: R^1^ = 2-pyridyl, R^2^ = phenyl. **1c**: R^1^ = 4-pyridyl, R^2^ = phenyl. **1d**: R^1^ = 2-pyridyl, R^2^ = 2-pyridyl. **1e**: R^1^ = 2-pyridyl, R^2^ = 4-methylphenyl. **1f**: R^1^ = 2-pyridyl, R^2^ = 4-nitrophenyl.

**Figure 2:**
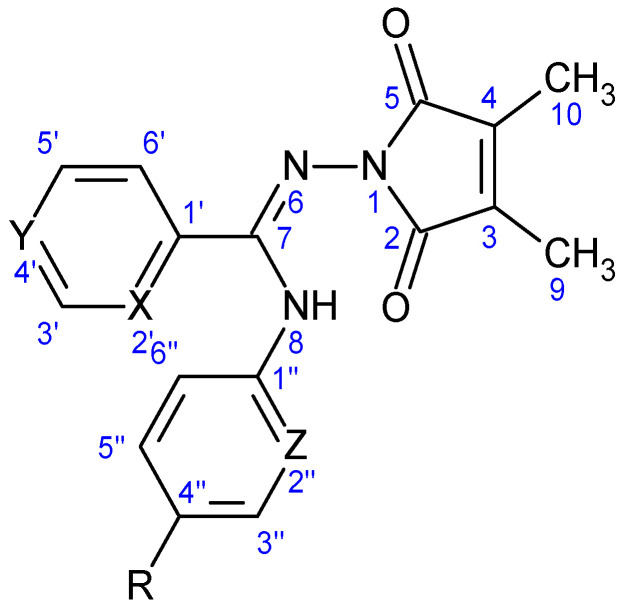
The studied N(1)-substituted derivatives of 3,4-dimethyl-*1H*-pyrrole-2,5-dione (**2a**–**2f**) together with the numbering scheme. **2a:** X = C, Y = C, Z = C, R = H. **2b:** X = N, Y = C, Z = C, R = H. **2c:** X = C, Y = N, Z = C, R = H. **2d:** X = N, Y = C, Z = N, R = H. **2e:** X = N, Y = C, Z = C, R = CH_3._ **2f:** X = N, Y = C, Z = C, R = NO_2_.

The aim of this study was the synthesis of six new 3,4-dimethyl-*1H*-pyrrole-2,5-diones, N(1)-substituted by the imino moieties derived from *N*^3^-substituted amidrazones (**1a**–**1f**, Figure 1) [41] which have the general formula shown in Figure 2 (**2a**–**2f**). Thereafter, the main goal was the investigation of their structural and spectroscopic (^1^H, ^13^C NMR) properties in their solid state and solution, together with the evaluation of their biological activity. This was done to gain insight into the influence of the R^1^ and R^2^ substituents on the molecular conformation and intermolecular interactions and the anti-inflammatory and antibacterial properties of the compounds.

Nitrogen-containing heterocycles, specifically cyclic imides, could be synthesised from amidrazones and dicarboxylic acid anhydrides [42,43]. The uncertainty in using this method is related to the fact that the behaviour of the best known *N*^3^-substituted amidrazones in reactions with such cyclic anhydrides is largely dependent on the type of R^1^ and R^2^ substituents, the applied anhydride and the reaction conditions. For example, the reactions of *N*^3^-substituted amidrazones (also including **1a**–**1f**) with maleic anhydride led to 1,2,4-triazole derivatives [44], whereas those with itaconic anhydride led to either acyclic compounds (in the case of **1b**–**1f**) [45,46] or 1,2,4-triazole derivatives (among others, in the case of **1a**–**1b** and **1d**–**1f**) [47]. In contrast, the reactions of **1d** with succinic, *trans*- and *cis*-1,2-cyclohexanedicarboxylic, maleic, phthalic, *cis*-1,2,3,6-tetrahydrophthalic, pyridine-2,3-dicarboxylic and pyridine-3,4-dicarboxylic anhydrides resulted only in acyclic species [48,49]. On the other hand, although a number of heterocycles containing the N(1)-substituted –CO–N(1)–CO– moiety was obtained in the reactions of **1a**–**1c** with *cis*-1,2-cyclohexanedicarboxylic anhydride, these were derivatives of 1,2-cyclohexanedicarboximide (i.e., hexahydrophtalimide or hexahydroisoindole-1,3-dione), possessing *1H*-pyrrolidine-2,5-dione and not the *1H*-pyrrole-2,5-dione moiety. Moreover, in the same syntheses, some acyclic compounds (the case of **1b**–**1d** and **1f**) and/or 1,2,4-triazole derivatives (the case of **1a** and **1d**–**1e**) were also formed, sometimes even simultaneously [42,43].

**Scheme 1:**
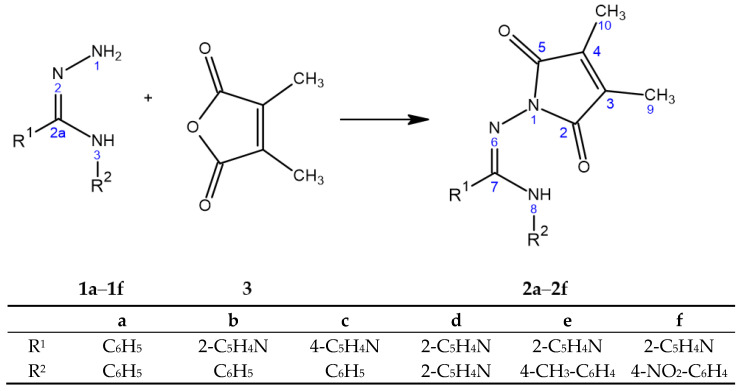
The synthesis of **2a**–**2f**.

Hence, the selective preparation of **2a**–**2f** seemed to be a challenge. Nevertheless, it was achieved after performing a series of successful reactions of **1a**–**1f** with 2,3-dimethylmaleic anhydride (**3**), as shown in Scheme 1.

## 2. Results and Discussion

### 2.1. The Syntheses of Compounds **2a**–**2f**

In this work, a series of N(1)-substituted derivatives of 3,4-dimethyl-*1H*-pyrrole-2,5-dione **2a**–**2f** were prepared from the respective *N*^3^-substituted amidrazones **1a**–**1f** and 2,3-dimethylmaleic anhydride **3** (Scheme 1). The syntheses of **2a**–**2c** and **2e**–**2f** were carried out in toluene, chloroform or diethyl ether. The best yields (75–95%) were obtained at the boiling points of chloroform or toluene in a much shorter time than at room temperature. The exception was compound **2d,** which was obtained only in diethyl ether at room temperature. Possibly the presence of two 2-pyridyl substituents hinders the formation of this product. The detailed dependencies of **2a**–**2f** yields with the solvent, temperature and time are presented in Tables S2–S7 (Supplementary Data, part B).

In contrast to our previous results [42,43,44,45,46,47,48,49], compounds **2a**–**2f** account for the first case where *N*^3^-substituted amidrazones (**1a**–**1f**) react with a cyclic anhydride, exclusively forming *1H*-pyrrole-2,5-dione derivatives, independent of the reaction conditions. Thus, one can suppose that 2,3-dimethylmaleic anhydride (**3**) facilitates just this course of a reaction. In fact, such behaviour is totally different from that observed during the reactions of **1a**–**1f** with maleic anhydride in diethyl ether (at room temperature for 48 h), leading to 3,4-disubstituted 1,2,4-triazol-5-yl β-derivatives of acrylic acid (as proved, for R^1^ = 2-pyridyl and R^2^ = 4-nitrophenyl; CSD refcode: QAHPIZ) [44]. It also differs from the one reported for the reaction of **1d** with maleic anhydride in toluene (at ambient conditions for 10 min), where the respective N^1^-acylamidrazone (with R^1^ = R^2^ = 2-pyridyl) derivative was formed [48].

The structures of **2a**–**2f** were confirmed by elemental analyses, mass spectra and ^1^H, ^13^C NMR spectra with the application of two-dimensional HMQC and HMBC techniques (Supplementary Data, parts C–D, including Figures S25–S48). The ^1^H-^13^C NMR correlation spectroscopy allowed us to assign all ^1^H and ^13^C signals for each **2a**–**2f** molecule exhibiting the presence of two isomeric forms (denoted generally as **A** and **B**; these symbols correspond to the species with the higher and the lower chemical shift of the most deshielded H(8) proton, i.e., NH) in DMSO-d_6_ solutions. The assigned ^1^H and ^13^C NMR chemical shifts for **A** and **B** isomers of **2a**–**2f**, compared to those for the parent amidrazones **1a**–**1f** (Supplementary Data, parts C–D, including Figures S1–S24) are summarized in Tables S8 and S9 (Supplementary Data, part E).

**Figure 3:**
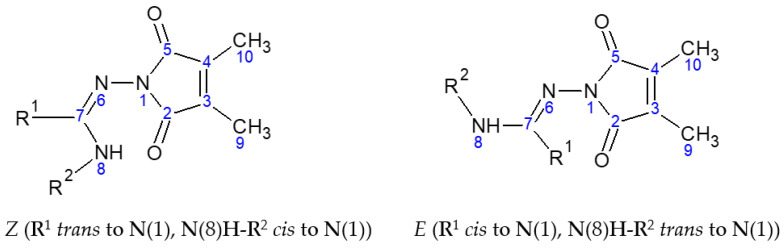
Two hypothetical geometric isomers of **2a**–**2f**.

A detailed description of our attempt to identify and attribute the **A** and **B** forms of **2a**–**2f**, based on the comparative analysis of their ^1^H and ^13^C NMR spectra in solution and partly on the single-crystal X-ray data for **2a** (R^1^ = R^2^ = phenyl) and **2d** (R^1^ = R^2^ = 2-pyridyl), is presented, in the form of comments below Tables S8 and S9. The resulting general conclusion is that **A** and **B** are most likely geometric isomers differing in the position of R^1^ and N(8)H-R^2^ substituents at the C(7) carbon. The lack of rotation around the N(6)=C(7) double bond probably results in *cis*-/*trans*- isomerism: in one stereomer, R^1^ is *trans* to N(1), and N(8)H-R^2^ is *cis* to N(1), whereas in the other one R^1^ is *cis* to N(1) and N(8)H-R^2^ is *trans* to N(1). Taking into account the spatial orientation of the N(1) and N(8) atoms, these are *Z* and *E* stereomers (Figure 3).

The hypothesis of *Z*/*E* isomerism is supported by the fact that, in the solid phase of **2a** or **2d**, where only one stereomer is observed, the crystal structures correspond to such distinct isomeric species: **2a** to *Z* and **2d** to *E*.

### 2.2. X-ray Crystallography

**Figure 4:**
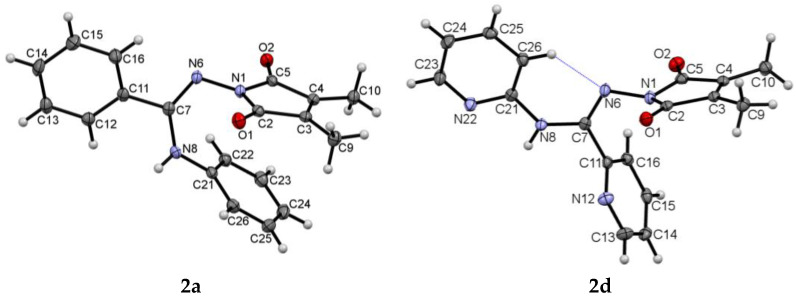
Labelling of atoms and the estimation of their thermal motion parameters as ADPs (50% probability level) in the studied crystals. The dashed line indicates the intramolecular C(26)−H(26)⋅⋅⋅N(6) hydrogen bond.

The molecular plots of **2a** and **2d** with the atom labelling schemes (modification of the general numbering presented in Figure 2) are shown in Figure 4. The selected geometric parameters of **2a** and **2d** are listed in Table S11 (Supplementary Data, part F) together with those for the previously reported, closely related derivative of hexahydro-2*H*-isoindole-1,3-dione (CSD refcode: LUZGUJ) [43,50], corresponding to the already mentioned analogue of **2a**.

The yellow (**2a**) and orange (**2d**) prismatic crystals, suitable for diffraction studies, were grown by recrystallization of the originally synthesized compounds from pure ethanol (99.8%) using the standard solvent evaporation technique.

The single-crystal X-ray diffraction analysis revealed that both **2a** and **2d** crystallize in the same centrosymmetric space group *P*2_1_/*c* with one molecule in the asymmetric part of the unit cell.

Both **2a** and **2d** have the same –N(6)=C(7)–N(8)H– bond system, as exhibited by the bond lengths proving the presence of the N(6)=C(7) double bond and the C(7)–N(8) single bond (Table S11, Supplementary Data, part F). This conclusion is consistent with the ^1^H-^13^C NMR correlation analysis results for all **2a**–**2f** compounds in the DMSO-d_6_ solutions (paragraph 2.1). However, these two molecules adopt different configurations in the solid state: *Z* for **2a** and *E* for **2d** (Figure 3), as confirmed by the corresponding N(1)−N(6)=C(7)−N(8) torsion angles of −13.0(2)° and 171.5(1)°. The latter value is very close to that found in LUZGUJ (173.1(3)^o^), which adopted the *E* geometry in its solid state [43].

The bond lengths in **2a** and **2d** are comparable, being in good agreement with those in LUZGUJ (Table S11, Supplementary Data, part F). This similarity is mainly observed within the N(1)–N(6)=C(7)–N(8) chain, as exemplified by the clear distinction between the N(1)–N(6) and C(7)–N(8) single bonds versus the N(6)=C(7) double bond. However, the C(7)−C(11) single bond is shorter in **2a** and LUZGUJ than in **2d**, whereas the N(8)-C(21)bond is longer in **2a** than in **2d** and LUZGUJ (Table S11, Supplementary Data, part F). On the other hand, in **2a** and **2d,** one can observe the elongation of the N(1)−N(6) and N(6)=C(7) bonds and the shortening of the C(7)−N(8) bond in comparison to those in the eight previously reported N^1^-acylamidrazones derived from **1d** (PAZDIF [48] and RIBVEG, RICGUI, RICHAP, RICHET, RICHIX, RICHOD, and RICHUJ [49] (Table S12, Supplementary Data, part G). These phenomena are well-exemplified when compared to **2a** vs. N^1^-acylamidrazones (as all have the same *Z* geometry, see Table S12) or **2d** vs. N^1^-acylamidrazones (as all contain the same R^1^ = R^2^ = 2-pyridyl substituents). Thus, the respective bond lengths are as follows (in the order: **2a** and **2d** vs. N^1^-acylamidrazones): N(1)−N(6) 1.409(1) Å and 1.419(2) Å vs. N(1)−N(2) 1.371(3)-1.388(3) Å; N(6)=C(7) 1.307(2) Å and 1.301(2) Å vs. N(2)=C(**2a**) 1.284(3)-1.301(5) Å [48,49], reflecting the above relationships as predominant. Moreover, in both **2a** and **2d,** the N(6)=C(7) bonds are longer, and the C(7)−N(8) bonds are shorter than the respective standard N*sp*^2^=C*sp*^2^ (1.28 Å) and C*sp*^2^−NH(−C_ar_) (1.38 Å) bonds [51]. This suggests an extended π-electron delocalization in **2a** and **2d** molecules and can explain the propensity of all **2a**–**2f** compounds to exist in the solutions as various geometric (*Z*/*E*) isomers. The bond angles within the N(1)–N(6)=C(7)–N(8) chain in **2a**, **2d** and LUZGUJ are largely variable (Table S11, Supplementary Data, part F). From these data, it can be seen that there is a greater similarity between **2d** and LUZGUJ (having the same *E* geometry) than between **2a** and LUZGUJ (having the same R^1^ = R^2^ = phenyl substituents). Thus, the spatial arrangement of substituents seems to depend mainly on the molecule configuration. On the other hand, an important role is also played by the type of a substituent at N(1), as the differences between the N(1)-N(6)-C(7) and N(6)-C(7)-N(8) bond angles in **2a** or **2d** and the corresponding ones in already mentioned N^1^-acylamidrazones derived from **1d** (Table S12, Supplementary Data, part F) are even more evident. Generally, both parameters in these N^1^-acylamidrazones are almost always greater than those in **2a** and **2d**: N(1)-N(2)-C(**2a**) 118.1(1)-120.3(3)^o^ vs. N(1)-N(6)-C(7) 113.5(1)^o^ and 112.6^o^; N(2)-C(**2a**)-N(3) 125.1(1)-136.9(2)^o^ vs. N(6)-C(7)-N(8) 128.8(1)^o^ and 120.8(1)^o^, respectively. Hence, the steric crowding of substituents at N(1), N(6) and C(7) causes a change in the valence angles around these atoms.

The 3,4-dimethyl-*1H*-pyrrole-2,5-dione ring system in **2a** and **2d** is essentially planar but with slight distortions, as revealed by the N(1) atom displacement from the N(1)>>C(5) best plane (0.033 Å in **2a** and 0.047 Å in 2d) and the torsion angles inside the pyrrole ring varying from −5.2(1)^o^ to 6.0(1)^o^ (**2a**) and from −7.7(2)^o^ to 8.1(2)^o^ (**2d**) (Table S11, Supplementary Data, part F). The bond lengths and angles in the 3,4-dimethyl-*1H*-pyrrole-2,5-dione moiety of **2a** and **2d** are typical of this ring system; for comparison, seethe mean N(1)-C(2)/C(5) and C(2)-O(1)/C(5)-O(2) bond lengths, as well as the C(2)-N(1)-C(5) bond angles with those in other 1*H*-pyrrole-2,5-dione derivatives [15,24,35,37,39,40] (Table S13 Supplementary Data, part F).

The formal *sp*^2^ hybridization of N(1) in **2a** results in near co-planarity of the N(6) atom with the 3,4-dimethyl-*1H*-pyrrole-2,5-dione ring, as revealed by only a slight N(6) displacement from the N(1)>>C(5) best plane, being 0.059 Å. In contrast, the same parameter in **2d** is much greater, being as much as 0.380 Å due to the partial sp^3^ N(1) hybridization. This difference between N(1) atoms in both compounds is also reflected by the sum of bond angles around this atom, which in **2a** is 359.2(1)^o^, whereas in **2d**, it is only 353.4(1)^o^.

The steric crowding of the 3,4-dimethyl-*1H*-pyrrole-2,5-dione ring system and the R^2^ substituent, as observed in **2a,** results in significant conformational adjustment by the simultaneous rotation around the N(1)−N(6), C(7)−N(8) and N(8)−C(21) single bonds. In consequence, the *1H*-pyrrole-2,5-dione ring in **2a** is significantly twisted with respect to the N(6)−C(7)−N(8) moiety, while in **2d,** it is almost perpendicular, as shown by the dihedral angle between the N(1) >> C(5) best plane and the N(6)−C(7)−N(8) plane, being 64.8° for **2a** and 85.6° for **2d**.

Similarly, in **2a,** the R^1^ and R^2^ substituents are noticeably twisted with respect to the N(6)−C(7)−N(8) moiety, as shown by the dihedral angles between the C(11) >> C(16) best plane or the C(21) >> C(26) best plane and the N(6)−C(7)−N(8) plane, being 29.5° and 61.4°, respectively. In **2d**, the R^1^ substituent is even more twisted, but the R^2^ one is much less twisted, as revealed by the relevant dihedral angles of 74.3^o^ and 10.2^o^. Therefore, the great level of co-planarity of the C(21) >> C(26) and N(6)−C(7)−N(8) moieties in **2d** enables the formation of the intramolecular C(26)−H(26)⋅⋅⋅N(6) hydrogen bond (d(H⋅⋅⋅N) = 2.23 Å, <(C−H⋅⋅⋅N) = 121°) (Figure 4, Table S14, Supplementary Data, part F), resulting in the S(6) ring motif [52].

Finally, in both **2a** and **2d**, the phenyl or 2-pyridyl substituents are almost perpendicular to each other, as shown by the dihedral angle between the C(11) << C(16) and the C(21) << C(26) best planes, which are 88.9° and 81.3°, respectively.

The studied molecules are proton-deficient, as each possesses one HB donor (N(8)−H(8)) and three or five potential HB acceptors (O(1), O(2) and N(6), as well as N(12) and N(22), optionally). The presence of numerous acceptor atoms, aromatic rings and ‘active’ methyl groups stimulates the formation of weak hydrogen bonds. Among the intermolecular interactions involved in the stabilization of **2a** and **2d** crystals, a number of weak C−H⋅⋅⋅O/N/π hydrogen bonds (their full list, including geometric parameters and the symmetry codes, together with the selected C−H⋅⋅⋅C short contacts, is presented in Table S14 (Supplementary Data, part F)) and dipolar C=O⋅⋅⋅C contacts play an important role.

**Figure 5:**
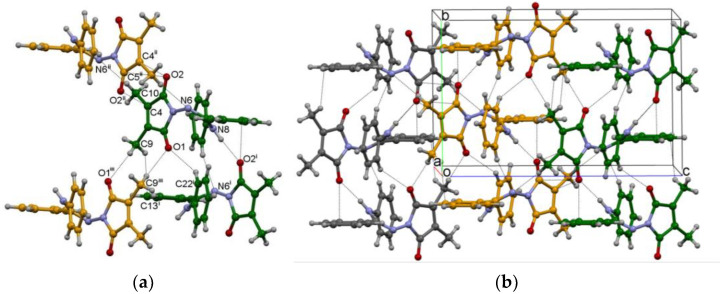
Part of the crystal structure of **2a** showing: (**a**) the molecular environment and main intermolecular interactions (symmetry codes: (i) −*x* + 1, *y* − 1/2, −*z* + 1/2; (ii) −*x* + 1, −*y* + 1, −*z*; (iii) −*x* + 1, −*y*, −*z)*; (**b**) antiparallel, helical chains viewed along the *a* axis. Dashed lines indicate the hydrogen bonds, short C−H⋅⋅⋅O/N/π or C=O⋅⋅⋅C contacts.

**Figure 6:**
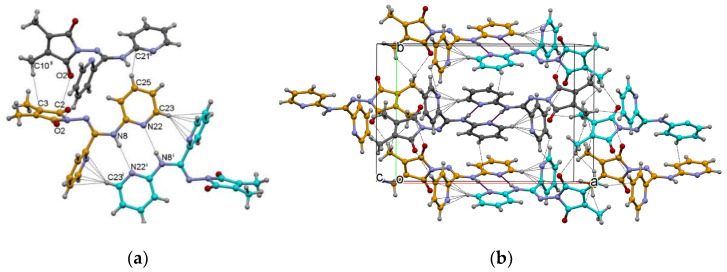
Part of the crystal structure of **2d** showing: (**a**) the molecular environment and main intermolecular interactions (symmetry codes: (i) −*x* + 1, −*y*, −*z*; (ii) *x*, −*y* + 1/2, *z*−1/2); (**b**) crystal packing viewed along the *c* axis. Dashed lines indicate the hydrogen bonds, short C−H⋅⋅⋅O/N/π or C=O⋅⋅⋅C contacts.

Generally, it must be noted that some substantial differences in molecular packing occur between **2a** and **2d** (Figure 5 and Figure 6).

In **2a,** the primary supramolecular motif is hydrogen-bonded chains (Figure 5) parallel to the *b* axis. Within each chain, the adjacent 2_1_-axis-related molecules are connected by strong, directional N(8)−H(8)···O(2) (−*x* + 1, *y* − 1/2, −*z* + ½) hydrogen bonds (Table S14, Supplementary Data, part F); the additional stabilization of the chain motif is provided by weak C(26)−H(26)···N(6) (−*x* + 1, *y* − 1/2, −*z* + 1/2) and C(22)−H(22)···O(1) (−*x* + 1, *y* + 1/2, −*z* + 1/2) hydrogen bonds. The neighbouring, inversion-related and hence antiparallel chains are connected by weak C(9)−H(9b)···O(1) (−*x* + 1, −*y*, −*z*) and C(10)−H(10b)···N(6) (−*x* + 1, −*y* + 1, −*z*) hydrogen bonds, resulting in a three-dimensional architecture. It is noteworthy that apart from the already-mentioned strong N−H···O hydrogen bond, the O(2) atom is also engaged in short C=O···C_ar_ and dipolar C=O···C=O interactions (Figure 5a). Taking into account the geometry of these contacts (*d*_O(2)…C(2#)_ = 2.974(2) Å, *θ*_C(5)-O(2)…C(2#)_ = 146.5°, −*x* + 1, *y* + 1/2, −*z* + 1/2; *d*_O(2)…C(5##)_ = 3.048(2) Å, *θ*_C(5)-O(2)…C(5##)_ = 89.6°, -*x* + 1, -*y* + 1, -*z*), the former can be classified as the ‘edge-on’ C=O···π interactions [53], while the latter represents a classic example of the antiparallel carbonyl−carbonyl contacts [54].

The main forces promoting the self-assembly of molecules in the crystal lattice of **2d** seem to result from hydrogen bonding involving amine and pyridine functions (Figure 6). The presence of the additional N(22) acceptor atom and the *E* configuration enables the adjacent, inversion-related molecules to interact by strong, relatively short (*d*_H(8)…N(22)_ = 2.24(2) Å) N(8)−H(8)···N(22) (−*x* + 1, −*y*, −*z*) hydrogen bonds (Table S14, Supplementary Data, part F), creating the $R_{2}^{2}$ (8) ring motif. The additional stabilization of the resulting dimers is provided via the C−H···π contacts involving the highly polarized C(23)−H(23) group and the pyridyl C(11) << C(16) ring (−*x* + 1, −*y*, −*z*). The directionality of this contact with the C−H vector oriented towards the centre of the aromatic ring and all H···C/N distances below the sum of the van der Waals radii of the respective atoms are worth noting. The interactions linking the dimers into the three-dimensional supramolecular net are numerous weak C−H⋅⋅⋅O/N/π hydrogen bonds (Figure 6b), π-stacking contacts between the overlapping C(21) << C(26) pyridyl rings, and electrostatic C=O⋅⋅⋅π interactions involving the *1H*-pyrrole-2,5-dione system.

### 2.3. Toxic Activity of **2a**–**2f**

The effect of different concentrations of **2a**–**2f** or ibuprofen (as a reference drug) on the viability of PBMCs in 24 h cell culture was studied. Compounds **2a**–**2f** and ibuprofen induced no apoptosis or necrosis of the analyzed cells at low (10 µg/mL) or medium (50 µg/mL) concentrations (data not shown). However, in the highest dose (100 µg/mL), **2a** and **2f** appeared to be slightly toxic (79% and 64% of viable cells, respectively), as shown in Figure S49 (Supplementary Data, part G).

### 2.4. Anti-Inflammatory Activity of **2a**–**2f**

#### 2.4.1. Antiproliferative Activity of **2a**–**2f**

**Figure 7:**
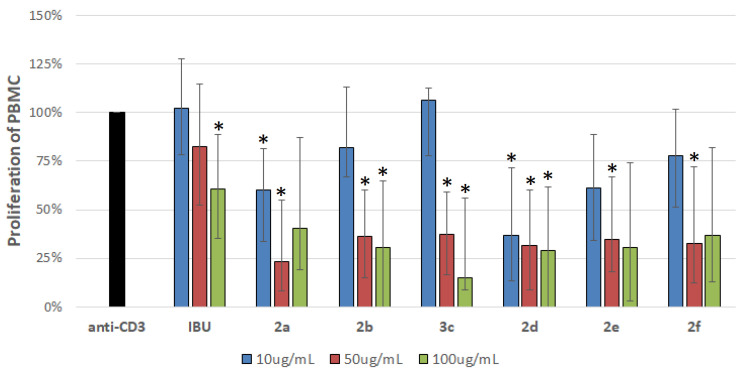
The effect of **2a**–**2f** on the proliferation of PBMCs induced by the soluble anti-CD3 antibody (the results are shown as a percentage of positive control (anti-CD3 antibody alone), with values expressed as medians from four independent experiments and interquartile ranges (Q1–Q3)). * Significant difference compared to positive control (anti-CD3 antibody alone) at *p* < 0.05.

The effect of different concentrations of **2a**–**2f** or ibuprofen on soluble anti-CD3 antibody-induced PBMC proliferation in 72 h cell culture is shown in Figure 7. Generally, all compounds **2a**-**2f** inhibited this process (except for **2c** in the lowest 10 µg/mL dose). Derivative **2d** significantly suppressed PBMC proliferation in each dose (39–77% of inhibition compared to 18–39% for ibuprofen). Significant differences were obtained for compounds **2a–2c** in the selected concentrations, while derivatives **2e** and **2f** inhibited PBMC proliferation only in the medium dose. The strongest inhibitory effect was observed for **2c** in the highest 100 µg/mL concentration (85% inhibition).

#### 2.4.2. The Effects of Compounds **2a**–**2f** on Pro-Inflammatory and Anti-Inflammatory Cytokine Production

**Figure 8:**
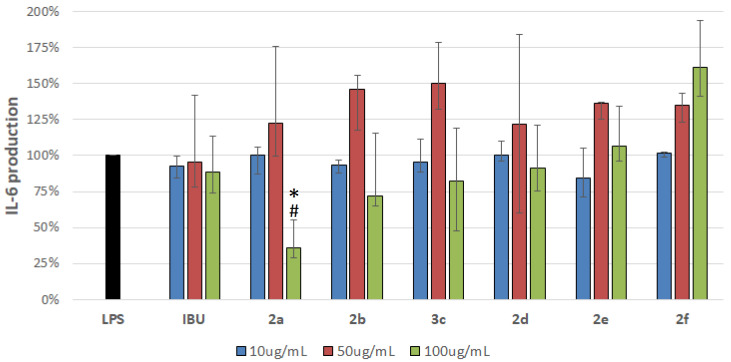
The effect of **2a**–**2f** on the LPS-induced production of IL-6 in PBMC cultures (the results are shown as a percentage of positive control (LPS alone), with values expressed as medians from five independent experiments and interquartile ranges (Q1–Q3)). * Significant difference compared to a positive control (LPS alone) at *p* < 0.01. # Significant difference compared to ibuprofen at *p* < 0.05.

**Figure 9:**
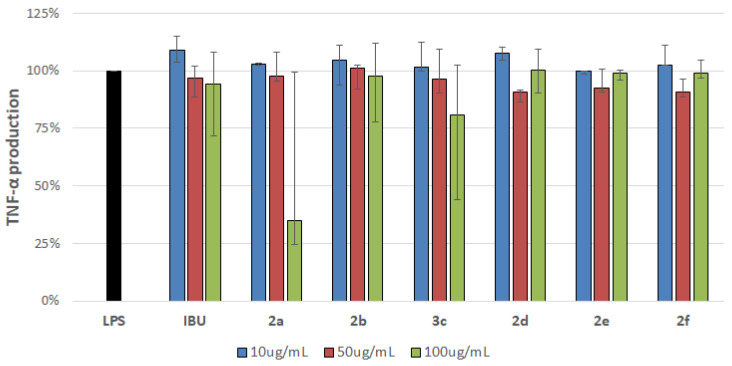
The effect of **2a**–**2f** on the LPS-induced production of TNF-α in PBMC cultures (the results are shown as a percentage of positive control (LPS alone), with values expressed as medians from three independent experiments and interquartile ranges (Q1–Q3).

**Figure 10:**
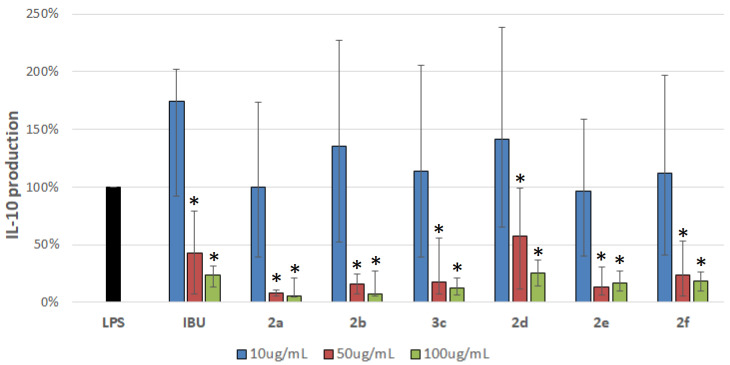
The effect of **2a**–**2f** on the LPS-induced production of IL-10 in PBMC cultures (the results are shown as a percentage of positive control (LPS alone), with values expressed as medians from four independent experiments and interquartile ranges (Q1–Q3)). * Significant difference compared to a positive control (LPS alone) at *p <* 0.05.

The effect of different concentrations of **2a**–**2f** or ibuprofen (as a reference compound) on the LPS-induced production of pro-inflammatory (IL-6 and TNF-α) and anti-inflammatory (IL-10) cytokines in 24 h PBMC culture is presented in Figure 8, Figure 9 and Figure 10. LPS is an endotoxin of Gram-negative bacteria, used extensively for inducing an immune response in vitro. It promotes cytokine production in PBMC cultures, including pro-inflammatory TNF-α and IL-6 and anti-inflammatory IL-10 [33]. TNF-α is the early pro-inflammatory cytokine produced by monocytes, macrophages and lymphocytes in response to inflammatory stimuli, which, together with IL-6, has a broad spectrum of action. Production of TNF-α and IL-6 induces basic symptoms of inflammation such as heat, swelling, redness and pain. In contrast, IL-10, also produced by monocytes, macrophages and lymphocytes (especially type 2 T helper cells, regulatory T and B cells), has anti-inflammatory properties, and LPS could also mediate its production.

The strongest inhibition of pro-inflammatory IL-6 production in LPS-stimulated PBMC culture (Figure 8) was also observed for **2a** in the highest 100 µg/mL dose (64% of inhibition compared to 11% for ibuprofen). At this concentration, **2b** and **2c** exhibited a tendency to inhibit IL-6 production (by 28% and 18%, respectively).

In regards to pro-inflammatory TNF-α production (Figure 9), a strong inhibitory effect in LPS-stimulated PBMC culture was observed for **2a**, only in the highest 100 µg/mL dose (65% inhibition, in comparison to 6% for ibuprofen). In contrast, **2c** produced a 19% inhibition of TNF-α, while **2b** and **2d–f** revealed only small or even negligible impacts in all doses compared to LPS alone or ibuprofen.

Finally, we observed a significant inhibition of anti-inflammatory IL-10 production (Figure 10) for derivatives **2a**–**2c**, **2e** and **2f** in medium (50 µg/mL) or their highest (100 µg/mL) doses (76–92% and 71–95% inhibition, in comparison to 57% and 77% for ibuprofen). However, compound **2d** showed a similar inhibitory profile to ibuprofen (42 and 75% inhibition, respectively). All tested derivatives and ibuprofen elevated IL-10 production in the lowest concentration.

### 2.5. Antibacterial Activity of **2a**–**2f**

The results of MIC determination, presented in Table S15 (Supplementary Data, part H), exhibited the best antibacterial activity for **2a** and **2c** against *Staphylococcus aureus*, as well as for **2d** against *Yersinia enterocolitica* (all MICs = 128 µg/mL). Moreover, **2b** inhibited the growth of *S. aureus*, **2c** inhibited *Y. enterocolitica* and *M. smegmatis*, and **2d** inhibited *Escherichia coli* and *S. aureus* (all MICs = 256 µg/mL). In contrast, **2e** and **2f** had no impact on any studied strains.

## 3. Materials and Methods

### 3.1. General Information

^1^H and ^13^C NMR spectra (including ^13^C DEPT and ^1^H-^13^C HMQC and HMBC) were recorded by a Bruker Avance III 400 MHz NMR spectrometer 295–300 K (Bruker Corporation, Billerica, MA, USA) in DMSO-d_6_. Melting points were measured with the MEL-Temp apparatus (Electrothermal, Stone, UK). Mass spectra were collected on an LCQ Adventage Max (Thermo Finnigan, San Jose, CA, USA). The ^1^H and ^13^C chemical shifts were referenced to TMS, with residual ^1^H and ^13^C solvent signals as primary references (DMSO-d_6_: 2.50 ppm and 40.0 ppm, respectively). Elemental analyses were performed on a CHN Vario MACRO analyzer (Elementar Analysensysteme GmbH, Langenselbold, Germany). The retention factors were determined in reverse-faced plates (nano-silica gel RP-18W on alu foil with fluorescent indicator, (Merck, Darmstadt, Germany)) using a methanol-water (1:1) mixture as eluent.

### 3.2. General Method of Syntheses

*N*^3^-substituted amidrazones **1a**–**1f** were obtained using previously described procedures [41]. A mixture of 1 mmol of **1a**–**1f** and 1 mmol (0.126 g) 2,3-dimethylmaleic anhydride **3** was dissolved in 25 mL of toluene, chloroform or diethyl ether and left for 2–21 days (method A) or dissolved in 25 mL of toluene or chloroform and heated at their respective boiling points for 5 h (method B). The formed **2a**–**2f** solids were collected by filtration at room temperature and purified by crystallization from ethanol.

The detailed reaction conditions (solvent, temperature, time) are given in Tables S2–S7 (Supplementary Data, part B). Products **2a**–**2f** are characterised below. The ^1^H and ^13^C NMR signals are listed as read from one-dimensional spectra, i.e., with no separation of the overlapping resonances and with only the most obvious proton assignments (all others were done further based on the analysis of two-dimensional ^1^H-^13^C HMQC and HMBC spectra; for details see Supplementary Data, parts D-E, including Tables S8 and S9). The NMR spectra of all types for **2a**–**2f** are reproduced in Figures S25–S48 (Supplementary Data, part C).

*N*’-(3,4-dimethyl-2,5-dioxo-2,5-dihydro-1*H*-pyrrol-1-yl)-*N*-phenylbenzene-carboximidamide **(2a**)—yellow crystals, yield: 91%, m.p. 152–154 °C. ^1^H NMR (DMSO-d_6_, 400 MHz): δ 9.54 (s, 0.6H)-NH, 9.23 (s, 0.4H)-NH, 6.74–7.80 (m, 10H)-all phenyl protons in R^1^, R^2^, 1.87 (s, 2.4H)-CH_3_, 1.76 (s, 3.6H)-CH_3_. ^13^C NMR (DMSO-d_6_, 400 MHz): δ 169.4, 168.9, 168.5, 162.8, 140.6, 140.4, 136.2, 135.8, 133.8, 133.5, 131.2, 130.3, 129.6, 3 × 128.9, 128.7, 127.5, 124.0, 123.4, 123.5, 121.0, 9.0, 8.9. Anal. Calcd. for C_19_H_17_N_3_O_2_: C, 71.46; H, 5.37; N, 13.16%. Found: C, 71.50; H, 5.35; N, 12.97%. MS (*m/z*): 319; R_f_ = 0.29 (methanol:water 1:1).

*N*’-(3,4-dimethyl-2,5-dioxo-2,5-dihydro-1*H*-pyrrol-1-yl)-*N*-phenylpyridine-2-carboximidamide (**2b**)—yellow crystals, yield: 95%, m.p. 177–179 °C. ^1^H NMR (DMSO-d_6_, 400 MHz): δ 9.57 (s, 0.4H)-NH, 9.50 (s, 0.6H)-NH, 6.76–8.61 (m, 9H)-all 2-pyridyl/phenyl protons in R^1^, R^2^, 1.77 (s, 2.4H)-CH_3_, 1.73 (s, 3.6H)-CH_3_. ^13^C NMR (DMSO-d_6_, 400 MHz): δ 169.2, 168.2, 165.5, 158.5, 151.6, 151.1, 149.7, 149.0, 140.4, 139.2, 137.8, 137.2, 135.9, 135.7, 129.0, 128.4, 126.2, 125.3, 124.7, 124.4, 124.0, 123.5, 123.4, 120.9, 8.9, 8.8. Anal. Calcd. for C_18_H_16_N_4_O_2_: C, 67.49; H, 5.03; N, 17.49%. Found: C, 67.31; H, 5.17; N, 17.40%. MS (*m/z*): 320; R_f_ = 0.34 (methanol:water 1:1).

*N*’-(3,4-dimethyl-2,5-dioxo-2,5-dihydro-1*H*-pyrrol-1-yl)-*N*-phenylpyridine-4-carboximidamide **(2c)**—yellow crystals, yield: 92%, m.p. 208–211 °C. ^1^H NMR (DMSO-d_6_, 400 MHz): δ 9.71 (s, 0.6H)-NH, 9.46 (s, 0.4H)-NH, 6.76–8.65 (m, 9H)-all 4-pyridyl/phenyl protons in R^1^, R^2^, 1.87 (s, 2.4H)-CH_3_, 1.77 (s, 3.6H)-CH_3_. ^13^C NMR (DMSO-d_6_, 400 MHz): δ 169.3, 168.6, 166.2, 160.9, 150.4, 150.2, 2 × 141.3, 140.2, 139.6, 136.4, 136.2, 2 × 129.1, 124.6, 123.9, 2 × 123.7, 122.2, 121.0, 9.0, 8.9. Anal. Calcd. for C_18_H_16_N_4_O_2_: C, 67.49; H, 5.03; N 17.49%. Found: C, 67.78; H, 4.95; N, 17.44%. MS (*m/z*): 320; R_f_ = 0.32 (methanol:water 1:1).

*N*’-(3,4-dimethyl-2,5-dioxo-2,5-dihydro-1*H*-pyrrol-1-yl)-*N*-(pyridin-2-yl)pyridine-2-carboximidamide **(2d)**—orange crystals, yield: 67%, m.p. 180–183 °C. ^1^H NMR (DMSO-d_6_, 400 MHz): δ 9.95 (s, 0.2H)-NH, 9.84 (s, 0.8H)-NH, 6.80–8.58 (m, 8H)-all 2-pyridyl protons in R^1^, R^2^, 1.88 (s, 4.8H)-CH_3_, 1.78 (s, 1.2H)-CH_3_. ^13^C NMR (DMSO-d_6_, 400 MHz): δ 168.9, 168.4, 164.8, 160.3, 154.0, 152.7, 152.4, 150.7, 149.7, 2 × 148.6, 147.6, 2 × 137.8, 2 × 137.4, 136.4, 136.2, 125.5, 125.0, 2 × 124.3, 119.4, 118.6, 115.3, 115.2, 2 × 9.0. Anal. Calcd. for C_17_H_15_N_5_O_2_: C, 63.54; H, 4.71; N, 21.79%. Found: C, 63.52; H, 4.70; N, 21.86%. MS (*m/z*): 321; R_f_ = 0.39 (methanol:water 1:1).

*N*’-(3,4-dimethyl-2,5-dioxo-2,5-dihydro-1*H*-pyrrol-1-yl)-*N*-(4-methylphenyl)pyridine-2-carboximidamide **(2e)**—yellow crystals, yield: 84%, m.p. 199–201 °C. ^1^H NMR (DMSO-d_6_, 400 MHz): δ 9.47 (s, 0.2H)-NH, 9.42 (s, 0.8H)-NH, 6.65–8.59 (m, 8H)-all 2-pyridyl/phenyl protons in R^1^, R^2^, 2.26 (s, 0.6H)-CH_3_, 2.18 (s, 2.4H)-CH_3_, 1.76 (s, 1.2H)-CH_3_, 1.72 (s, 4.8H)-CH_3_. ^13^C NMR (DMSO-d_6_, 400 MHz): δ 169.2, 168.6, 165.4, 158.6, 151.7, 151.1, 149.6, 148.9, 137.9, 137.8, 137.2, 136.4, 135.9, 135.6, 134.3, 132.4, 129.4, 128.9, 126.1, 125.2, 124.7, 123.9, 123.4, 120.9, 20.9, 20.8, 8.9, 8.7. Anal. Calcd. for C_19_H_18_N_4_O_2_: C, 68.25; H, 5.43; N, 16.76%. Found: C, 68.58; H, 5.52; N, 16.78%. MS (*m/z*): 334; R_f_ = 0.28 (methanol:water 1:1).

*N*’-(3,4-dimethyl-2,5-dioxo-2,5-dihydro-1*H*-pyrrol-1-yl)-*N*-(4-nitrophenyl)pyridine-2-carboximidamide **(2f)**—yellow crystals, yield: 70%, m.p. 220–224 °C. ^1^H NMR (DMSO-d_6_, 400 MHz): δ 10.26 (s, 0.6H)-NH, 9.94 (s, 0.4H)-NH, 6.79–8.66 (m, 8H)-all 2-pyridyl/phenyl protons in R^1^, R^2^, 1.89 (2.4H)-CH_3_, 1.80 (3.6H)-CH_3_. ^13^C NMR (DMSO-d_6_, 400 MHz): δ 168.8, 168.2, 165.2, 159.4, 150.8, 150.7, 149.9, 149.4, 147.0, 146.5, 142.3, 142.2, 138.1, 137.4, 136.7, 136.3, 126.5, 125.7, 125.3, 125.1, 124.7, 123.7, 121.4, 120.4, 9.0, 8.6. Anal. Calcd. for C_18_H_15_N_5_O_4_: C, 59.18; H, 4.14; N, 19.17%. Found: C, 59.22; H, 4.54; N, 19.16%. MS (*m/z*): 365; R_f_ = 0.24 (methanol:water 1:1).

### 3.3. Crystal Structure Determination

Single-crystal X-ray diffraction data for **2a** and **2d** were collected using the Oxford Diffraction Xcalibur CCD diffractometer with the graphite-monochromated MoKα radiation (λ= 0.7107 Å). The standard data collection temperature was 100 K, which was maintained using the Oxford Cryosystems nitrogen gas-flow device (Cobra Plus). The CRYSALIS [55] suite of programs was used for data collection, cell refinement and data reduction. A multi-scan absorption correction was applied. The structures were solved by direct methods implemented in SHELXS-97 [56] and refined with the SHELXL-97 program [56] (both operating with WinGX) [57]. All non-H atoms were refined with the anisotropic displacement parameters. The H atoms attached to carbon were positioned geometrically and refined using the riding model with U_iso_(H) = 1.2−1.5 U_eq_(C). The amine H(8) atoms were found in the Fourier maps and refined with the isotropic displacement parameters. CCDC 2,059,216 (**2a**) and 2,059,217 (**2d**) contain the supplementary crystallographic data for this paper. A copy of the data can be obtained free of charge via http://www.ccdc.cam.ac.uk/conts/retrieving.html (accessed on 30 March 2022) or upon application to CCDC, 12 Union Road, Cambridge CB21EZ, UK (fax: +44 1223-336-033; e-mail: deposit@ccdc.cam.ac.uk).

Crystal data for **2a** (C_19_H_17_N_3_O_2_, *M* = 319.36 g⋅mol^−1^): monoclinic, space group *P*2_1_/c, *a* = 10.2634(4) Å, *b* = 10.2227(4) Å, *c* = 15.6644(6) Å, *β* = 98.593(3)°, *V* = 1625.1(1) Å^3^, *Z* = 4, *D_calc_* = 1.305 g⋅cm^−3^, *μ* = 0.087 mm^−1^, 11,798 refl. measured (2.63 ≤ θ ≤ 27.48°), 3726 unique (*R*_int_ = 0.0379), GOF = 1.022. The final *R*_1_ = 0.0418 (*I* > 2*σ*(*I*)) and *wR*_2_ = 0.1032 (all data).

Crystal data for **2d** (C_17_H_15_N_5_O_2_, *M* = 321.34 g⋅mol^−1^): monoclinic, space group *P*2_1_/c, *a* = 16.4300(9) Å, *b* = 11.2891(5) Å, *c* = 8.3230(4) Å, *β* = 92.472(4)°, *V* = 1542.3(1) Å^3^, *Z* = 4, *D_calc_* = 1.384 g⋅cm^−3^, *μ* = 0.095 mm^−1^, 13,934 refl. measured (3.04 ≤ θ ≤ 27.48°), 3542 unique (*R*_int_ = 0.0425), GOF = 1.029. The final *R*_1_ = 0.0444 (*I* > 2*σ*(*I*)) and *wR*_2_ = 0.1044 (all data).

### 3.4. Peripheral Blood Mononuclear Cell Preparation

After informed consent, fresh blood (18 mL) was obtained from five healthy donors at the Occupational Medicine Clinic located in Dr. Antoni Jurasz University Hospital in Bydgoszcz, Poland.

Peripheral blood mononuclear cells (PBMCs) were isolated by density gradient centrifugation (Lymphosep, BioWest, Nuaille, France). The cells were then washed twice in phosphate-buffered saline (PBS, Biomed Lublin, Poland) and re-suspended in PBS (10–20 cell/mL) or RPMI 1640 medium (Biomed Lublin, Lublin, Poland) supplied with 5% pooled, heat-inactivated AB Rh+ human serum (1 × 10^6^ cell/mL). After isolation, trypan blue assessed cell viability, which was above 90%. The **2a**–**2f** compounds and racemic ibuprofen (Sigma-Aldrich, Burlington, MA, USA) were initially dissolved in dimethyl sulfoxide (DMSO, Sigma-Aldrich), then in a culture medium to obtain concentrations of 10, 50 and 100 μg/mL. The maximum concentration of DMSO in the individual assay was <0.5% and demonstrated no cell lethality.

### 3.5. In Vitro Toxic Effects on PBMCs by APC Annexin V and Propidium Iodide Staining Assay and Flow Cytometry

The effects of compounds **2a**–**2f** on cell viability were studied in PBMCs culture by flow cytometry. The cells (1 × 10^6^ cells/mL) were seeded in 24-well polypropylene, non-adherent plates (Cytogen, Zgierz, Poland). After that, increasing amounts of 2a–2f in DMSO were added to the cells and incubated for 24 h at 37 °C at 5% CO_2_ conditions. The final concentrations of **2a**–**2f** were 10, 50 and 100 µg/mL. Control samples contained DMSO or ibuprofen. After stimulation, the tubes were centrifuged at 400 g at 4 °C for 5 min and washed once with PBS. Then, the cells were stained with allophycocyanin-conjugated Annexin V (APC Annexin V) and propidium iodide (PI) (both from BD Pharmingen, San Diego, CA, USA) in accordance with the manufacturer’s manual. A total of 10,000 cells were acquired on an FACSCanto II flow cytometer (Becton Dickinson, Franklin Lakes, NJ, USA) and analyzed with FlowJo software (v 7.6.1, Tree Star, Ashland, OR, USA).

### 3.6. Anti-Inflammatory Activity

#### 3.6.1. In Vitro Antiproliferative Effects by VPD-450 Staining Assay and Flow Cytometry

Antiproliferative effects were examined by flow cytometry in BD Horizon Violet Proliferation Dye 450 (VPD450, BD Pharmingen)-labeled PBMCs. Flow cytometry assay was employed to find the cytotoxic potential of compounds **2a**–**2f** on the proliferation of soluble anti-human CD3 monoclonal antibody (mouse IgG2a, clone OKT3, Sigma-Aldrich)-induced PBMCs. Briefly, freshly isolated PBMCs at a concentration of 10–20 × 10^6^ cells/mL in PBS were labeled for 11 min with VPD450 (1uM) at 37 °C. The VPD450 labeling reaction was terminated with complete media containing 10% fetal bovine serum (FBS) and then re-suspended at a 1 × 106 cells/mL concentration in 5% FBS/RPMI1640. VPD450-stained cells were cultured in conical polypropylene tubes (BD Bioscience) for 72 h in 37 °C at 5% CO_2_ atmosphere with anti-CD3 (1 µg/mL, positive control) and/or increasing concentration of **2a**–**2f** in DMSO (10, 50 and 100 μg/mL). Control samples contained DMSO or ibuprofen. The culture tubes were centrifuged at 400× *g* at RT for 5 min, washed once in PBS, and 10,000 cells from every sample were acquired on a FACSCanto II flow cytometer (Becton Dickinson) and analyzed with FlowJo software (v 7.6.1, Tree Star, Ashland, OR, USA).

#### 3.6.2. In Vitro Anti- and Proinflammatory Cytokine Production Effect by the Enzyme-Linked Immunosorbent Assay (ELISA)

The assay was conducted as described earlier [45]. PBMCs were cultured with lipopolysaccharide (LPS, from *E. coli*, O55:B5, (Sigma-Aldrich), 1 μg/mL, positive control) and/or increasing concentrations of **2a**–**2f** compounds in DMSO (10, 50 and 100 μg/mL) for 24 h in 24-well polypropylene, non-adherent plates (Cytogen). Control cultures contained DMSO or ibuprofen. According to the manufacturer’s instructions, the cytokine levels (TNF-α, IL-6 and IL-10) were measured by means of commercially available ELISA kits (DuoSet, BD Bioscience). The samples were analyzed with iEMS Reader MF (Labsystems, Vantaa, Finland). The contents of analyzed cytokines were calculated by Genesis version 2.2 software.

### 3.7. Antibacterial Activity

The broth microdilution method determined the minimum inhibitory concentration (MIC), defined as the lowest concentration of the compounds **2a–2f** that inhibited bacterial growth. The strains used in the study: *Staphylococcus aureus* ATCC 25923, *Enterococcus faecalis* ATCC 29212, *Escherichia coli* ATCC 25922 and *Pseudomonas aeruginosa* ATCC 27853 came from the American Type Culture Collection (Manassas, VA, USA) and are the recommended reference strains for antibiotic susceptibility testing. Other strains, including *Micrococcus luteus, Yersinia enterocolitica* O3, *Mycobacterium smegmatis* and *Nocardia corallina* (currently *Rhodococcus* sp.), came from environmental sources, are deposited in the Department of Genetics and Microbiology collection, and have been used by us in previously published experiments [45,46].

Compounds **2a**–**2f** were dissolved in DMSO, diluted tenfold in Mueller–Hinton broth (MHB) to the concentration of 1.024 mg/mL, and then serially diluted in MHB to concentrations ranging from 512 µg/mL to 0.25 µg/mL.

The wells were inoculated with bacterial cultures to the final concentration of 10^4^ colony-forming units (CFU) per mL. Bacterial growth was assayed by measuring optical density at OD 550 nm after 18 h incubation at 37 °C. The wells containing only MHB and 2.5% dimethyl sulfoxide were applied as a negative control. All MIC determinations were carried out in triplicates.

### 3.8. Data Analysis

Data were analyzed in Statistica 13.3 software (StatSoft, Cracow, Poland) and graphed in Excel 2016 (Microsoft, Redmond, WA, USA). All *p*-values represent the nonparametric Mann–Whitney U test.

## 4. Conclusions

Six new *1H*-pyrrole-2,5-dione derivatives **2a**–**2f** were selectively obtained in reactions of various *N*^3^-substituted amidrazones with 2,3-dimethylmaleic anhydride. In contrast to the previous results, no linear or 1,2,4-triazole products or by-products were formed.

The comparative analysis of the ^1^H-^13^C NMR spectra of **2a**–**2f** to those for the parent amidrazones **1a**–**1f** demonstrated that they appeared in DMSO-d_6_ as a mixture of distinct **A** and **B** forms, being most likely geometric *Z* and *E* isomers, respectively. This is consistent with the results of single-crystal X-ray diffraction studies of **2a** and **2d**, which revealed the respective *Z* and *E* isomers in their solid phase.

All studied compounds possess anti-inflammatory properties by inhibiting PBMC proliferation (especially **2c** and **2d**) as well as TNF-α and IL-6 production (only **2a**). Additionally, **2a** and **2c** exhibit antibacterial activity, particularly against *S. aureus*.

## Data Availability

Data are available from the authors.

## Supplementary Materials

The following supporting information can be downloaded at: https://www.mdpi.com/article/10.3390/molecules27092891/s1. A. Table S1: 1H, 13C NMR and single-crystal X-ray data for selected derivatives of 1H-pyrrole-2,5-dione. B. Tables S2–S7: Details of syntheses of 2a–2f. C. 1H and 13C NMR data of 1a–1f and 2a–2f (A, B isomers). Figure S1. 1H NMR spectrum of 1a (in DMSO-d6). Figure S2. 13C NMR spectrum of 1a (in DMSO-d6). Figure S3. 1H-13C HMQC spectrum of 1a (in DMSO-d6). Figure S4. 1H-13C HMBC spectrum of 1a (in DMSO-d6). Figure S5. 1H NMR spectrum of 1b (in DMSO-d6). Figure S6. 13C NMR spectrum of 1b (in DMSO-d6). Figure S7. 1H-13C HMQC spectrum of 1b (in DMSO-d6). Figure S8. 1H-13C HMBC spectrum of 1b (in DMSO-d6). Figure S9. 13C NMR spectrum of 1c (in DMSO-d6). Figure S10. 1H-13C HMQC spectrum of 1c (in DMSO-d6). Figure S11. 1H-13C HMBC spectrum of 1c (in DMSO-d6). Figure S12. 1H-13C HMBC spectrum of 1c (in DMSO-d6). Figure S13. 1H NMR spectrum of 1d (in DMSO-d6). Figure S14. 13C NMR spectrum of 1d (in DMSO-d6). Figure S15. 1H-13C HMQC spectrum of 1d (in DMSO-d6). Figure S16. 1H-13C HMBC spectrum of 1d (in DMSO-d6). Figure S17. 1H NMR spectrum of 1e (in DMSO-d6). Figure S18. 13C NMR spectrum of 1e (in DMSO-d6). Figure S19. 1H-13C HMQC spectrum of 1e (in DMSO-d6). Figure S20. 1H-13C HMBC spectrum of 1e (in DMSO-d6). Figure S21. 1H NMR spectrum of 1f (in DMSO-d6). Figure S22. 13C NMR spectrum of 1f (in DMSO-d6). Figure S23. 1H-13C HMQC spectrum of 1f (in DMSO-d6). Figure S24. 1H-13C HMBC spectrum of 1f (in DMSO-d6). Figure S25. 1H NMR spectrum of 2a (in DMSO-d6). Figure S26. 13C NMR spectrum of 2a (in DMSO-d6). Figure S27. 1H-13C HMQC spectrum of 2a (in DMSO-d6). Figure S28. 1H-13C HMBC spectrum of 2a (in DMSO-d6). Figure S29. 1H NMR spectrum of 2b (in DMSO-d6). Figure S30. 13C NMR spectrum of 2b (in DMSO-d6). Figure S31. 1H-13C HMQC spectrum of 2b (in DMSO-d6). Figure S32. 1H-13C HMBC spectrum of 2b (in DMSO-d6). Figure S33. 1H NMR spectrum of 2c (in DMSO-d6). Figure S34. 13C NMR spectrum of 2c (in DMSO-d6). Figure S35. 1H-13C HMQC spectrum of 2c (in DMSO-d6). Figure S36. 1H-13C HMBC spectrum of 2c (in DMSO-d6). Figure S37. 1H NMR spectrum of 2d (in DMSO-d6). Figure S38. 13C NMR spectrum of 2d (in DMSO-d6). Figure S39. 1H-13C HMQC spectrum of 2d (in DMSO-d6). Figure S40. 1H-13C HMBC spectrum of 2d (in DMSO-d6). Figure S41. 1H NMR spectrum of 2e (in DMSO-d6). Figure S42. 13C NMR spectrum of 2e (in DMSO-d6). Figure S43. 1H-13C HMQC spectrum of 2e (in DMSO-d6). Figure S44. 1H-13C HMBC spectrum of 2e (in DMSO-d6). Figure S45. 1H NMR spectrum of 2f (in DMSO-d6). Figure S46. 13C NMR spectrum of 2f (in DMSO-d6). Figure S47. 1H-13C HMQC spectrum of 2f (in DMSO-d6). Figure S48. 1H-13C HMBC spectrum of 2f (in DMSO-d6). D. 1H and 13C NMR data of 1a–1f and 2a–2f (A, B isomers). E. Table S8. 1H NMR chemical shifts for A and B forms of 2a–2f, and 1a–1f (in italics), in DMSO-d6 (δ1H, ppm), at 298 K. Table S9. 13C NMR chemical shifts for A and B forms of 2a–2f, and 1a-1f (in italics), in DMSO-d6 (δ1H, ppm), at 298 K. Table S10: 13C NMR chemical shifts for selected N(1)-amino, N(1)-amido and N(1)-imino derivatives of 1H-pyrrole-2,5-diones. F. Table S11: Selected bond lengths (Å), bond angles (°) and torsion angles (°) in the molecules 2a and 2d, and the closely related, CSD-reported X-ray structure LUZGUJ. Table S12: Selected bond lengths in the aliphatic chain of 2a, 2d and of the X-ray reported N1-acylamidrazones. Table S13: Selected bond lengths and angles in the 1H-pyrrole-2,5-dione moiety of 2a, 2d and some other X-ray reported 1H-pyrrole-2,5-dione derivatives. Table S14: Geometries of hydrogen bonds and selected short contacts in the crystals of 2a and 2d. G. Figure S49: The effect of ibuprofen (IBU) and 2a–2f at 100 µg/mL dose on the cell viability in PBMC cultures. H. Table S15: MIC values of 2a–2f, ampicillin and tetracycline against the tested bacterial strains.

## Sample Availability

Samples of the compounds **2a–2f** are available from the authors.
