# Supplementary material for: Synthesis and Structural Study of Amidrazone Derived Pyrrole-2,5-Dione Derivatives: Potential Anti-Inflammatory Agents
Source: Molecules. 2022 Apr 30;27(9):2891. doi: 10.3390/molecules27092891 (PMC9099820; doi:10.3390/molecules27092891)
Supplement: Supplementary file 1 [file molecules-27-02891-s001.zip › molecules-1685329-SI.pdf]

Synthesis and structural study of pyrrole-2,5-dione derivatives: potential anti-inflammatory agents

Renata Paprocka*, Leszek Pazderski, Liliana Mazur, Małgorzata Wiese-Szadkowska, Jolanta Kutkowska, Michalina Nowak, Anna Helmin-Basa*

*Correspondence: renata.bursa@cm.umk.pl and a.helmin-basa@cm.umk.pl.

TABLE OF CONTENTS:

A. REVIEW OF ^{1}H, ^{13}C NMR AND SINGLE CRYSTAL X-RAY DATA FOR SELECTED DERIVATIVES OF 1*H*-PYRROLE-2,5-DIONE
B. DETAILS OF SYNTHESES OF 2a-2f
C. ^{1}H AND ^{13}C NMR SPECTRA OF 1a-1f and 2a-2f (A, B ISOMERS)
D. ^{1}H AND ^{13}C NMR DATA OF 1a-1f and 2a-2f (A, B ISOMERS)
E. SUMMARY AND DISCUSSION OF ^{1}H AND ^{13}C NMR CHEMICAL SHIFTS FOR 2a-2f (A AND B ISOMERS), COMPARED TO THOSE OF 1a-1f
F. X-RAY STRUCTURES OF 2a AND 2d
G. TOXICITY OF 2a-2f IN PBMC CULTURE
H. ANTIBACTERIAL ACTIVITY OF 2a-2f

PART A. REVIEW OF ^{1}H, ^{13}C NMR AND SINGLE CRYSTAL X-RAY DATA FOR SELECTED DERIVATIVES OF 1*H*-PYRROLE-2,5-DIONE

Table S1. ^{1}H, ^{13}C NMR and single crystal X-ray data for selected derivatives of 1*H*-pyrrole-2,5-dione

**Class of chemicals: 1*H*-pyrrole-2,5-diones (L)**	Compound	^{1}H and ^{13}C NMR data	Single crystal X-ray data [a]
1H-pyrrole-2,5-dione (L^{1}) and its derivatives	L^{1}	[A,B]	TEKQAB [C]
	1-methyl-L^{1}	[D,E,F]	
	1,1-dimethylamino-L^{1}	[G]	
	1-phenylamino-L^{1}	[G,H]	
	1-(4-methylphenyl)amino-L^{1}	[G]	
	1-(4-methoxyphenyl)amino-L^{1}	[G]	
	1-(4-bromophenylamino)-L^{1}	[I]	CARHAF [I]
	1-(piperidyn-1-yl)-L^{1}	[G]	
	1-(morpholin-4-yl)-L^{1}	[G]	
	1-(4-methylpiperazin-1-yl)-L^{1}	[G]	
	1,1-diphenylamino-L^{1}	[G]	
	1-(*E*-4-phenylbut-3-en-2-ylideneimino)-L^{1}	[J]	
	1-(*E*-4-(2-fluorophenyl)but-3-en-2-ylideneimino)-L^{1}	[J]	

	1-(*E*-4-(3,5-bis(trifluoromethyl)phenyl)but-3-en-2-ylidene)imino)-L[1]	[J]	
	1-(*E*-4-(2,4-dichlorophenyl)but-3-en-2-ylideneimino)-L[1]	[J]	
	1-(*E*-4-(4-nitrophenyl)but-3-en-2-ylideneimino)-L[1]	[J]	
3-methyl-1H-pyrrole-2,5-dione (L[2]) and its derivatives	L[2]	[K,L,M,N,O]	
	1-methyl-L[2] [b]	[K,N,P]	
	1-phenylamino-L[2]	[Q]	COXYEV [Q]
	L[2]-L[2] [c]	[Q]	TUBBID [Q]
	1-(*E*-4-phenylbut-3-en-2-ylideneimino)-L[2]	[J]	
	1-(*E*-4-(3,5-bis(trifluoromethyl)phenyl)but-3-en-2-ylidene)imino)-L[2]	[J]	
	1-(*E*-4-(2,4-dichlorophenyl)but-3-en-2-ylideneimino)-L[2]	[J]	
3,4-dimethyl-1H-pyrrole-2,5-dione (L[3]) and its derivatives	L[3]	[B,K,R,S]	
	1-methyl-L[3] [d]	[K]	
	1-phenylamino-L[3]	[Q]	COXYUL [Q]
	L[3]-L[3] [e]	[Q]	COXYAR [Q]<br>COXYAR01 [Q]
	1,1-dimethylamino-L[3]	[T]	
3,4-diethyl-1H-pyrrole-2,5-dione (L[4]) and its derivatives/analogues	L[4]	[U]	
3,4-diphenyl-1H-pyrrole-2,5-dione (L[5]) and its derivatives/analogues	L[5]<br>L[5] ·1-methylpyrrolidyn-2-one	[R,V]	 NIXRUJ [W]
	1-methyl-L[5]	[X,Y,Z,AA]	
	1-benzamido-L[5]	[BB]	
	1-(4-methoxybenzamido)-L[5]	[BB]	
	1-(4-bromobenzamido)-L[5]	[BB]	KUQRIZ [BB]
	1-(4-nitrobenzamido)-L[5]	[BB]	
	1-methoxycarbonylamino-3,4-bis(4-nitrophenyl)-*1H*-pyrrole-2,5-dione		TAJSIG [CC]

[a] Reference codes from Cambridge Structure Database [DD]
[b] Systematic name of this species: 1,3-dimethyl-1*H*-pyrrole-2,5-dione
[c] Systematic name of this dimeric species: 3,3'-dimethyl-1,1'-bipyrrole-2,2',5,5'-tetraone
[d] Systematic name of this species: 1,3,4-trimethyl-1*H*-pyrrole-2,5-dione
[e] Systematic name of this dimeric species: 3,3',4,4'-tetramethyl-1,1'-bipyrrole-2,2',5,5'-tetraone

PART B. DETAILS OF SYNTHESES OF 2a-2f

Table S2. The details of synthesis of **2a** ($R^1 = R^2$ = phenyl)

**Solvent**	**temp.**	**yield**	**m.p.**
toluene	r.t. 14d	59%	152-154 °C
toluene	b.p. 5h	91%	152-154 °C
chloroform	r.t. 4d	84%	152-155 °C
chloroform	b.p. 5h	71%	153-154 °C
diethyl ether	r.t. 2d	53%	153-155 °C

Table S3. The details of synthesis of **2b** (R^1 = 2-pyridyl, R^2 = phenyl)

**solvent**	**temp.**	**yield**	**m.p.**
Toluene	r.t. 10d	69%	174-177 °C
toluene	b.p. 5h	95%	177-179 °C
chloroform	r.t. 20d	84%	176-178 °C
chloroform	b.p. 5h	78%	177-179 °C
diethyl ether	r.t. 21d	21%	173-175 °C

Table S4. The details of synthesis of **2c** (R^1 = 4-pyridyl, R^2 = phenyl)

**Solvent**	**temp.**	**yield**	**m.p.**
toluene	r.t. 11d	73%	206-215 °C
toluene	b.p. 5h	64%	210-215 °C
chloroform	r.t. 11d	84%	214-217 °C
chloroform	b.p. 5h	92%	208-211 °C
diethyl ether	r.t. 12d	74%	214-216 °C

Table S5. The details of synthesis of **2d** ($R^1 = R^2$ = 2-pyridyl)

**Solvent**	**temp.**	**yield**	**m.p.**
diethyl ether	r.t. 11d	67%	180-183°C

Table S6. The details of synthesis of **2e** (R^1 = 2-pyridyl, R^2 =4-methylphenyl)

**solvent**	**temp.**	**yield**	**m.p.**
toluene	r.t. 11d	39%	198-201 °C
toluene	b.p. 5h	76%	195-200 °C
chloroform	r.t. 11d	84%	199-201 °C
chloroform	b.p. 5h	68%	198-201 °C
diethyl ether	r.t. 14d	51%	198-201 °C

Table S7. The details of synthesis of **2f** (R^1 = 2-pyridyl, R^2 =4-nitrophenyl)

**solvent**	**temp.**	**Yield**	**m.p.**
toluene	r.t. 7d	38%	206-211 °C
toluene	b.p. 5h	35%	216-222 °C
chloroform	r.t. 7d	75%	218-223 °C
chloroform	b.p. 5h	70%	220-224 °C
diethyl ether	r.t. 11d	18%	219-223 °C

r.t. - room temperature, b.p. – boiling point

PART C. ^{1}H AND ^{13}C NMR SPECTRA OF 1a-1f and 2a-2f (A, B ISOMERS)

The standard ^{1}H and ^{13}C NMR spectra, as well as the ^{1}H-^{13}C HMQC and ^{1}H-^{13}C HMBC two-dimensional ones of **1a**-**1f** and **2a**-**2f** compounds, are shown below at Figures S1-S48.

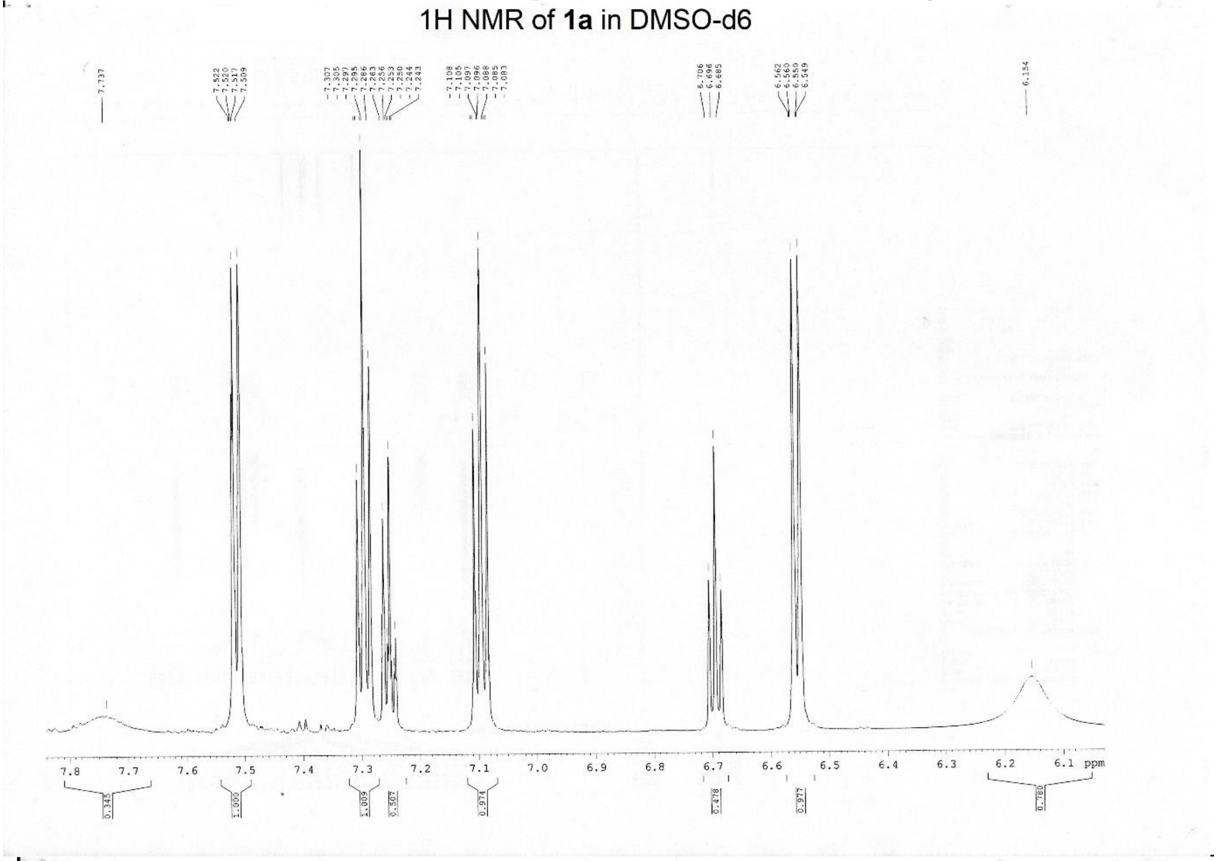

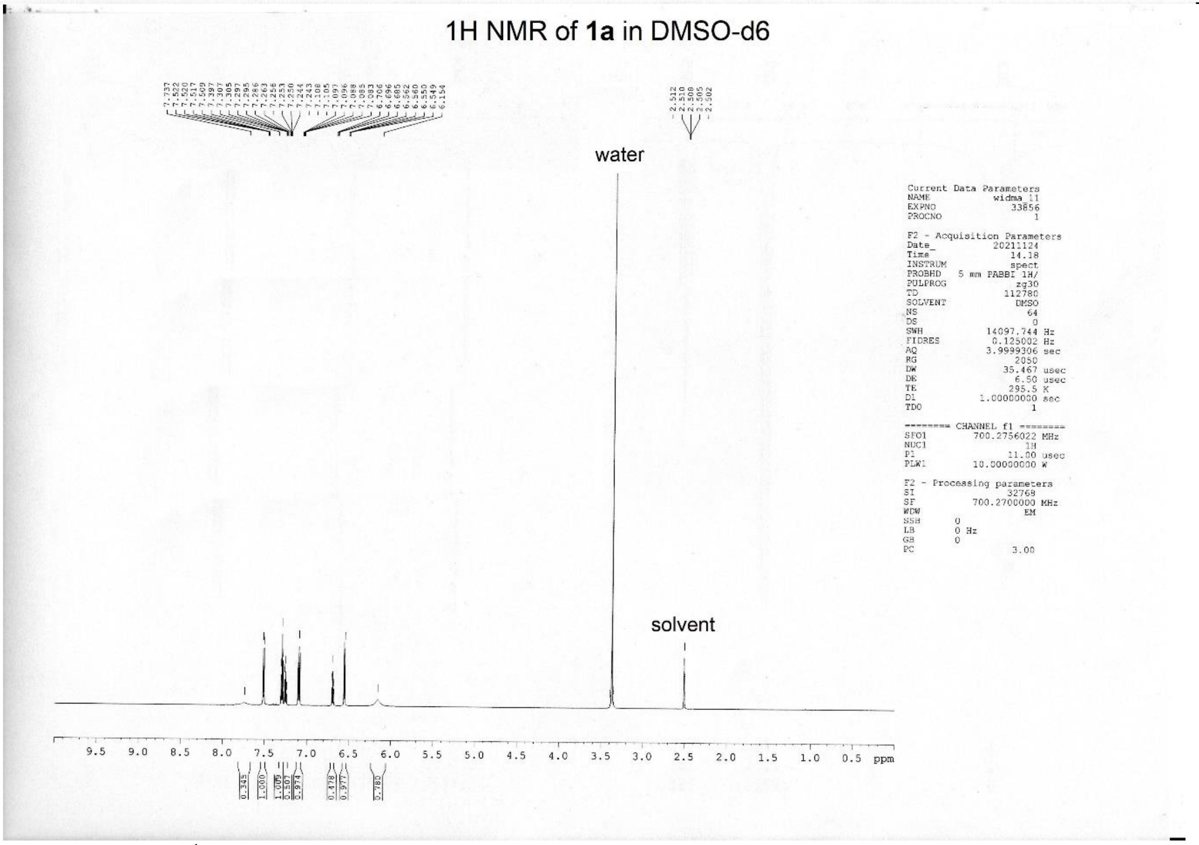

Figure S1. ^{1}H NMR spectrum of **1a** (in DMSO-d_6), with enlarged fragment at the top

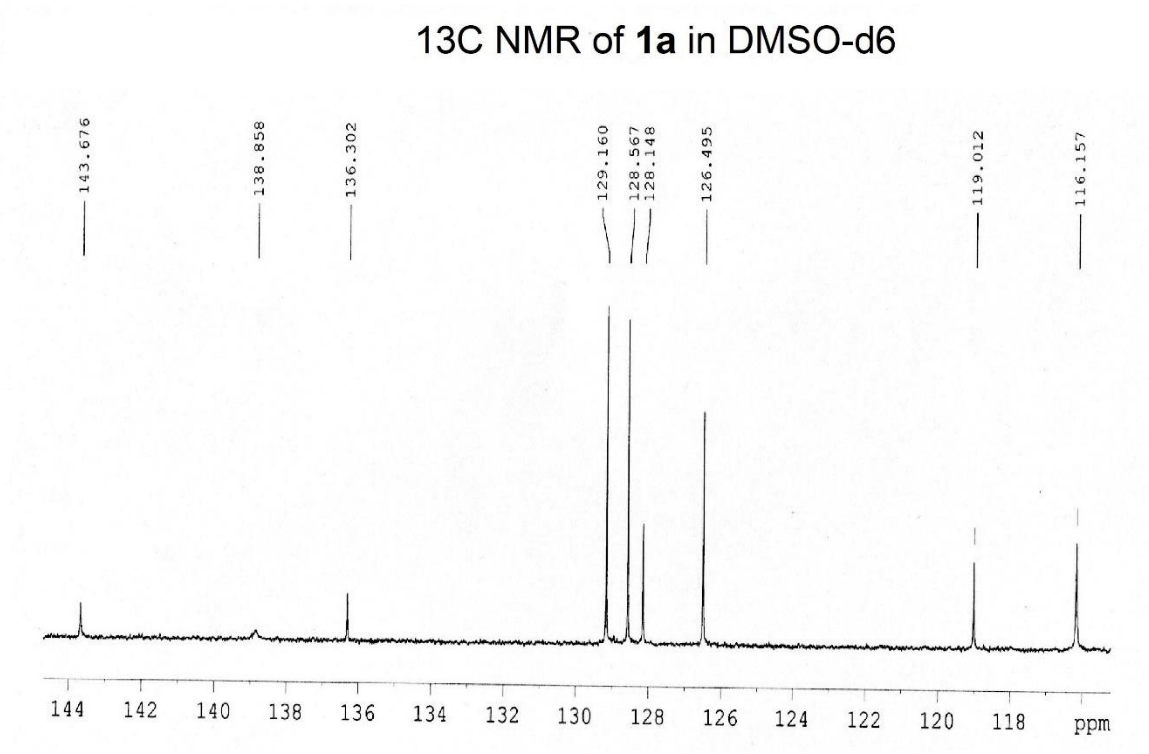

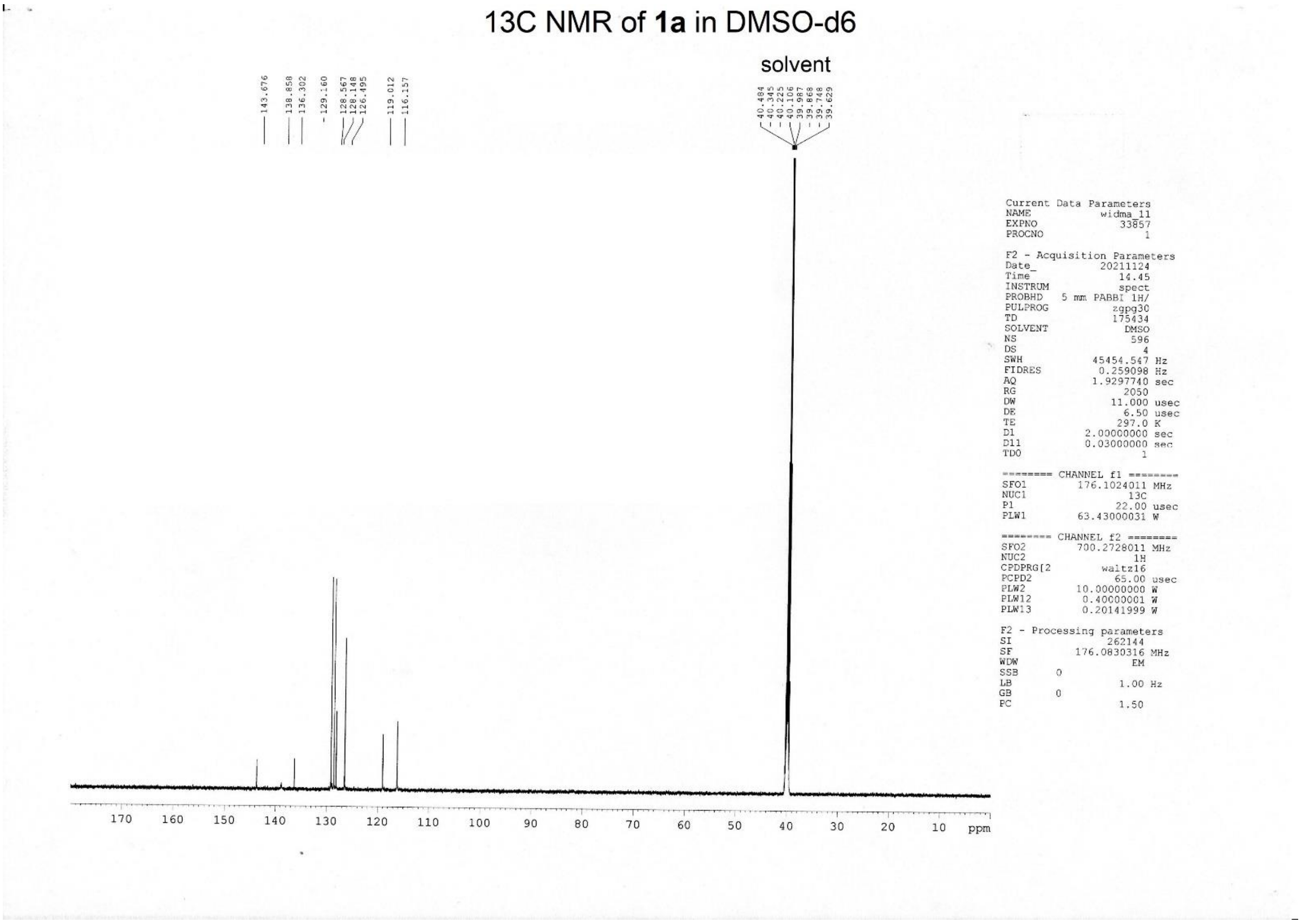

Figure S2. ^{13}C NMR spectrum of **1a** (in DMSO-d_6), with enlarged fragment at the top

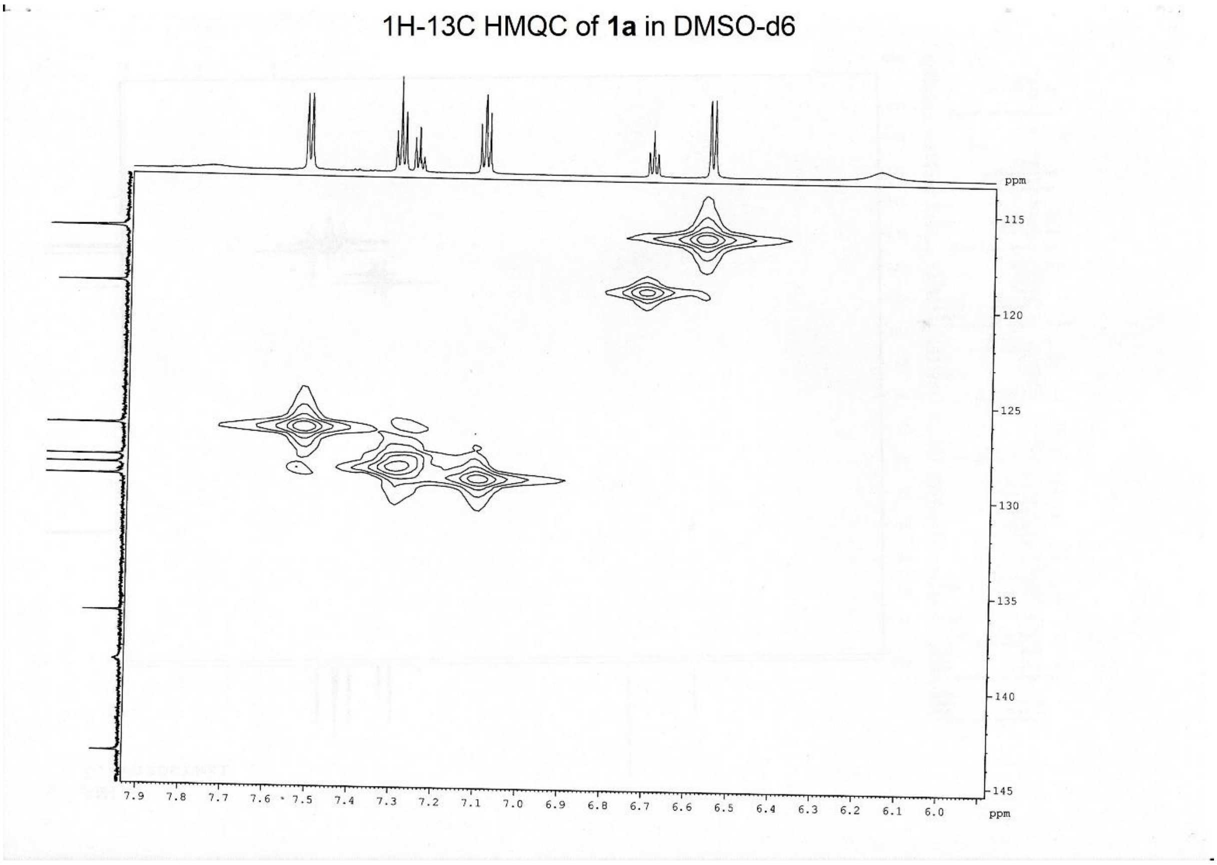

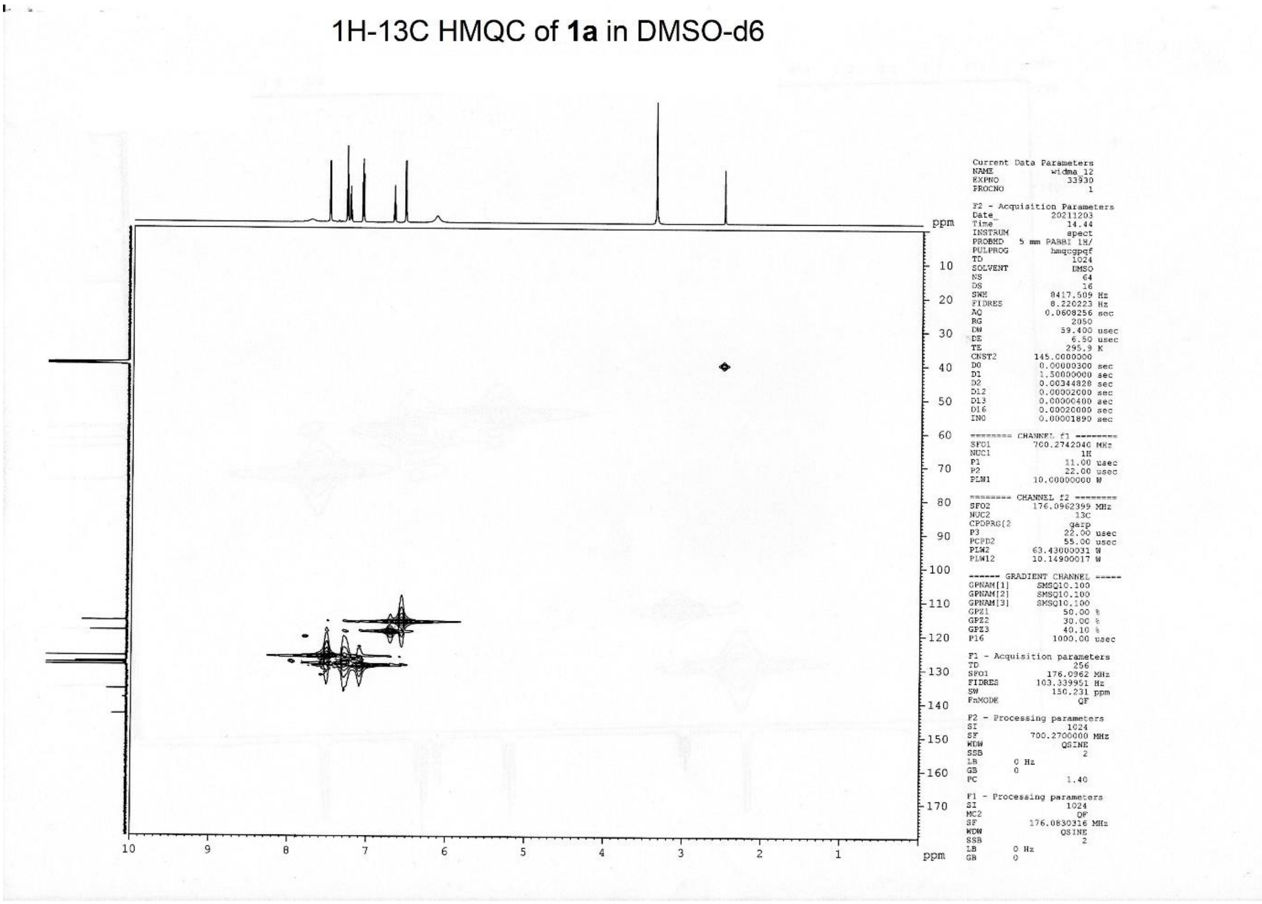

Figure S3. ^{1}H-^{13}C HMQC spectrum of **1a** (in DMSO-d$_6$), with enlarged fragment at the top

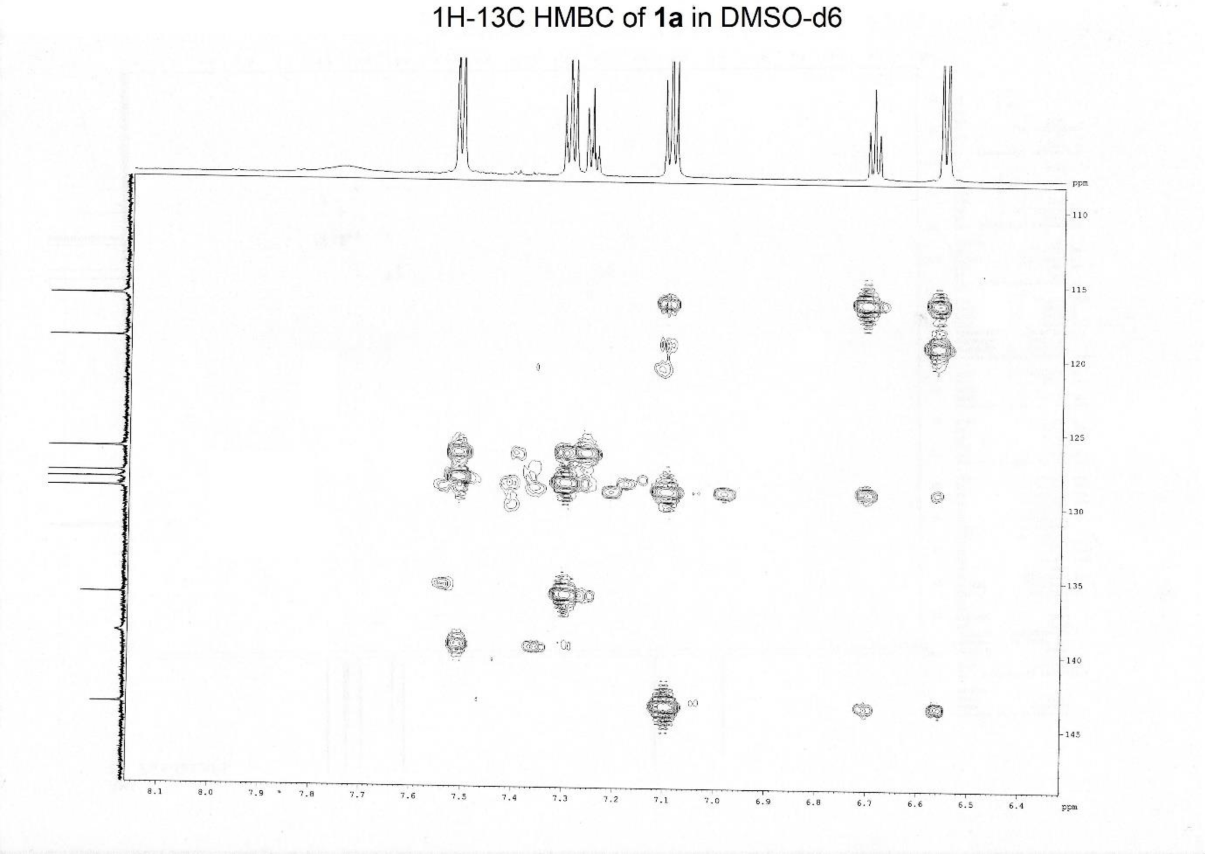

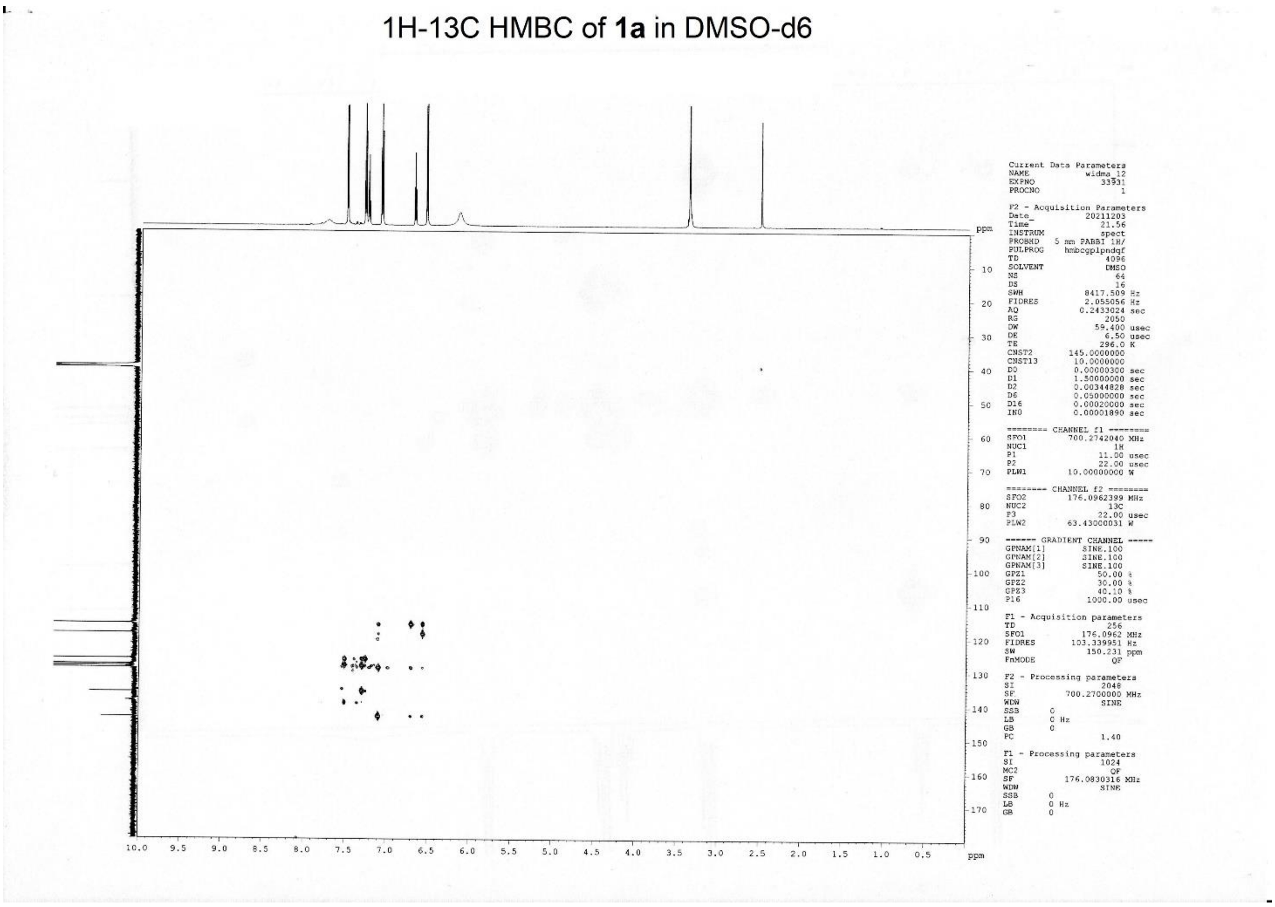

Figure S4. ^{1}H-^{13}C HMBC spectrum of **1a** (in DMSO-d_6), with enlarged fragment at the top

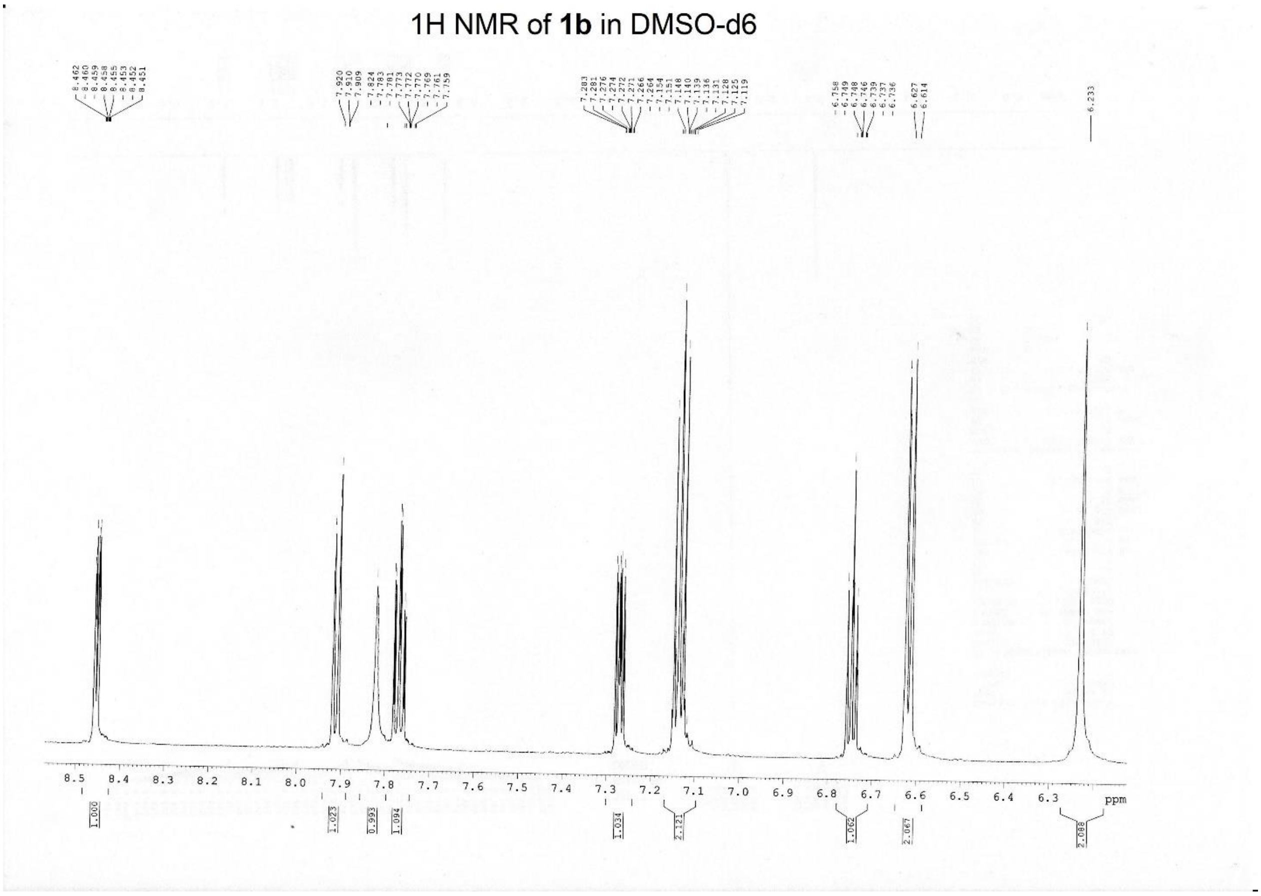

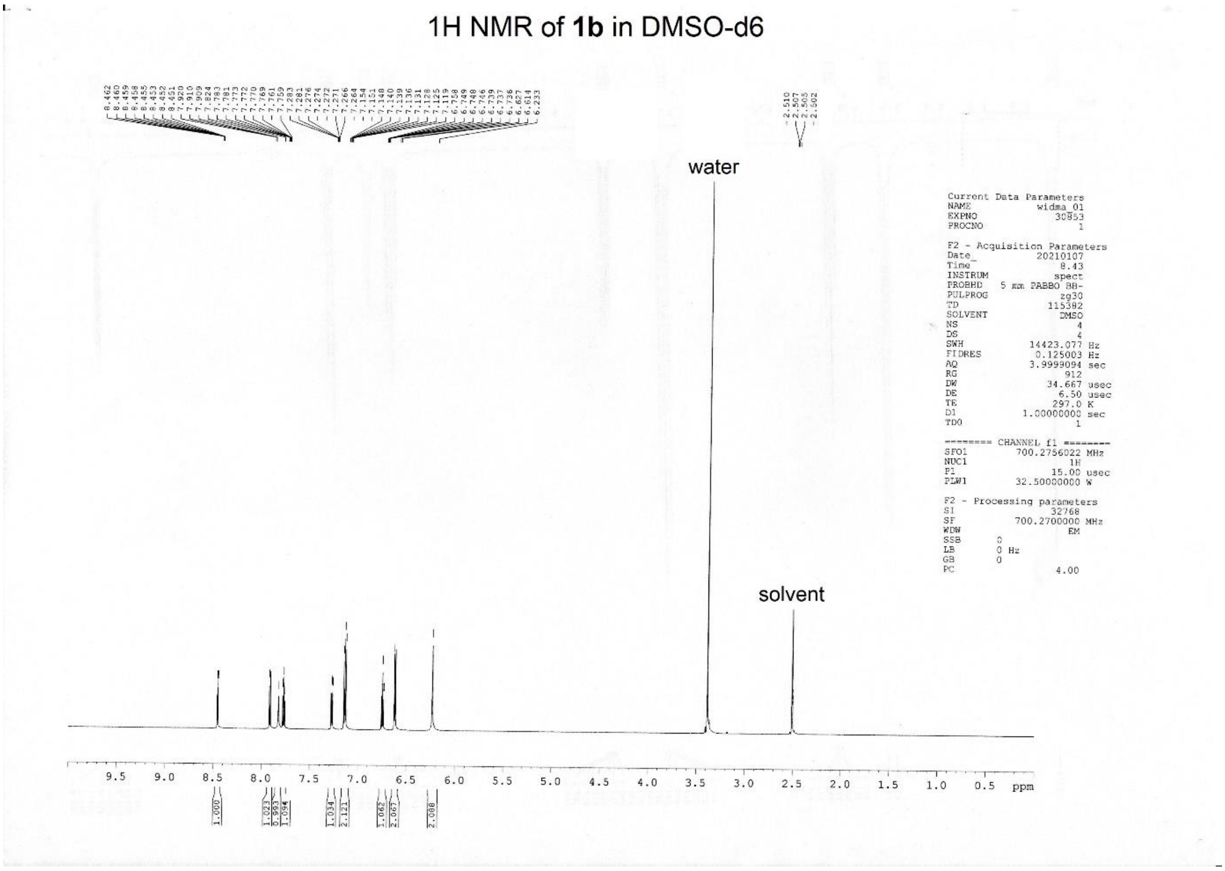

Figure S5. ^{1}H NMR spectrum of **1b** (in DMSO-d_6), with enlarged fragment at the top

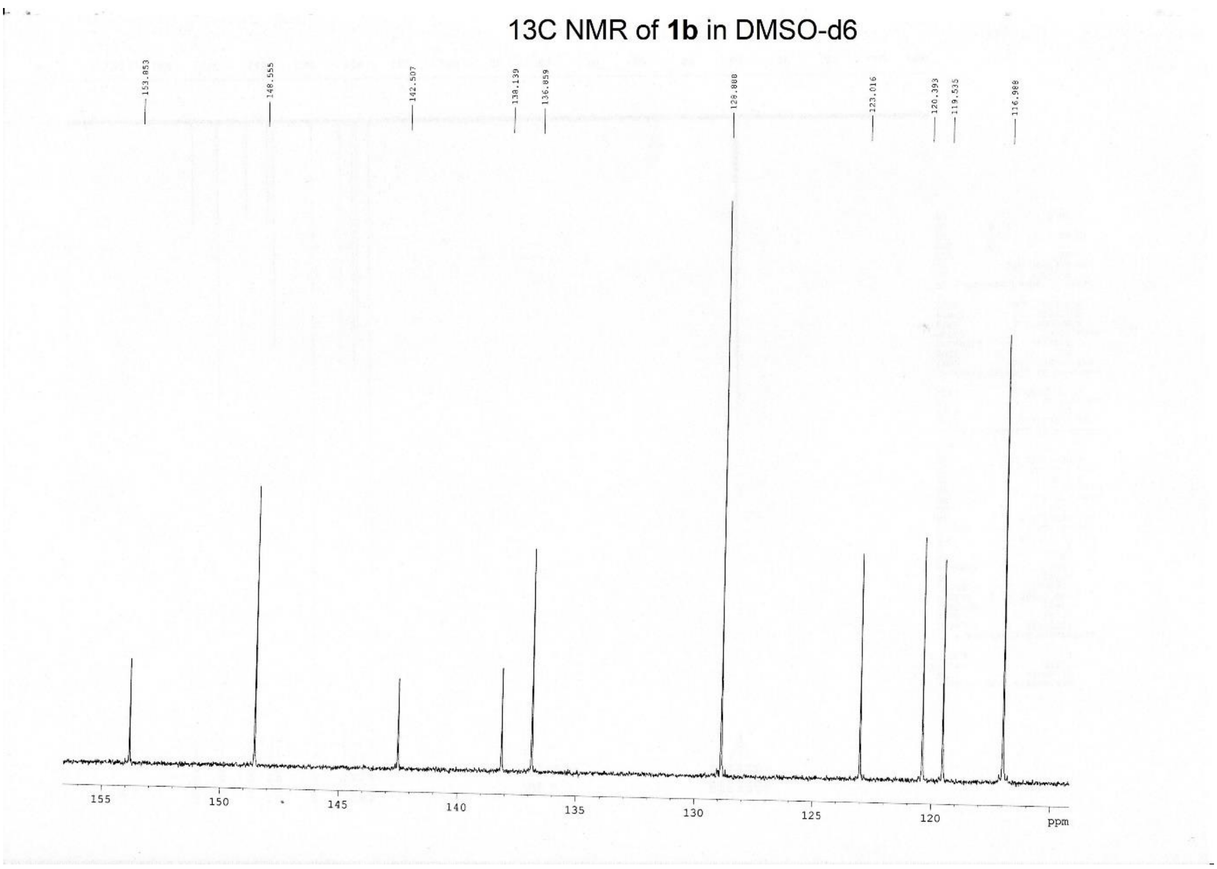

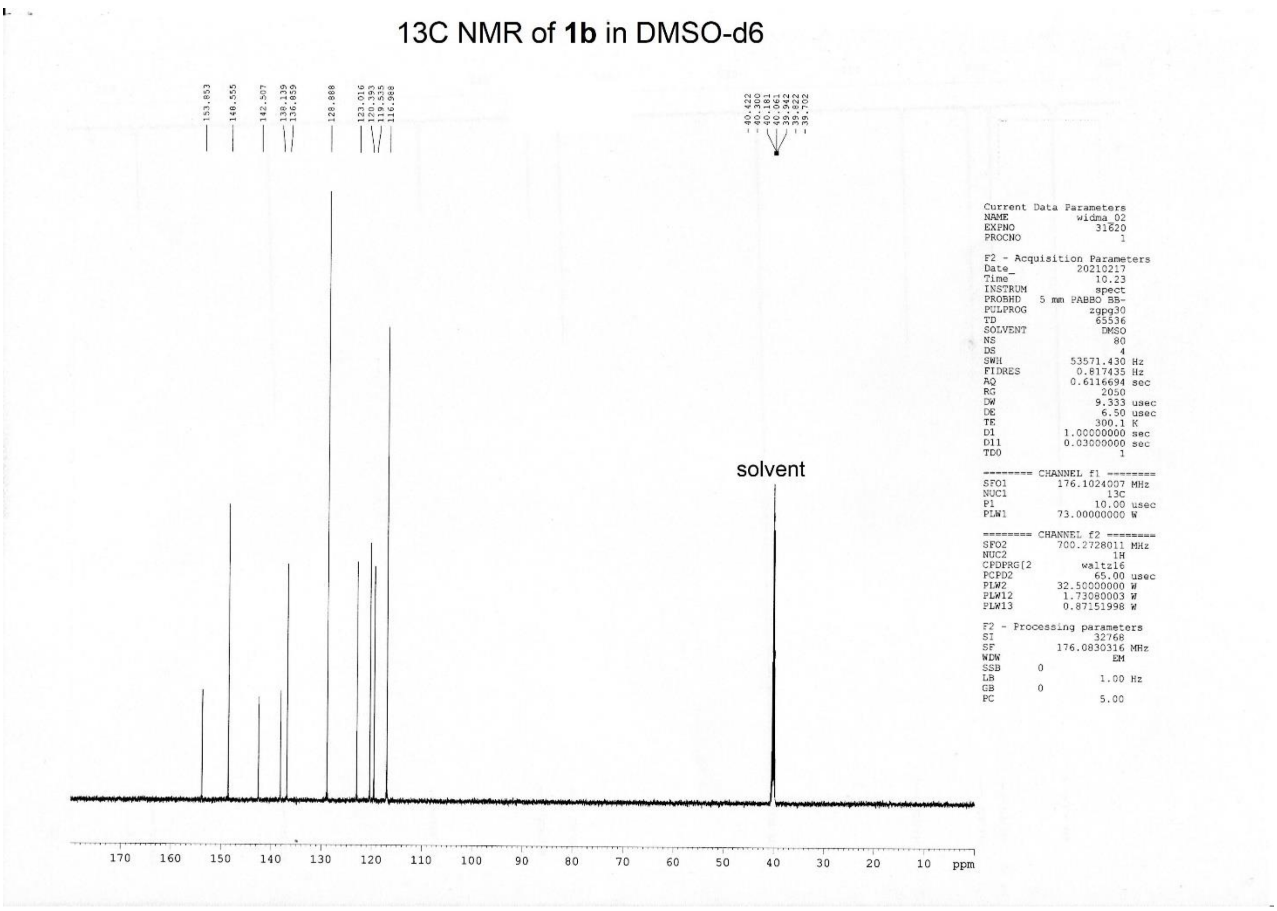

Figure S6. ^{13}C NMR spectrum of **1b** (in DMSO-d_6), with enlarged fragment at the top

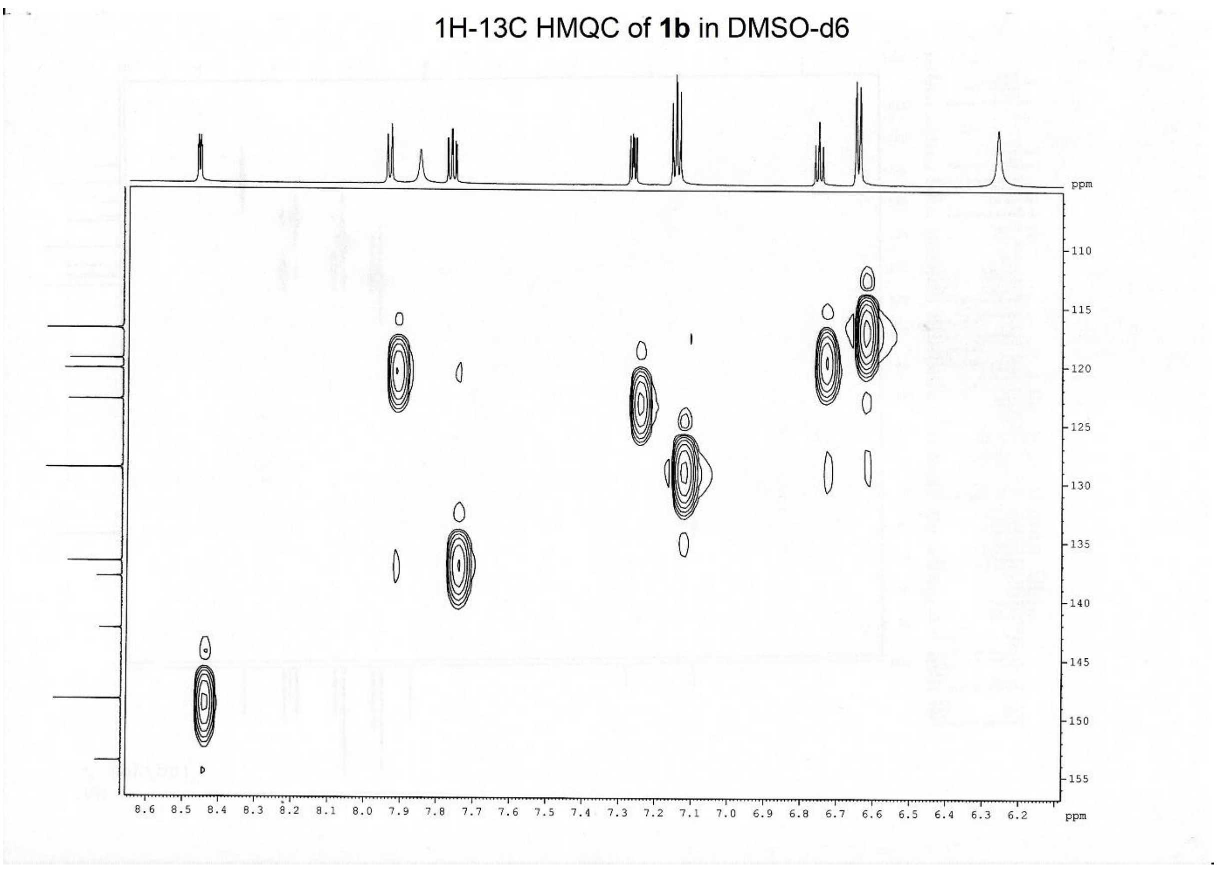

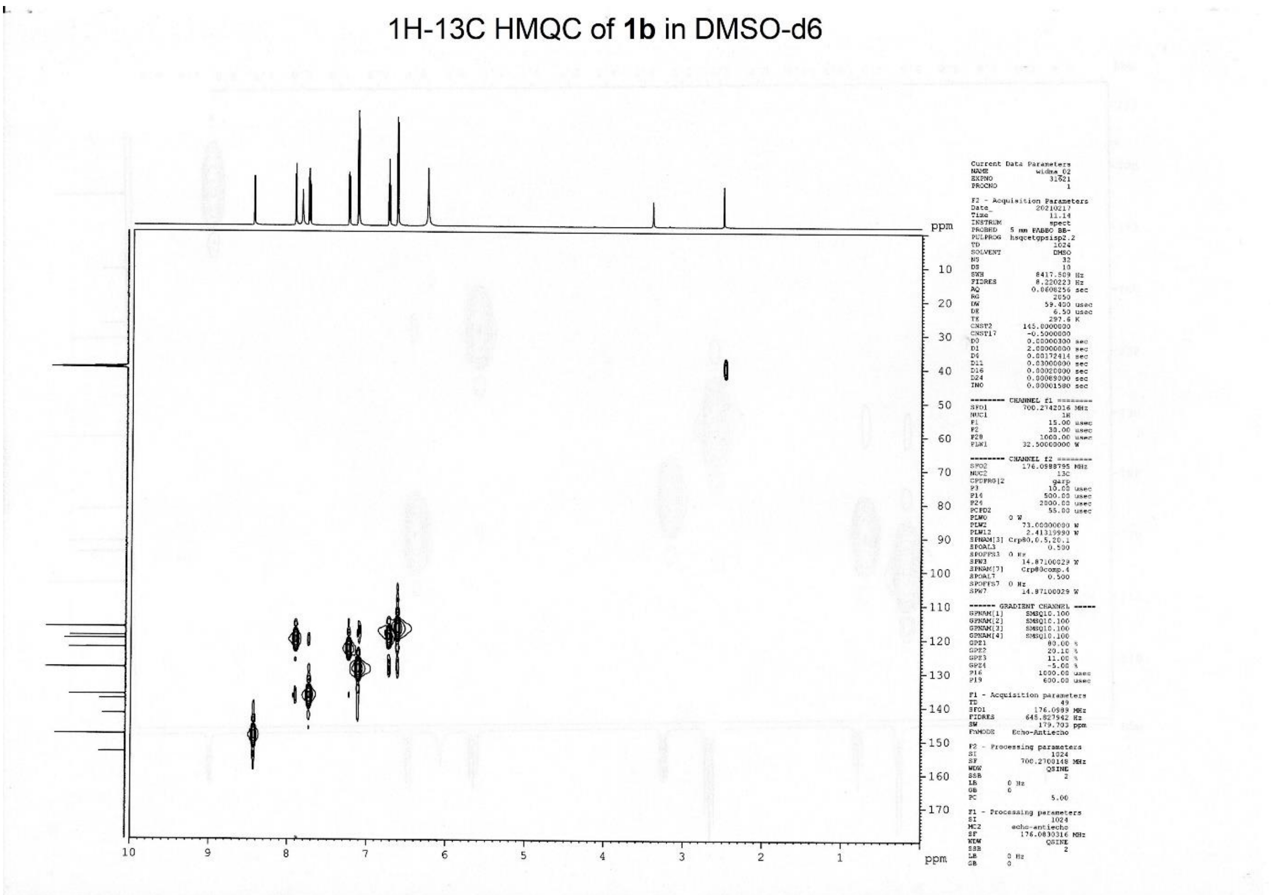

Figure S7. ^{1}H-^{13}C HMQC spectrum of **1b** (in DMSO-d_6), with enlarged fragment at the top

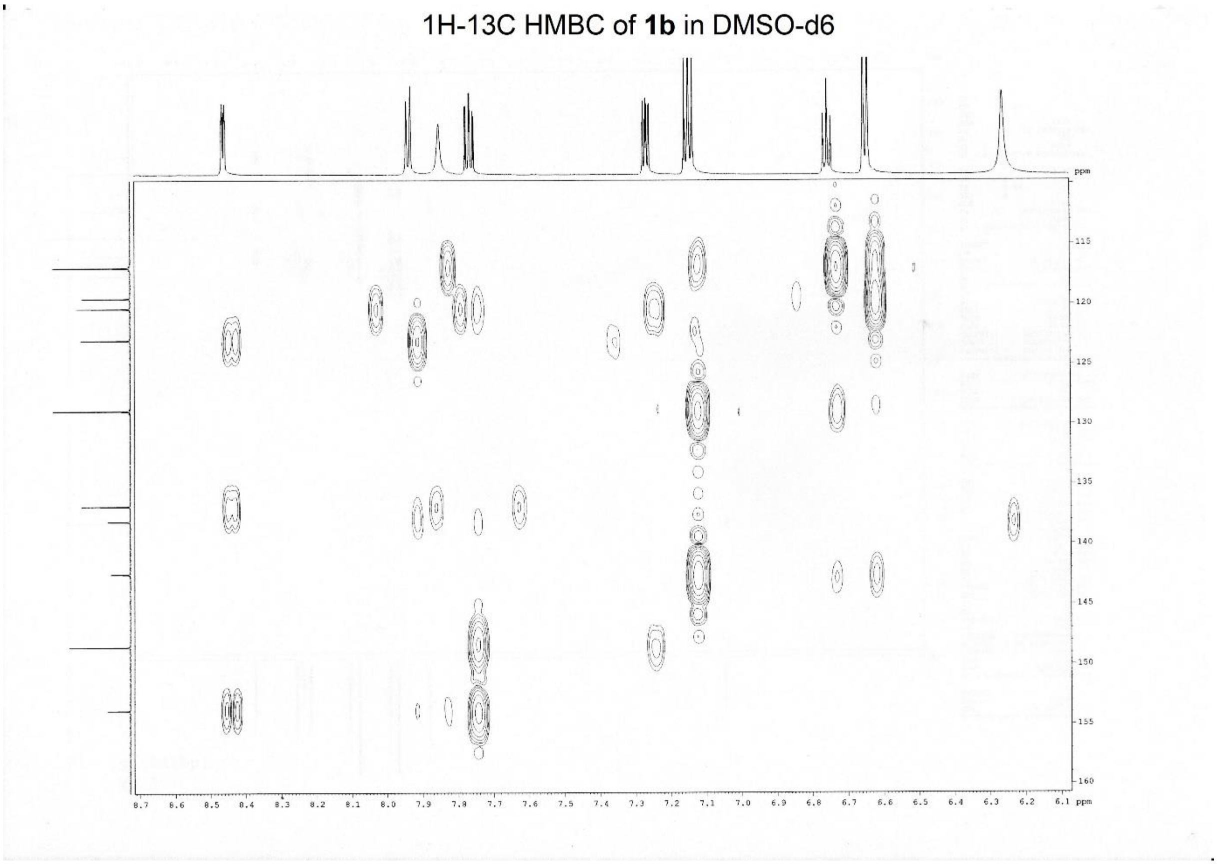

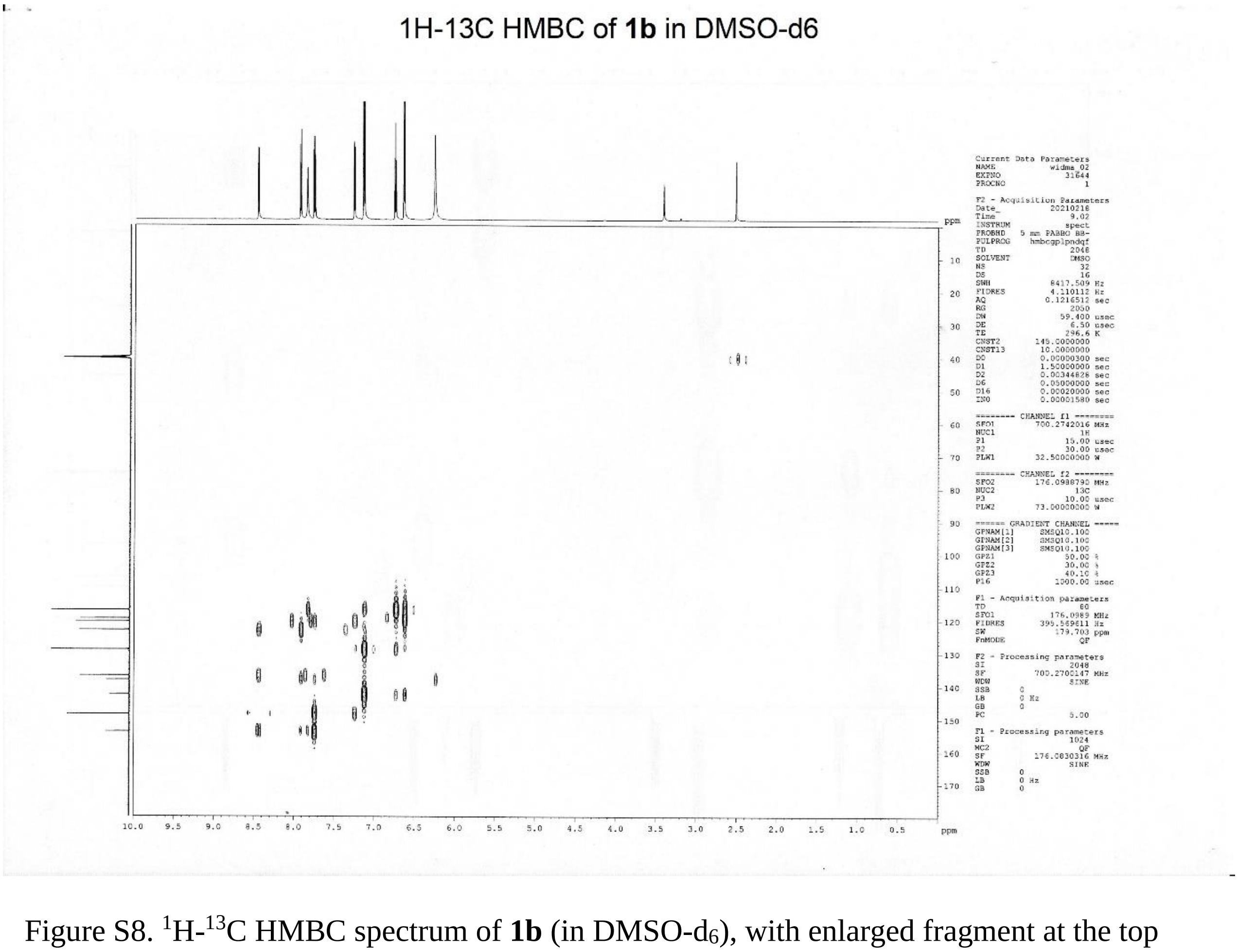

Figure S8. ^{1}H-^{13}C HMBC spectrum of **1b** (in DMSO-d$_6$), with enlarged fragment at the top

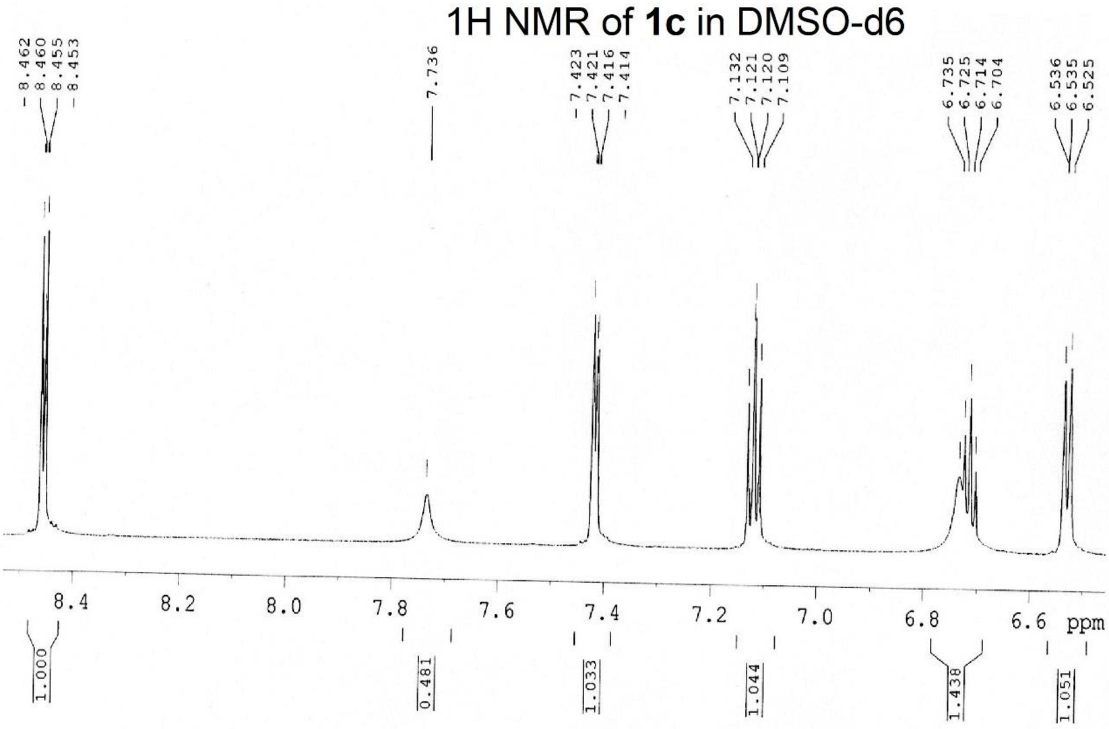

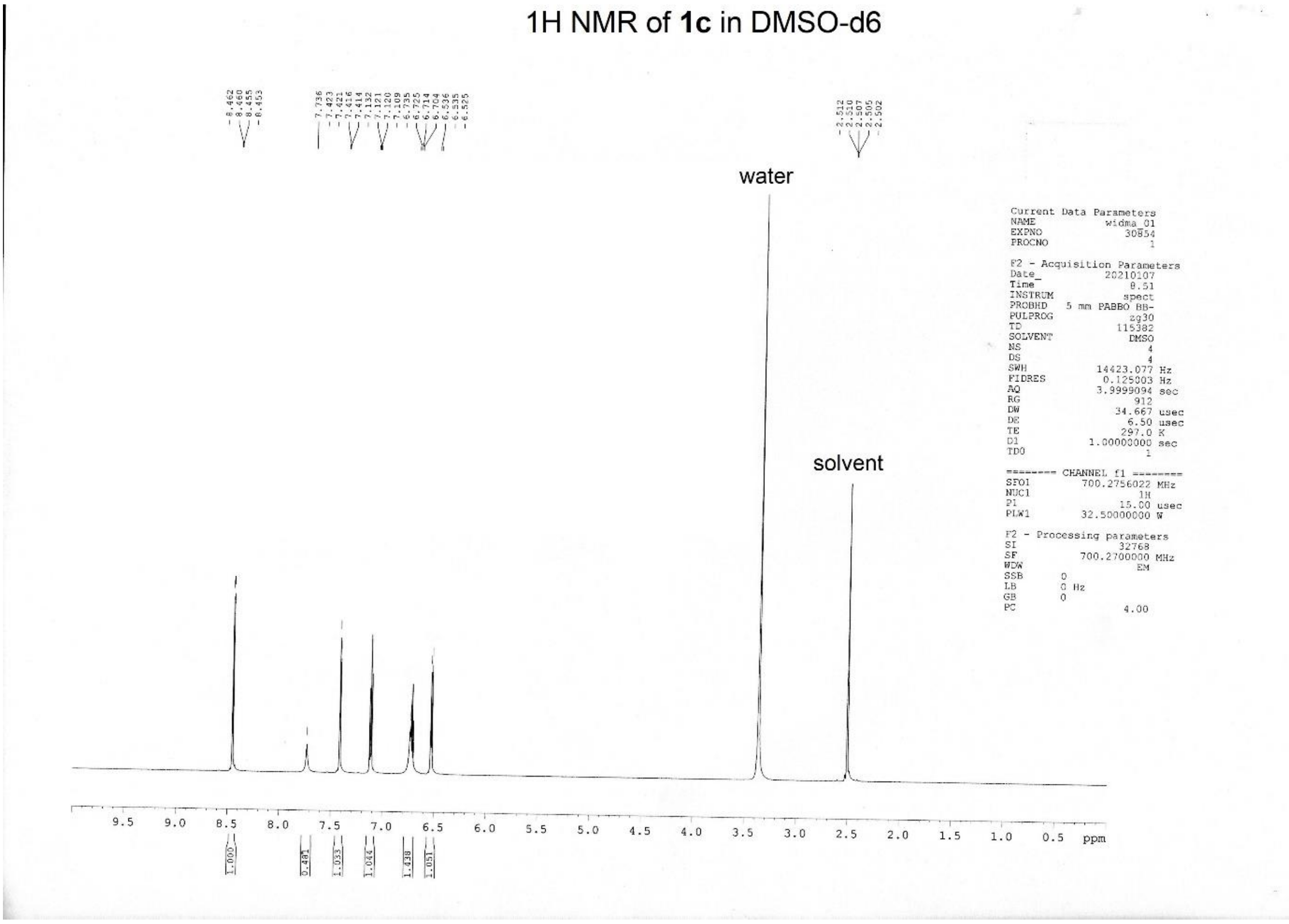

Figure S9. ^{1}H NMR spectrum of **1c** (in DMSO-d_6), with enlarged fragment at the top

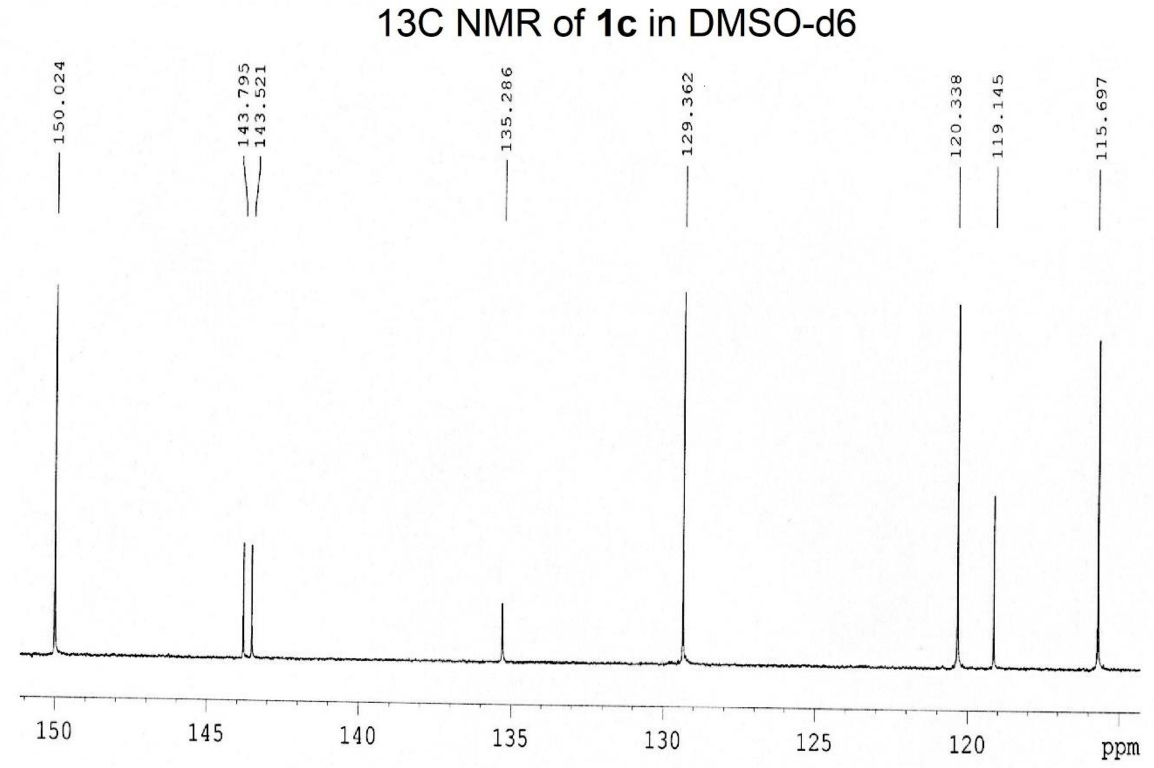

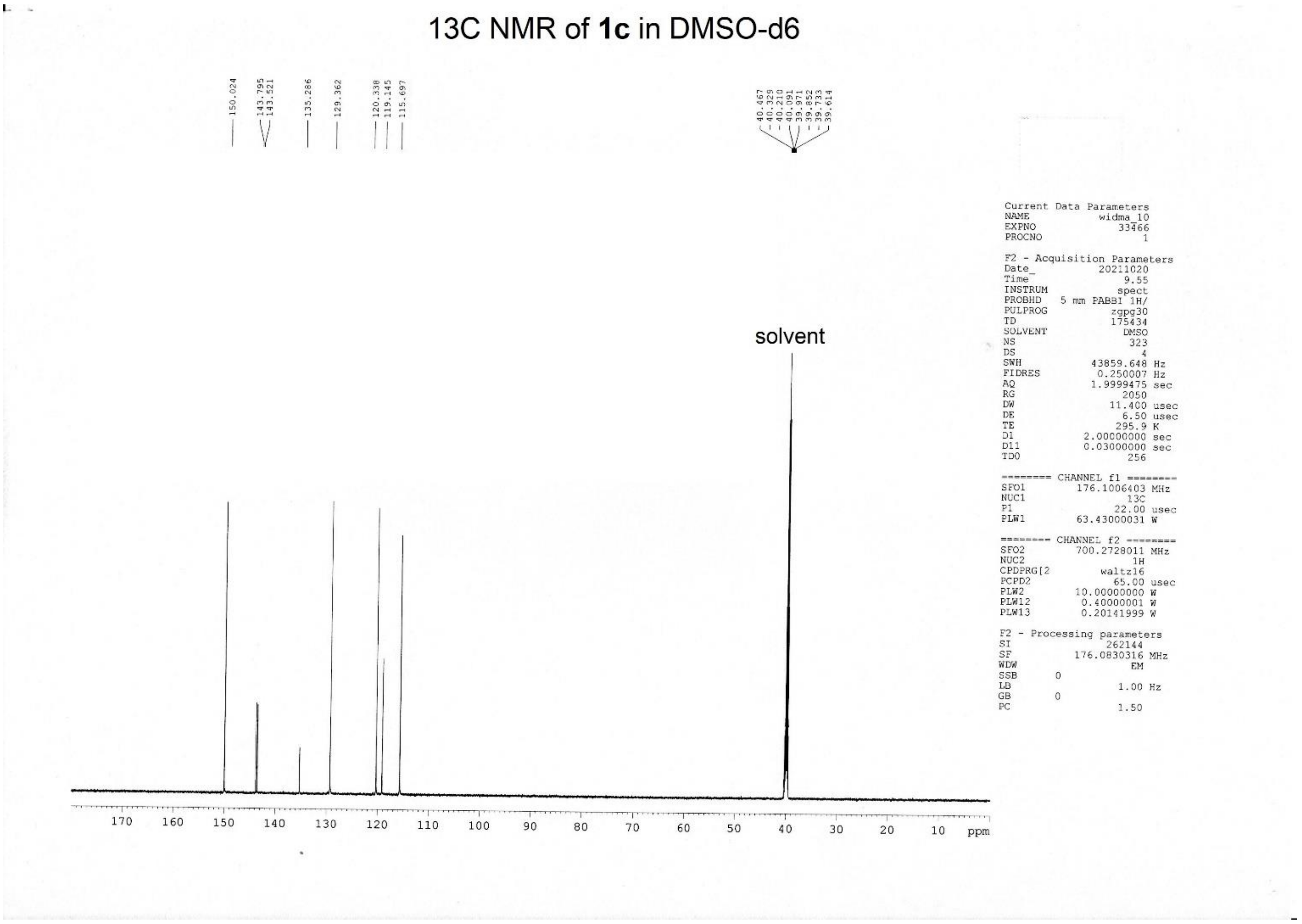

Figure S10. ^{13}C NMR spectrum of **1c** (in DMSO-d_6), with enlarged fragment at the top

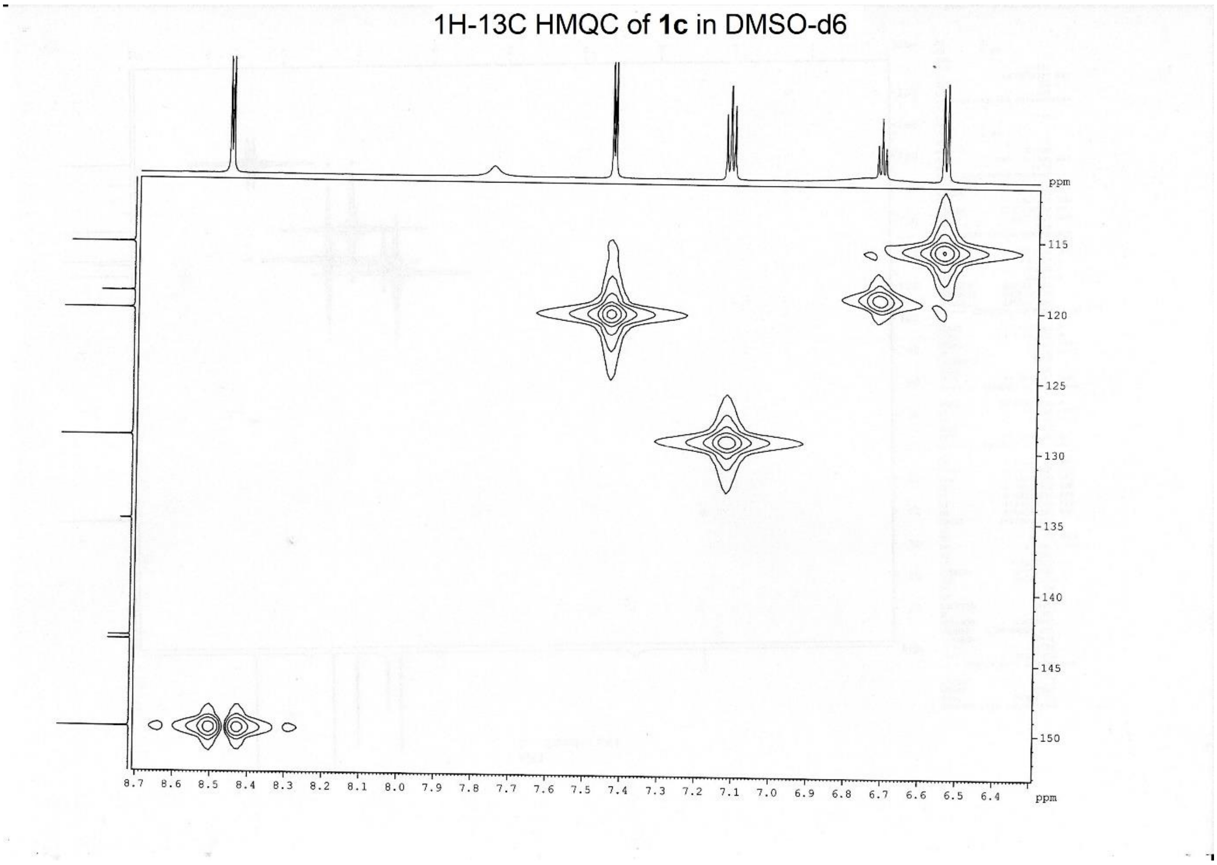

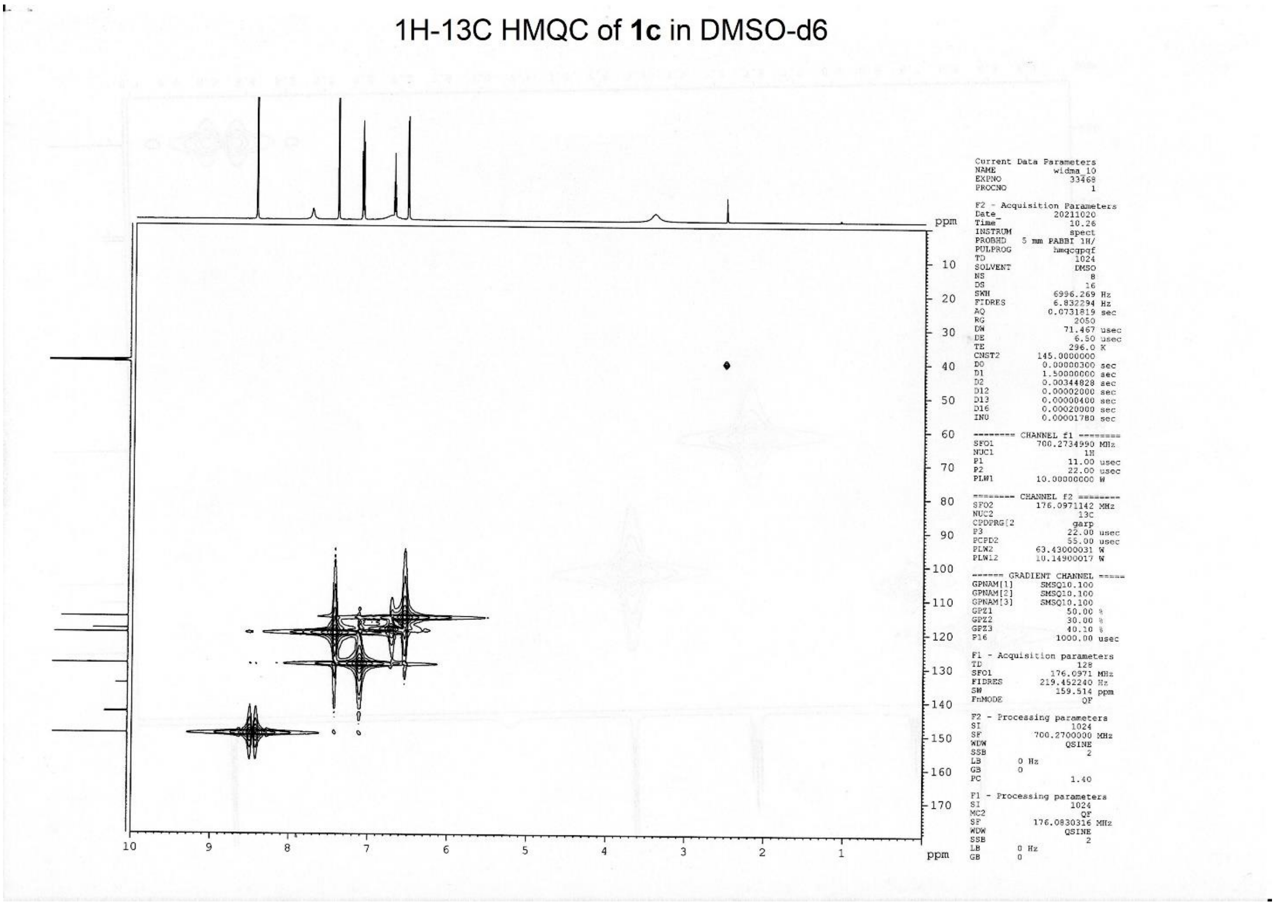

Figure S11. ^{1}H-^{13}C HMQC spectrum of **1c** (in DMSO-d_6), with enlarged fragment at the top

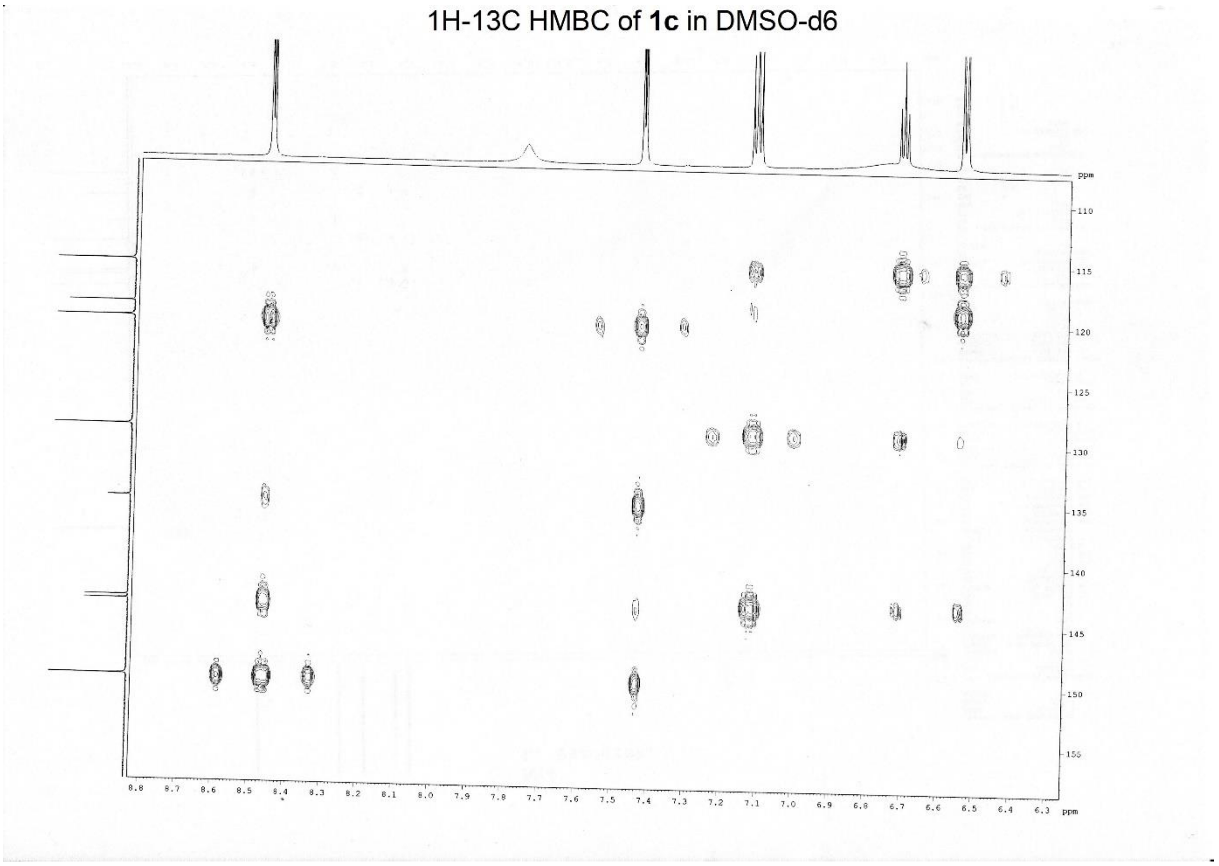

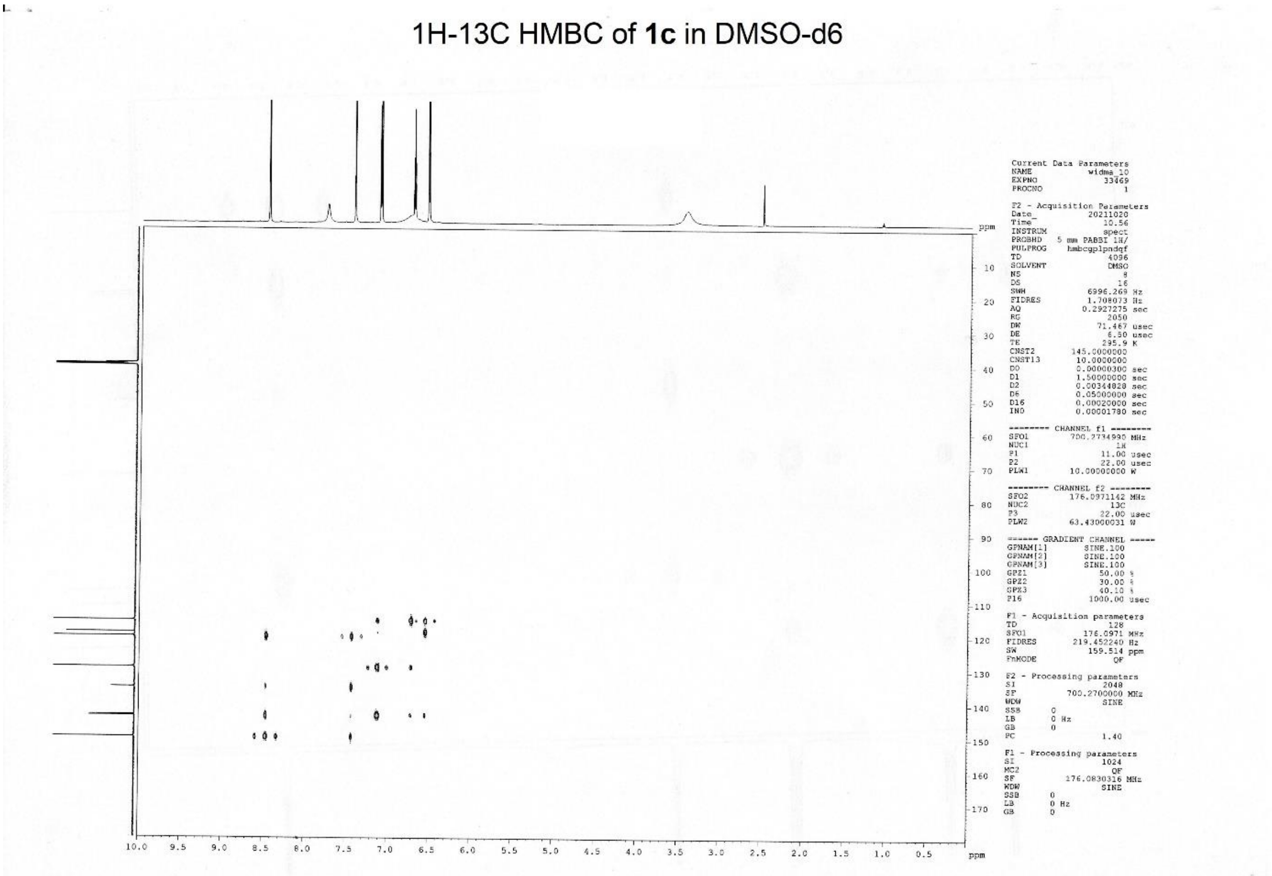

Figure S12. ^{1}H-^{13}C HMBC spectrum of **1c** (in DMSO-d$_6$), with enlarged fragment at the top

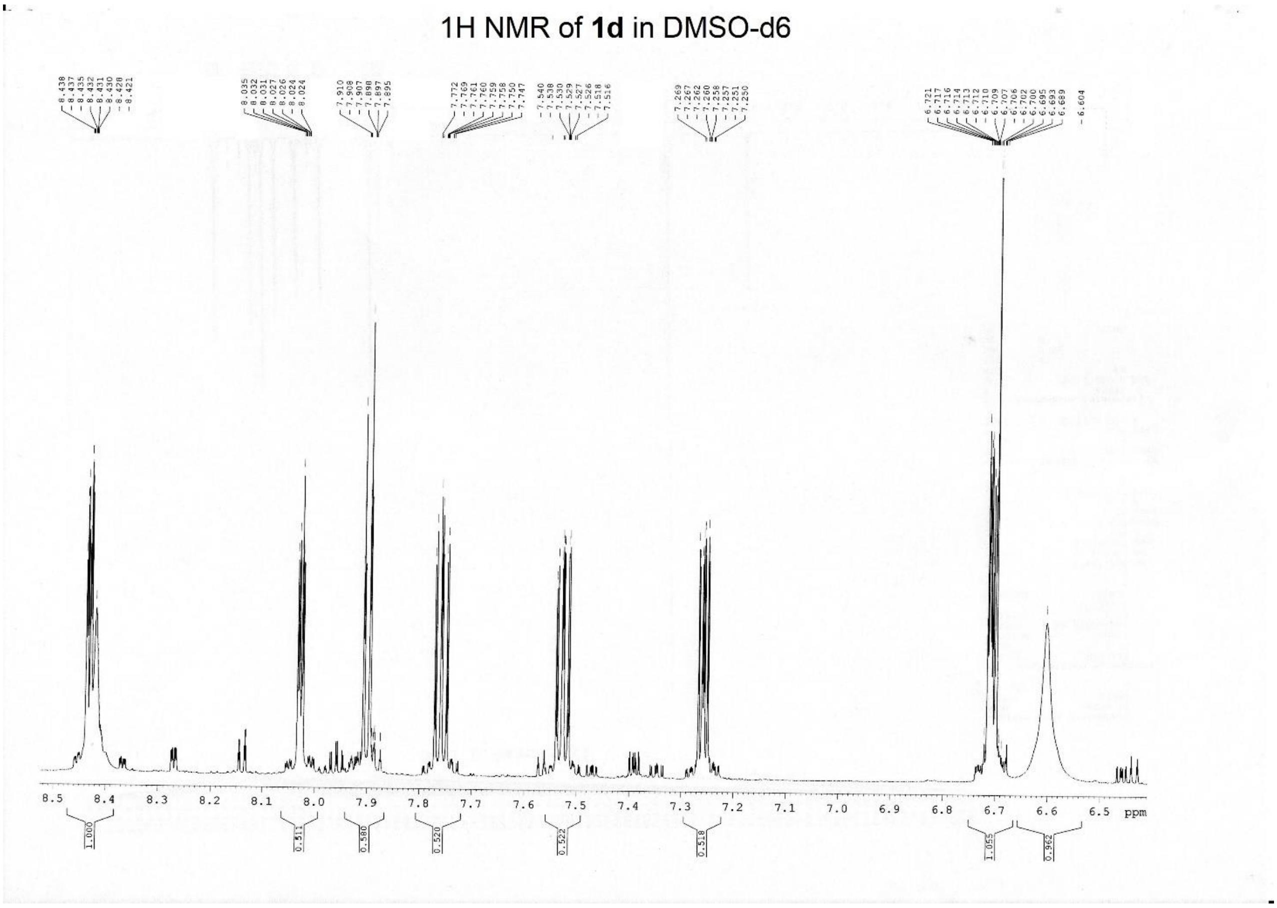

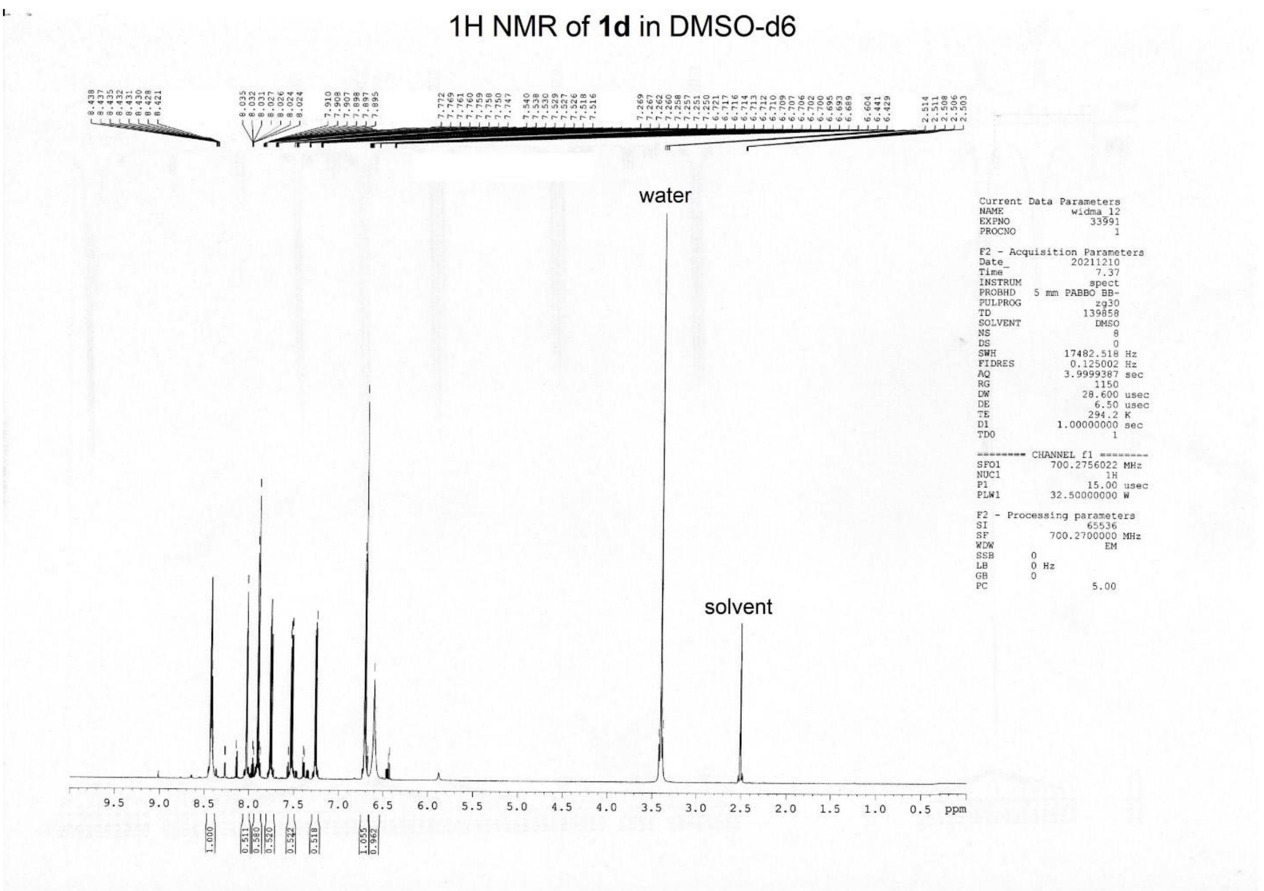

Figure S13. ^{1}H NMR spectrum of **1d** (in DMSO-d_6), with enlarged fragment at the top

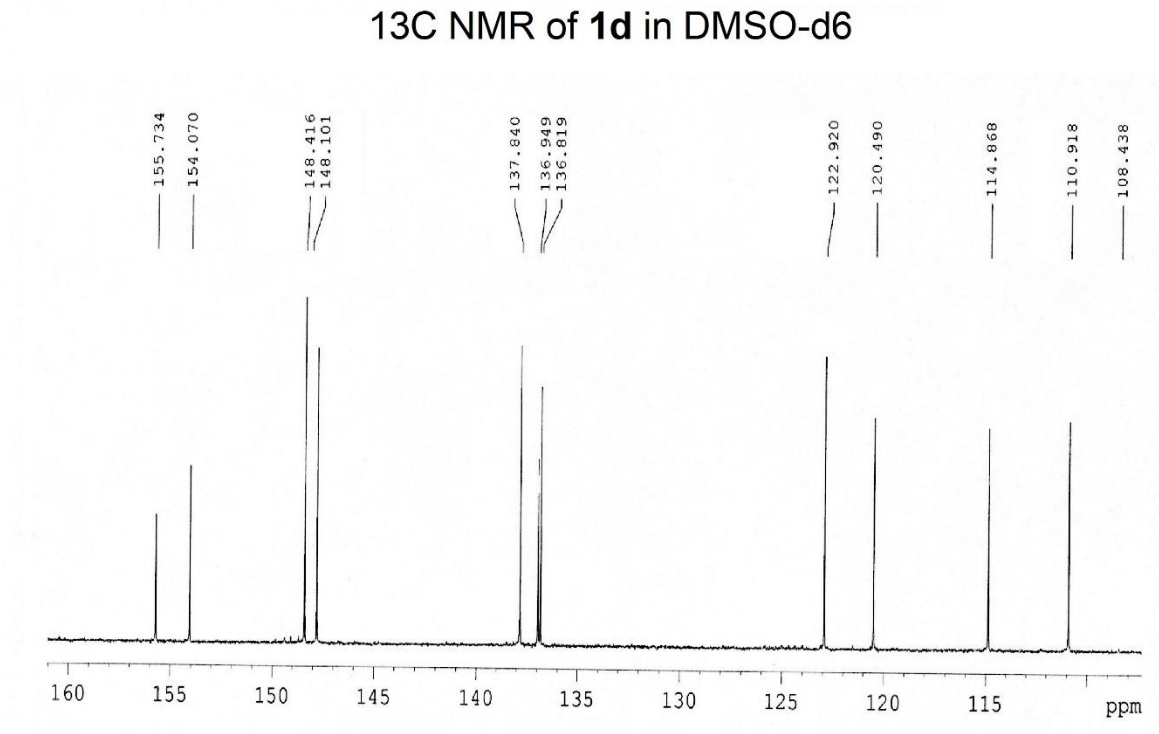

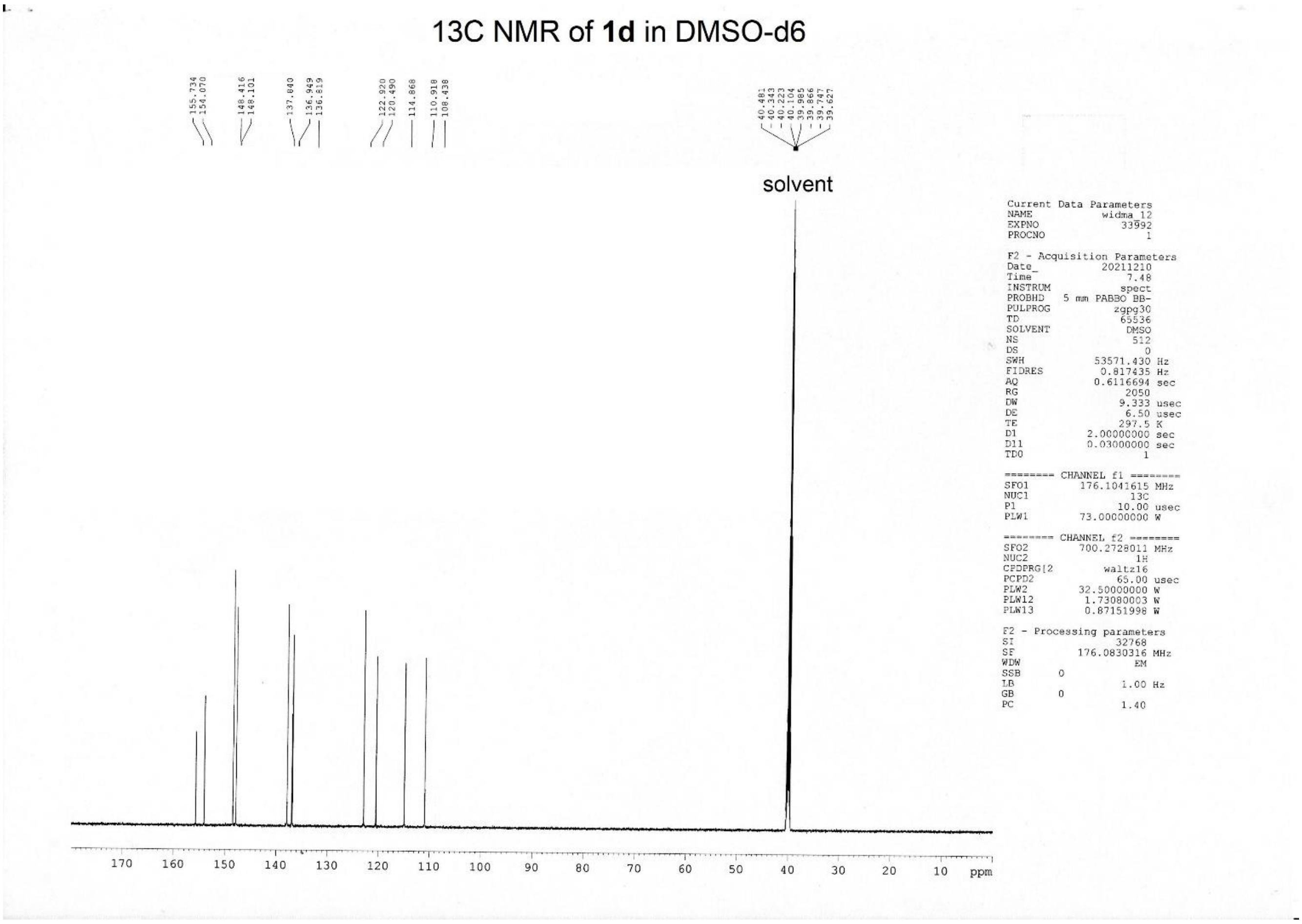

Figure S14. ^{13}C NMR spectrum of **1d** (in DMSO-d$_6$), with enlarged fragment at the top

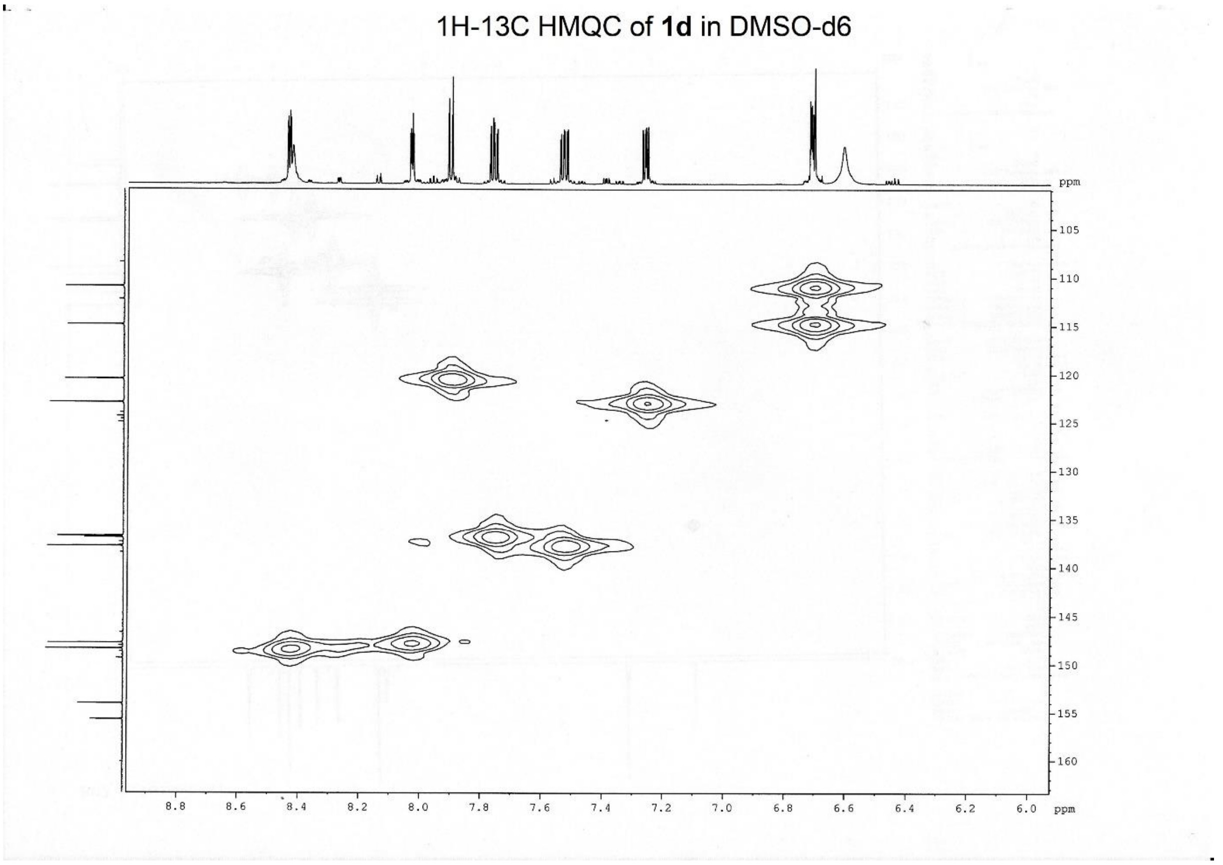

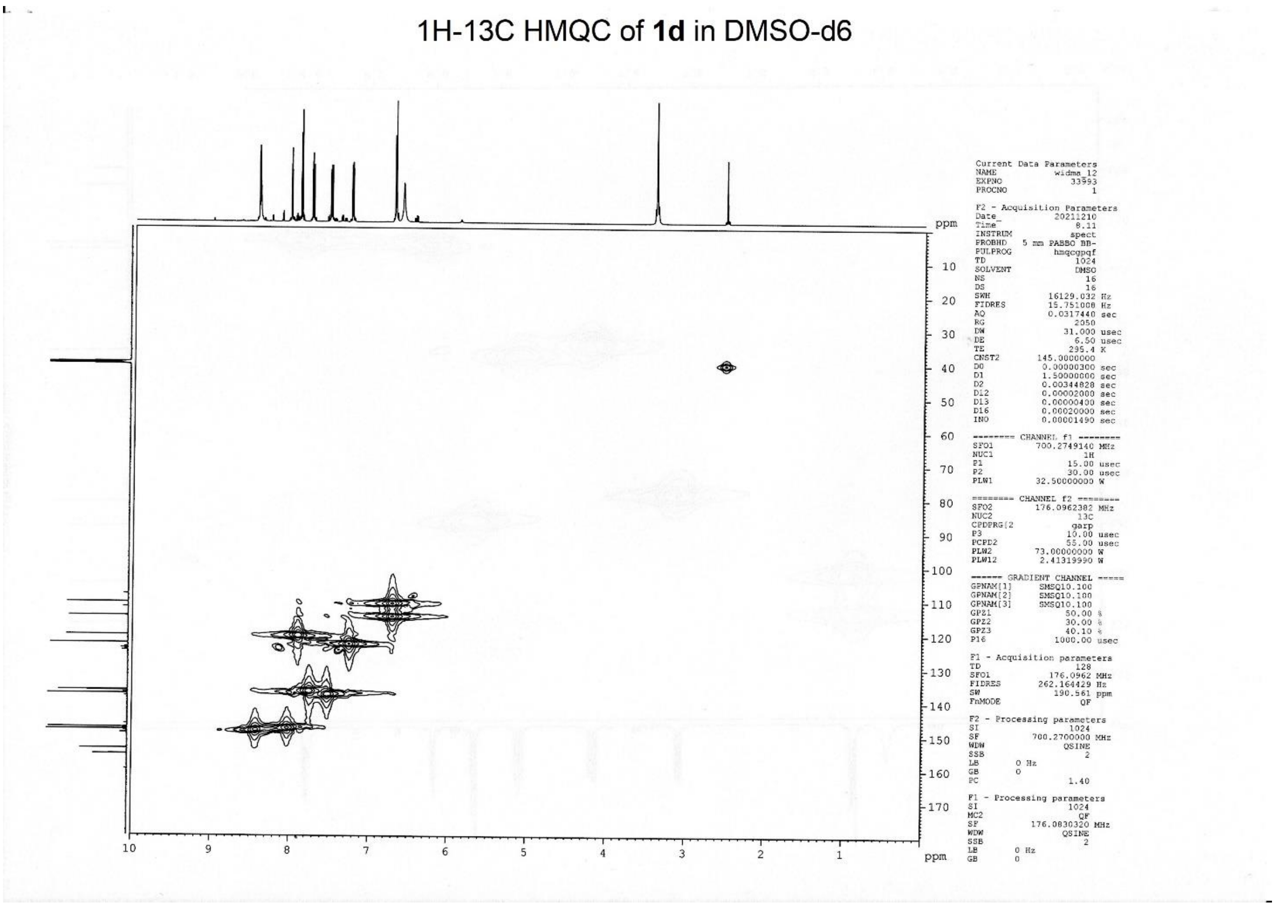

Figure S15. ^{1}H-^{13}C HMQC spectrum of **1d** (in DMSO-d$_6$), with enlarged fragment at the top

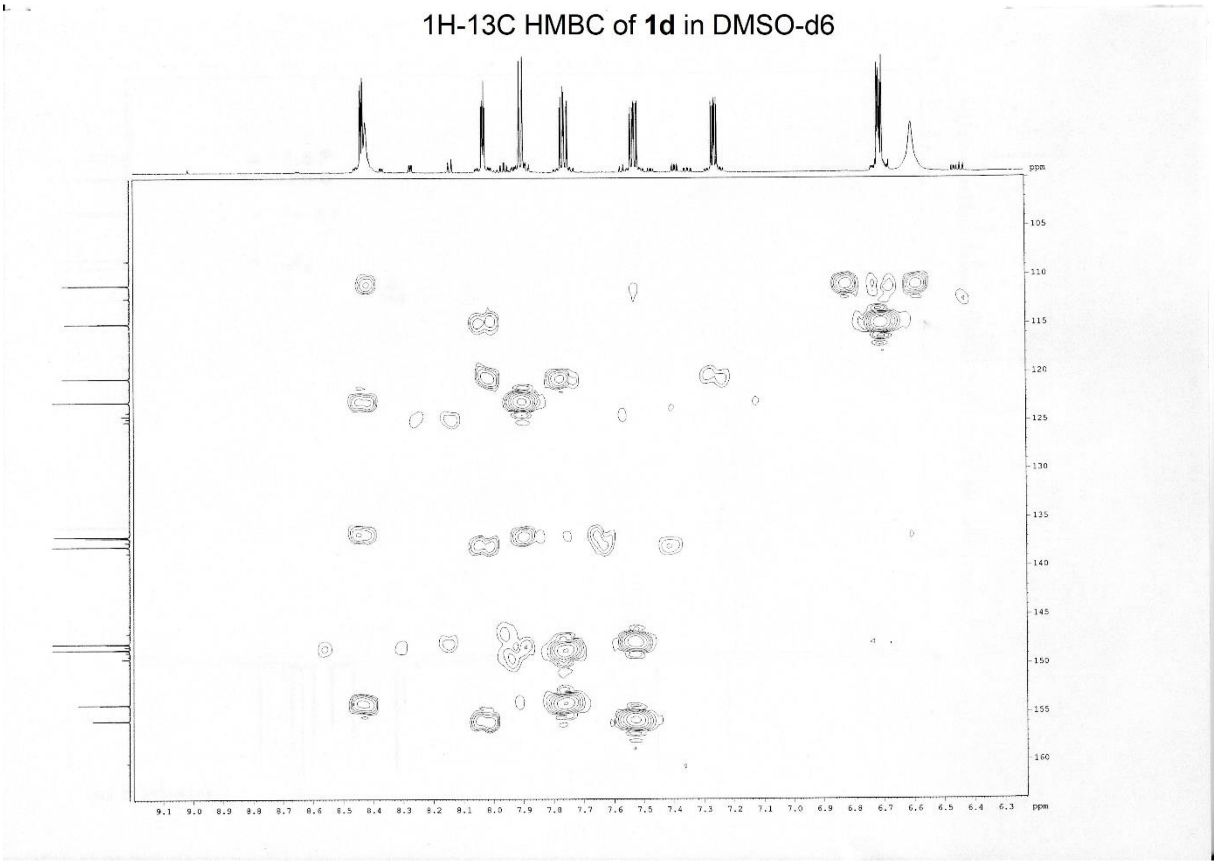

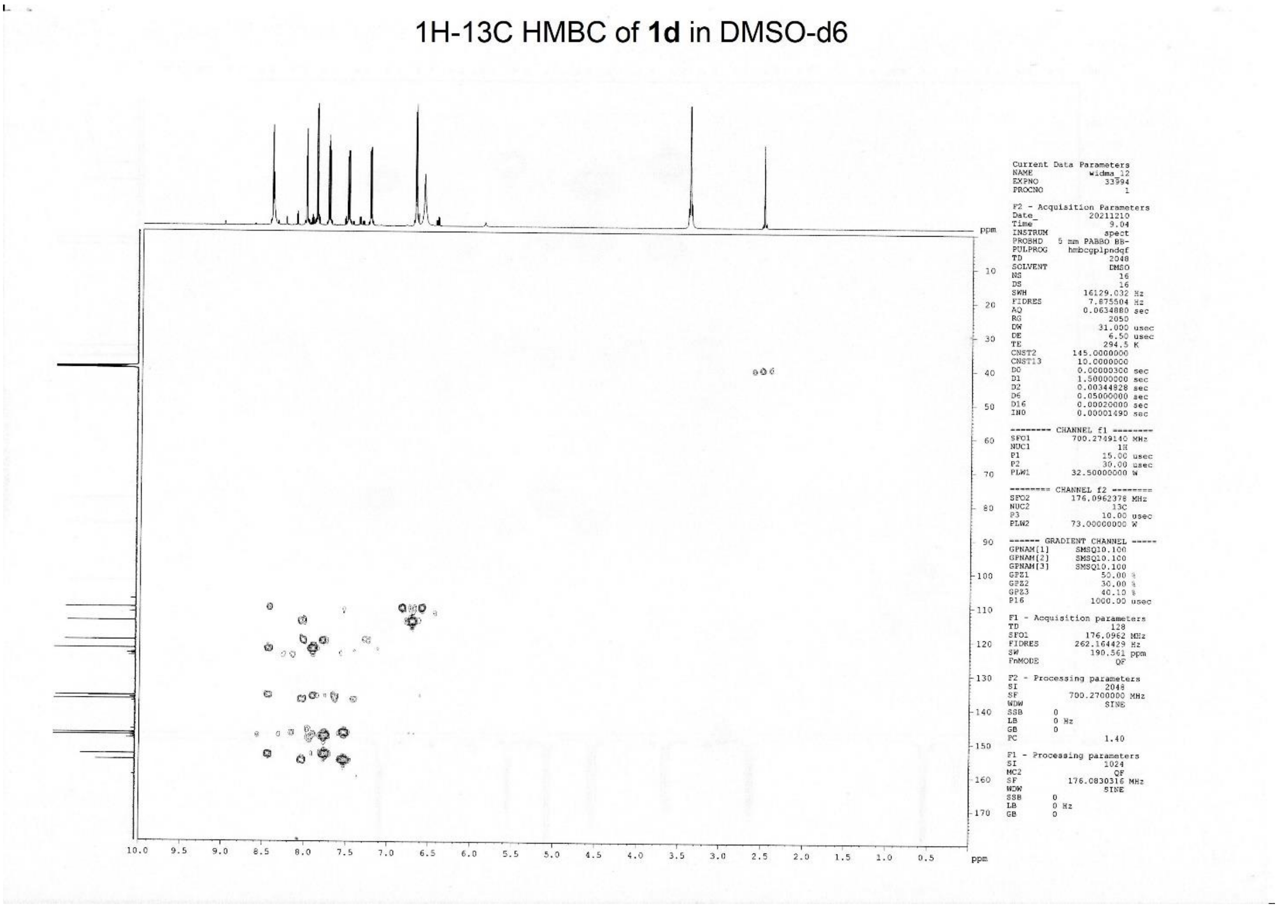

Figure S16. ^{1}H-^{13}C HMBC spectrum of **1d** (in DMSO-d_6), with enlarged fragment at the top

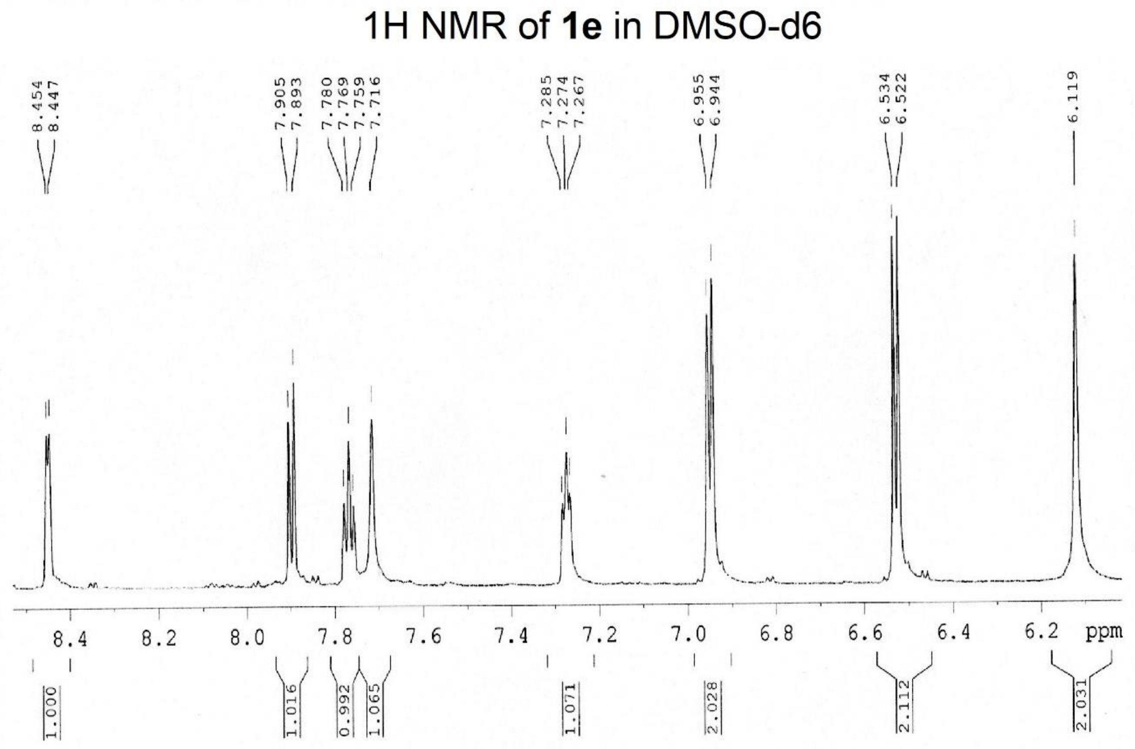

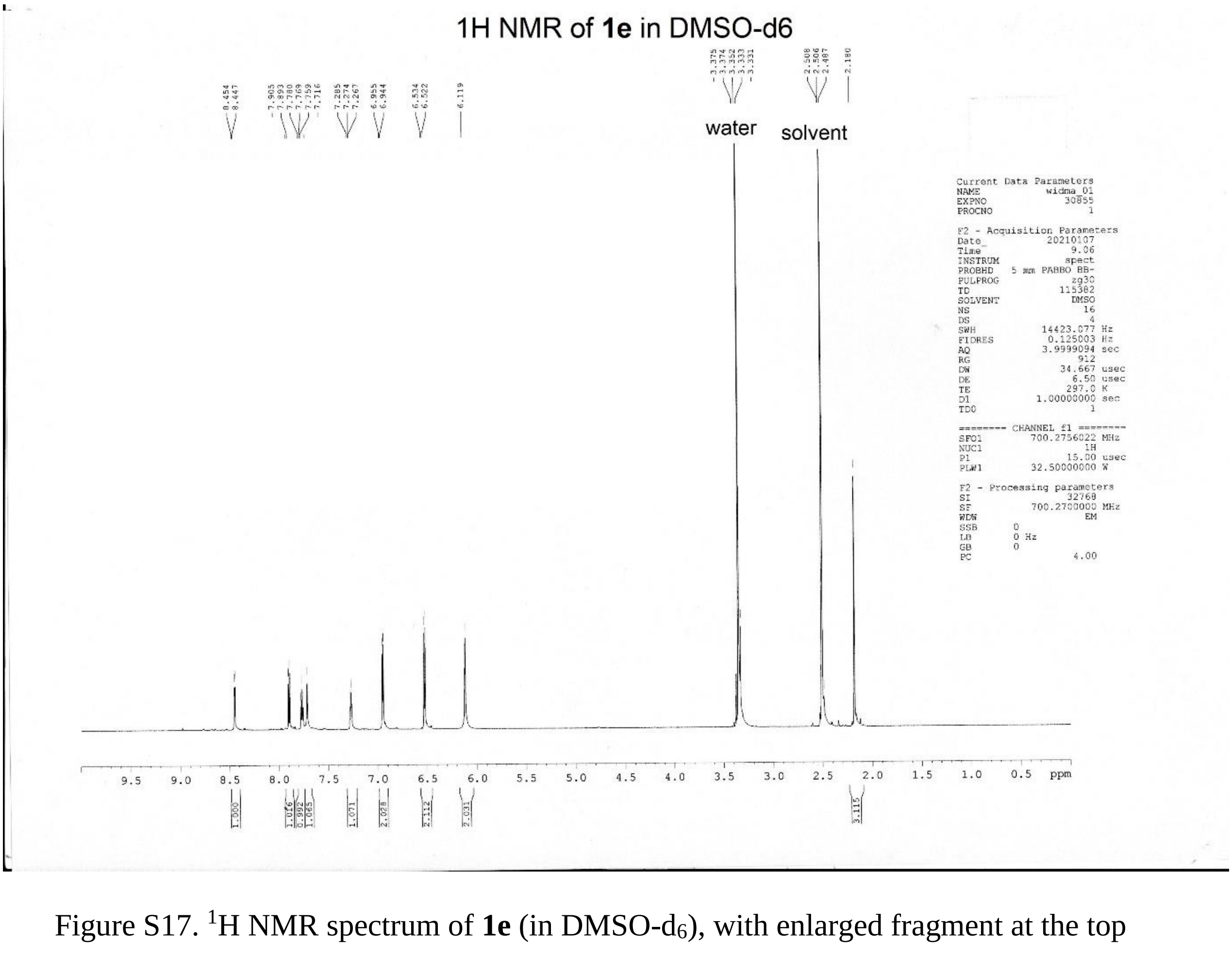

Figure S17. ^{1}H NMR spectrum of **1e** (in DMSO-d_6), with enlarged fragment at the top

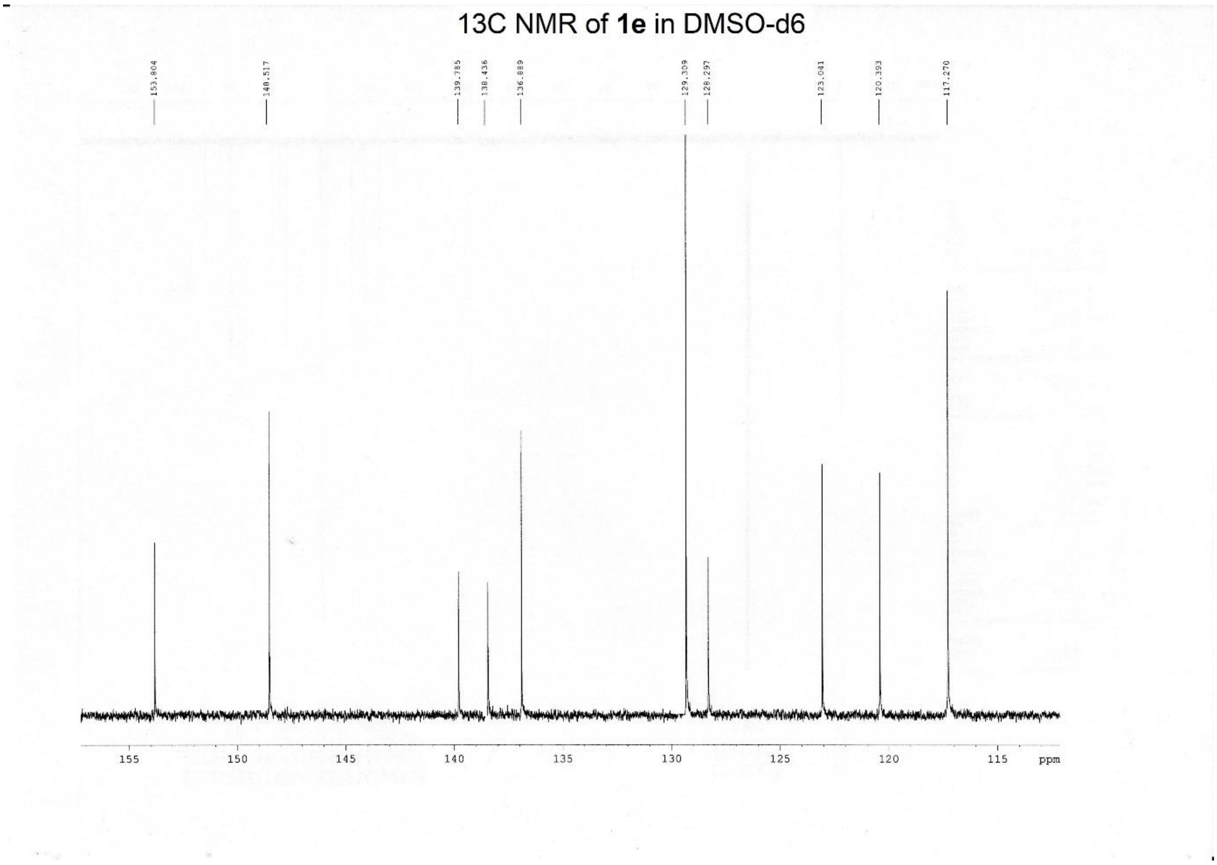

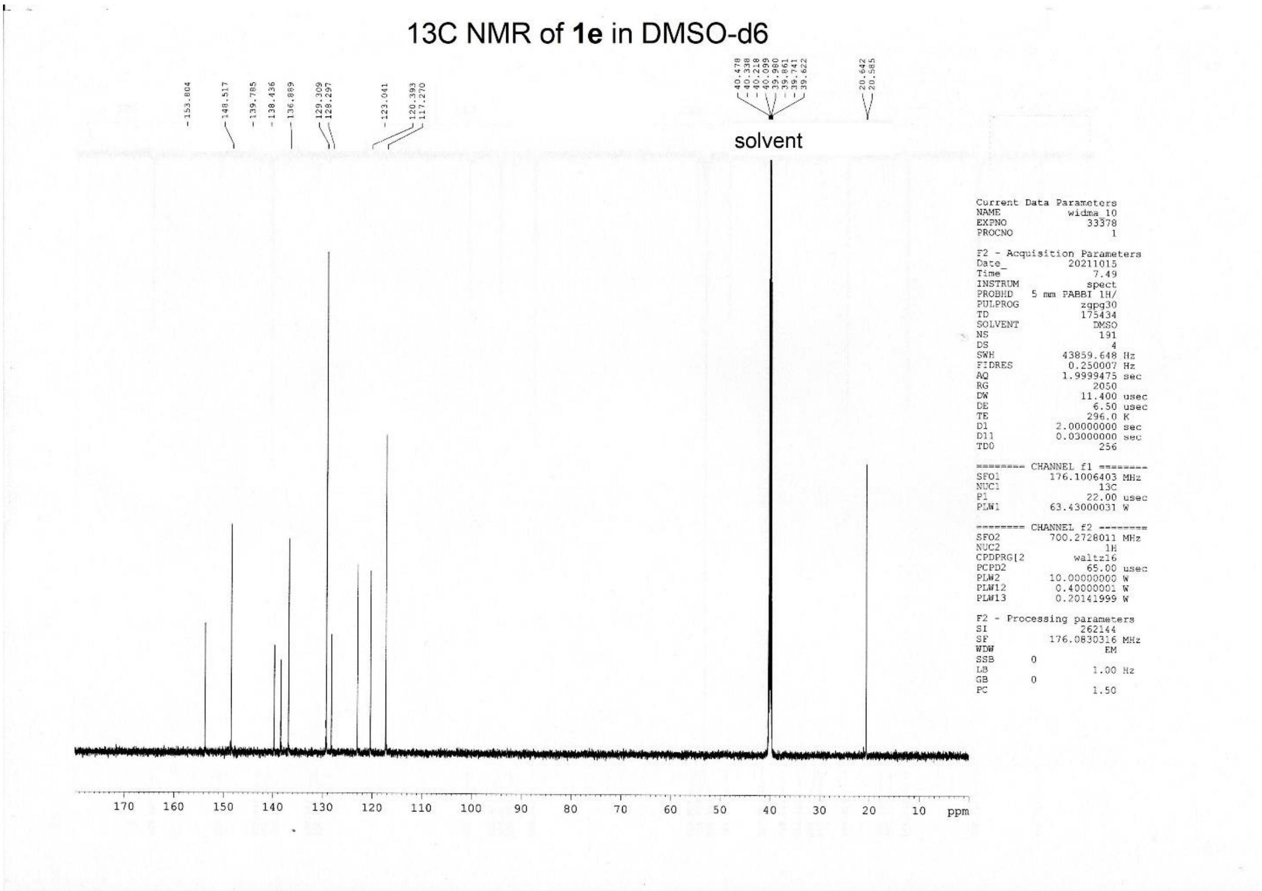

Figure S18. ^{13}C NMR spectrum of **1e** (in DMSO-d$_6$), with enlarged fragment at the top

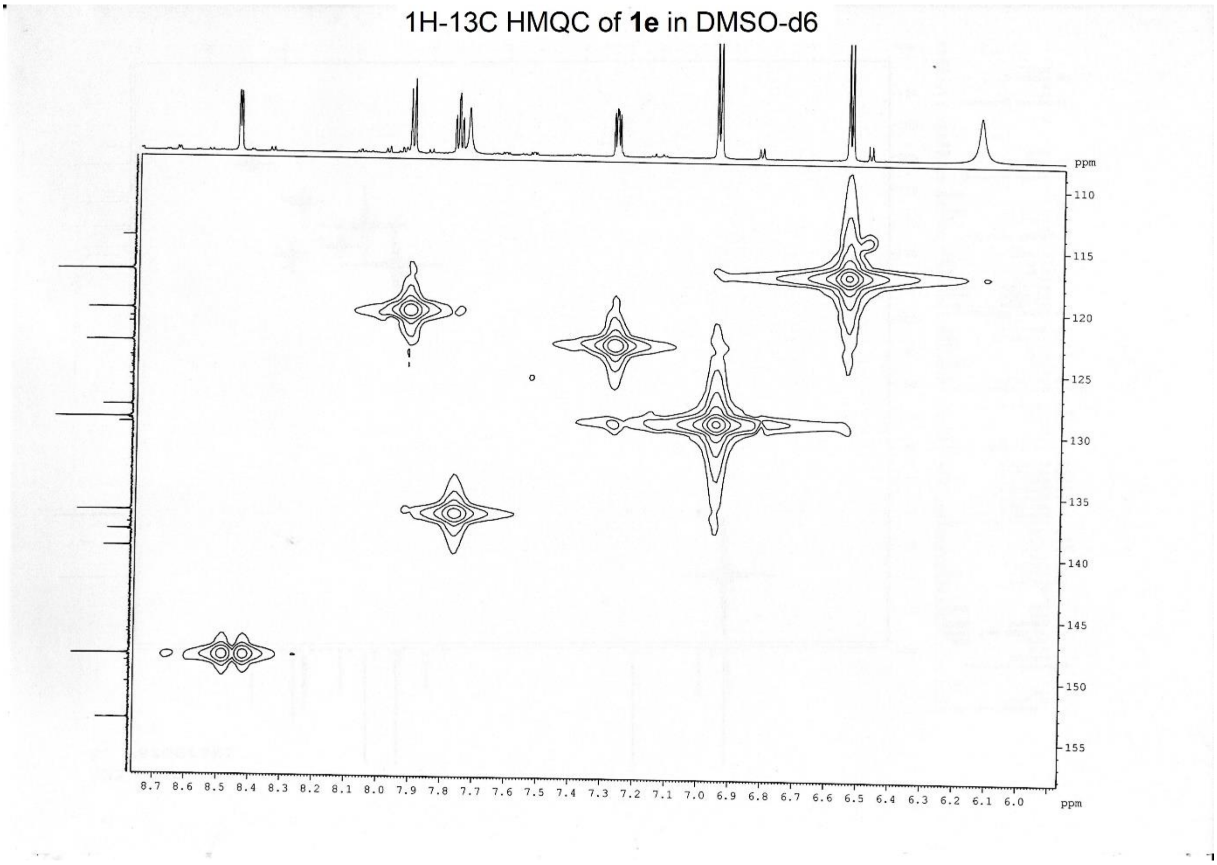

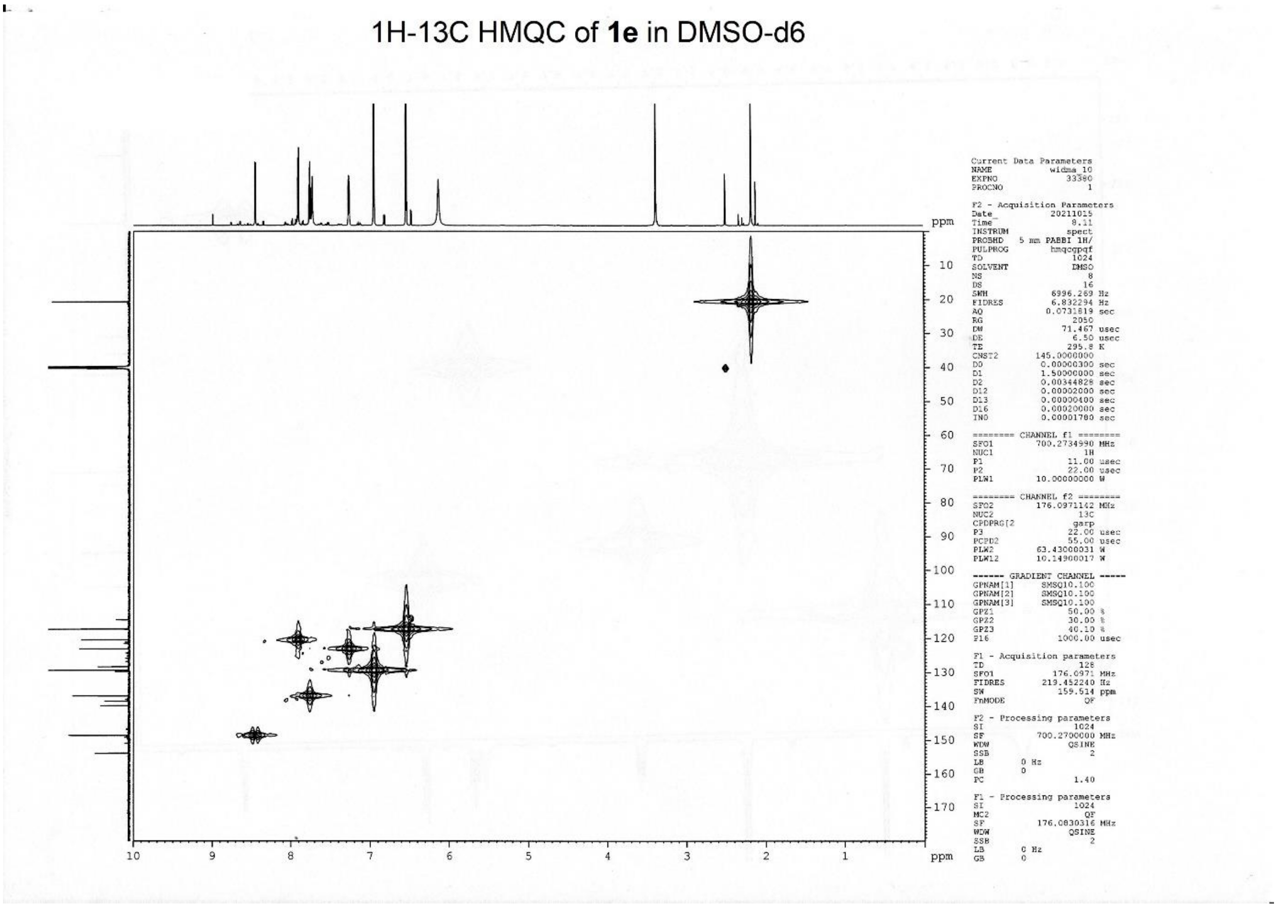

Figure S19. ^{1}H-^{13}C HMQC spectrum of **1e** (in DMSO-d_6), with enlarged fragment at the top

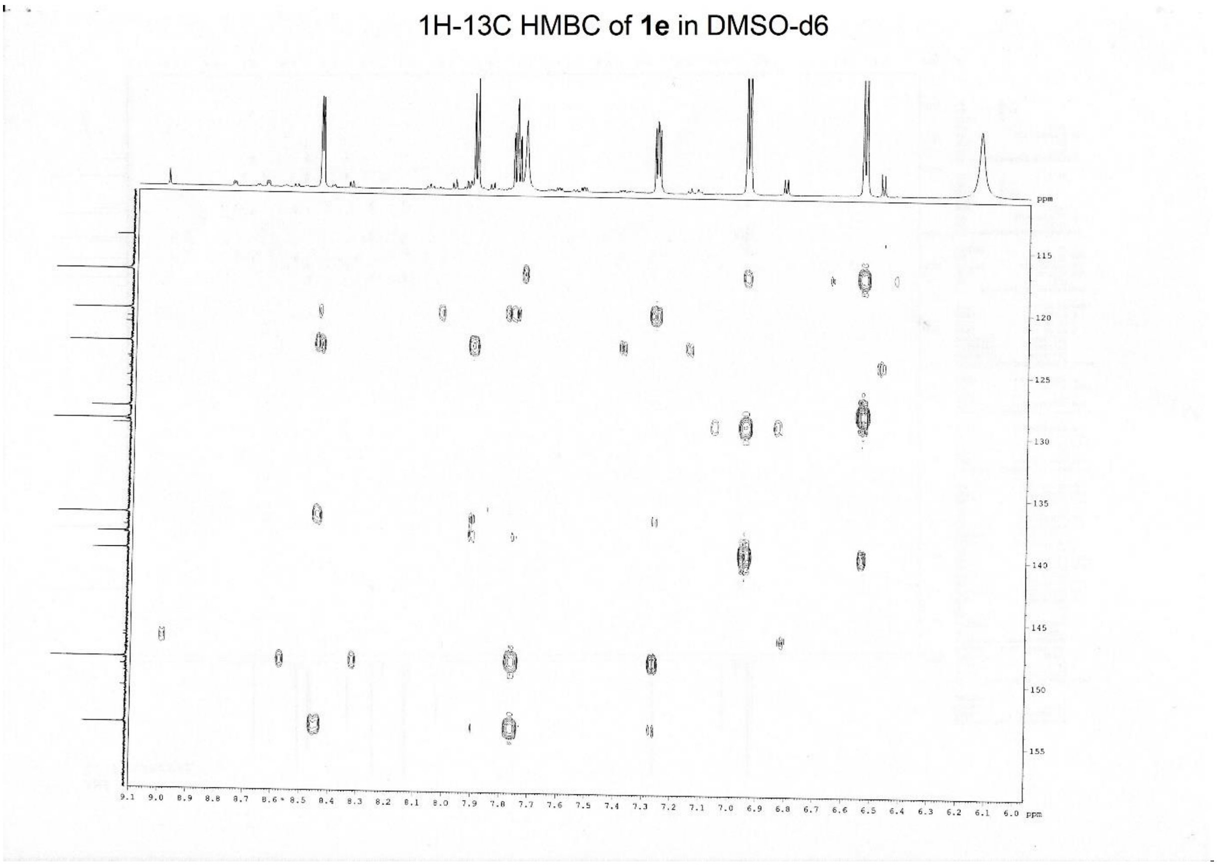

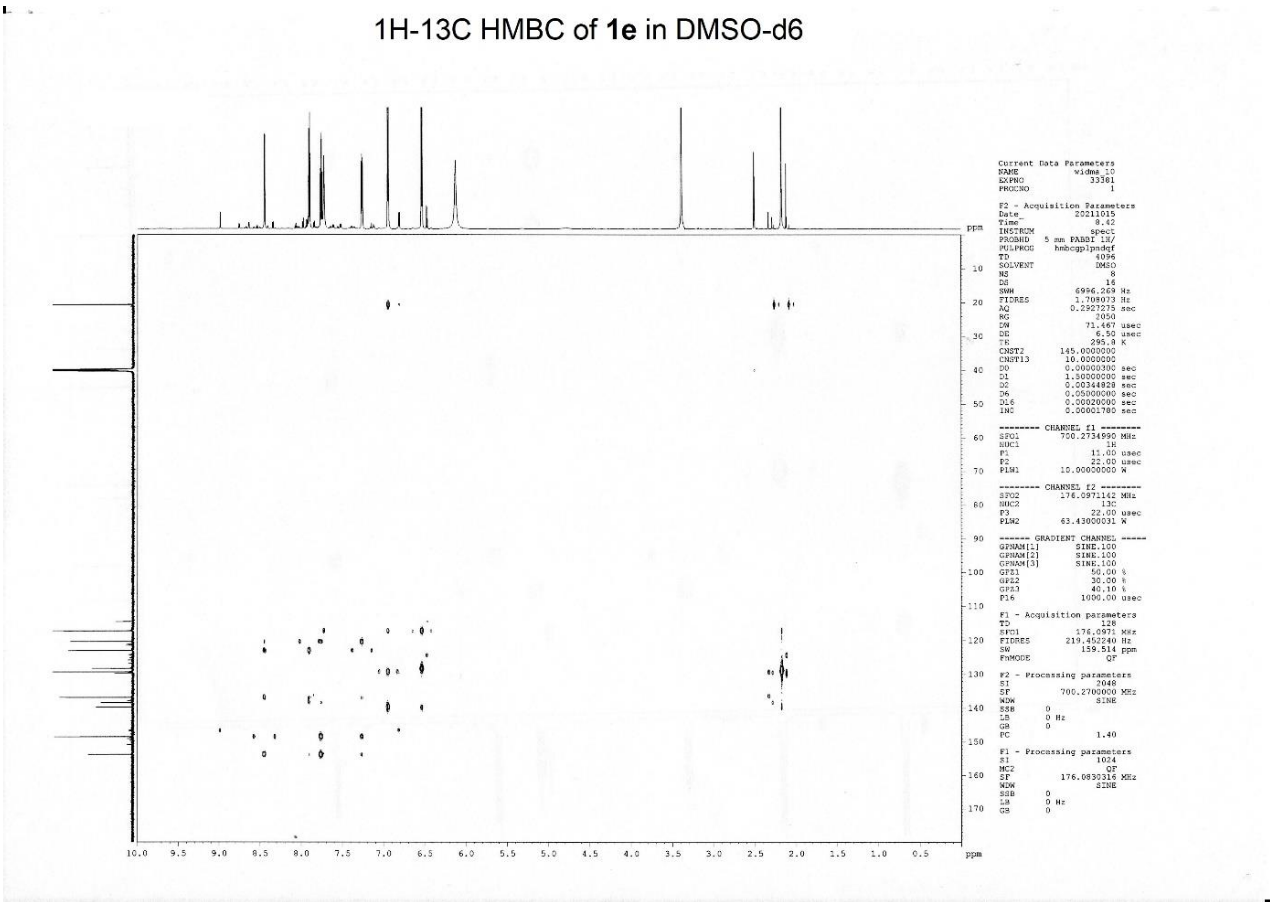

Figure S20. ^{1}H-^{13}C HMBC spectrum of **1e** (in DMSO-d_6), with enlarged fragment at the top

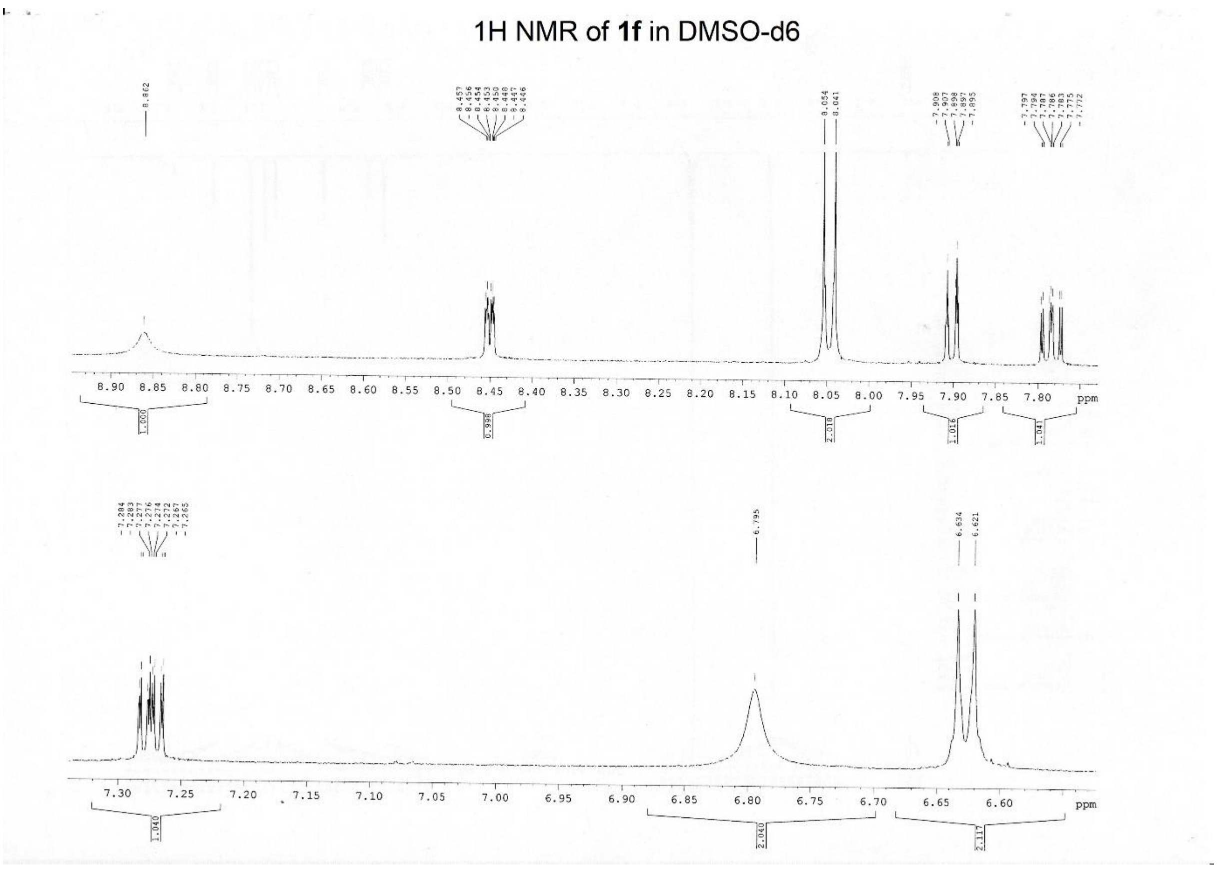

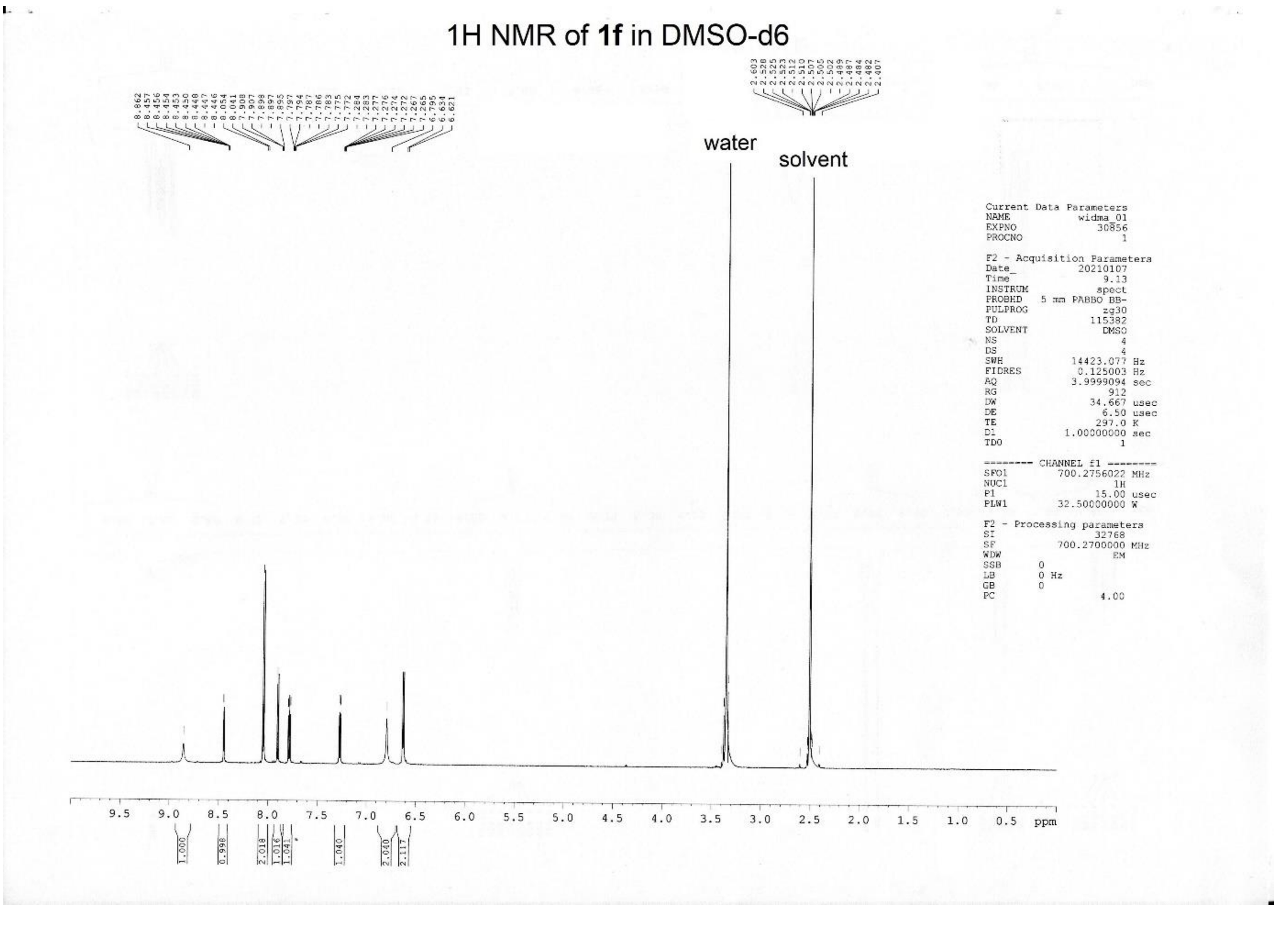

Figure S21. ^{1}H NMR spectrum of **1f** (in DMSO-d_6), with enlarged fragment at the top

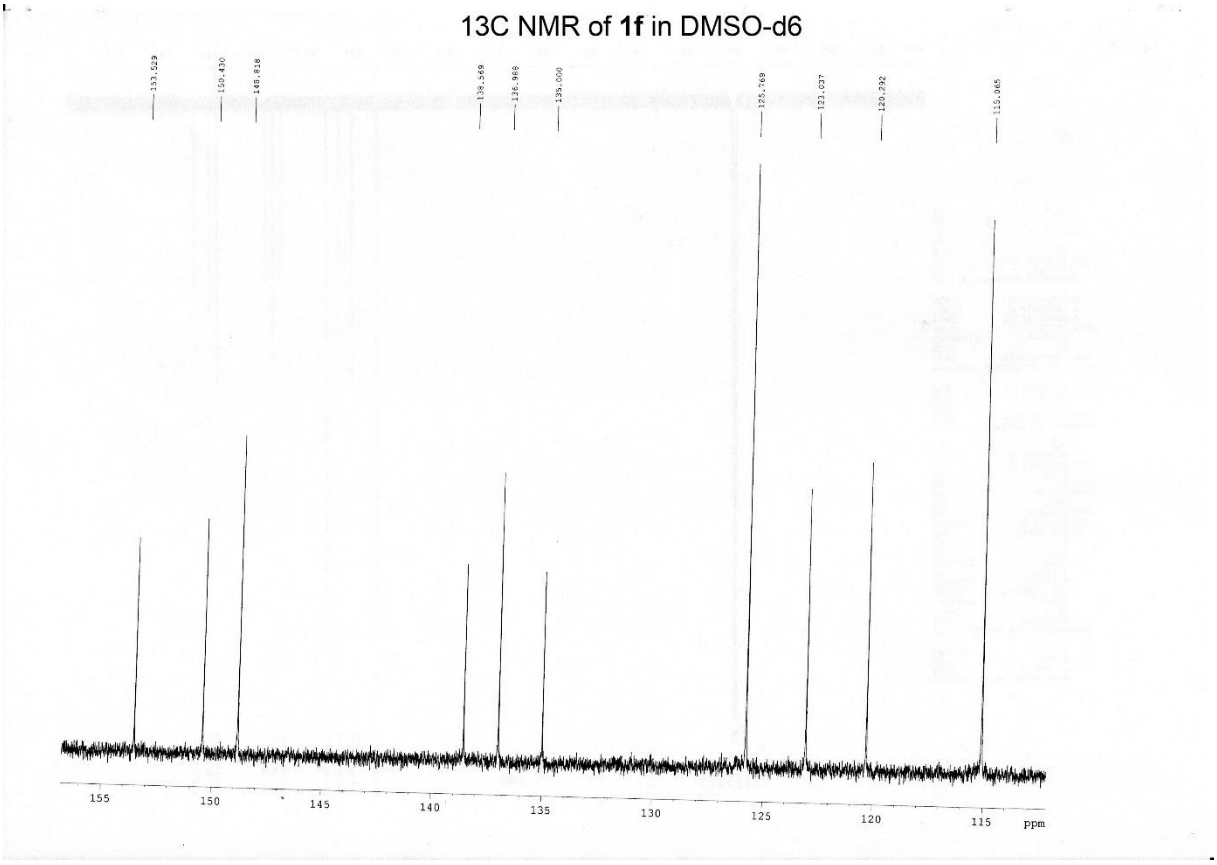

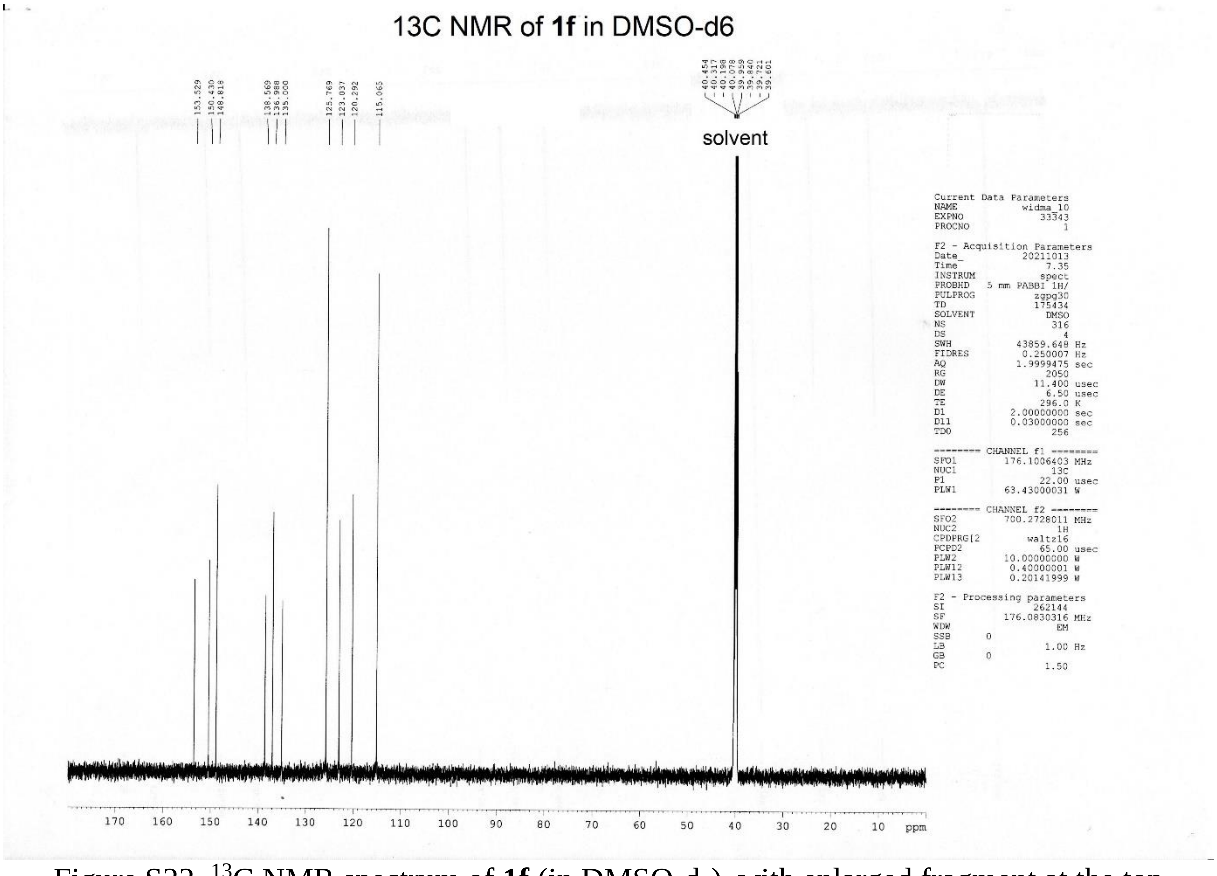

Figure S22. ^{13}C NMR spectrum of **1f** (in DMSO-d$_6$), with enlarged fragment at the top

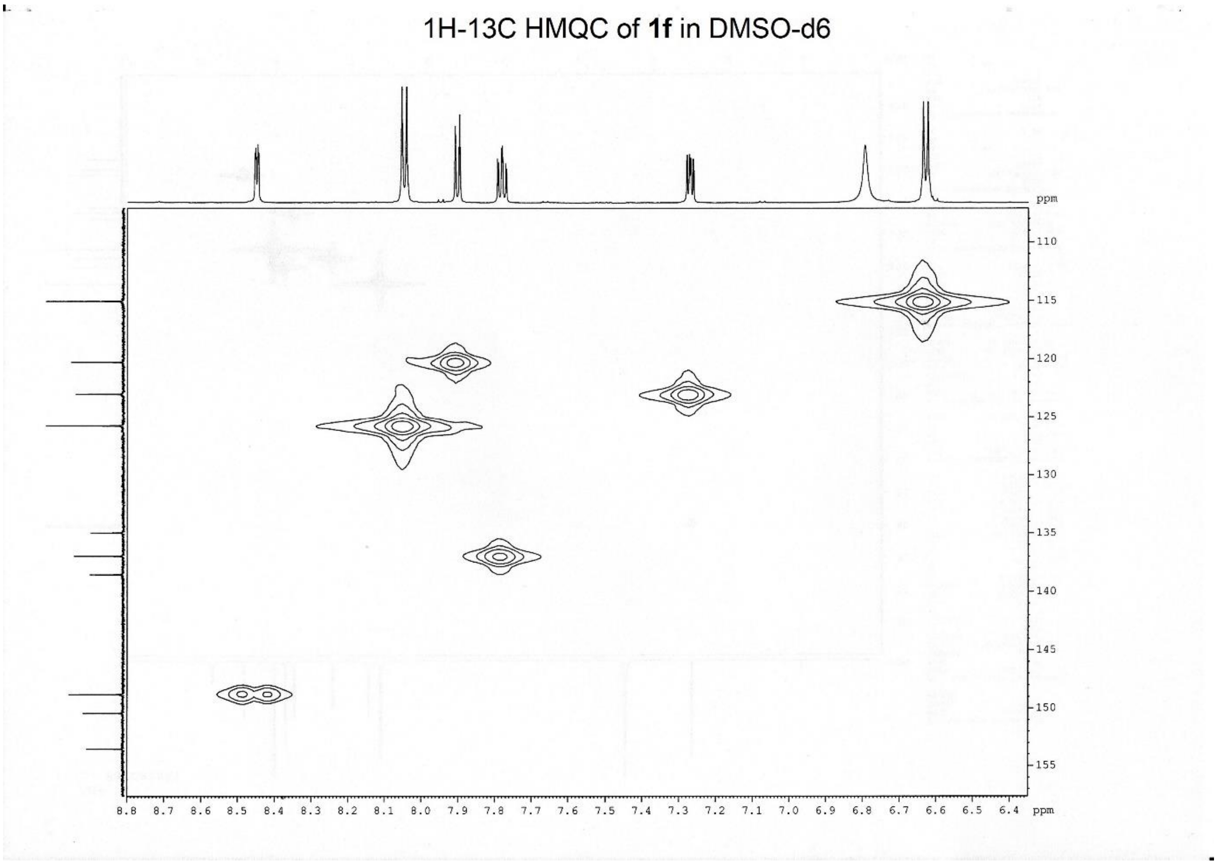

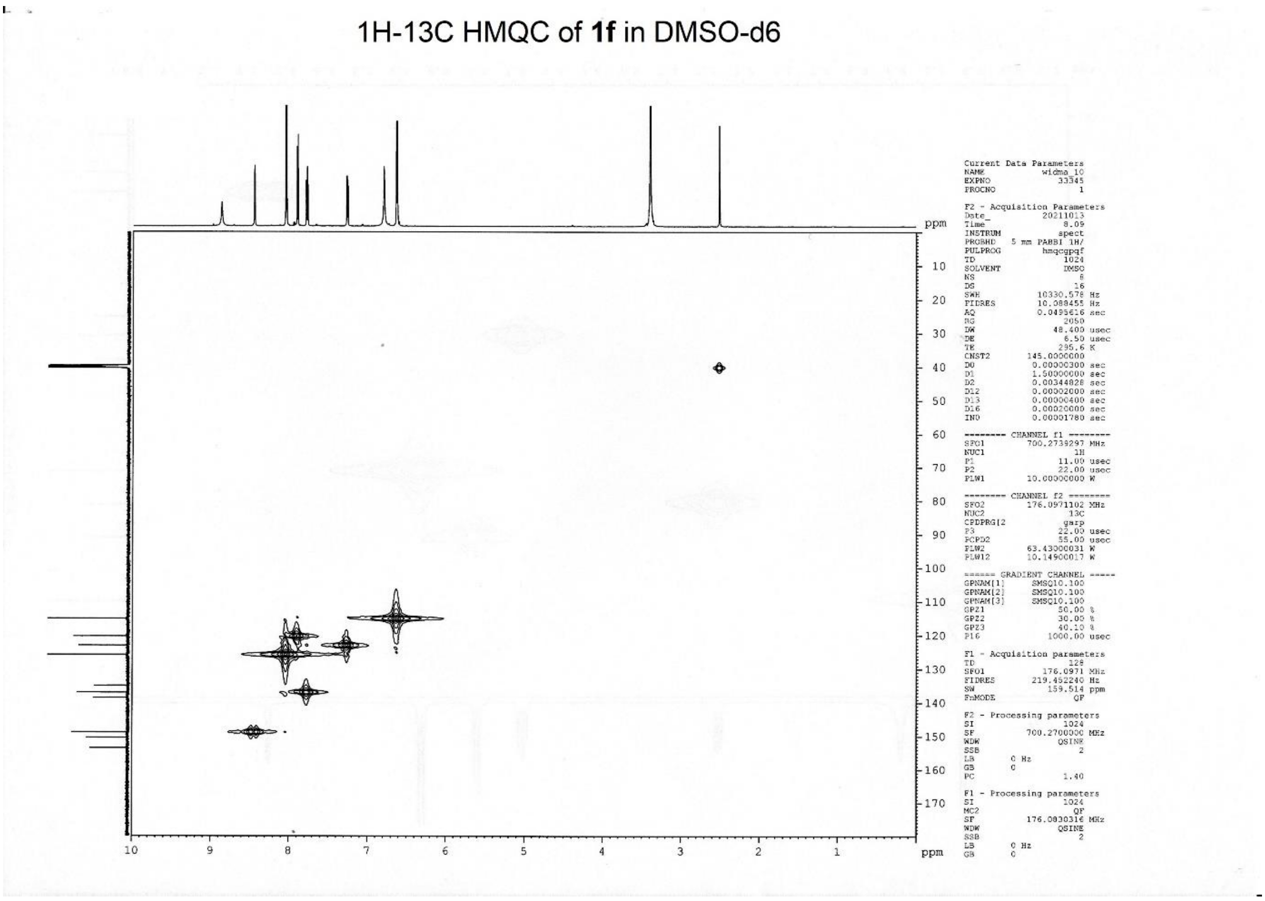

Figure S23. ^{1}H-^{13}C HMQC spectrum of **1f** (in DMSO-d_6), with enlarged fragment at the top

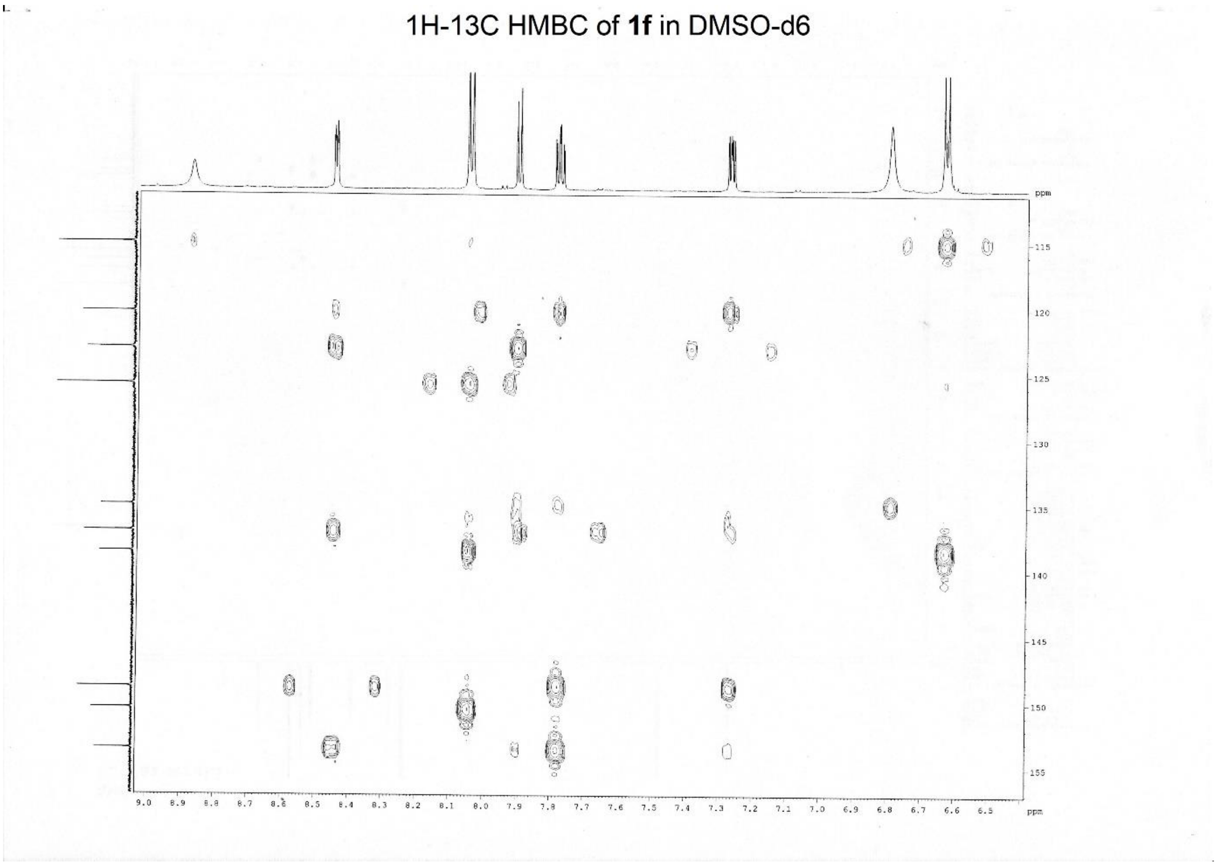

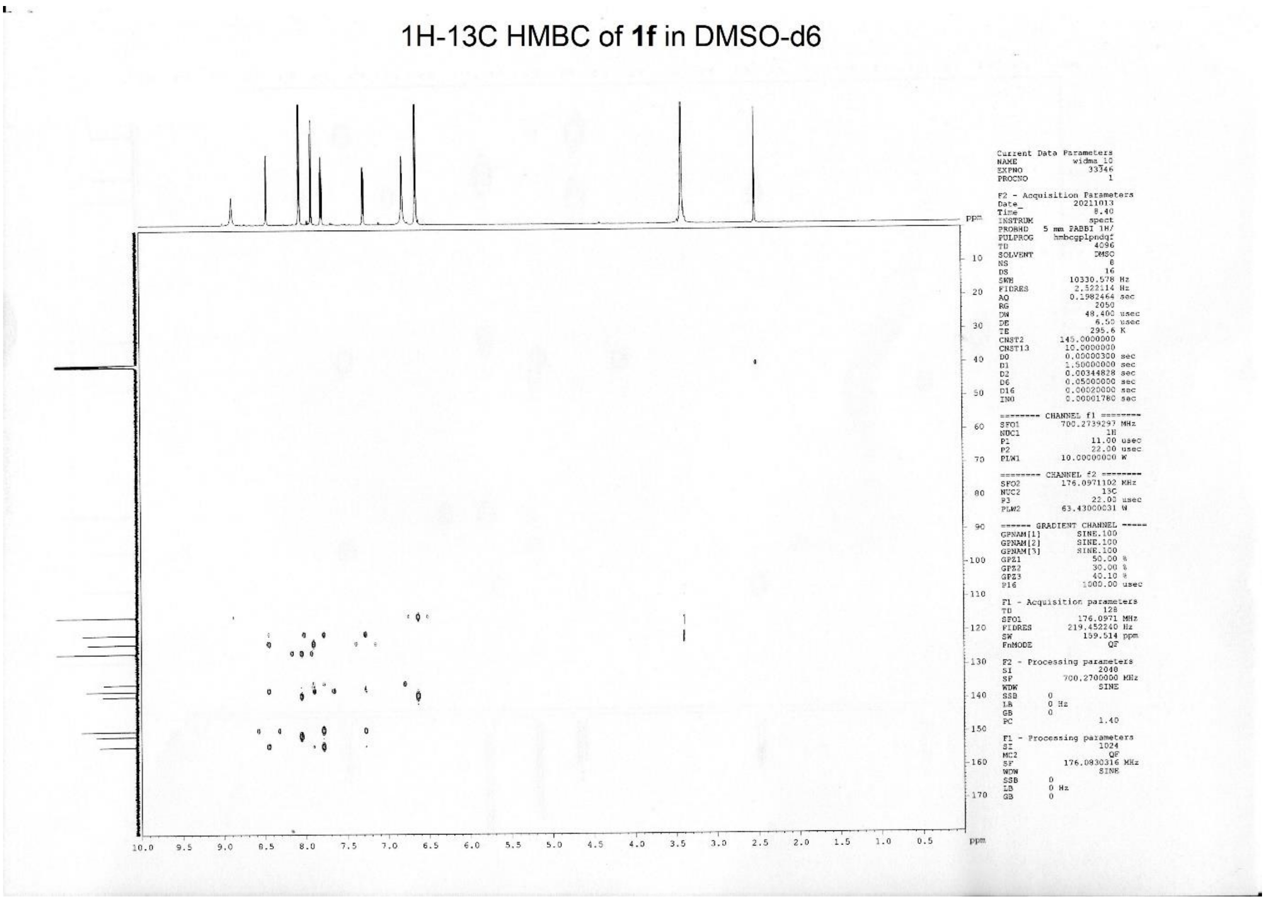

Figure S24. ^{1}H-^{13}C HMBC spectrum of **1f** (in DMSO-d_6), with enlarged fragment at the top

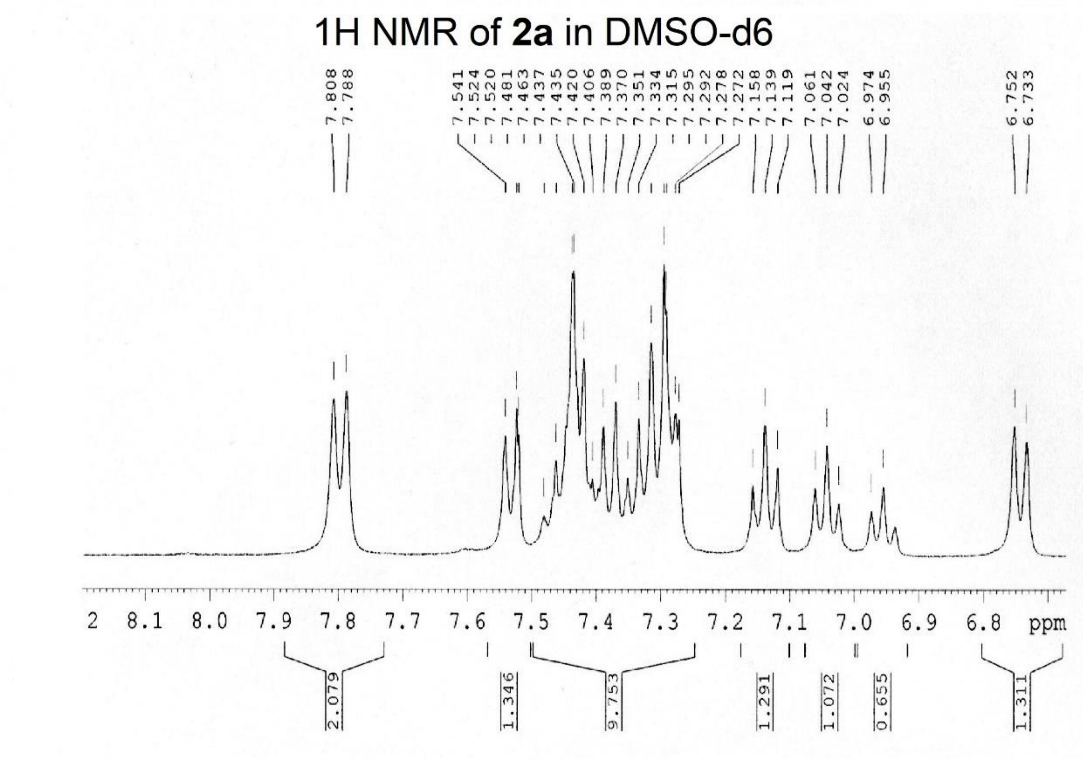

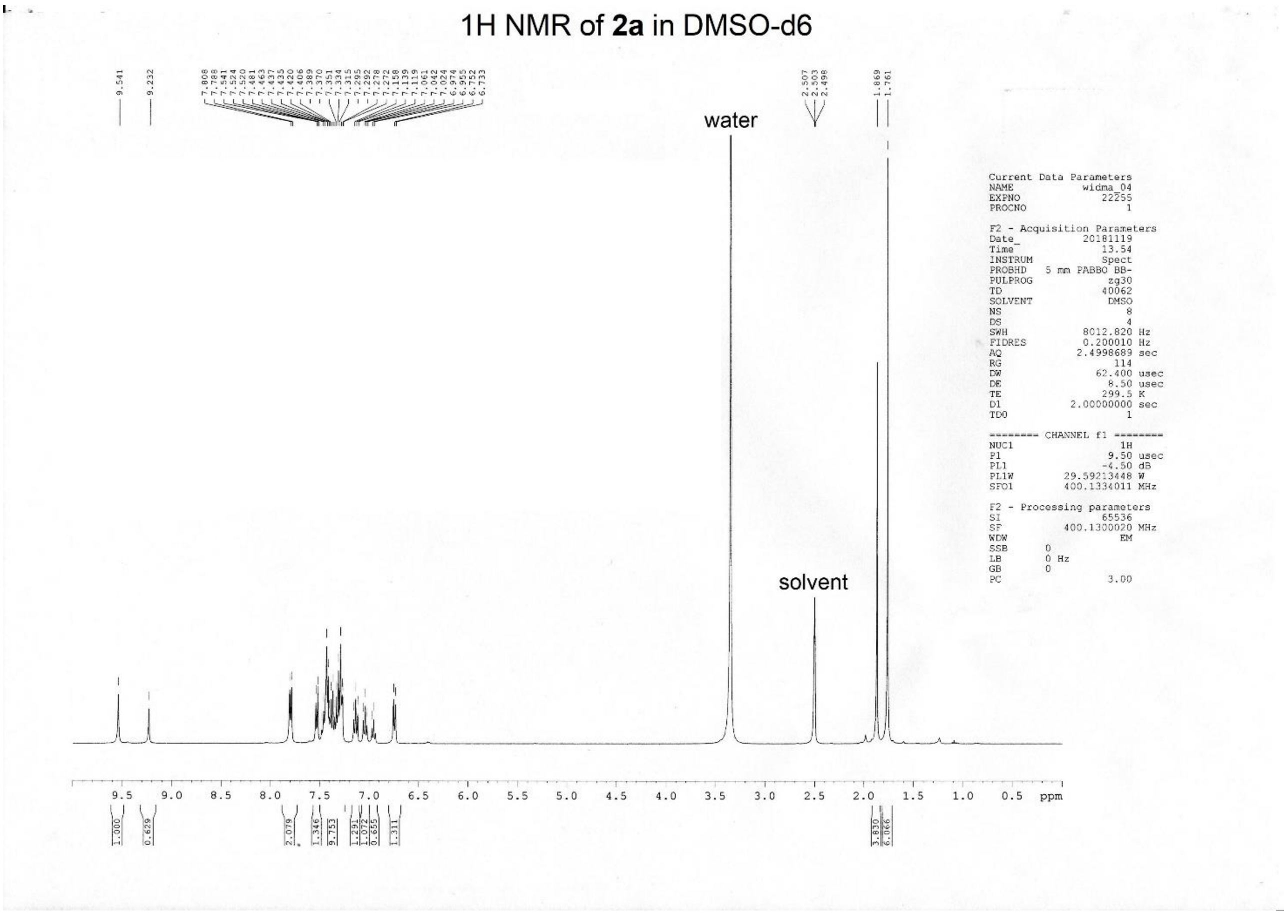

Figure S25. ^{1}H NMR spectrum of **2a** (in DMSO-d_6), with enlarged fragment at the top

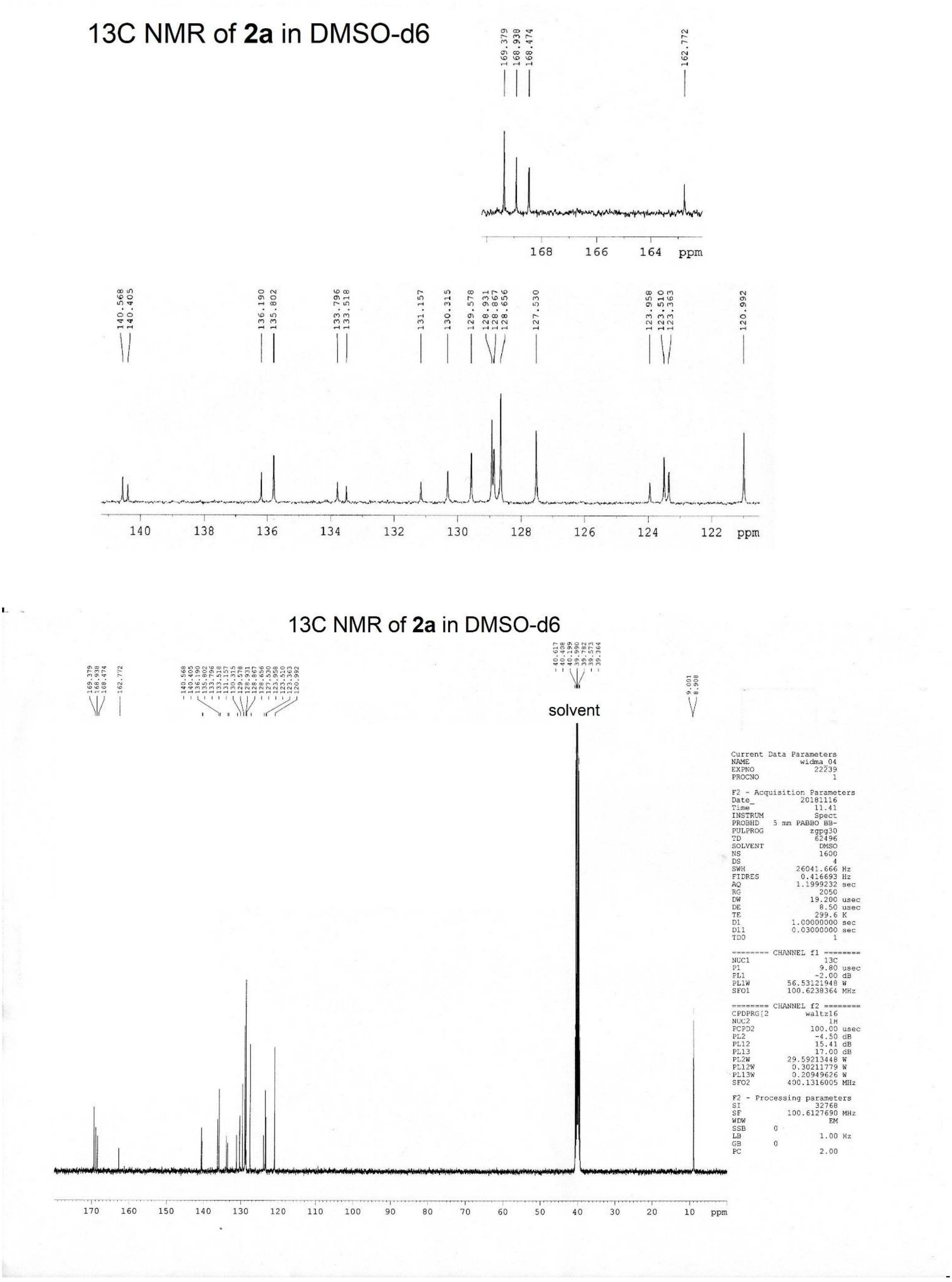

Figure S26. ^{13}C NMR spectrum of **2a** (in DMSO-d$_6$), with enlarged fragment at the top

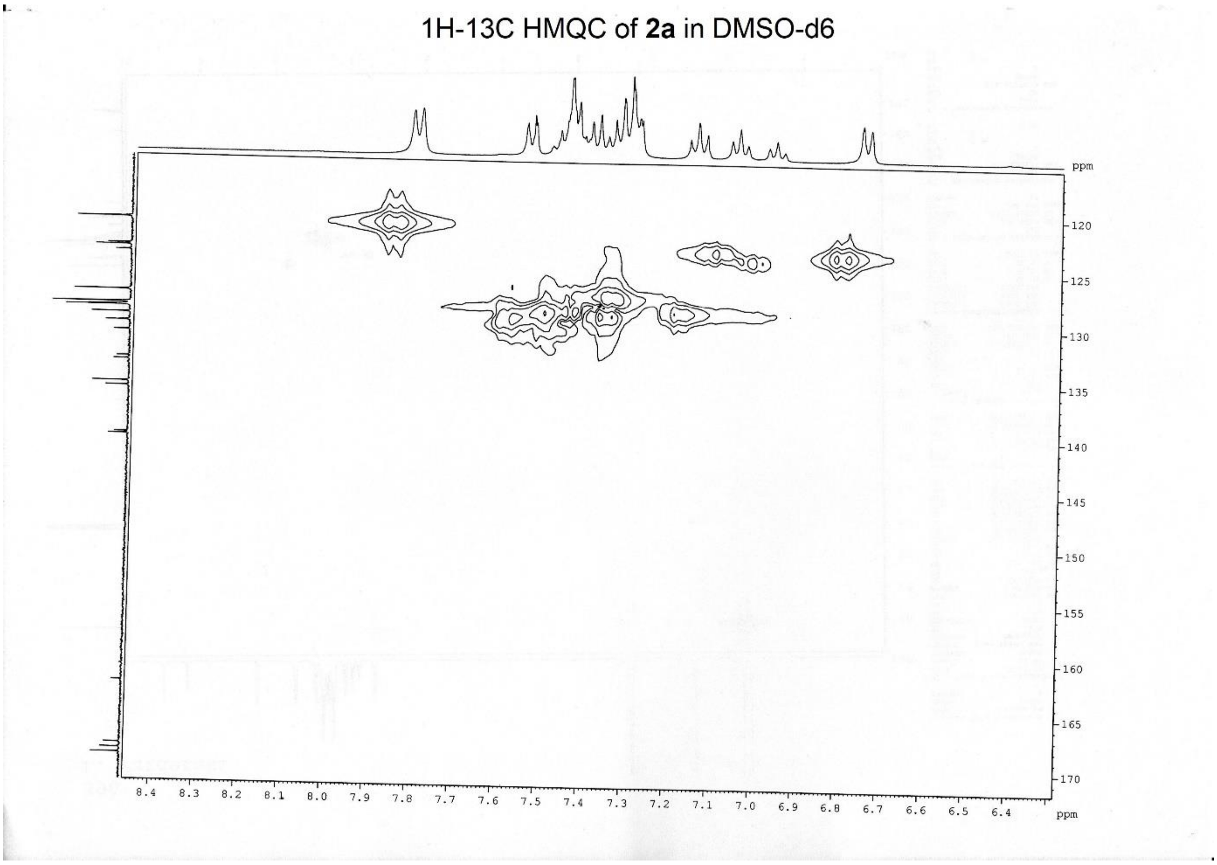

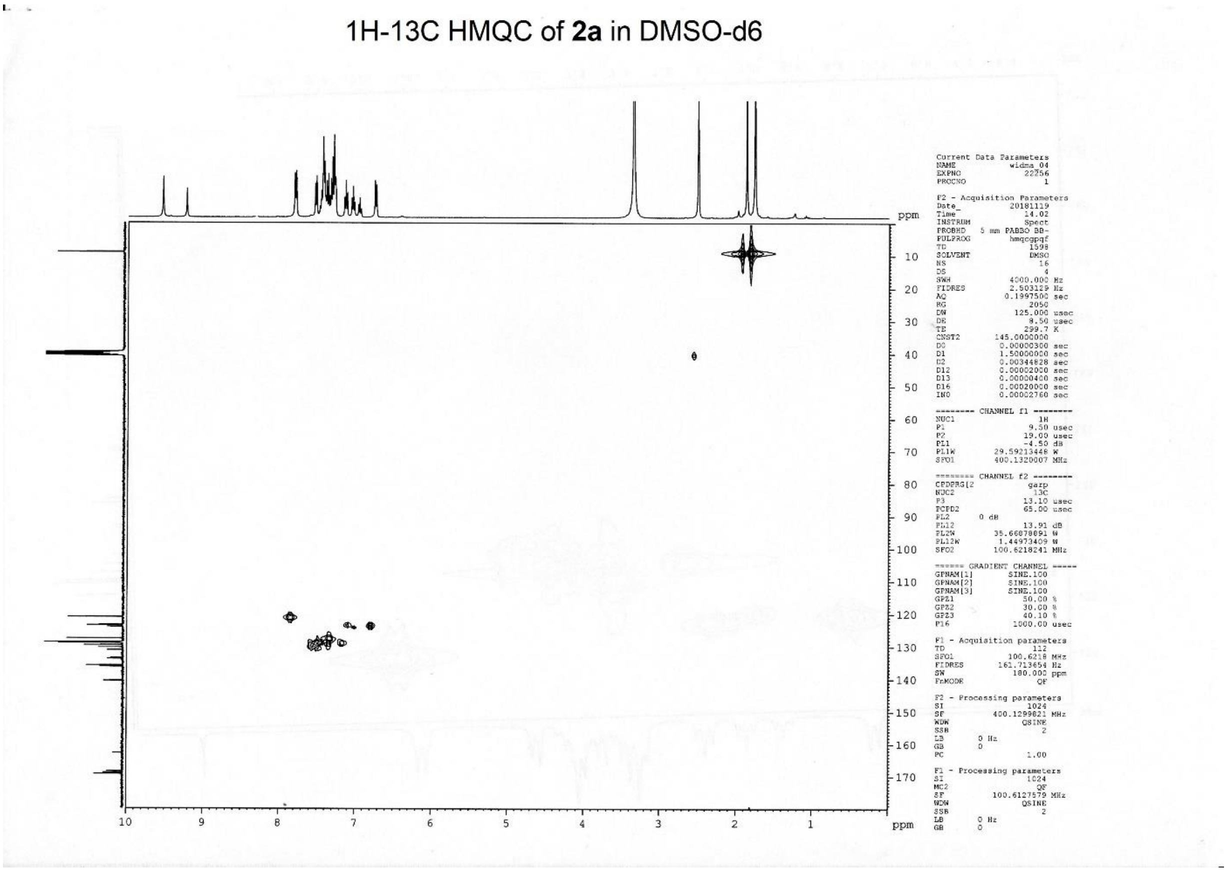

Figure S27. ^{1}H-^{13}C HMQC spectrum of **2a** (in DMSO-d_6), with enlarged fragment at the top

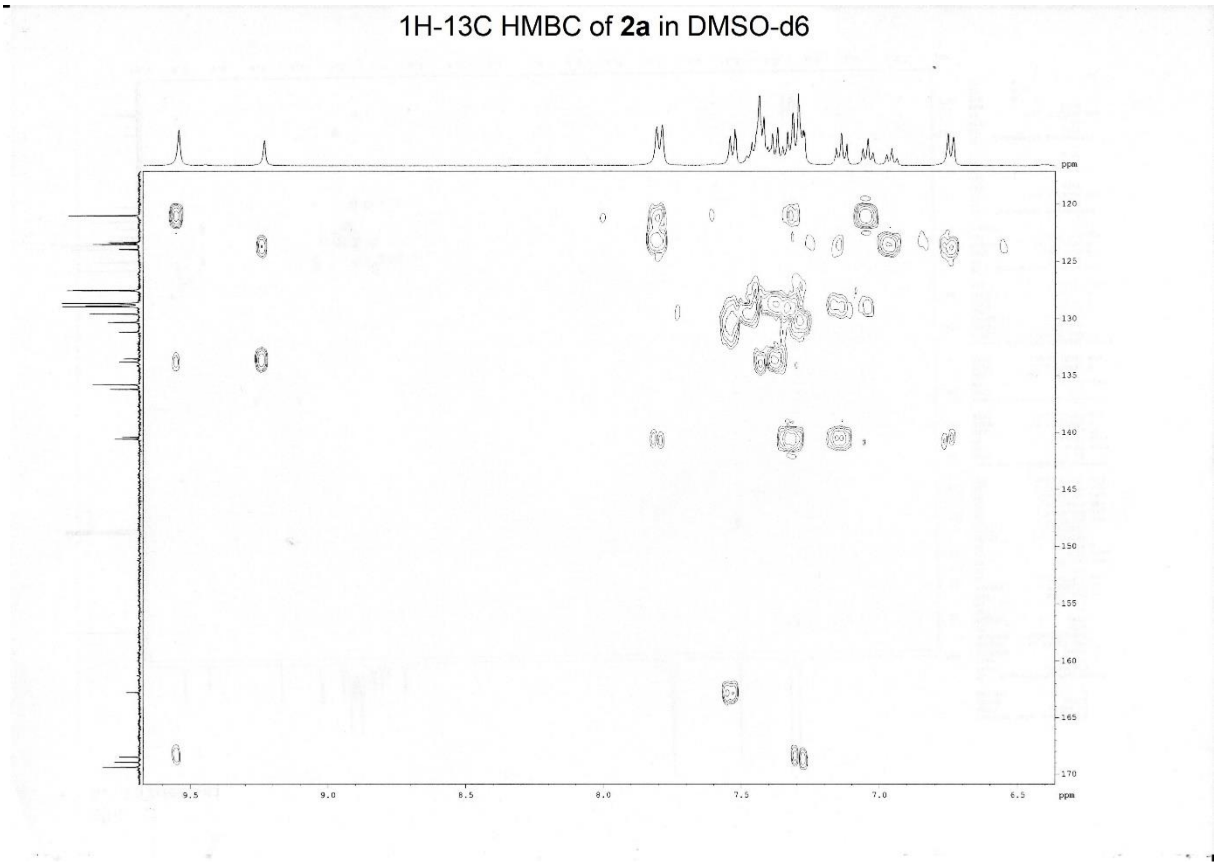

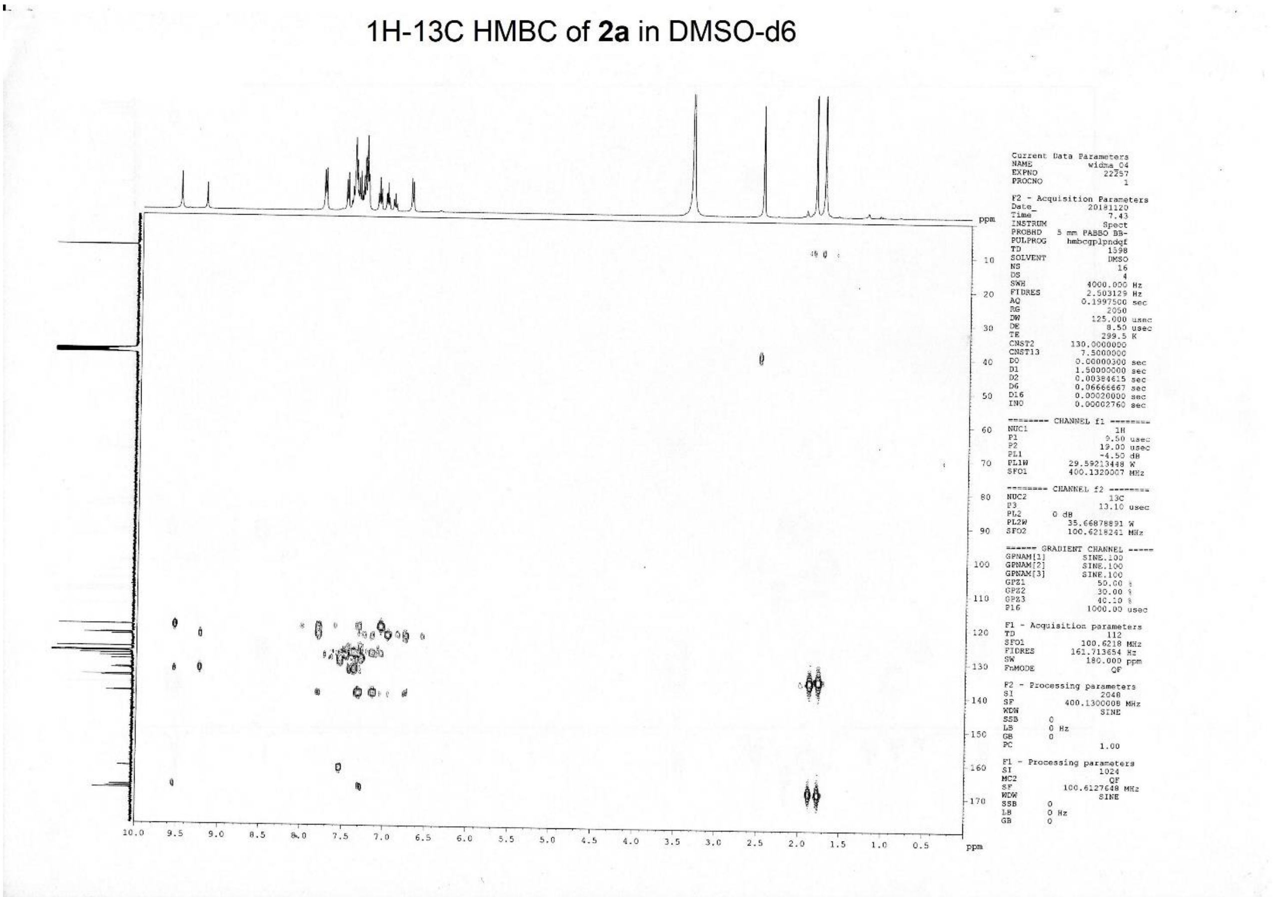

Figure S28. ^{1}H-^{13}C HMBC spectrum of **2a** (in DMSO-d_6), with enlarged fragment at the top

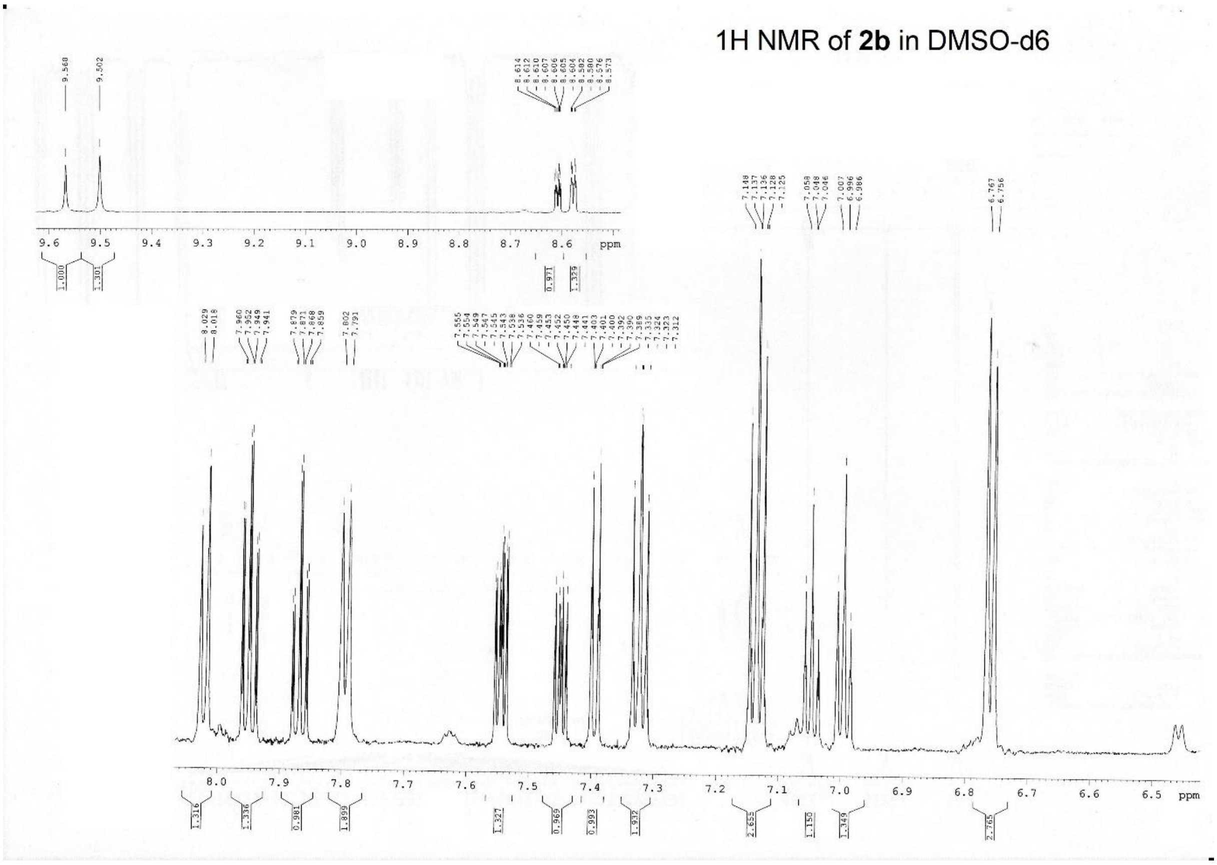

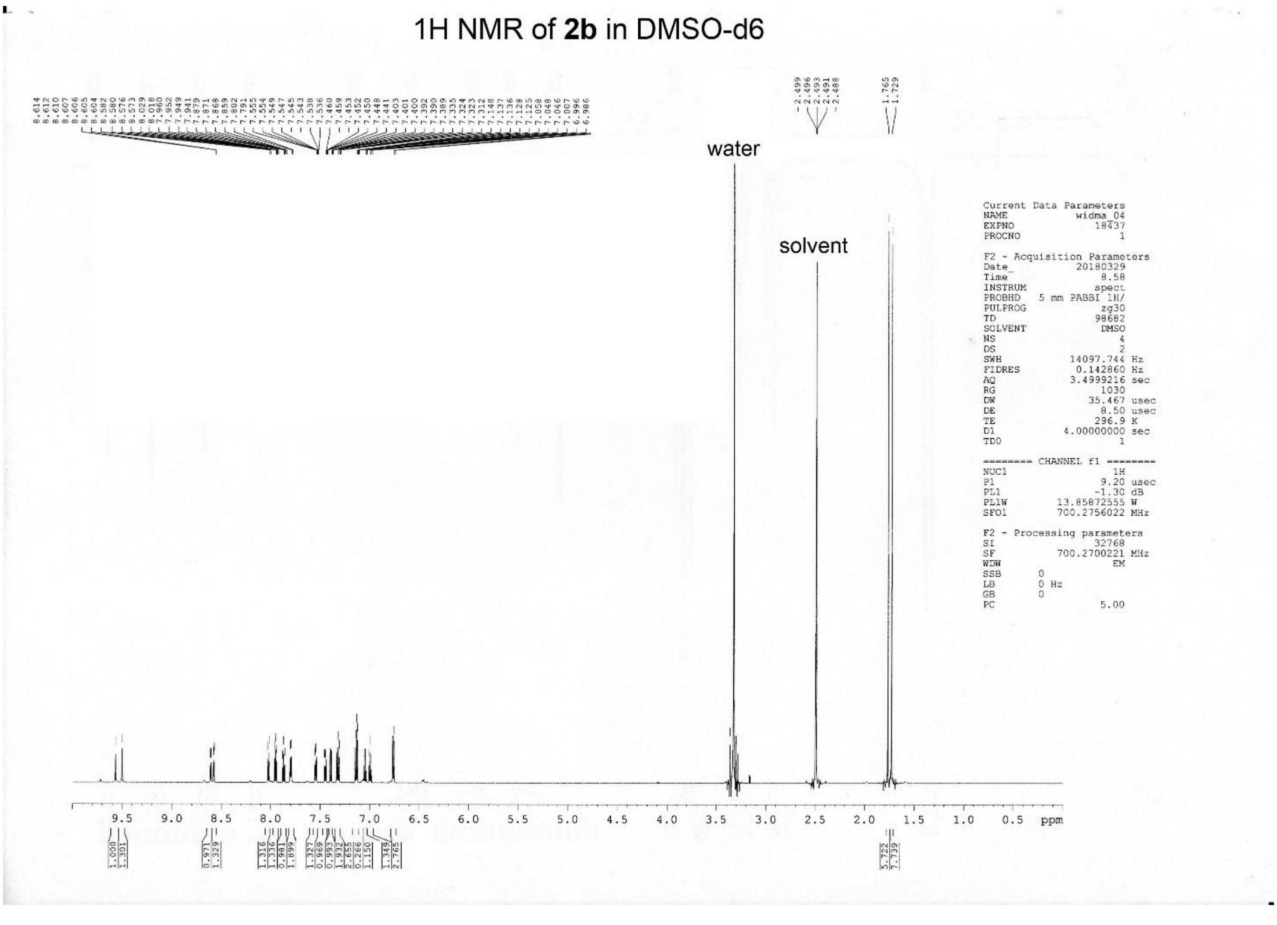

Figure S29. ^{1}H NMR spectrum of **2b** (in DMSO-d_6), with enlarged fragment at the top

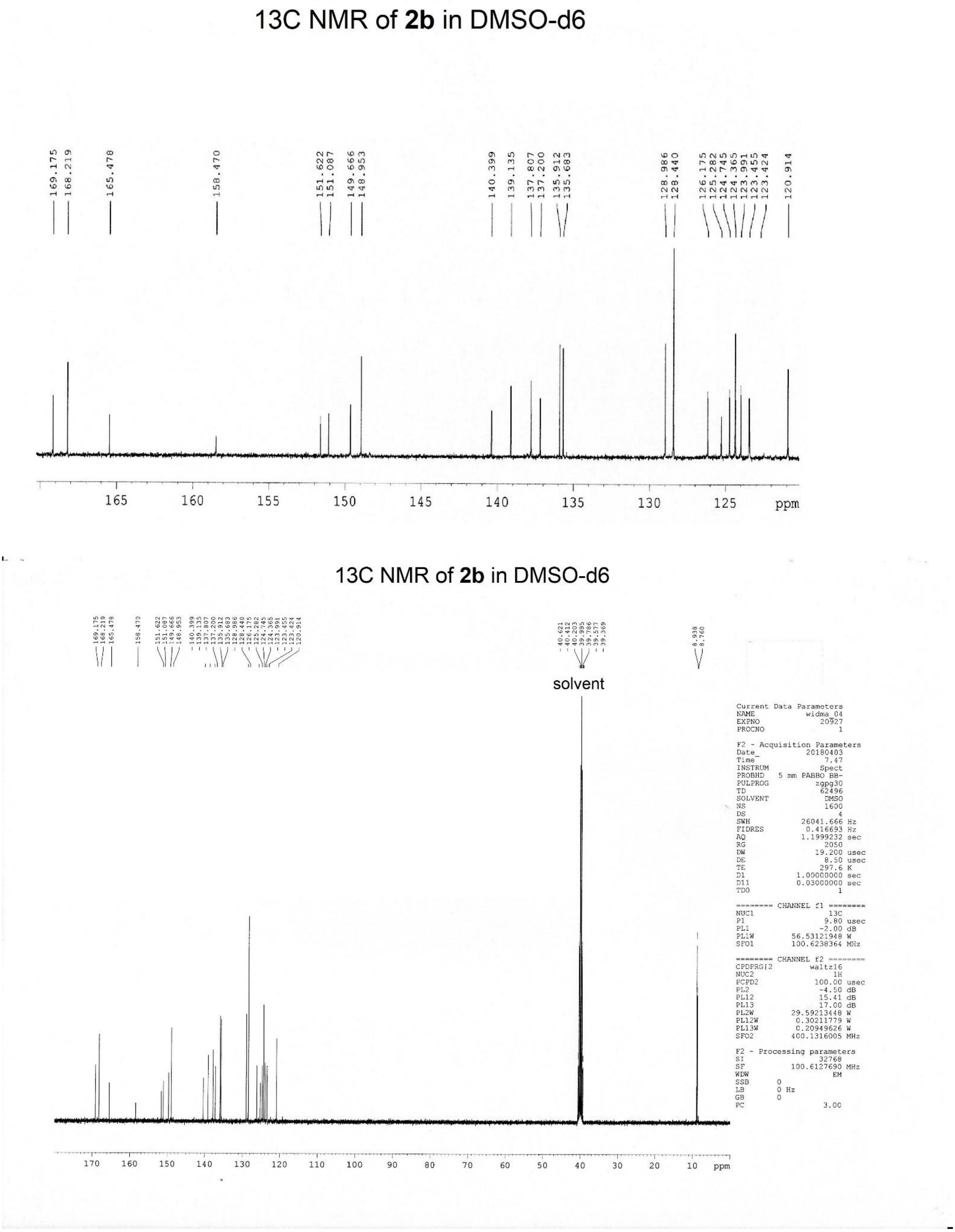

Figure S30. ^{13}C NMR spectrum of **2b** (in DMSO-d_6), with enlarged fragment at the top

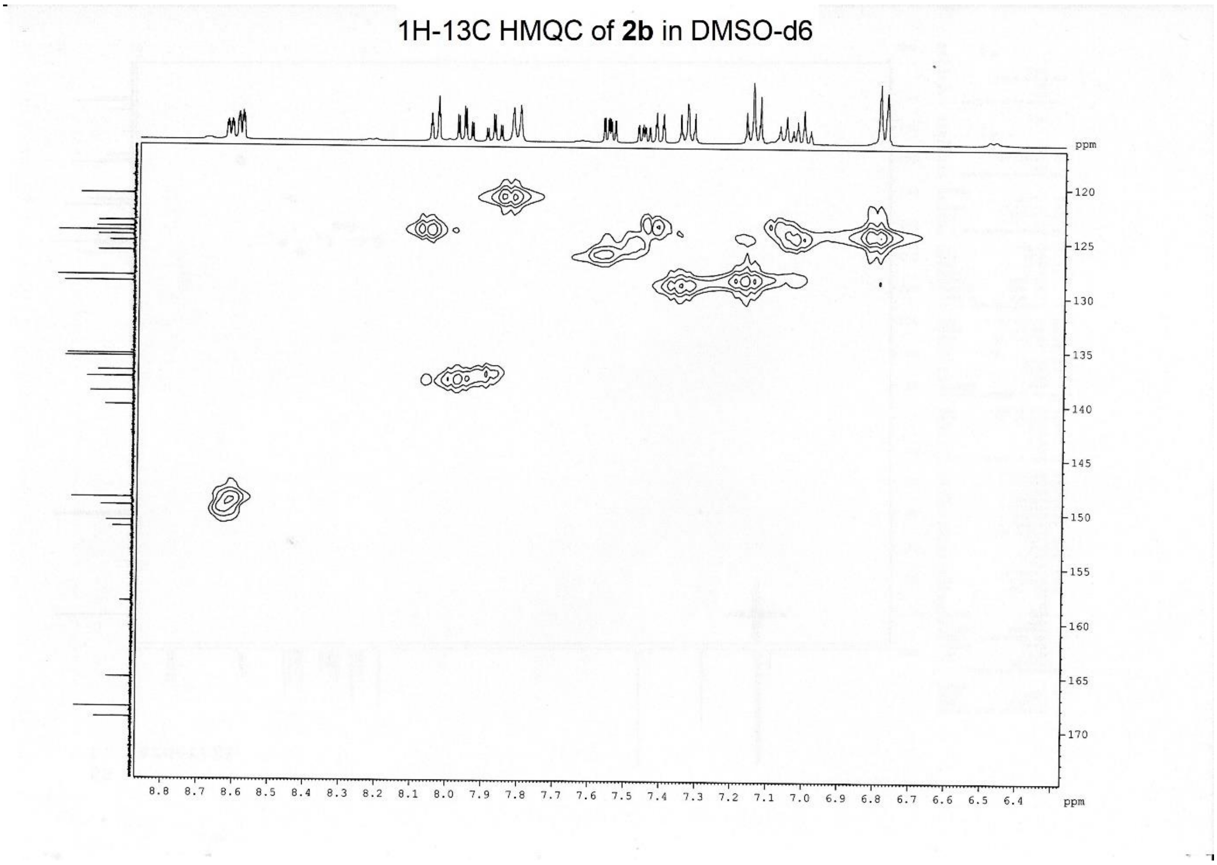

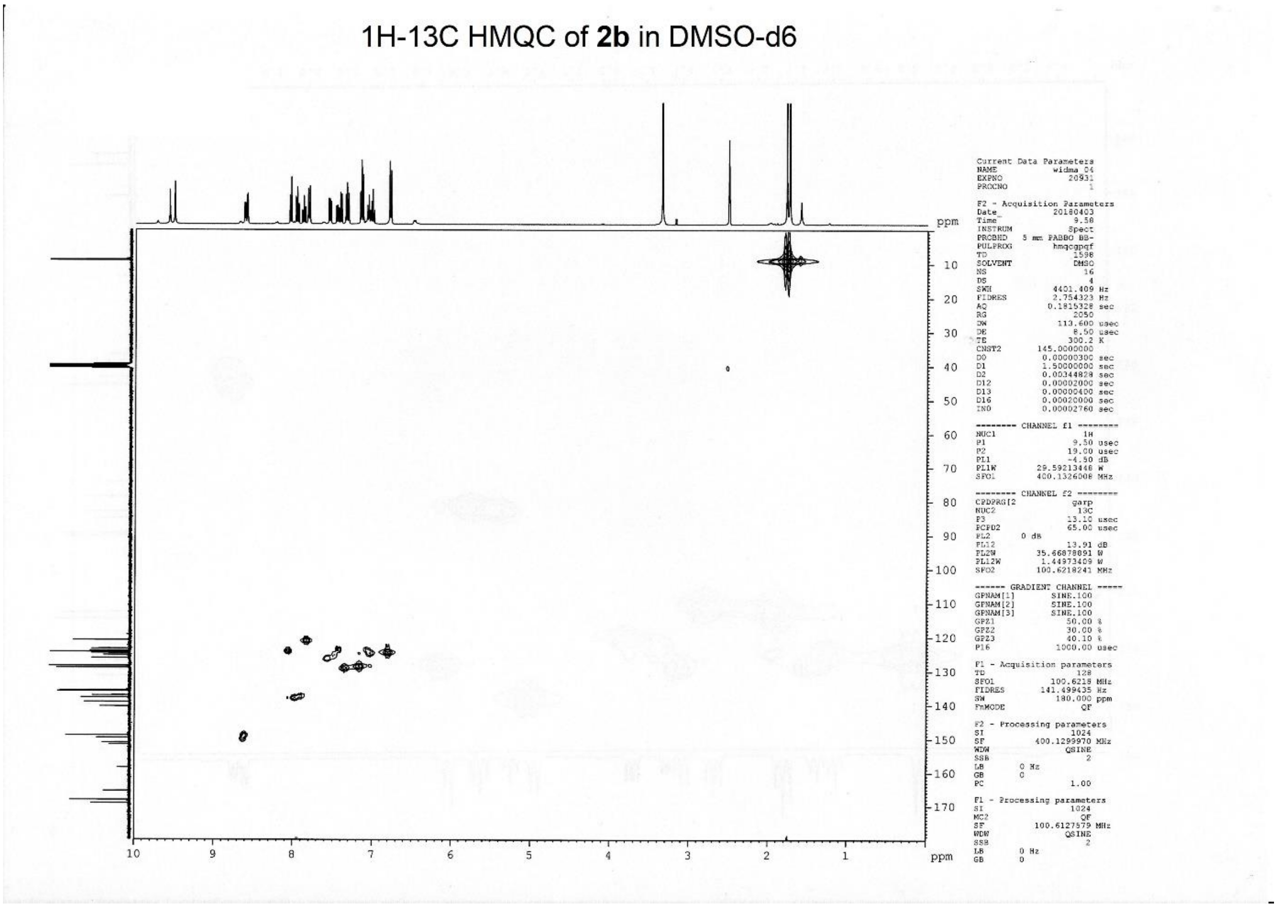

Figure S31. ^{1}H-^{13}C HMQC spectrum of **2b** (in DMSO-d_6), with enlarged fragment at the top

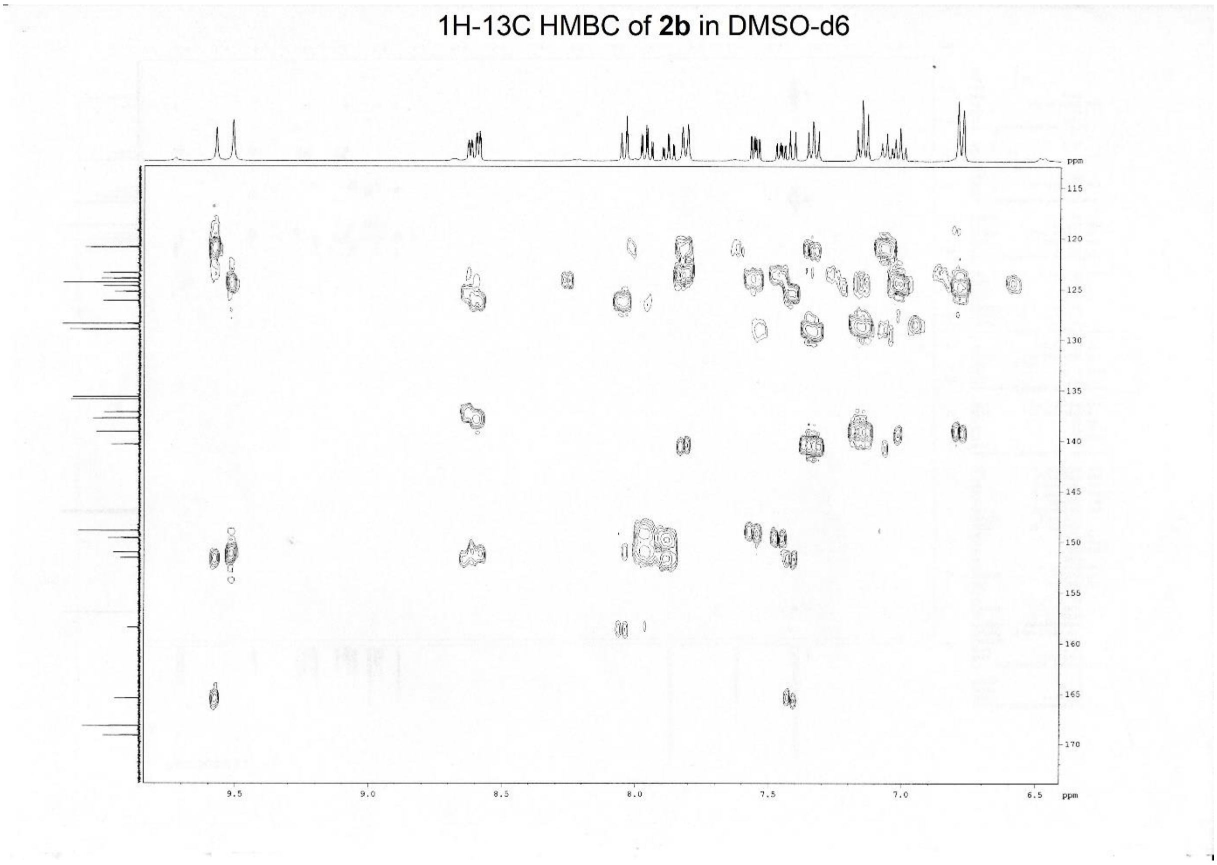

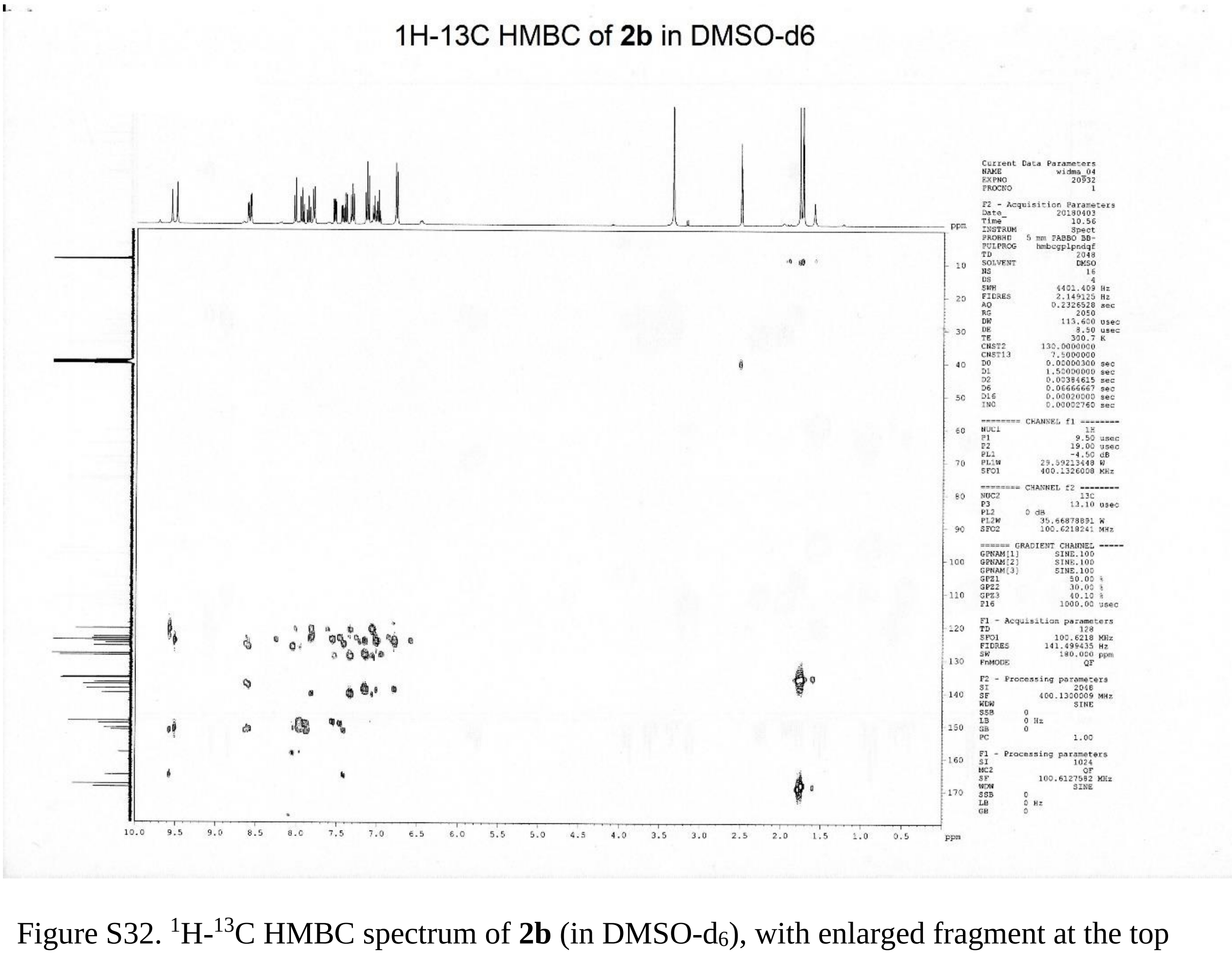

Figure S32. ^{1}H-^{13}C HMBC spectrum of **2b** (in DMSO-d_6), with enlarged fragment at the top

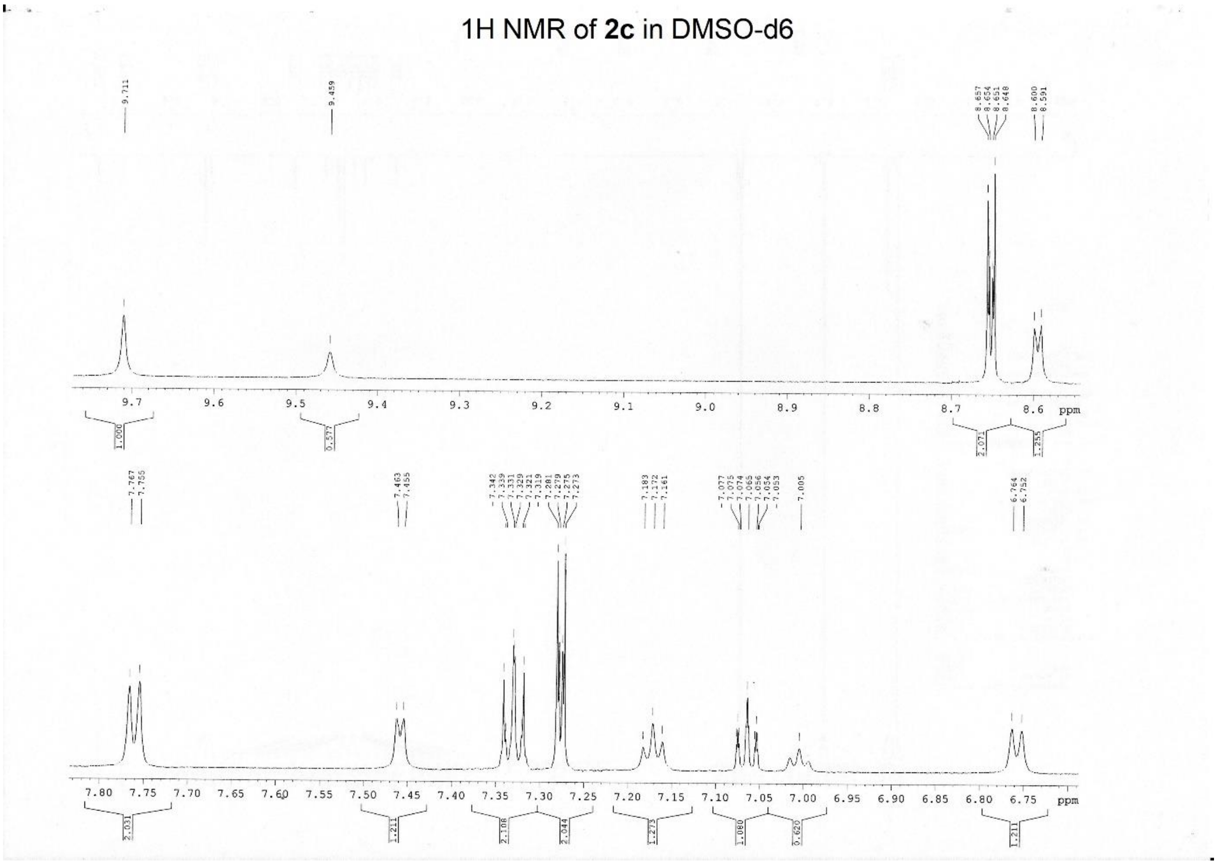

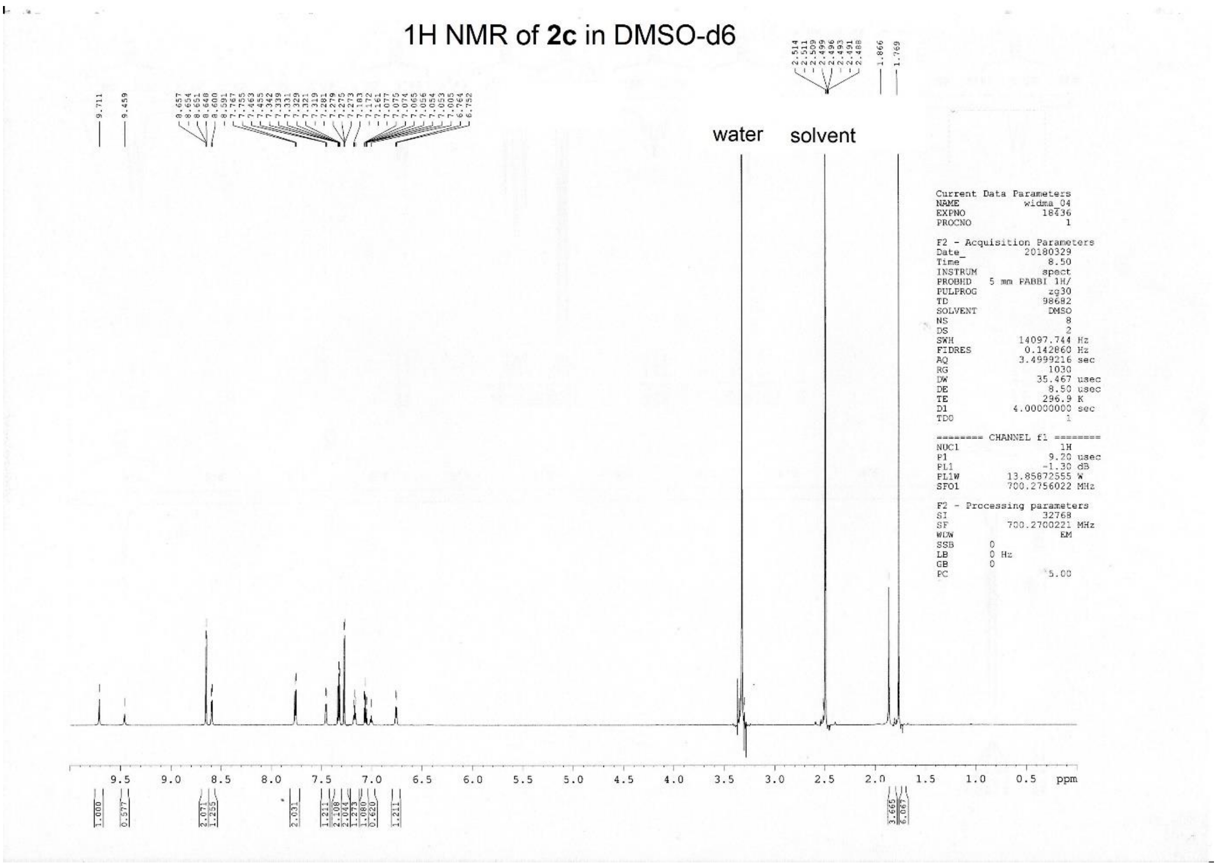

Figure S33. ^{1}H NMR spectrum of **2c** (in DMSO-d_6), with enlarged fragment at the top

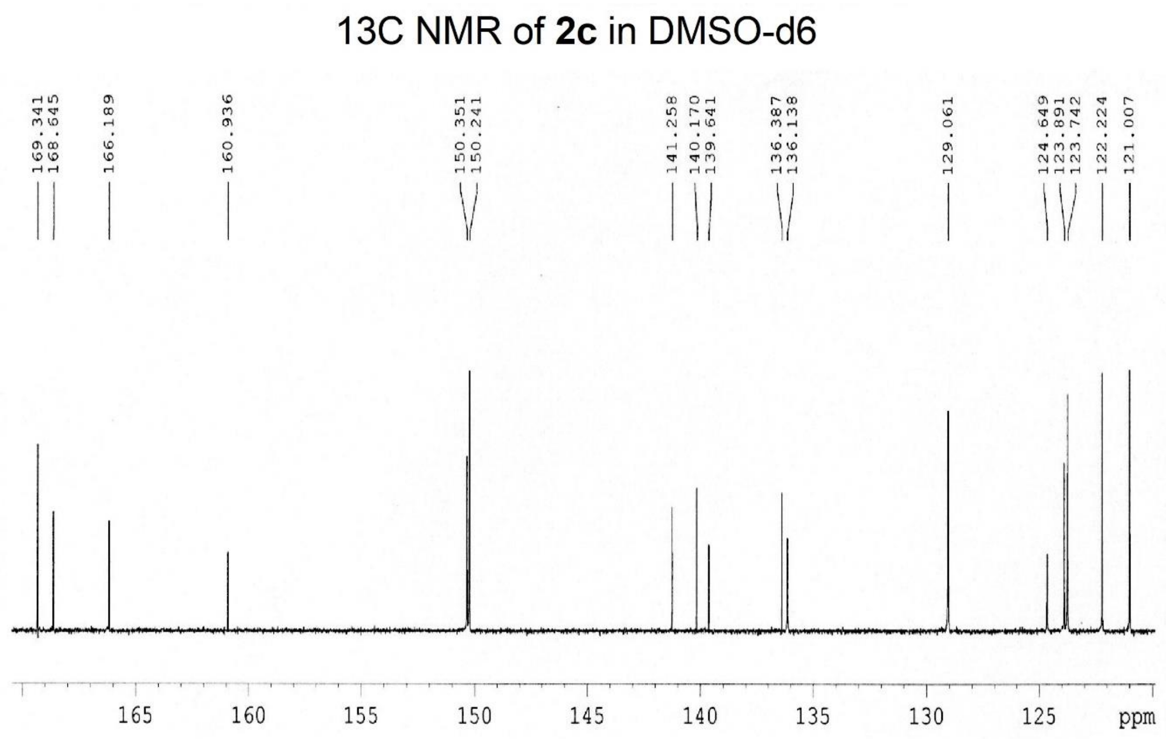

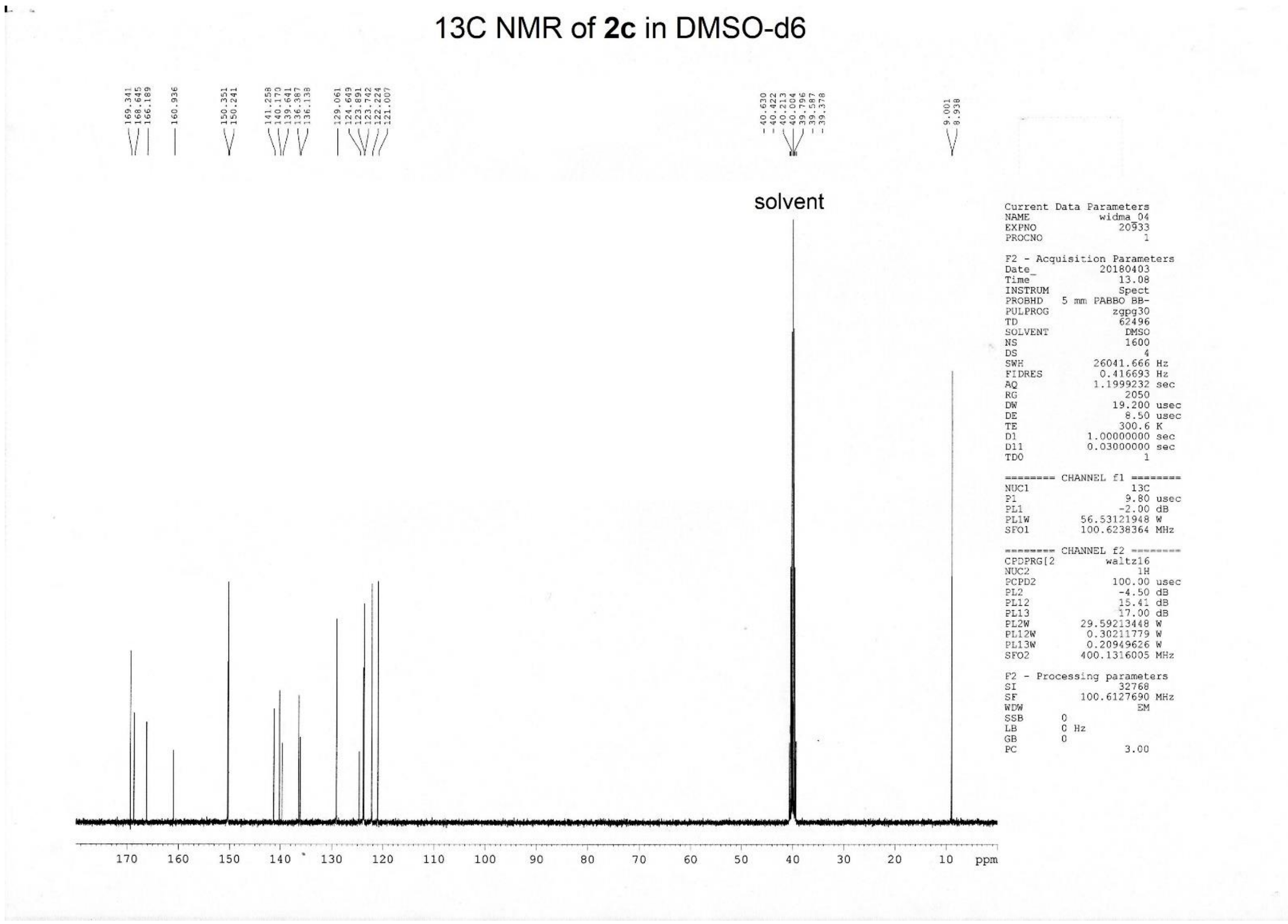

Figure S34. ^{13}C NMR spectrum of **2c** (in DMSO-d_6), with enlarged fragment at the top

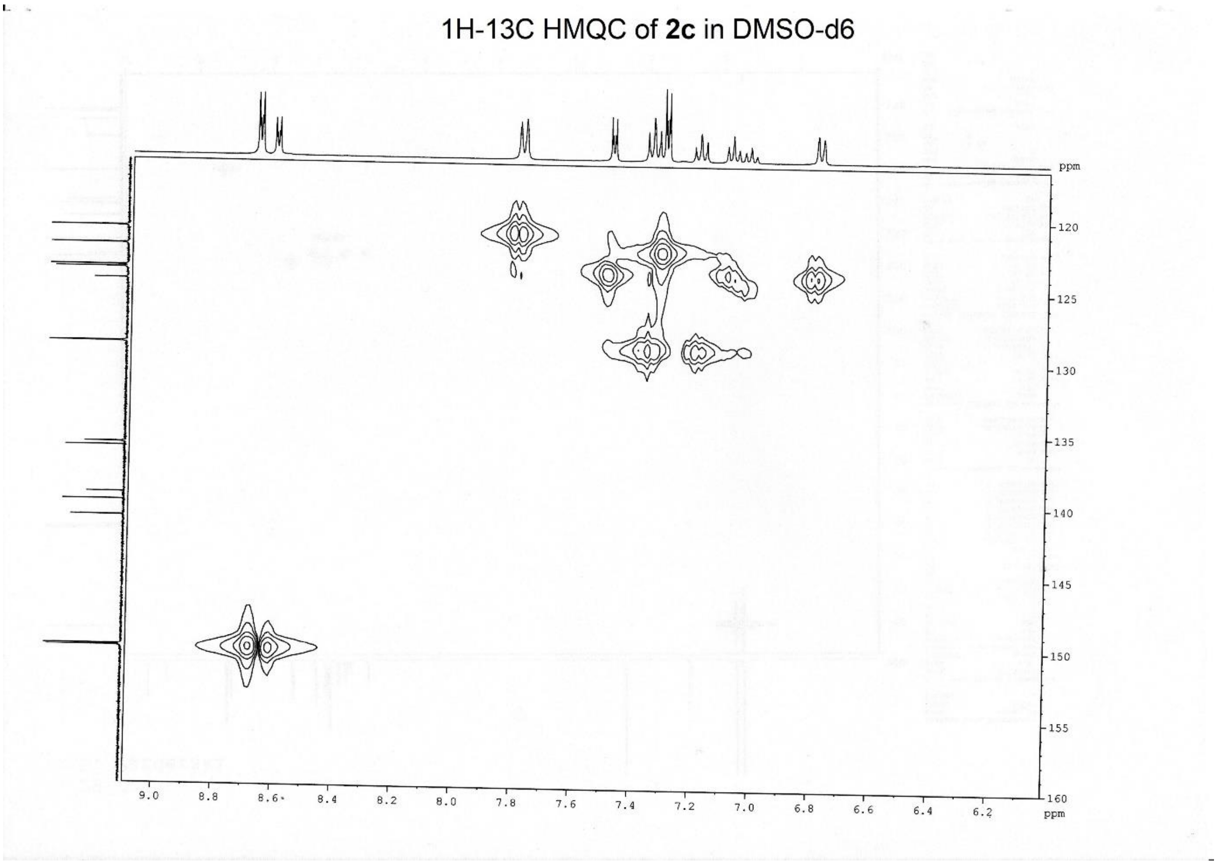

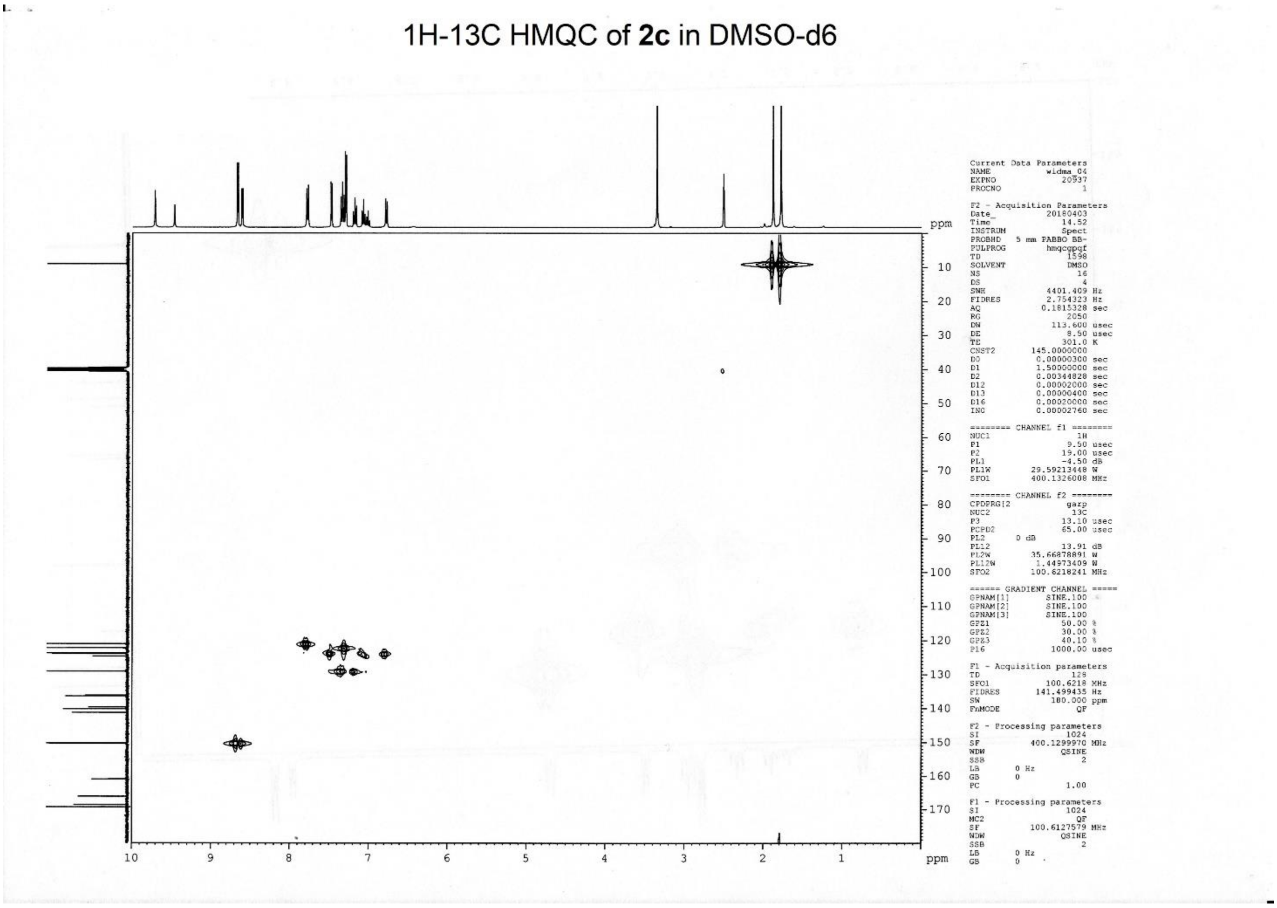

Figure S35. ^{1}H-^{13}C HMQC spectrum of **2c** (in DMSO-d_6), with enlarged fragment at the top

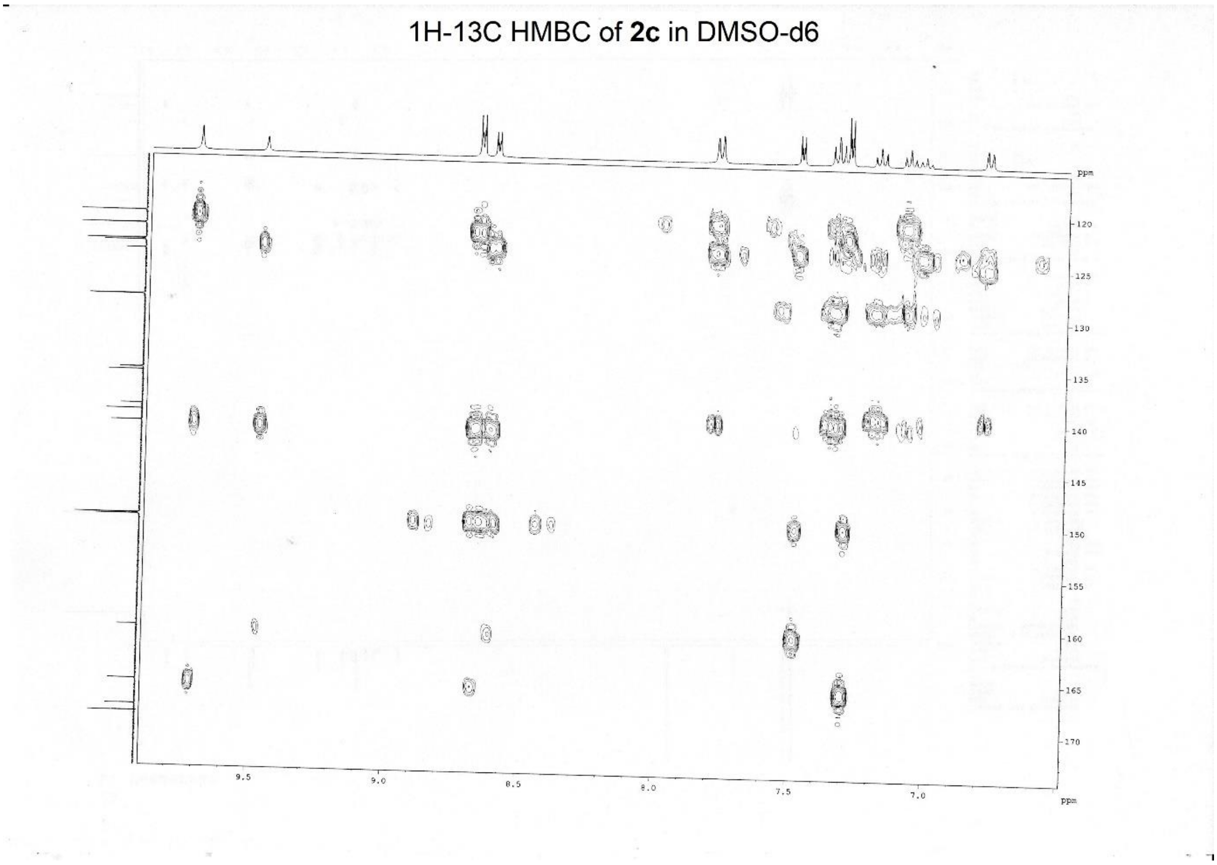

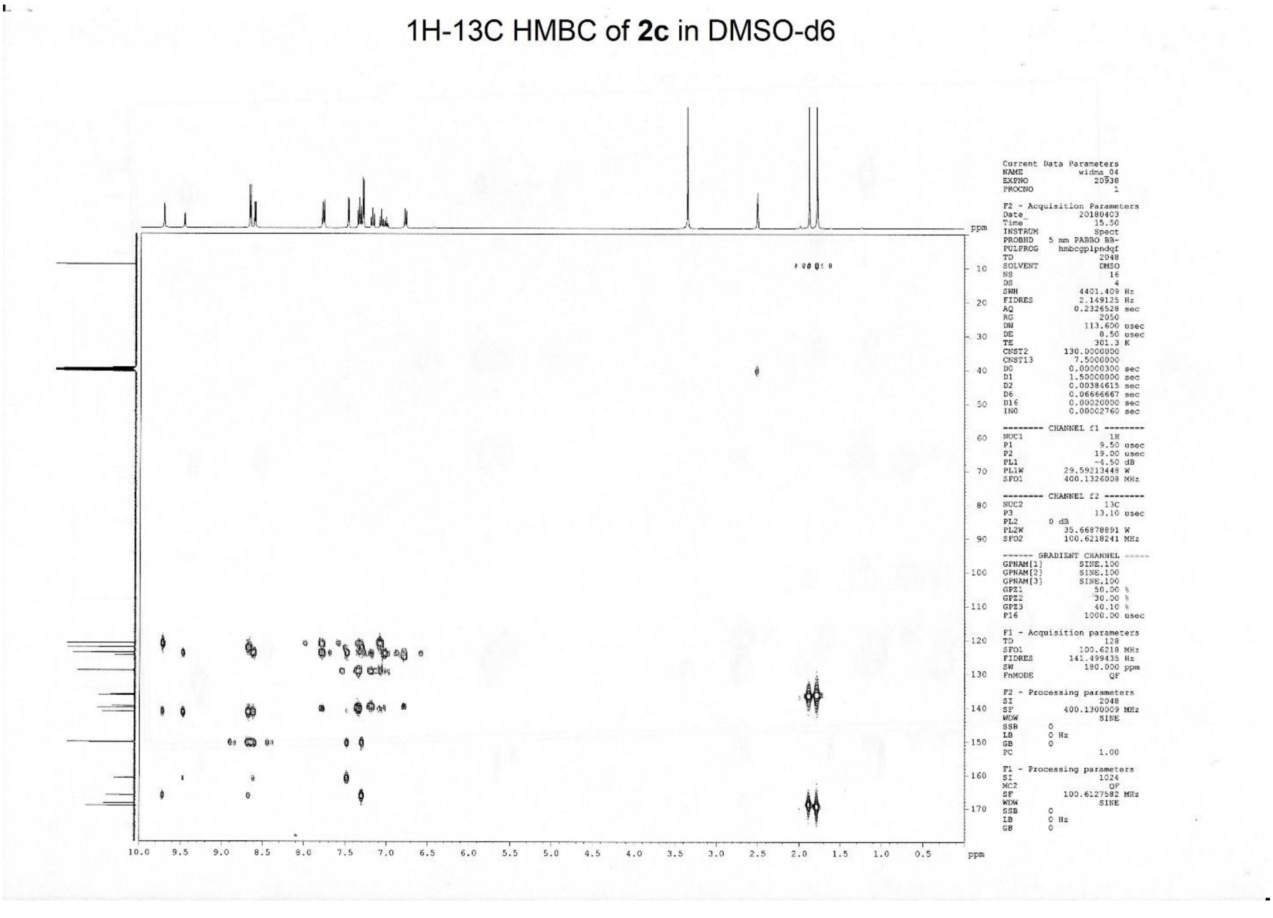

Figure S36. ^{1}H-^{13}C HMBC spectrum of **2c** (in DMSO-d_6), with enlarged fragment at the top

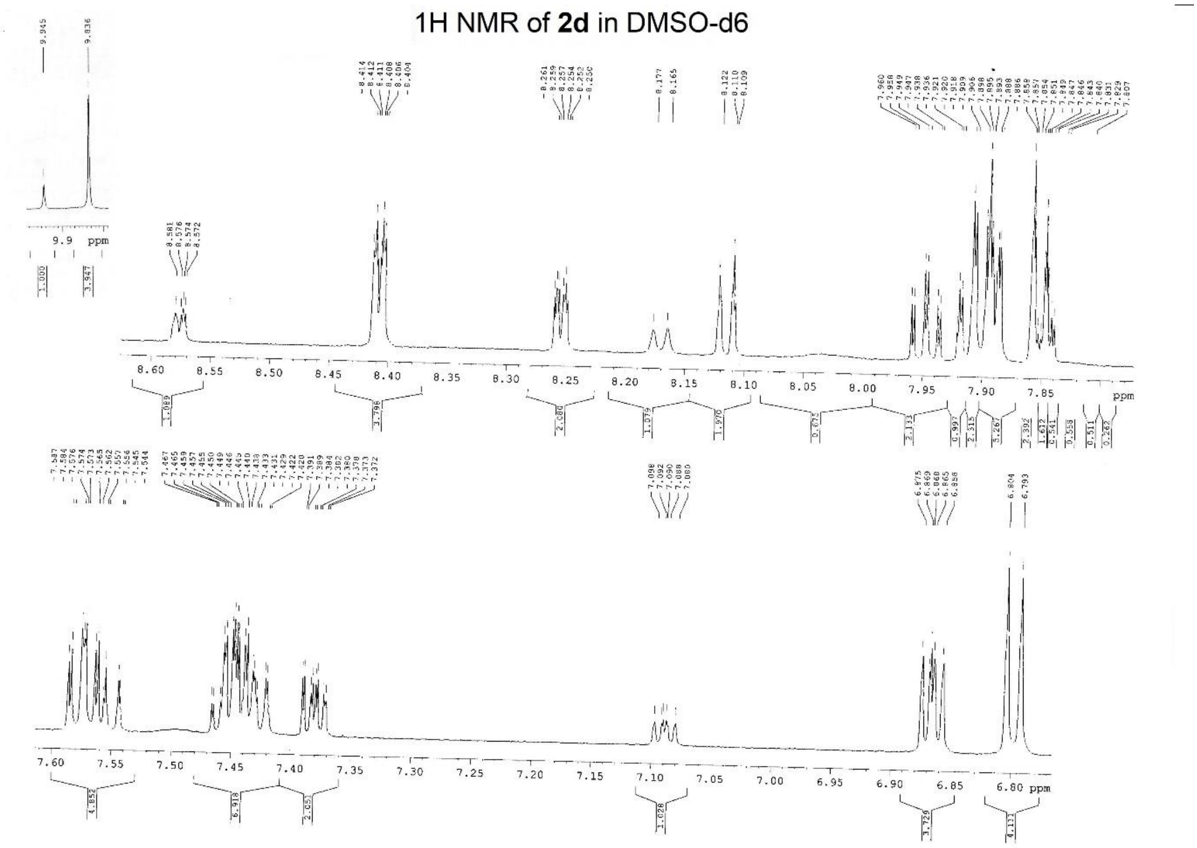

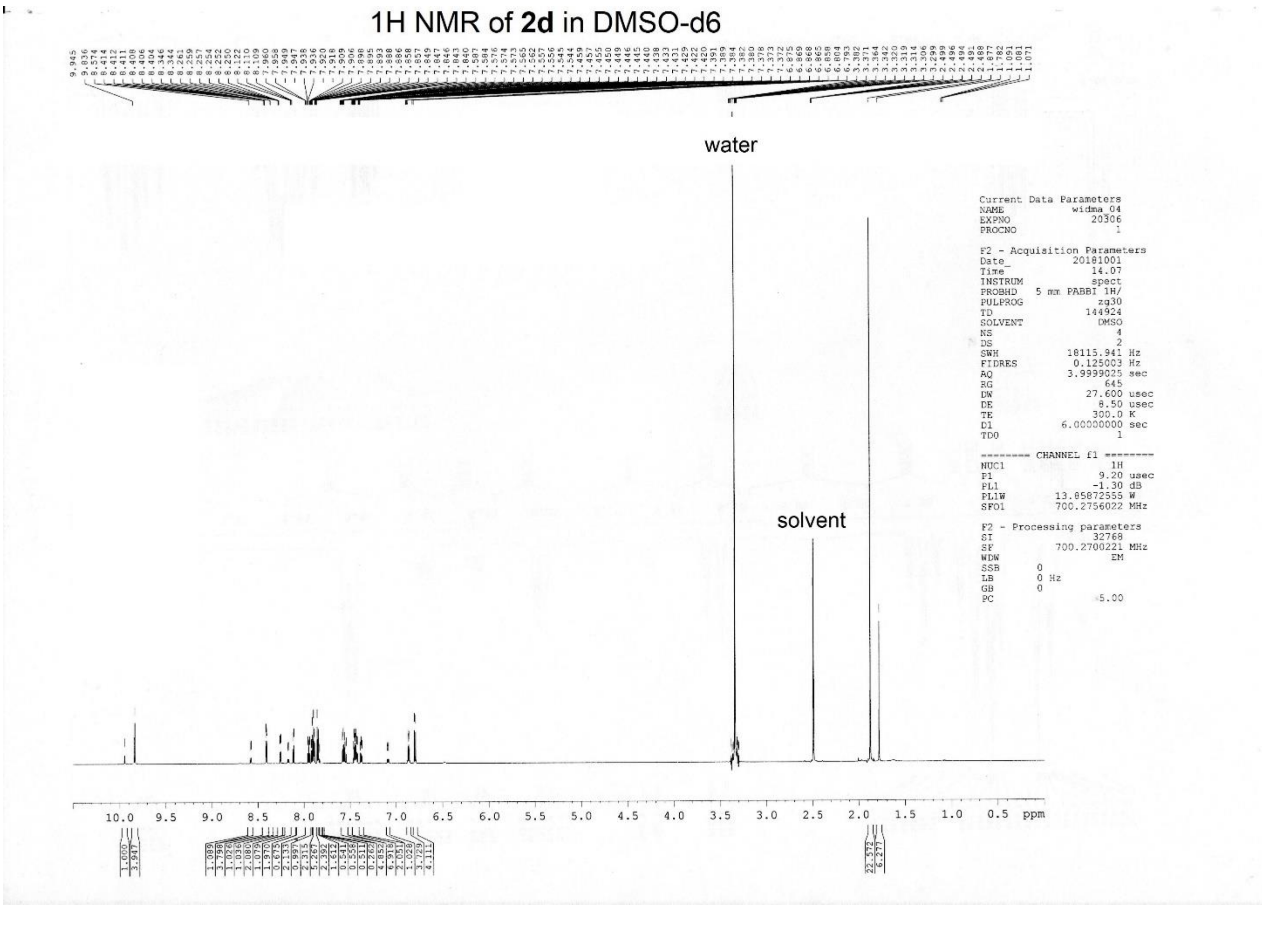

Figure S37. ^{1}H NMR spectrum of **2d** (in DMSO-d_6), with enlarged fragment at the top

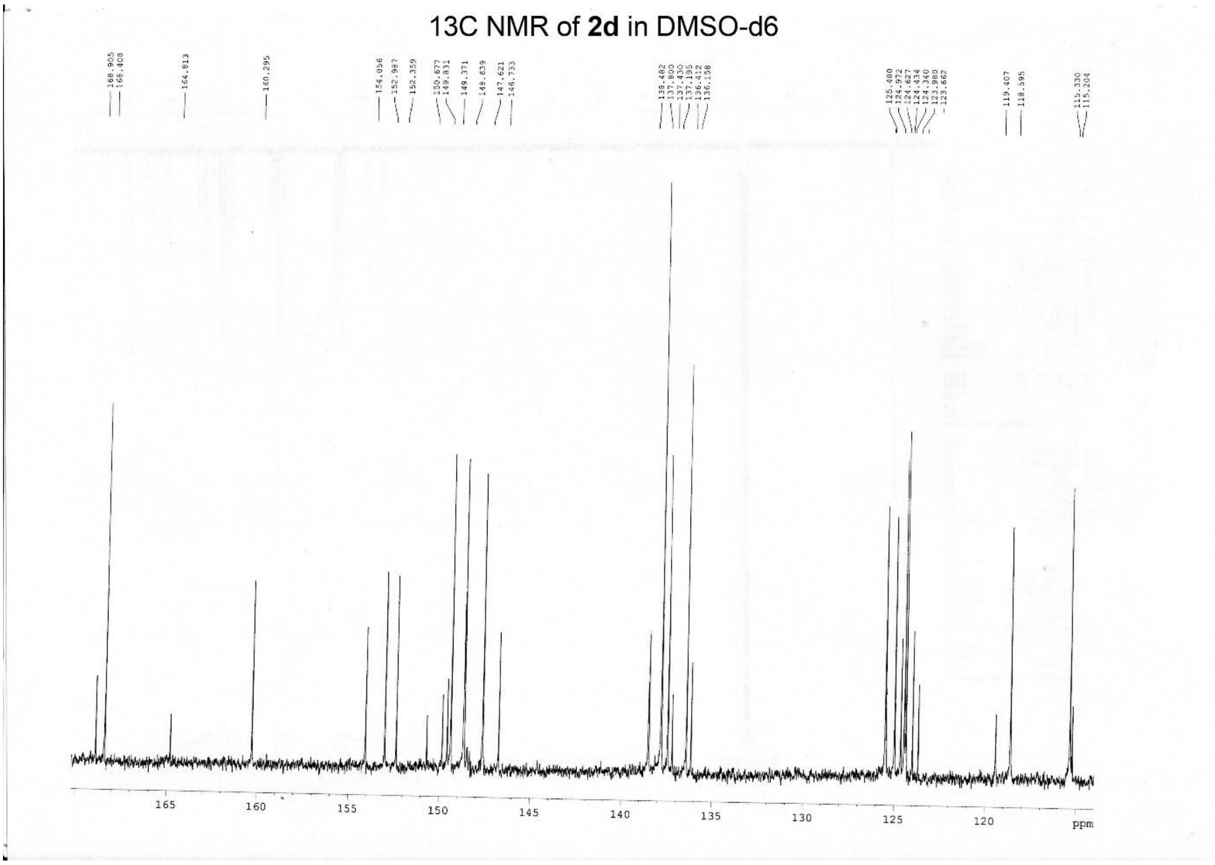

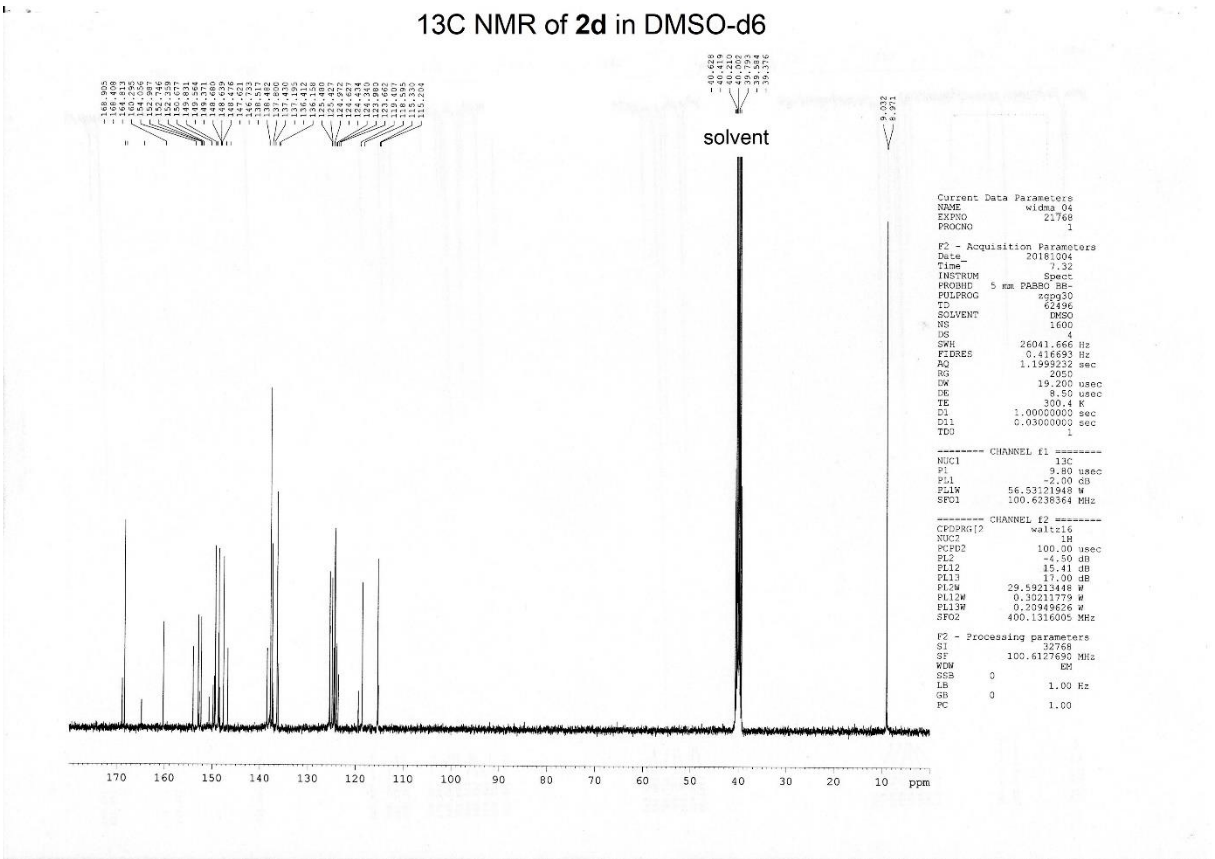

Figure S38. ^{13}C NMR spectrum of **2d** (in DMSO-d_6), with enlarged fragment at the top

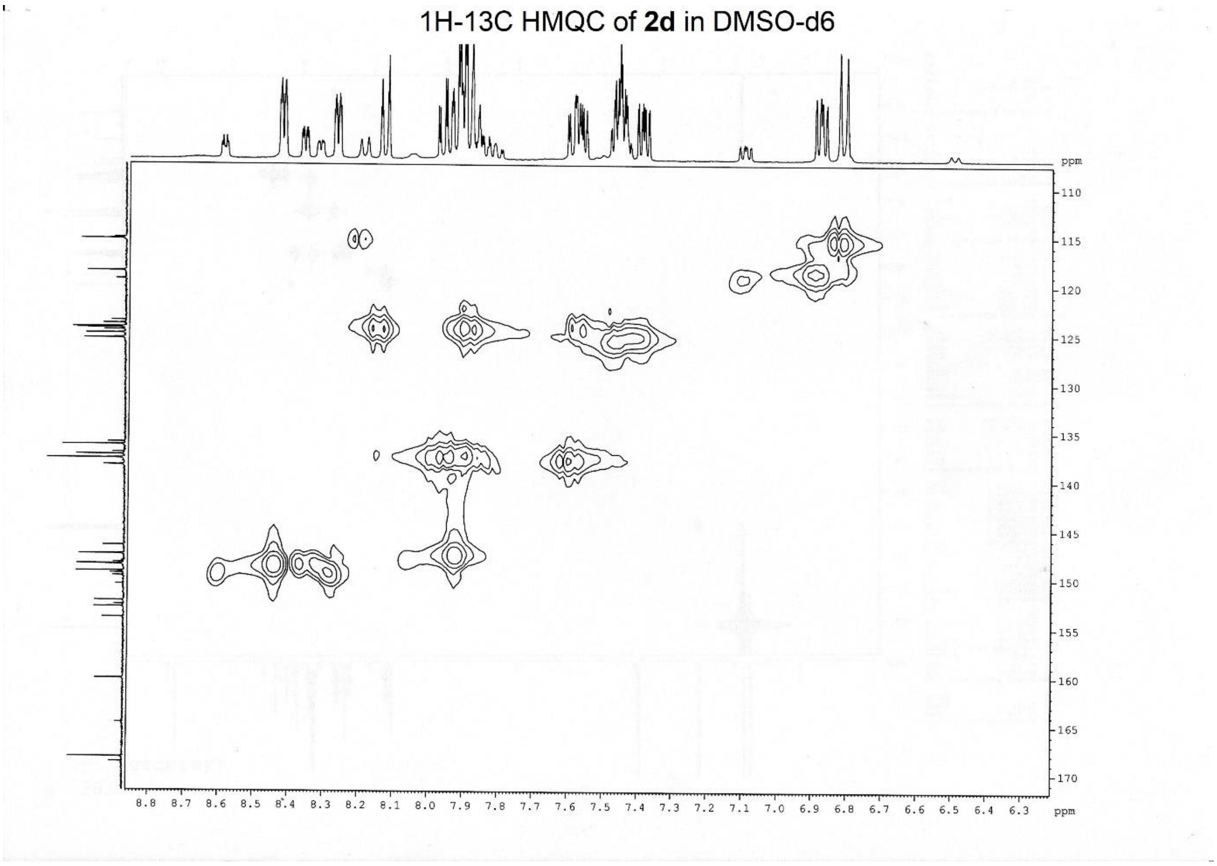

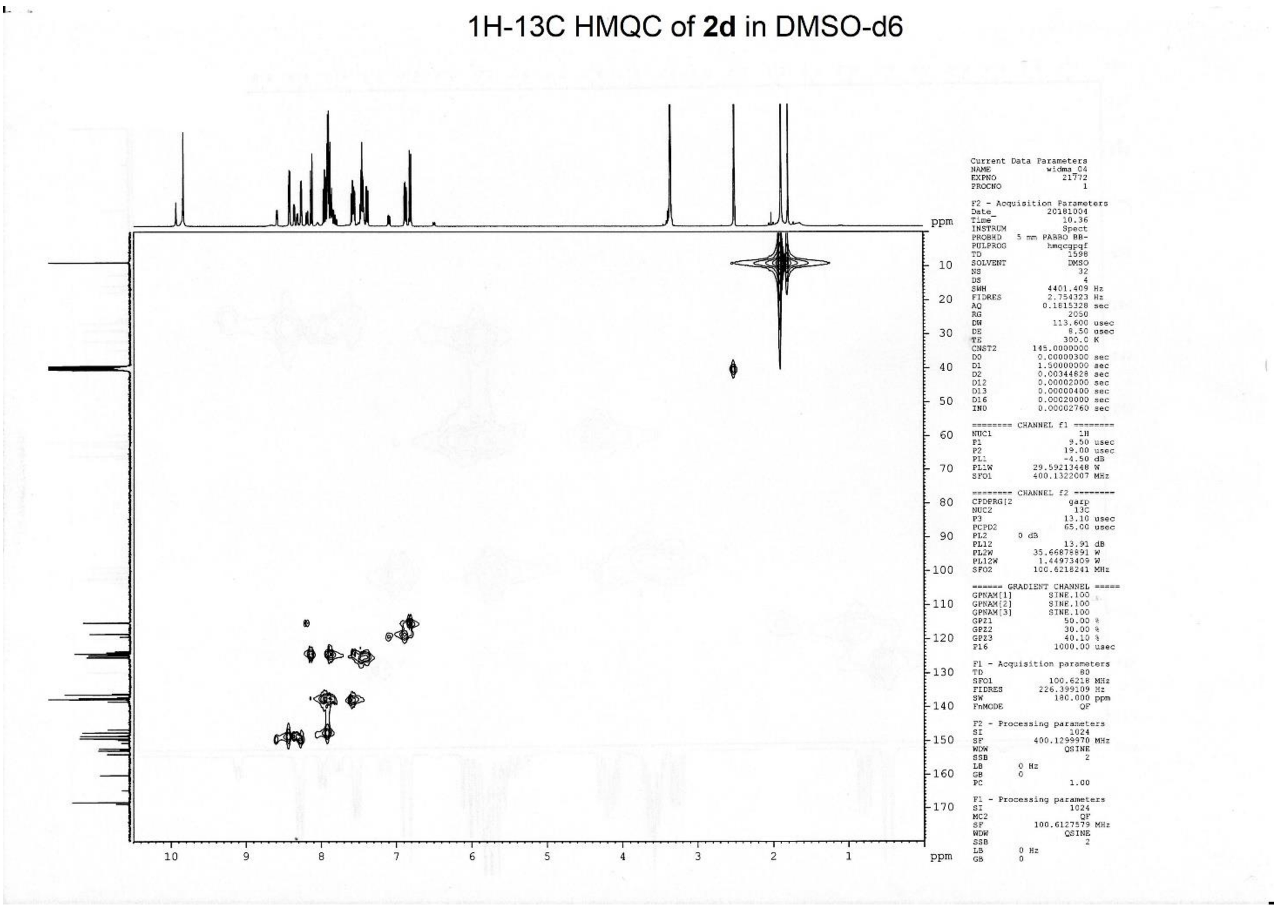

Figure S39. ^{1}H-^{13}C HMQC spectrum of **2d** (in DMSO-d_6), with enlarged fragment at the top

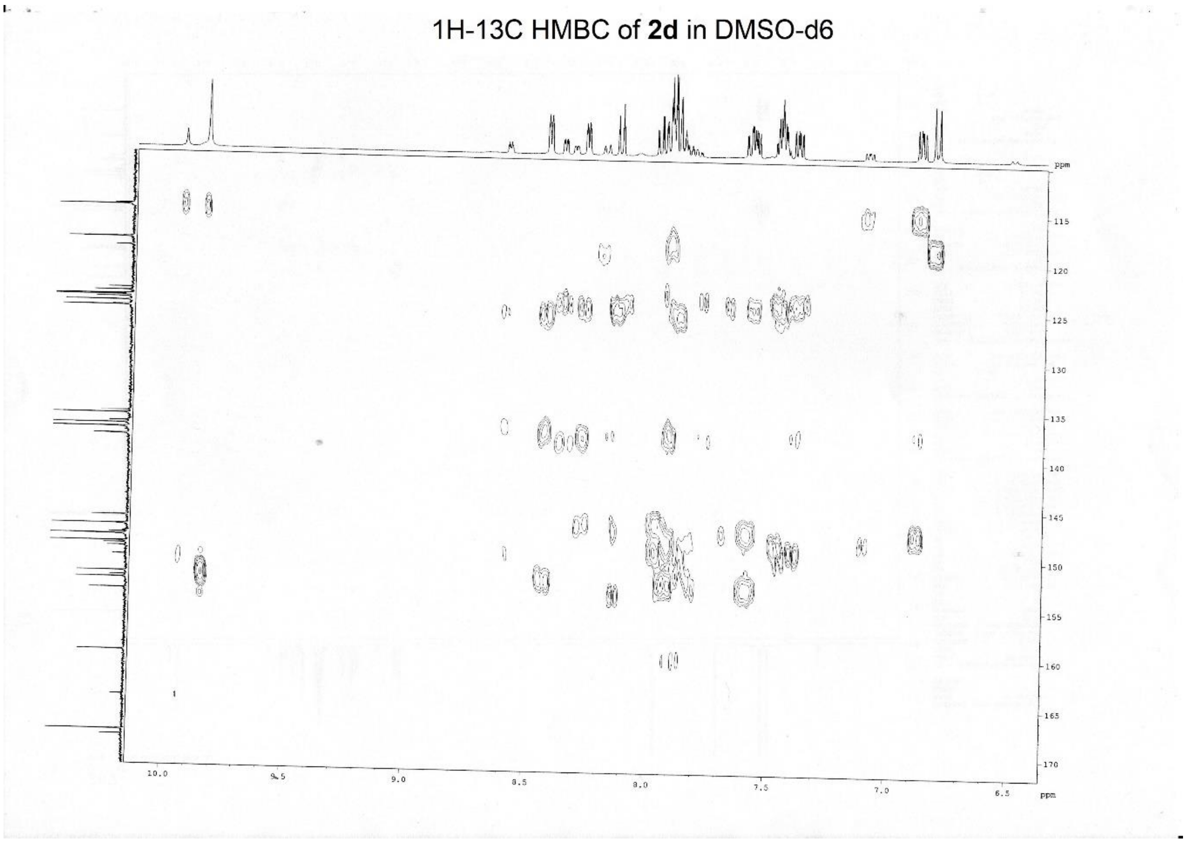

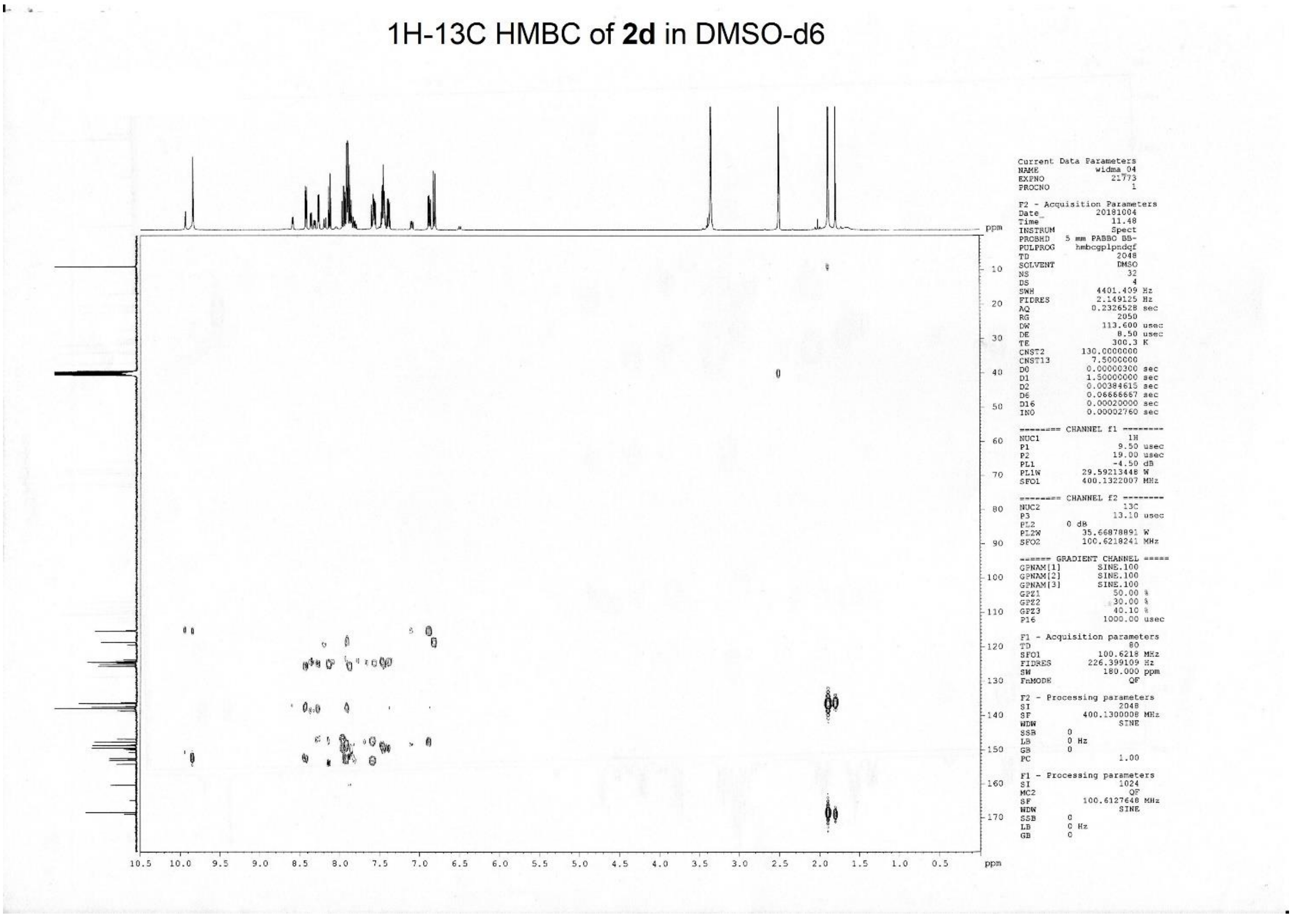

Figure S40. ^{1}H-^{13}C HMBC spectrum of **2d** (in DMSO-d_6), with enlarged fragment at the top

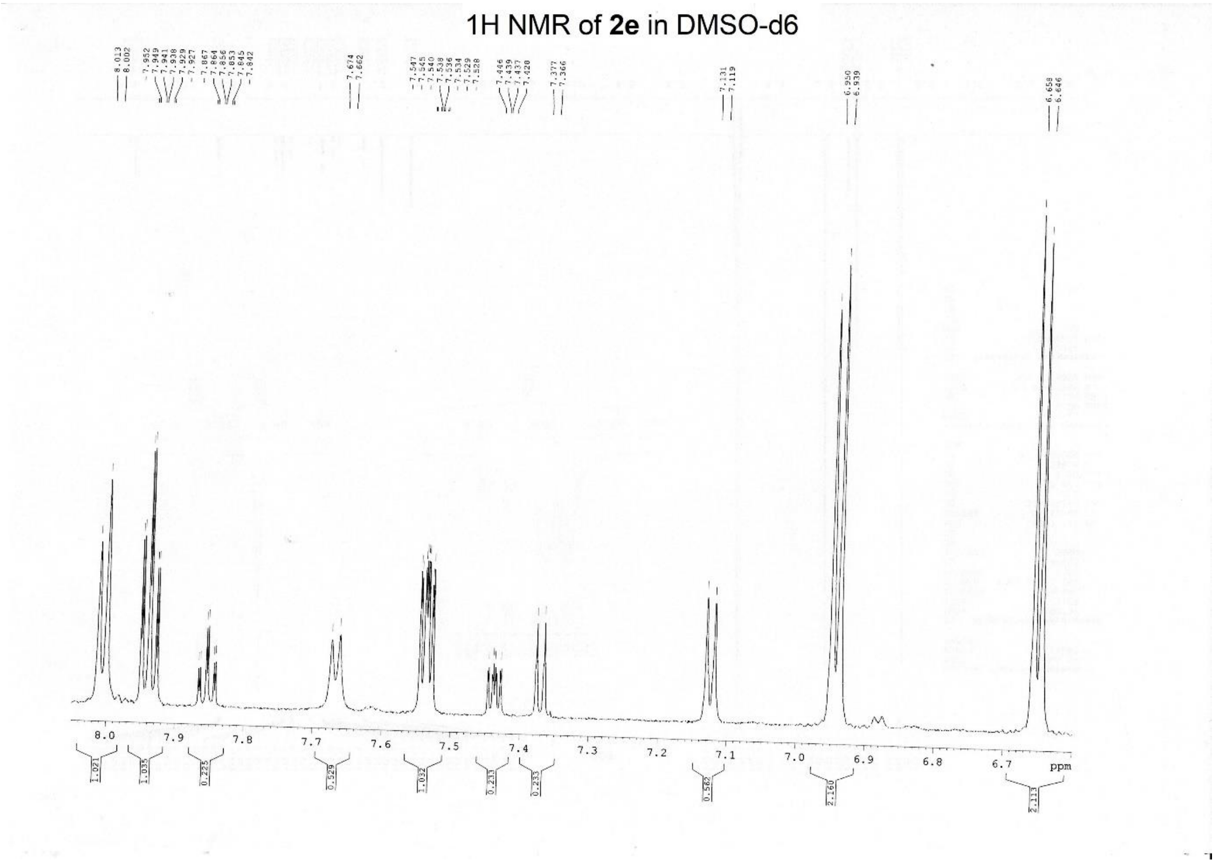

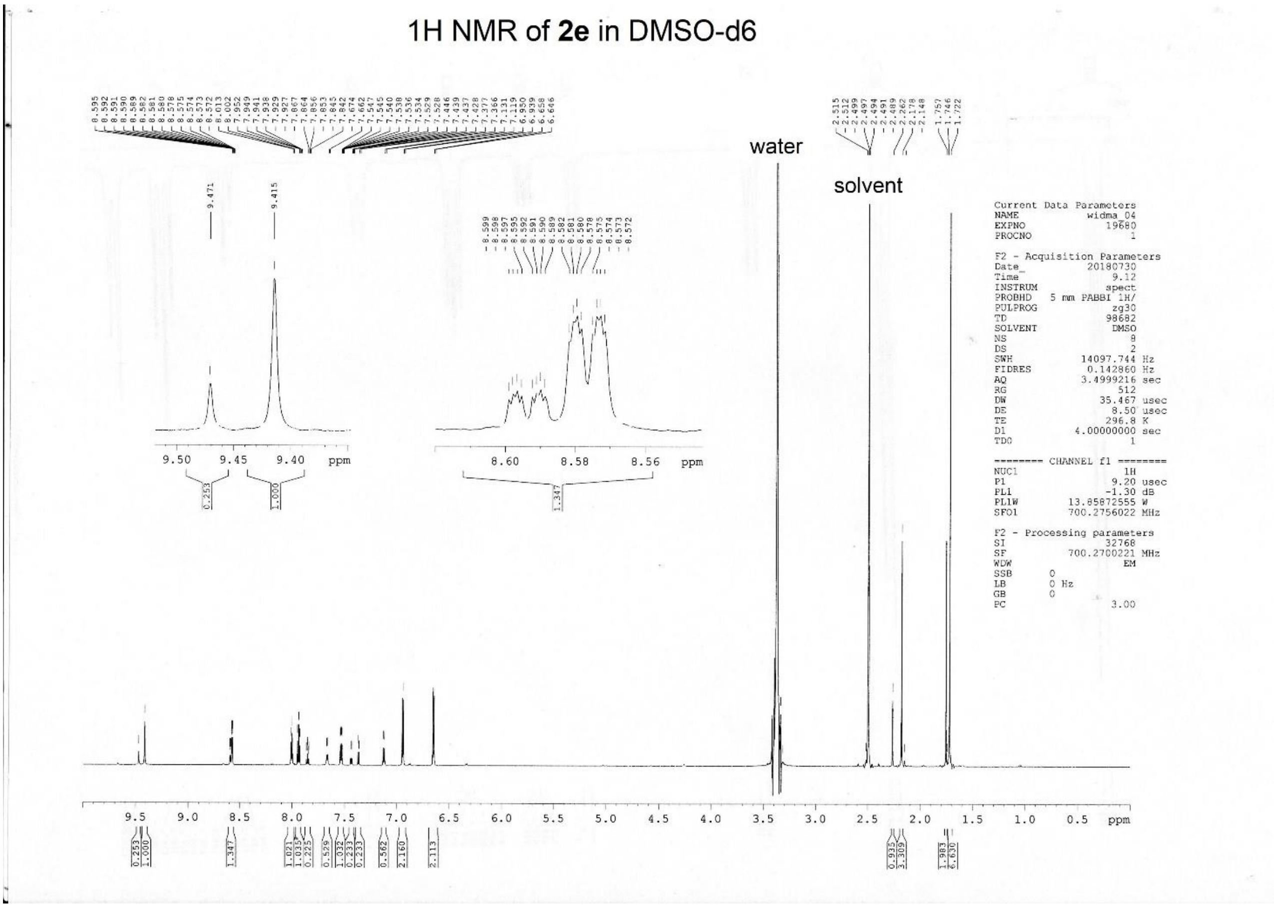

Figure S41. ^{1}H NMR spectrum of **2e** (in DMSO-d_6), with enlarged fragment at the top

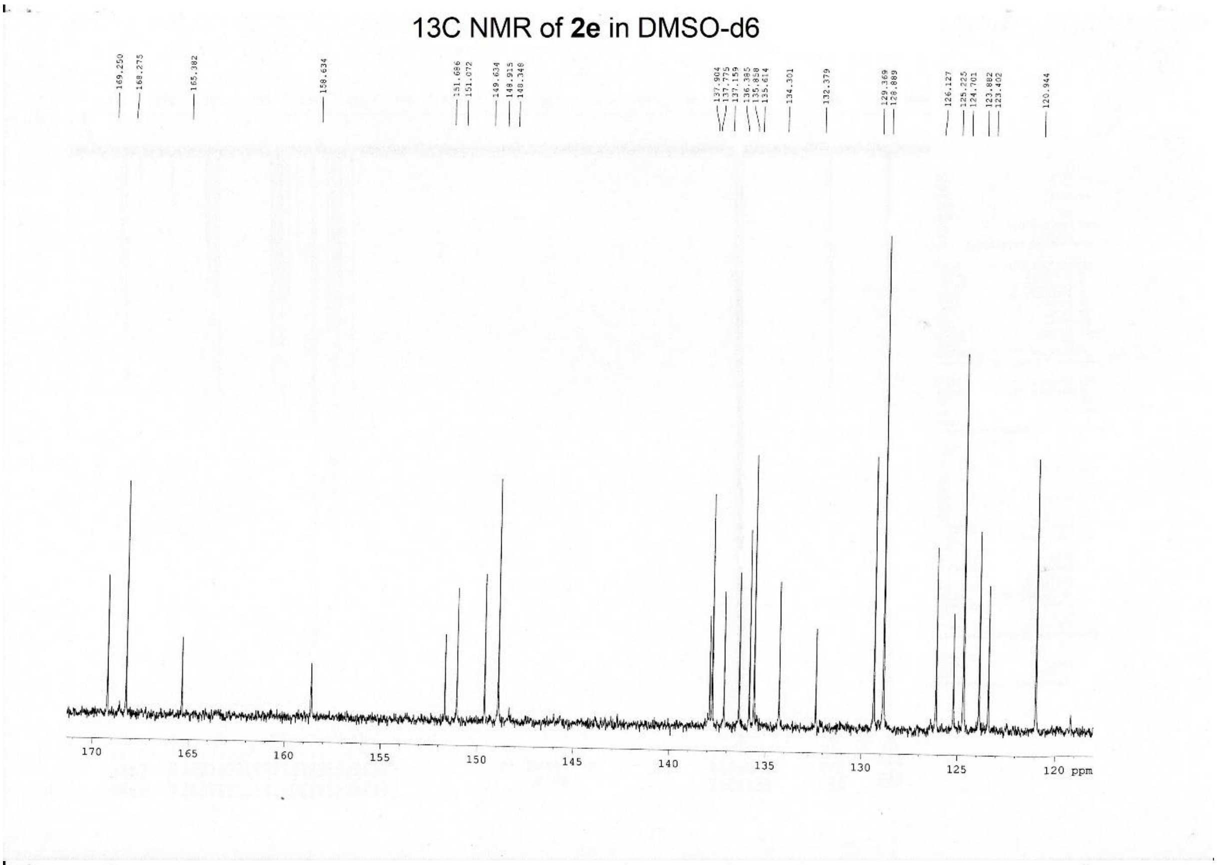

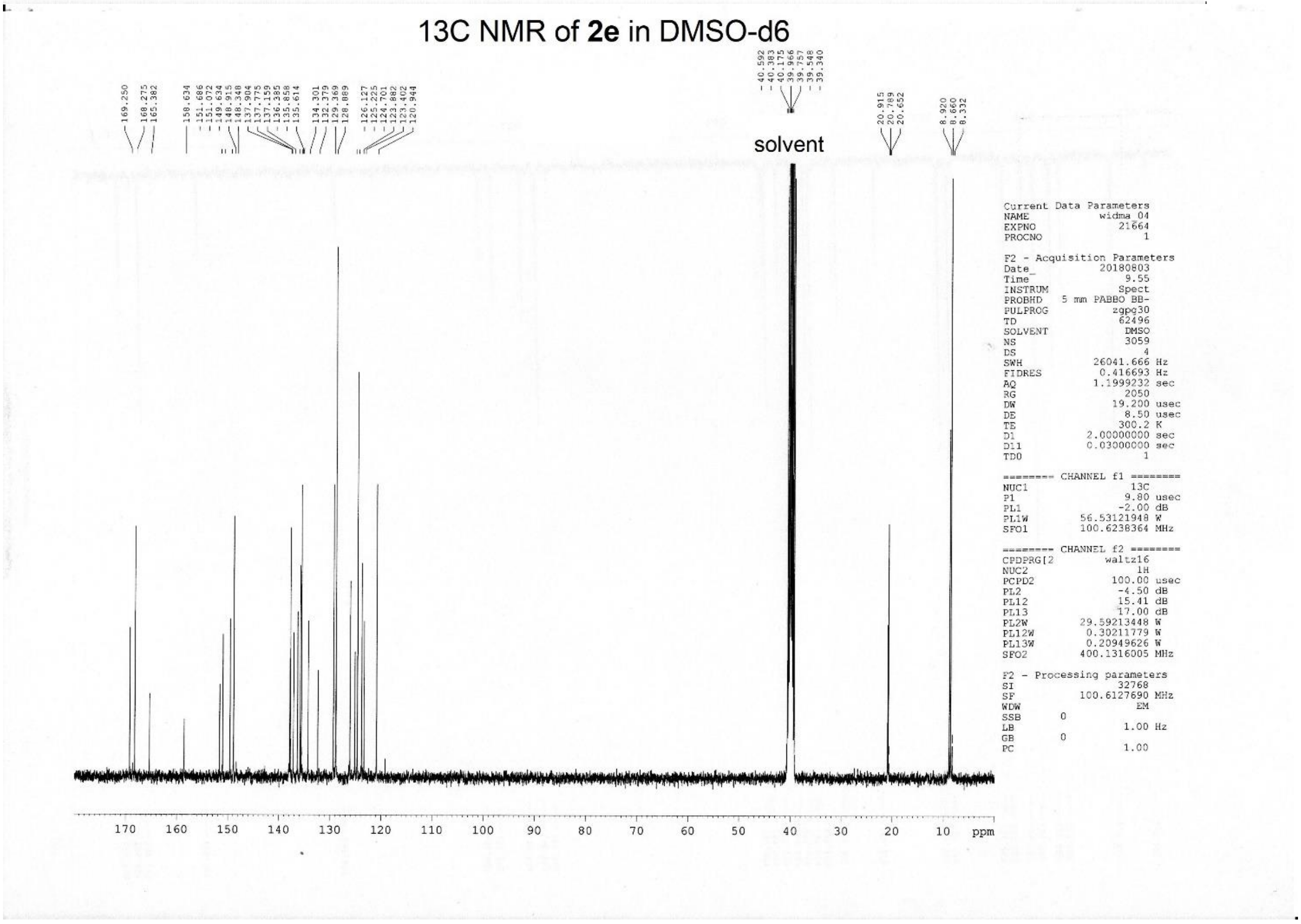

Figure S42. ^{13}C NMR spectrum of **2e** (in DMSO-d_6), with enlarged fragment at the top

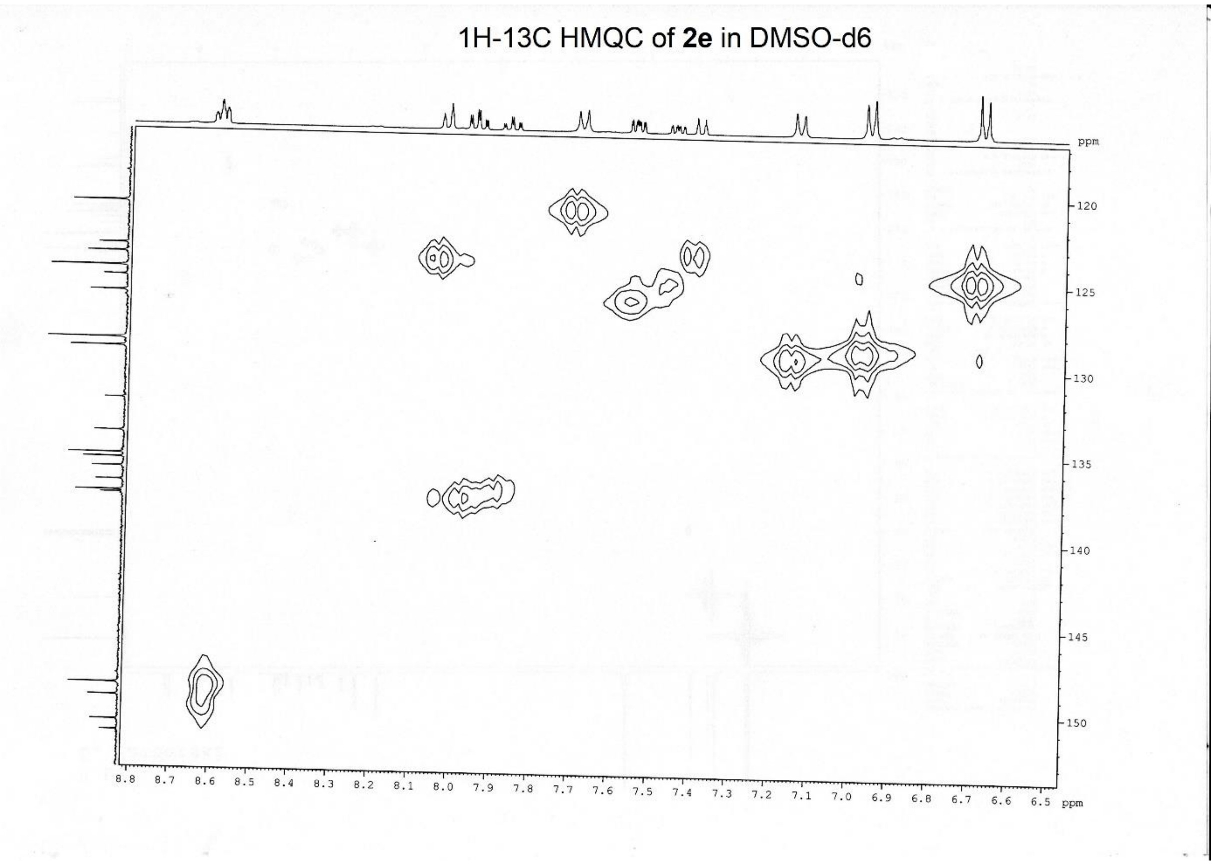

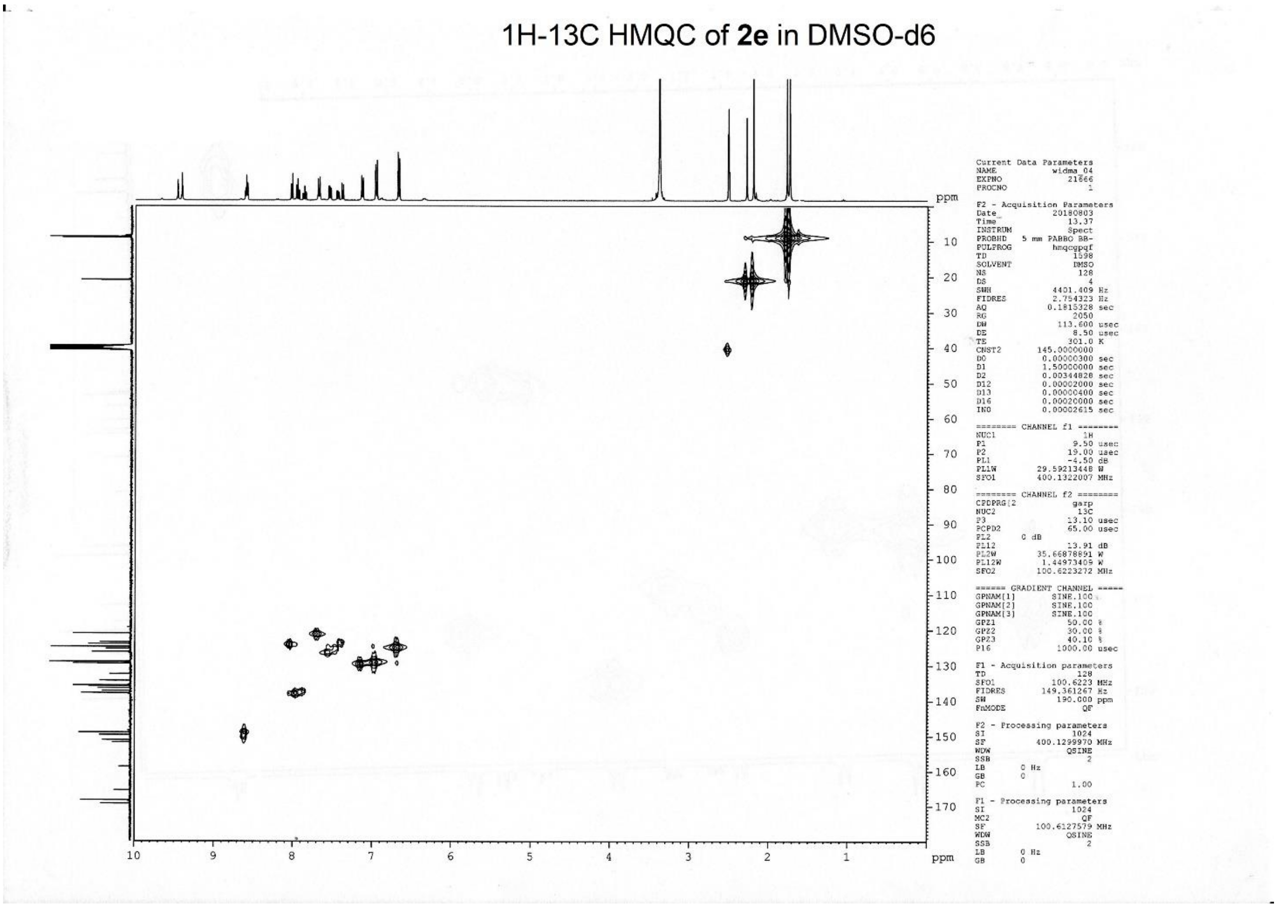

Figure S43. ^{1}H-^{13}C HMQC spectrum of **2e** (in DMSO-d_6), with enlarged fragment at the top

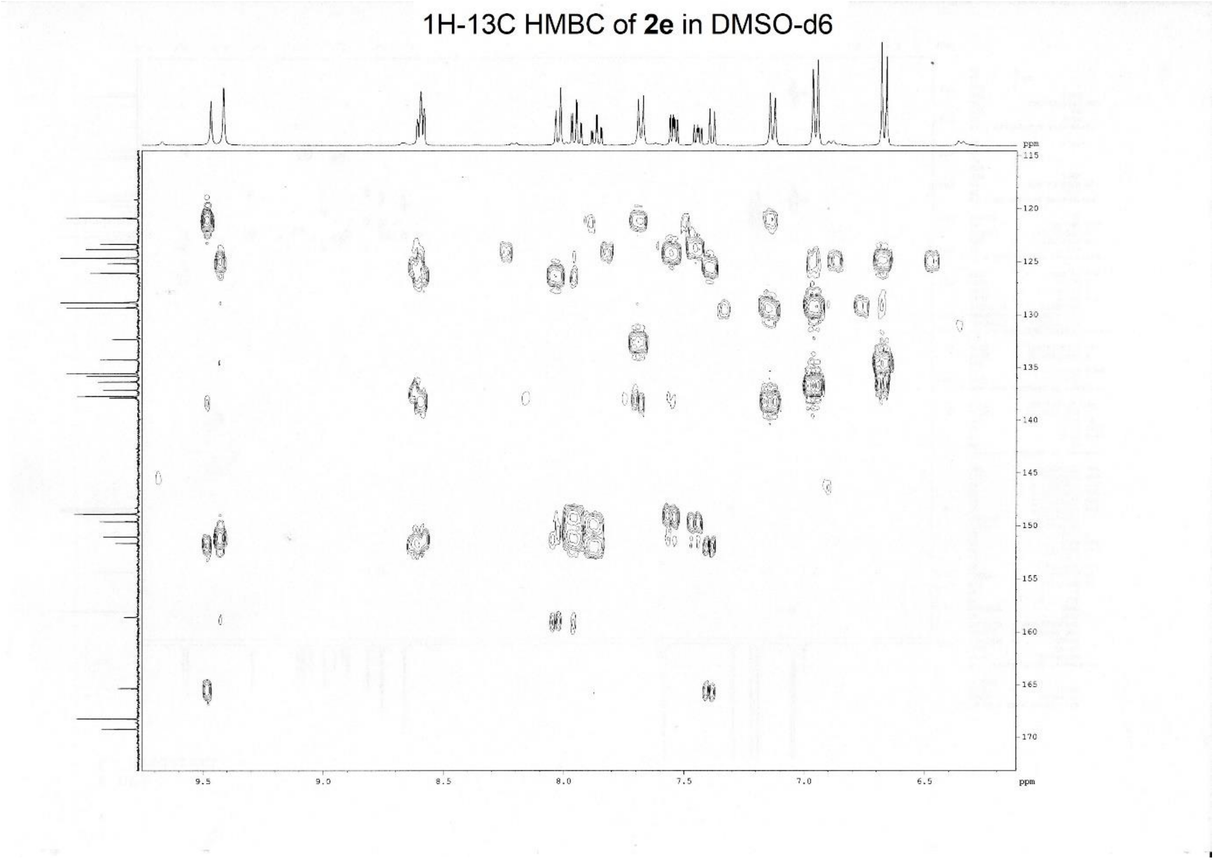

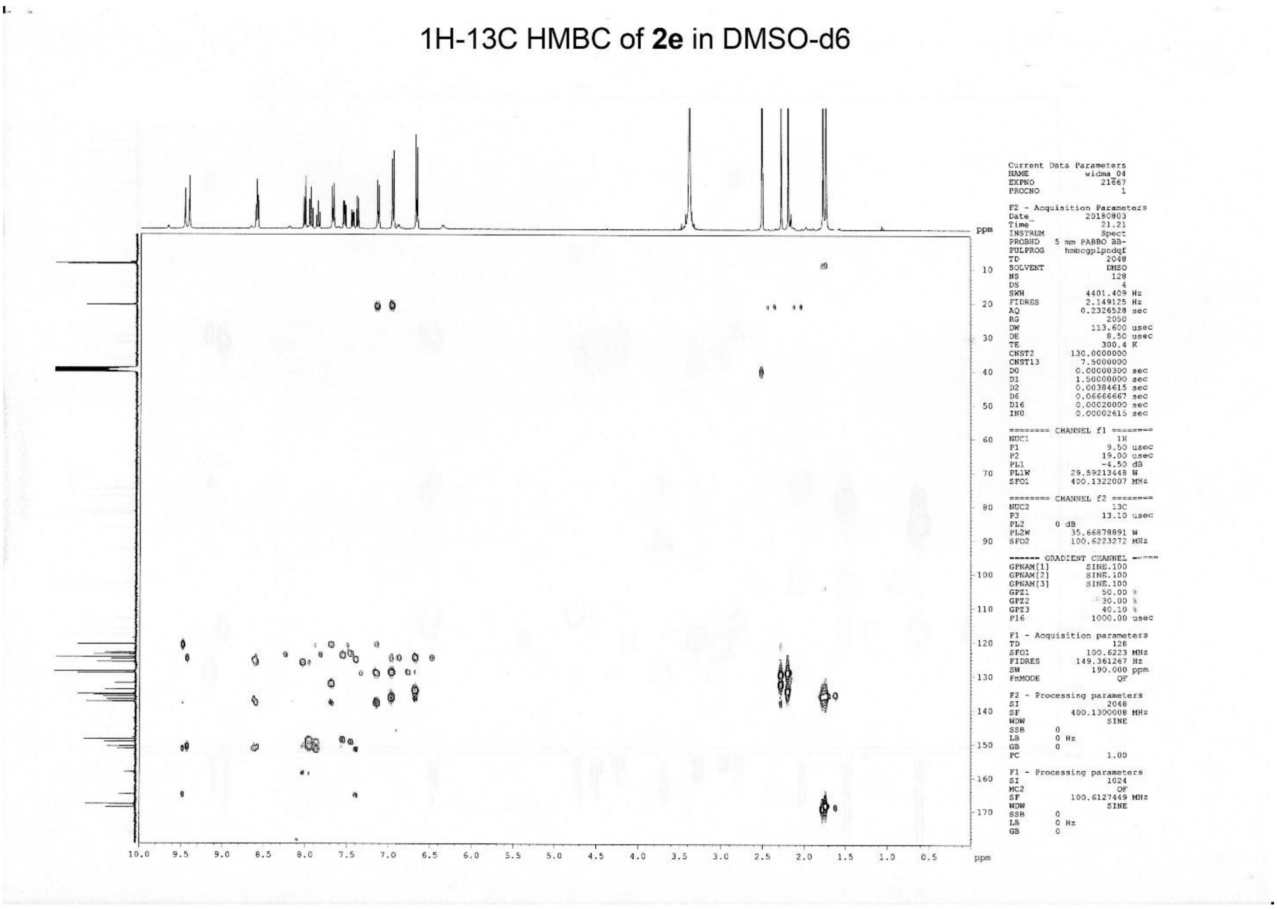

Figure S44. ^{1}H-^{13}C HMBC spectrum of **2e** (in DMSO-d_6), with enlarged fragment at the top

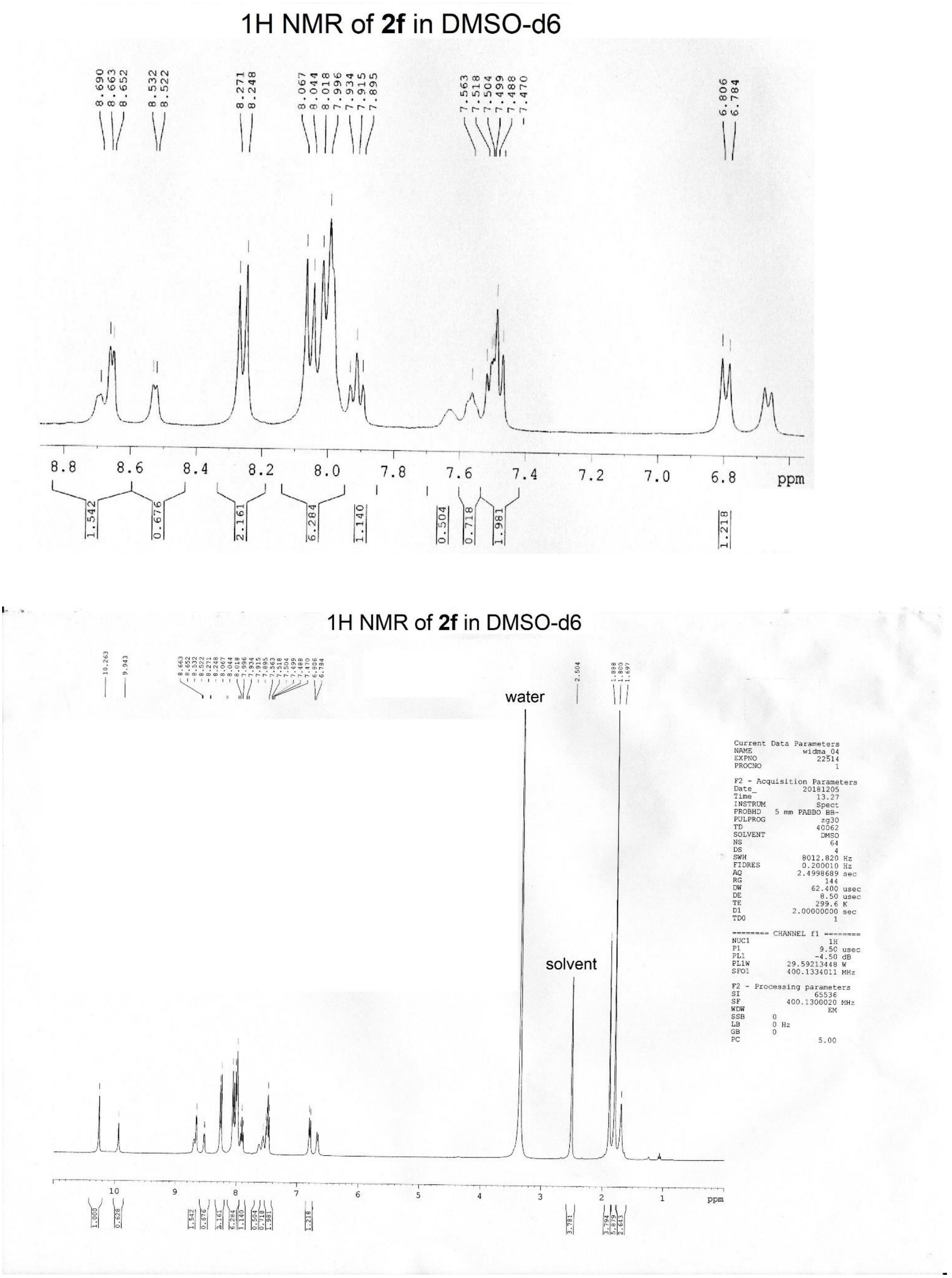

Figure S45. ^{1}H NMR spectrum of **2f** (in DMSO-d_6), with enlarged fragment at the top

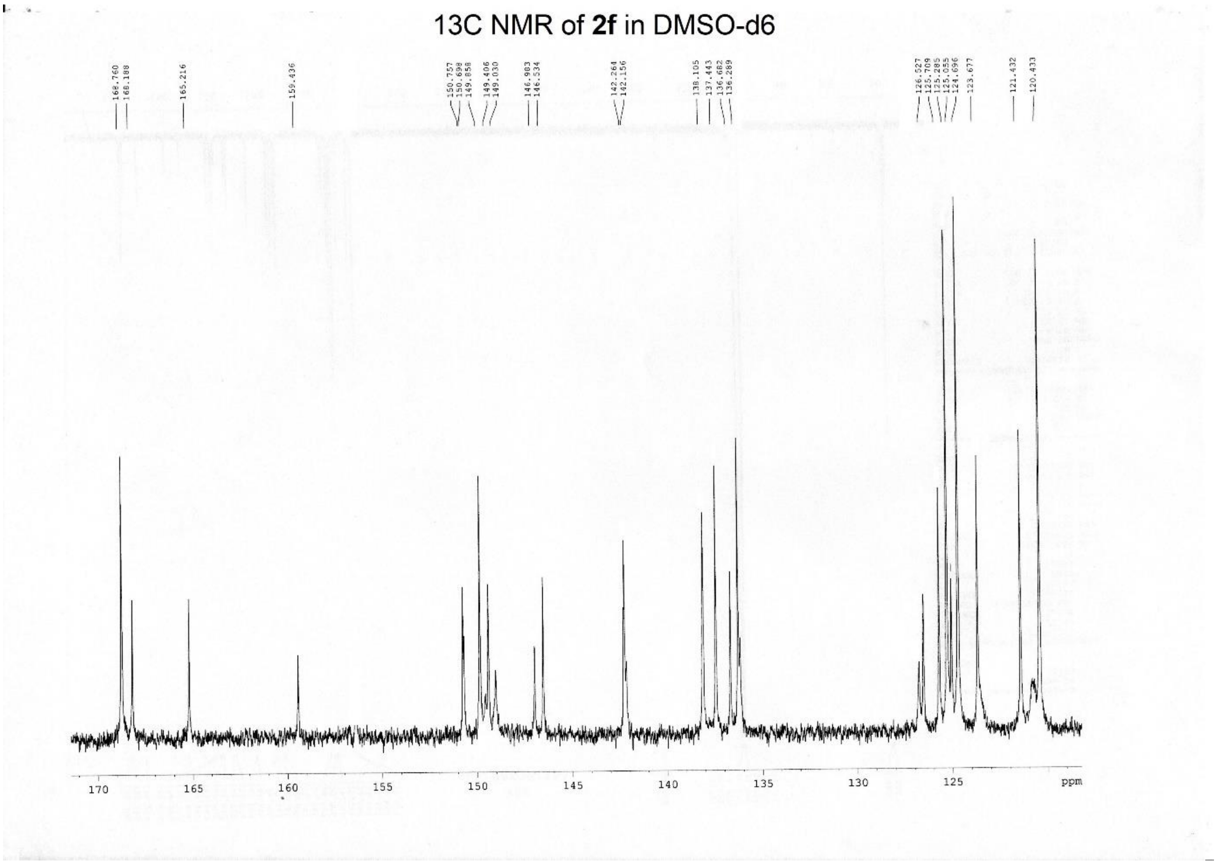

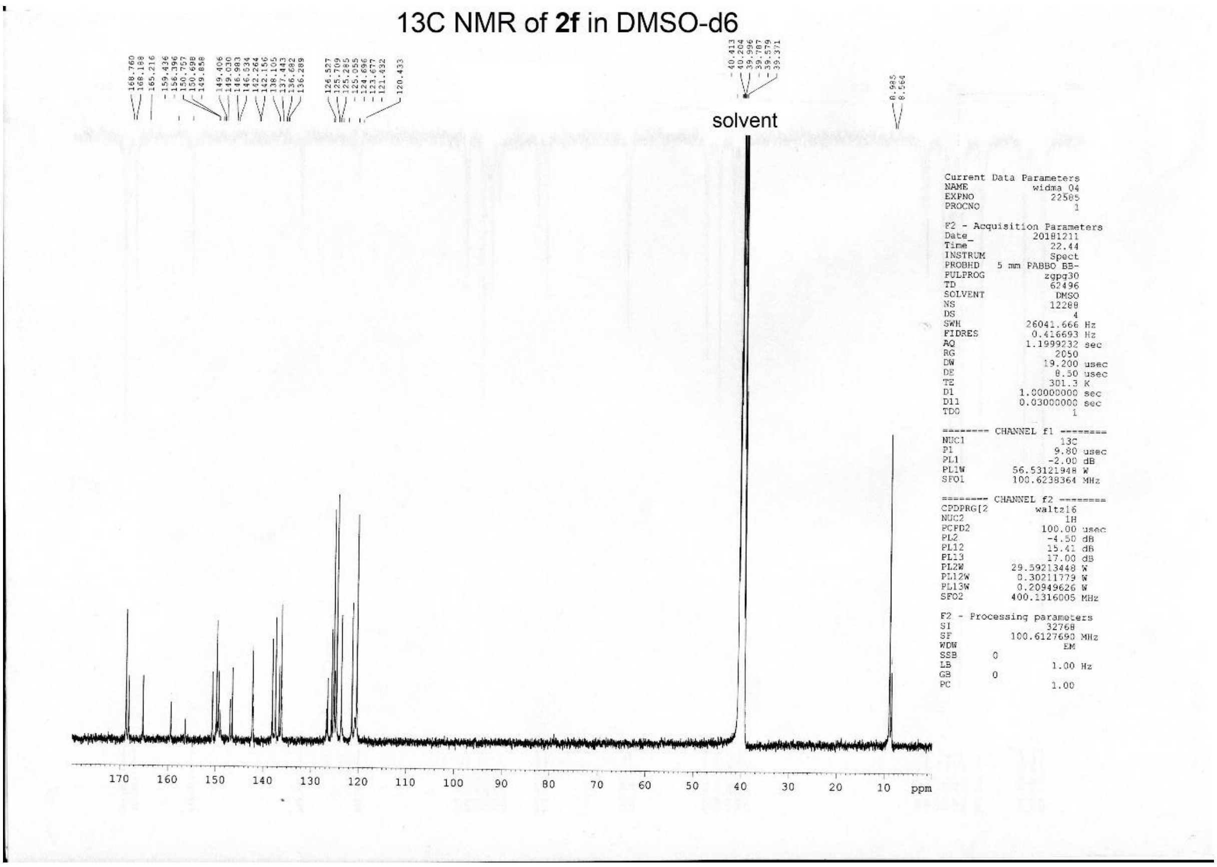

Figure S46. ^{13}C NMR spectrum of **2f** (in DMSO-d$_6$), with enlarged fragment at the top

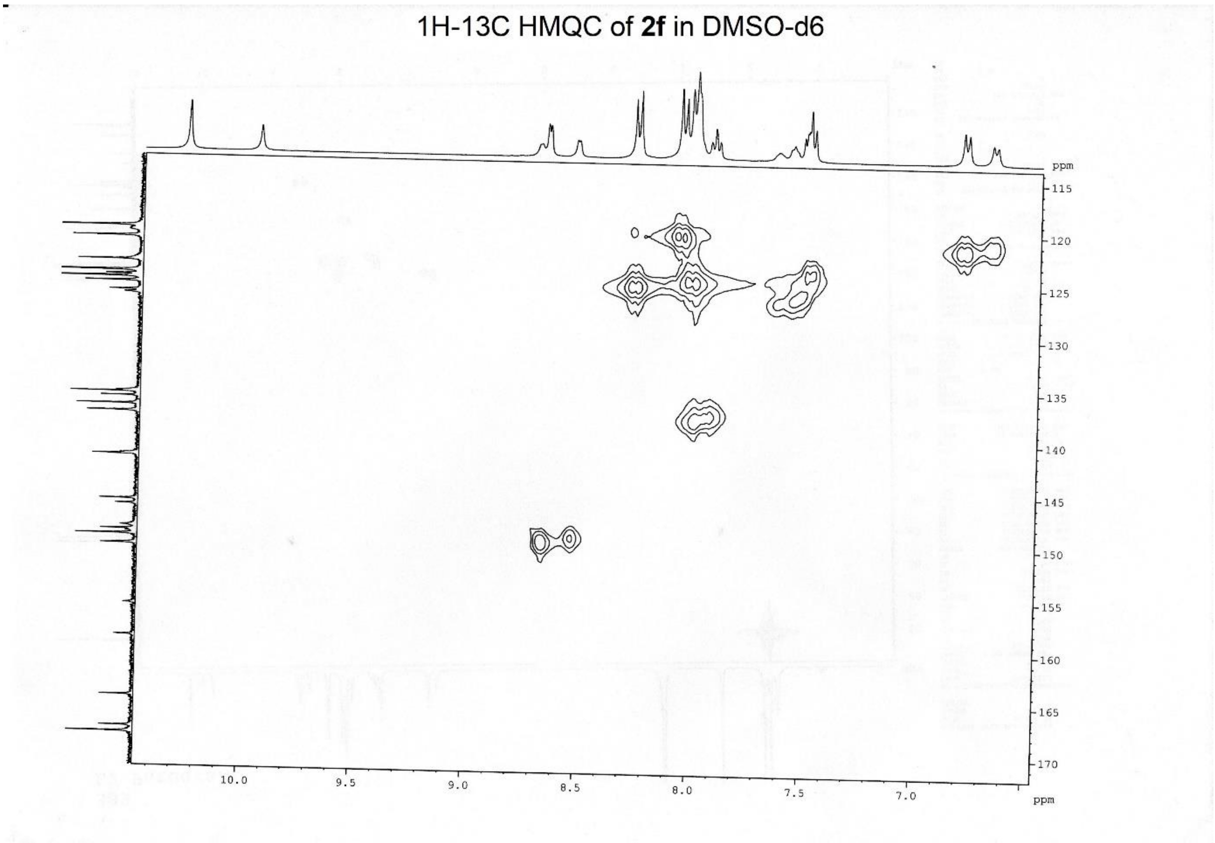

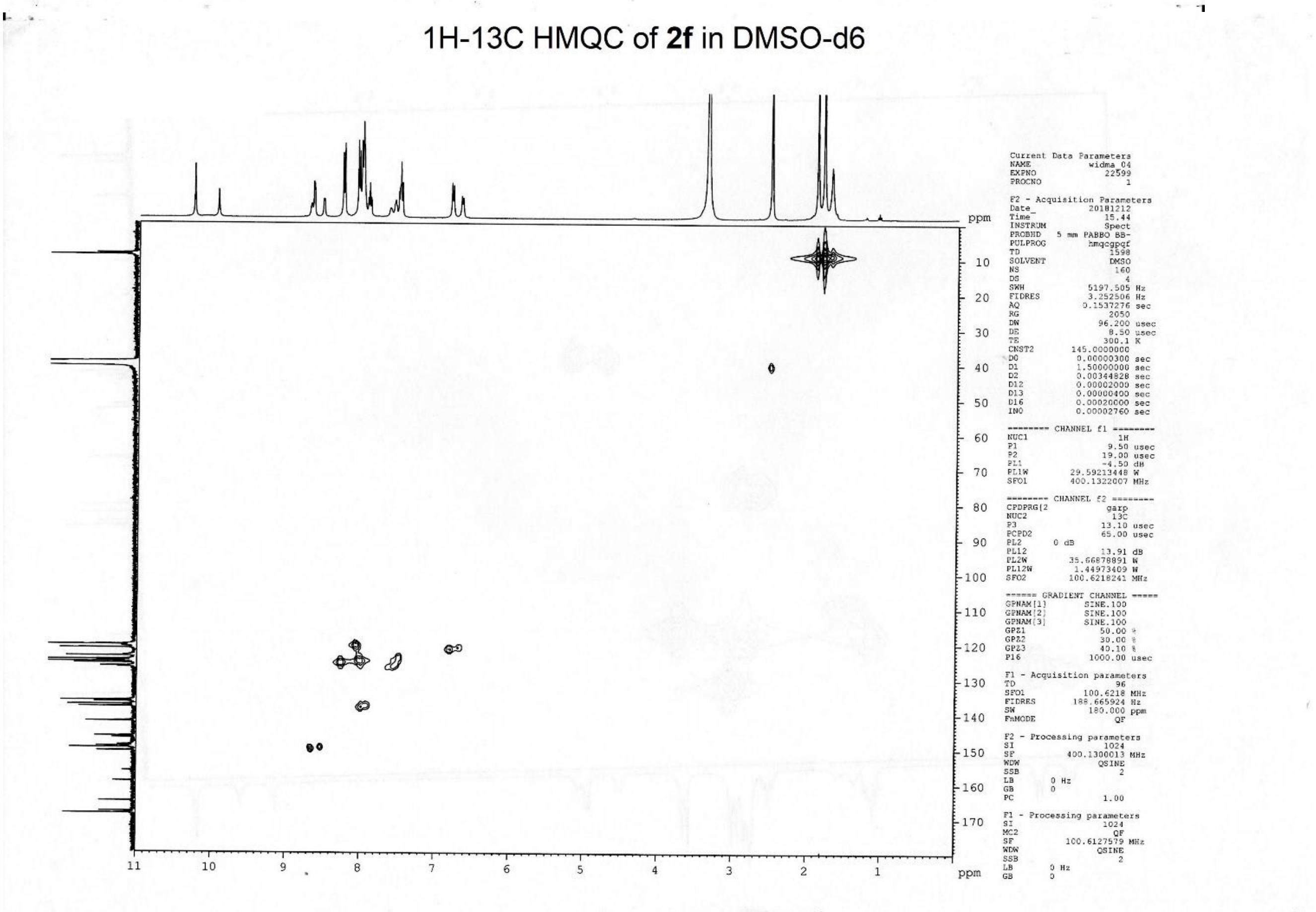

Figure S47. ^{1}H-^{13}C HMQC spectrum of **2f** (in DMSO-d_6), with enlarged fragment at the top

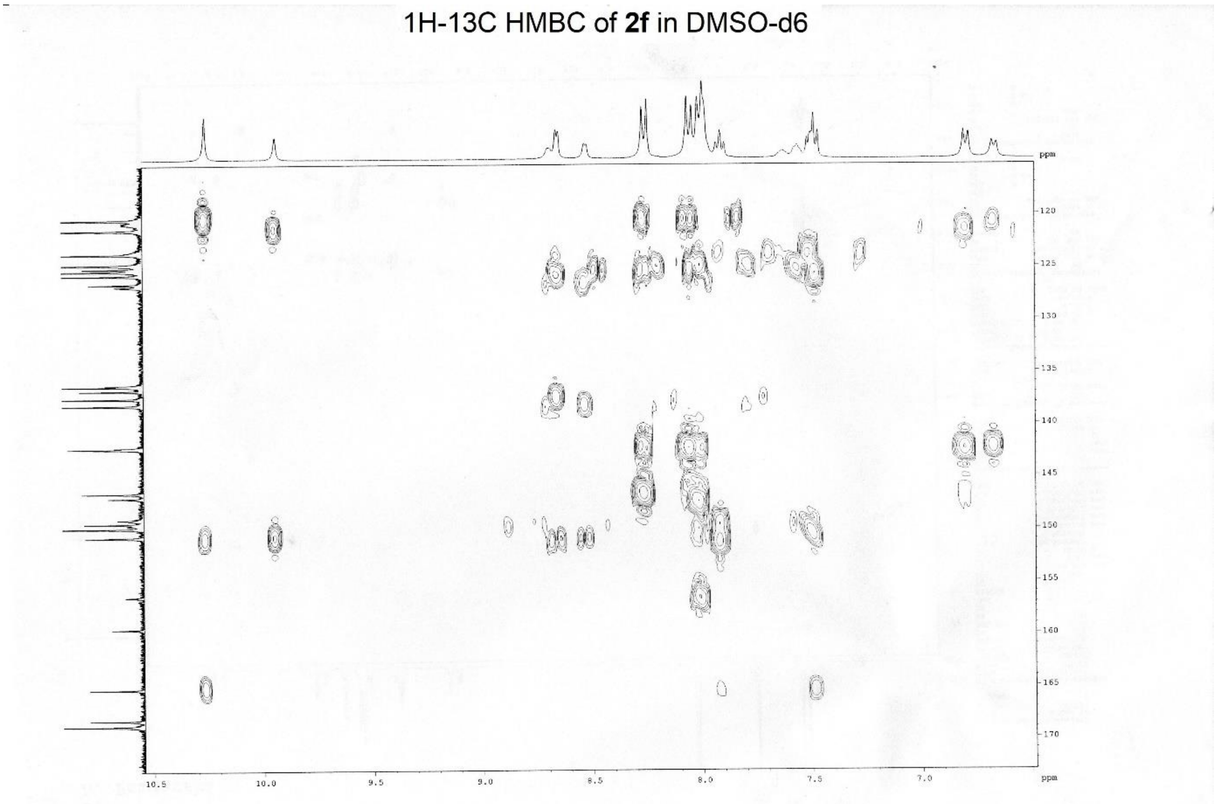

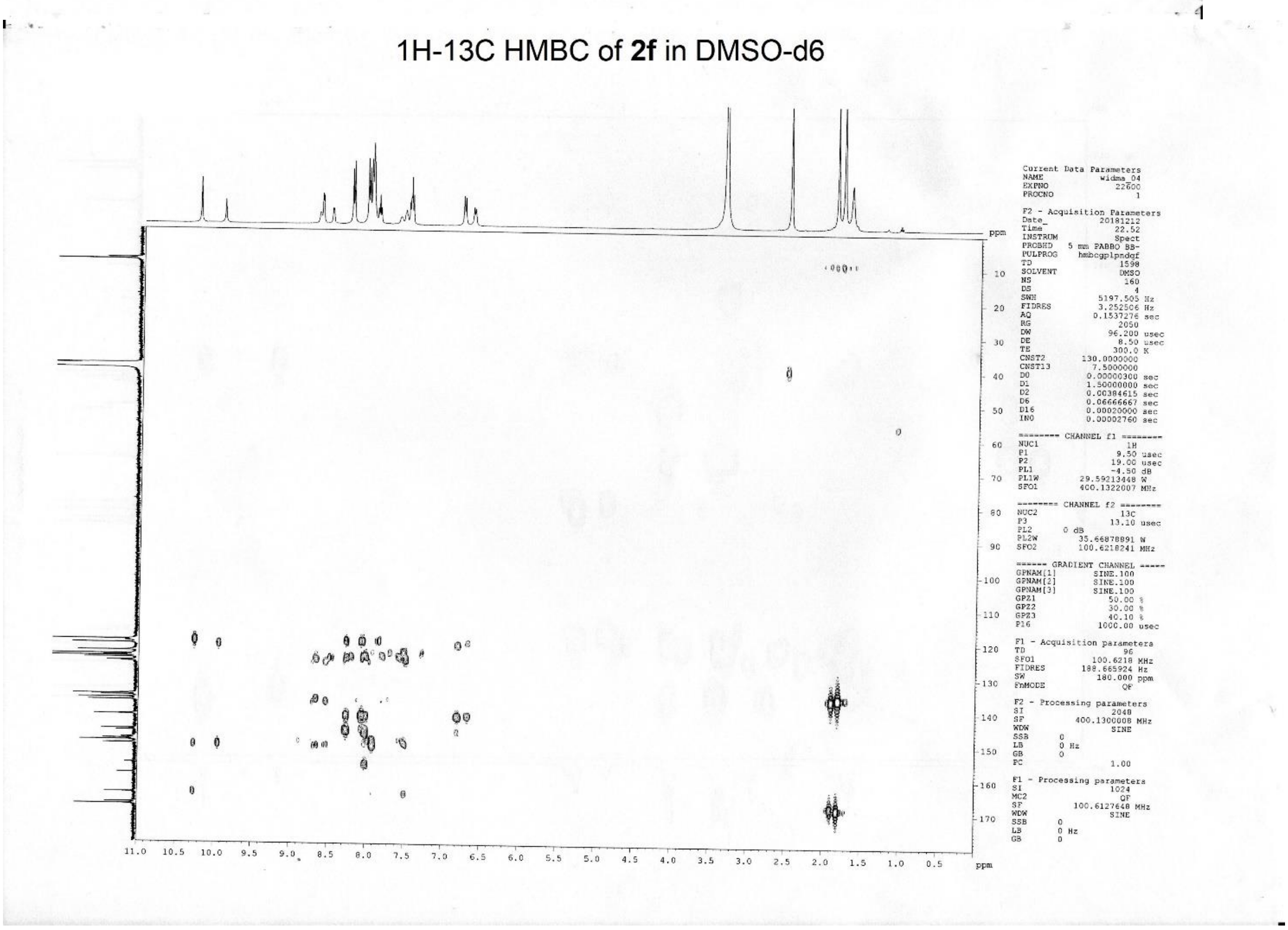

Figure S48. ^{1}H-^{13}C HMBC spectrum of **2f** (in DMSO-d_6), with enlarged fragment at the top

PART D. ^{1}H AND ^{13}C NMR DATA OF 1a-1f and 2a-2f (A, B ISOMERS)

COMPOUND 1a (R^1 = R^2 = phenyl)

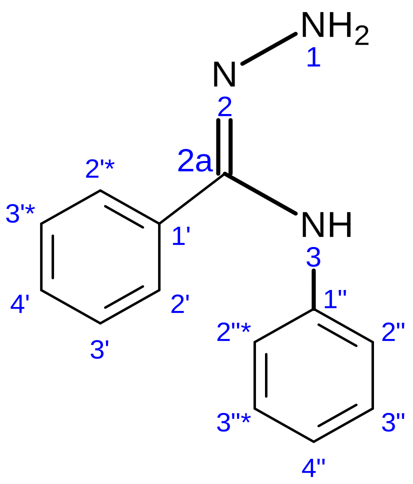

^{1}H NMR
Aliphatic chain
H(1), *i.e.* NH_2: 6.15
H(3), *i.e.* NH: 7.74

Phenyl ring (C(2a)-bonded)
H(2',2'*): 7.52; correlates with C(2a), C(2'*,2'), C(4')
H(3',3'*): 7.30; correlates with C(1'), C(3'*,3')
H(4'): 7.25; correlates with C(2',2'*)

Phenyl ring (N(3)-bonded)
H(2",2"*): 6.56; correlates with C(2"*,2"), C(4")
H(3",3"*): 7.10; correlates with C(1"), C(3"*,3")
H(4"): 6.70; correlates with C(2",2"*)

^{13}C NMR
Aliphatic chain
C(2a), *i.e.* >C=: 138.9; correlates with H(2',2'*)

Phenyl ring (C(2a)-bonded)
C(1'), *i.e.* C: 136.3; correlates with H(3',3'*)
C(2',2'*), *i.e.* 2 x CH: 126.5; correlates with H(2'*,2'), H(4')
C(3',3'*), *i.e.* 2 x CH: 128.6; correlates with H(3'*,3')
C(4'), *i.e.* CH: 128.1; correlates with H(2',2'*)

Phenyl ring (N(3)-bonded)
C(1") , *i.e.* C: 143.7; correlates with H(3",3"*)
C(2",2"*), *i.e.* 2 x CH: 116.2; correlates with H(2"*,2"), H(4")
C(3",3"*), *i.e.* 2 x CH: 129.2; correlates with H(3"*,3")
C(4"), *i.e.* CH: 119.0; correlates with H(2",2"*)

COMPOUND 1b (R^1 = 2-pyridyl, R^2 = phenyl)

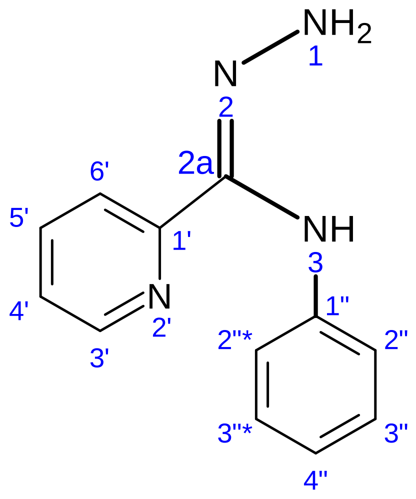

^{1}H NMR

Aliphatic chain

H(1), *i.e.* NH_2: 6.23

H(3), *i.e.* NH: 7.82

2-pyridyl ring (C(2a)-bonded)

H(3'): 8.46; correlates with C(1'), C(4'), C(5')

H(4'): 7.27; correlates with C(3'), C(6')

H(5'): 7.77; correlates with C(1'), C(3')

H(6'): 7.91; correlates with C(2a), C(4')

Phenyl ring (N(3)-bonded)

H(2",2"*): 6.62; correlates with C(2"*,2"), C(4")

H(3",3"*): 7.14; correlates with C(1"), C(3"*,3")

H(4"): 6.74; correlates with C(2",2"*)

^{13}C NMR

Aliphatic chain

C(2a), *i.e.* >C=: 138.1; correlates with H(6')

2-pyridyl ring (C(2a)-bonded)

C(1'), *i.e.* C: 153.9; correlates with H(3'), H(5')

C(3'), *i.e.* CH: 148.6; correlates with H(4'), H(5')

C(4'), *i.e.* CH: 123.0; correlates with H(3'), H(6')

C(5'), *i.e.* CH: 136.9; correlates with H(3')

C(6'), *i.e.* CH: 120.4; correlates with H(4')

Phenyl ring (N(3)-bonded)

C(1") , *i.e.* C: 142.5; correlates with H(3",3"*)

C(2",2"*), *i.e.* 2 x CH: 117.0; correlates with H(2"*,2"), H(4")

C(3",3"*), *i.e.* 2 x CH: 128.9; correlates with H(3"*,3")

C(4"), *i.e.* CH: 119.5; correlates with H(2",2"*)

COMPOUND 1c (R[1] = 4-pyridyl, R[2] = phenyl)

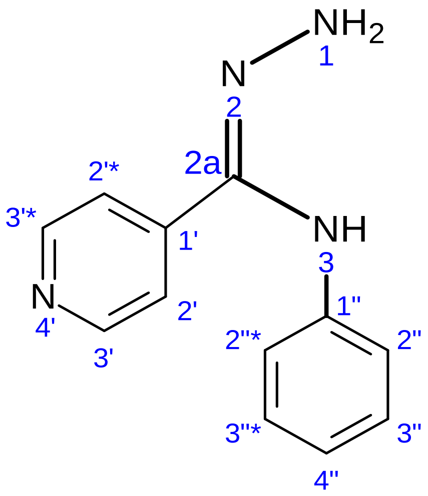

^{1}H NMR

Aliphatic chain

H(1), *i.e.* NH_2: 6.74

H(3), *i.e.* NH: 7.74

4-pyridyl ring (C(2a)-bonded)

H(2’,2’*): 7.42; correlates with C(2a), C(2’*,2’), C(3’,3’*)

H(3’,3’*): 8.46; correlates with C(1’), C(2’,2’*), C(3’*,3’)

Phenyl ring (N(3)-bonded)

H(2”,2”*): 6.53; correlates with C(2”*,2”), C(4”)

H(3”,3”*): 7.12; correlates with C(1”), C(3”*,3”)

H(4”): 6.71; correlates with C(2”,2”*)

^{13}C NMR

Aliphatic chain

C(2a), *i.e.* >C=: 135.3; correlates with H(2’,2’*)

4-pyridyl ring (C(2a)-bonded)

C(1’), *i.e.* C: 143.8; correlates with H(3’,3’*)

C(2’,2’*), *i.e.* CH: 120.3; correlates with H(2’*,2’), H(3’,3’*)

C(3’,3’*), *i.e.* CH: 150.0; correlates with H(2’,2’*), H(3’*,3’)

Phenyl ring (N(3)-bonded)

C(1”) , *i.e.* C: 143.5; correlates with H(3”,3”*)

C(2”,2”*), *i.e.* 2 x CH: 115.7; correlates with H(2”*,2”), H(4”)

C(3”,3”*), *i.e.* 2 x CH: 129.4; correlates with H(3”*,3”)

C(4”), *i.e.* CH: 119.1; correlates with H(2”,2”*)

COMPOUND 1d (R[1] = 2-pyridyl, R[2] = 2-pyridyl)

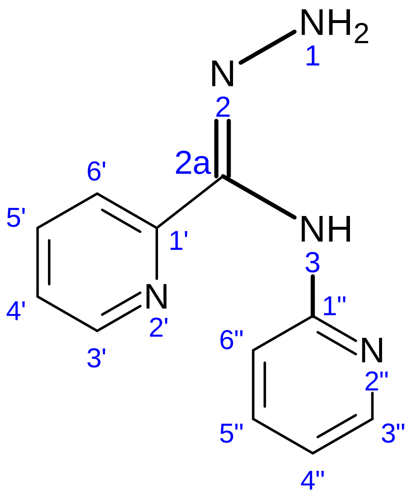

^{1}H NMR

Aliphatic chain

H(1), *i.e.* NH_2: 6.60

H(3), *i.e.* NH: 8.42

2-pyridyl ring (C(2a)-bonded)

H(3'): 8.43; correlates with C(1'), C(4'), C(5')

H(4'): 7.26; correlates with C(3'), C(6')

H(5'): 7.76; correlates with C(1'), C(3')

H(6') 7.90; correlates with C(2a), C(4')

2-pyridyl ring (N(3)-bonded)

H(3"): 8.03; correlates with C(1"), C(4"), C(5")

H(4"): 6.71; correlates with C(3"), C(6")

H(5"): 7.53; correlates with C(1"), C(3")

H(6"): 6.70; correlates with C(4")

^{13}C NMR

Aliphatic chain

C(2a), *i.e.* >C=: 136.9; correlates with H(6')

2-pyridyl ring (C(2a)-bonded)

C(1'), *i.e.* C: 154.1; correlates with H(3'), H(5')

C(3'), *i.e.* CH: 148.4; correlates with H(4'), H(5')

C(4'), *i.e.* CH: 122.9; correlates with H(3'), H(6')

C(5'), *i.e.* CH: 136.8; correlates with H(3')

C(6'), *i.e.* CH: 120.5; correlates with H(4')

2-pyridyl ring (N(3)-bonded)

C(1"), *i.e.* C: 155.7; correlates with H(3"), H(5")

C(3"), *i.e.* CH: 147.8; correlates with H(4"), H(5")

C(4"), *i.e.* CH: 114.9; correlates with H(3"), H(6")

C(5"), *i.e.* CH: 137.8; correlates with H(3")

C(6"), *i.e.* CH: 110.9; correlates with H(4")

COMPOUND 1e (R^1 = 2-pyridyl, R^2 = 4-methylphenyl)

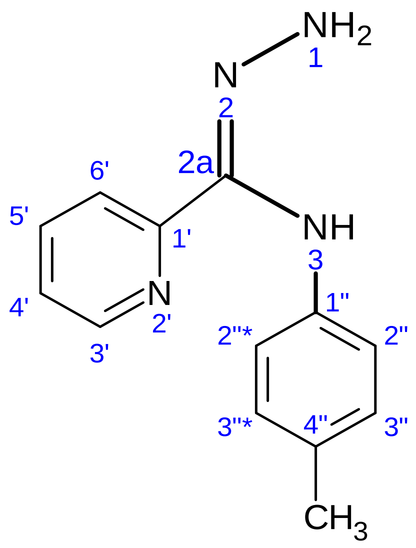

^{1}H NMR

Aliphatic chain

H(1), *i.e.* NH_2: 6.12

H(3), *i.e.* NH: 7.72

2-pyridyl ring (C(2a)-bonded)

H(3’): 8.45; correlates with C(1’), C(4’), C(5’)

H(4’): 7.27; correlates with C(3’), C(6’)

H(5’): 7.77; correlates with C(1’), C(3’)

H(6’): 7.90; correlates with C(2a), C(4’)

4-methylphenyl ring (N(3)-bonded)

H(2”,2”*): 6.53; correlates with C(2”*,2”), C(4”)

H(3”,3”*): 6.95; correlates with C(1”), C(3”*,3”), CH_3

CH_3: 2.18; correlates with C(3”,3”*)

^{13}C NMR

Aliphatic chain

C(2a), *i.e.* >C=: 138.4; correlates with H(6’)

2-pyridyl ring (C(2a)-bonded)

C(1’), *i.e.* C: 153.8; correlates with H(3’), H(5’)

C(3’), *i.e.* CH: 148.5; correlates with H(4’), H(5’)

C(4’), *i.e.* CH: 123.0; correlates with H(3’), H(6’)

C(5’), *i.e.* CH: 136.9; correlates with H(3’)

C(6’), *i.e.* CH: 120.4; correlates with H(4’)

4-methylphenyl ring (N(3)-bonded)

C(1”) , *i.e.* C: 139.8; correlates with H(3”,3”*)

C(2”,2”*), *i.e.* 2 x CH: 117.3; correlates with H(2”*,2”)

C(3”,3”*), *i.e.* 2 x CH: 129.3; correlates with H(3”*,3”), CH_3

C(4”), *i.e.* C: 128.3; correlates with H(2”,2”*)

CH_3: 20.6; correlates with H(3”,3”*)

COMPOUND 1f (R^1 = 2-pyridyl, R^2 = 4-nitrophenyl)

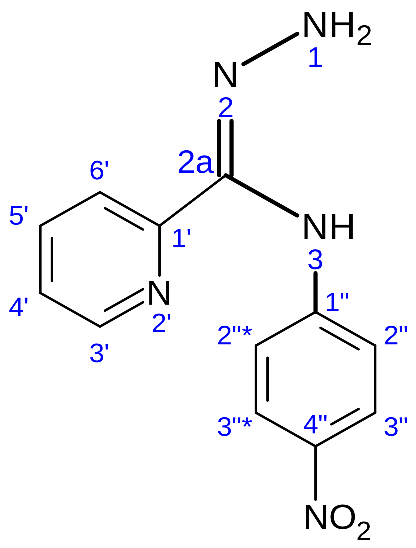

^{1}H NMR

Aliphatic chain

H(1), *i.e.* NH_2: 6.80

H(3), *i.e.* NH: 8.86

2-pyridyl ring (C(2a)-bonded)

H(3’): 8.45; correlates with C(1’), C(4’), C(5’)

H(4’): 7.27; correlates with C(3’), C(6’)

H(5’): 7.79; correlates with C(1’), C(3’)

H(6’): 7.90; correlates with C(2a), C(4’)

4-nitrophenyl ring (N(3)-bonded)

H(2”,2”*): 6.63; correlates with C(2”*,2”), C(4”)

H(3”,3”*): 8.05; correlates with C(1”), C(3”*,3”), C(4”)

^{13}C NMR

Aliphatic chain

C(2a), *i.e.* >C=: 135.0; correlates with H(6’)

2-pyridyl ring (C(2a)-bonded)

C(1’), *i.e.* C: 153.5; correlates with H(3’), H(5’)

C(3’), *i.e.* CH: 148.8; correlates with H(4’), H(5’)

C(4’), *i.e.* CH: 123.0; correlates with H(3’), H(6’)

C(5’), *i.e.* CH: 137.0; correlates with H(3’)

C(6’), *i.e.* CH: 120.3; correlates with H(4’)

4-nitrophenyl ring (N(3)-bonded)

C(1”) , *i.e.* C: 150.4; correlates with H(3”,3”*)

C(2”,2”*), *i.e.* 2 x CH: 115.1; correlates with H(2”*,2”)

C(3”,3”*), *i.e.* 2 x CH: 125.8; correlates with H(3”*,3”)

C(4”), *i.e.* C: 138.6; correlates with H(2”,2”*), H(3”,3”*)

COMPOUND 2a ($R^1 = R^2$ = phenyl)

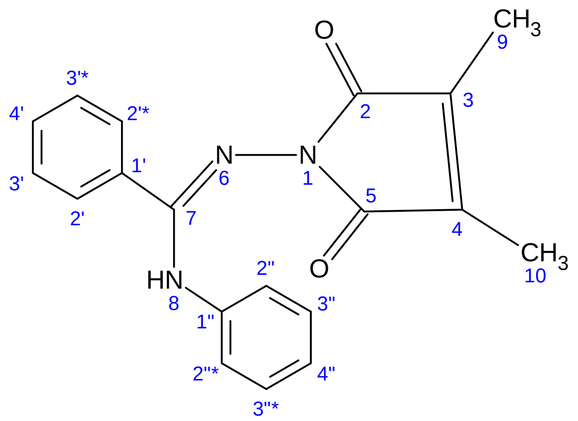

^{1}H NMR

Aliphatic chain+3,4-dimethyl-1H-pyrrole-2,5-dione ring

H(8), *i.e.* NH: **A** – 9.54, **B** – 9.23 (61% : 39%); correlates with C(7), C(1'), C(2",2"*)

3 x H(9,10), *i.e.* 2 x CH_3: **A** – 1.76, **B** – 1.87 (61% : 39%); correlates with C(2,5), C(3,4)

Phenyl ring (C(7)-bonded)

H(2',2'*): **A** – 7.53, **B** – 7.32; correlates with C(7), C(2'*,2'), C(4')

H(3',3'*): **A** – 7.44, **B** – 7.37; correlates with C(1'), C(3'*,3')

H(4'): **A** – 7.43, **B** – 7.28; correlates with C(2',2'*)

Phenyl ring (N(8)-bonded)

H(2",2"*): **A** – 7.80, **B** – 6.74; correlates with C(1"), C(2"*,2"), C(4")

H(3",3"*): **A** – 7.30, **B** – 7.14; correlates with C(1"), C(2",2"*), C(3"*,3")

H(4"): **A** – 7.04, **B** – 6.96; correlates with C(2",2"*), C(3",3"*)

^{13}C NMR

Aliphatic chain+3,4-dimethyl-1H-pyrrole-2,5-dione ring

C(2,5), *i.e.* 2 x CO: **A** – 169.4, **B** – 168.9; correlates with H(9,10)

C(3,4), *i.e.* 2 x >C=: **A** – 135.8, **B** – 136.2; correlates with H(9,10)

C(7), *i.e.* >C=: **A** – 168.5, **B** – 162.8; correlates with H(8), H(2',2'*)

C(9,10), *i.e.* 2 x CH_3: **A** – 8.9, **B** – 9.0; no correlation

Phenyl ring (C(7)-bonded)

C(1'), *i.e.* C: **A** – 133.8, **B** – 133.5; correlates with H(8), H(3',3'*)

C(2',2'*), *i.e.* 2 x CH: **A** – 128.9, **B** – 129.6; correlates with H(2'*,2'), H(4')

C(3',3'*), *i.e.* 2 x CH: **A** – 128.7, **B** – 127.5; correlates with H(3'*,3')

C(4'), *i.e.* CH: **A** – 130.3, **B** – 131.2; correlates with H(2',2'*)

Phenyl ring (N(8)-bonded)

C(1") , *i.e.* C: **A** – 140.6, **B** – 140.4; correlates with H(2",2"*), H(3",3"*)

C(2",2"*), *i.e.* 2 x CH: **A** – 121.0, **B** – 123.5; correlates with H(8), H(2"*,2"), H(3",3"*), H(4)"

C(3",3"*), *i.e.* 2 x CH: **A** and **B** – 2 x 128.9; correlates with H(3"*,3"), H(4")

C(4"), *i.e.* CH: **A** – 123.4, **B** – 124.0; correlates with H(2",2"*)

COMPOUND 2b (R^1 = 2-pyridyl, R^2 = phenyl)

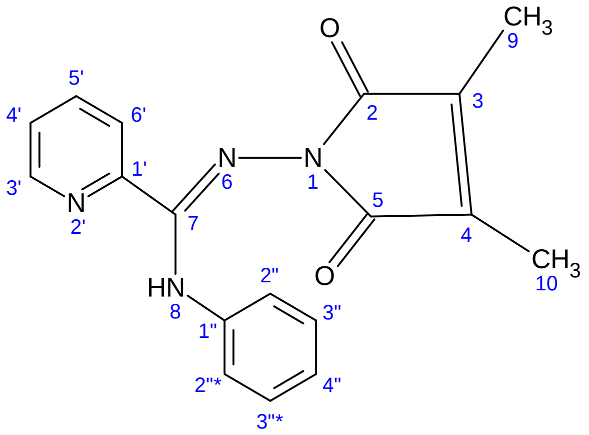

^{1}H NMR

Aliphatic chain+3,4-dimethyl-1H-pyrrole-2,5-dione ring

H(8), *i.e.* NH: **A** – 9.57, **B** – 9.50 (43% : 57%); correlates with C(7), C(1'), C(2",2"*)

3 x H(9,10), *i.e.* 2 x CH_3: **A** – 1.77, **B** – 1.73 (43% : 57%); correlates with C(2,5), C(3,4)

2-pyridyl ring

H(3'): **A** – 8.61, **B** – 8.58; correlates with C(1'), C(4'), C(5')

H(4'): **A** – 7.45, **B** – 7.55; correlates with C(3'), C(6')

H(5'): **A** – 7.87, **B** – 7.95; correlates with C(1'), C(3')

H(6'): **A** – 7.40, **B** – 8.02; correlates with C(7), C(1'), C(4')

Phenyl ring

H(2",2"*): **A** – 7.80, **B** – 6.76; correlates with C(1"), C(2"*,2"), C(4")

H(3",3"*): **A** – 7.32, **B** – 7.14; correlates with C(1"), C(2",2"*), C(3"*,3")

H(4"): **A** – 7.05, **B** – 7.00; correlates with C(2",2"*), C(3",3"*)

^{13}C NMR

Aliphatic chain+3,4-dimethyl-1H-pyrrole-2,5-dione ring

C(2,5), *i.e.* 2 x CO: **A** – 169.2, **B** – 168.2; correlates with H(9,10)

C(3,4), *i.e.* 2 x >C=: **A** – 135.9, **B** – 135.7; correlates with H(9,10)

C(7), *i.e.* >C=: **A** – 165.5, **B** – 158.5; correlates with H(8), H(2')

C(9,10), *i.e.* 2 x CH_3: **A** – 8.9, **B** – 8.8; no correlation

2-pyridyl ring

C(1'), *i.e.* C: **A** – 151.6, **B** – 151.1; correlates with H(8), H(3'), H(5'), H(6')

C(3'), *i.e.* CH: **A** – 149.7, **B** – 149.0; correlates with H(4'), H(5')

C(4'), *i.e.* CH: **A** – 125.3, **B** – 126.2; correlates with H(3'), H(6')

C(5'), *i.e.* CH: **A** – 137.2, **B** – 137.8; correlates with H(3')

C(6'), *i.e.* CH: **A** – 123.5, **B** – 124.0; correlates with H(4')

Phenyl ring

C(1"), *i.e.* C: **A** – 140.4, **B** – 139.1; correlates with H(2",2"*), H(3",3"*)

C(2",2"*), *i.e.* 2 x CH: **A** – 120.9, **B** – 124.4; correlates with H(8), H(2"*,2"), H(3",3"*), H(4")

C(3",3"*), *i.e.* 2 x CH: **A** – 129.0, **B** – 128.4; correlates with H(3"*,3"), H(4")

C(4"), *i.e.* CH: **A** – 123.4, **B** – 124.7; correlates with H(2",2"*)

COMPOUND 2c (R^1 = 4-pyridyl, R^2 = phenyl)

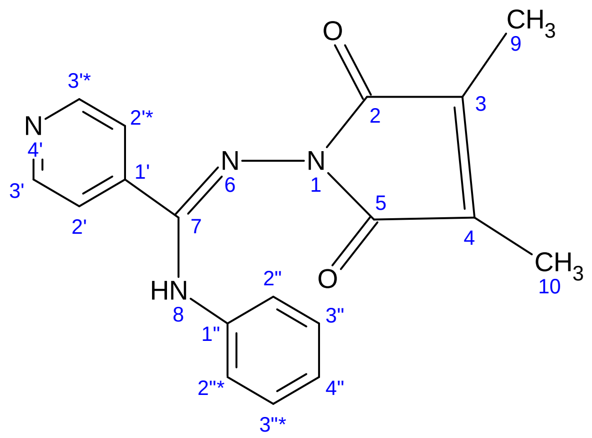

¹H NMR

Aliphatic chain+3,4-dimethyl-1H-pyrrole-2,5-dione ring

H(8), *i.e.* NH: **A** – 9.71, **B** – 9.46 (63% : 37%); correlates with C(7), C(1'), C(2",2"*)

3 x H(9,10), *i.e.* 2 x CH_3: **A** – 1.77, **B** – 1.87 (62% : 38%); correlates with C(2,5), C(3,4)

4-pyridyl ring

H(2',2'*): **A** – 7.28, **B** – 7.46; correlates with C(1'), C(2'*,2'), C(3',3'*)

H(3',3'*): **A** – 8.65, **B** – 8.60; correlates with C(7), C(1'), C(2',2'*), C(3'*,3')

Phenyl ring

H(2",2"*): **A** – 7.76, **B** – 6.76; correlates with C(1"), C(2"*,2"), C(4")

H(3",3"*): **A** – 7.33, **B** – 7.17; correlates with C(1"), C(2",2"*), C(3"*,3")

H(4"): **A** – 7.07, **B** – 7.01; correlates with C(2",2"*), C(3",3"*)

¹³C NMR

Aliphatic chain+3,4-dimethyl-1H-pyrrole-2,5-dione ring

C(2,5), *i.e.* 2 x CO: **A** – 169.3, **B** – 168.6; correlates with H(9,10)

C(3,4), *i.e.* 2 x >C=: **A** – 136.2, **B** – 136.4; correlates with H(9,10)

C(7), *i.e.* >C=: **A** – 166.2, **B** – 160.9; correlates with H(8), H(2')

C(9,10), *i.e.* 2 x CH_3: **A** – 9.0, **B** – 8.9; no correlation

4-pyridyl ring

C(1'), *i.e.* C: **A** and **B** – 2 x 141.3; correlates with H(8), H(2',2'*), H(3',3'*)

C(2',2'*), *i.e.* 2 x CH: **A** – 122.2, **B** – 123.7; correlates with H(2'*,2'), H(3',3'*)

C(3',3'*) *i.e.* 2 x CH: **A** – 150.2, **B** – 150.4; correlates with H(2',2'*), H(3'*,3')

Phenyl ring

C(1"), *i.e.* C: **A** – 140.2, **B** – 139.6; correlates with H(2",2"*), H(3",3"*)

C(2",2"*), *i.e.* 2 x CH: **A** – 121.0, **B** – 123.9; correlates with H(8), H(2"*,2"), H(3",3"*), H(4")

C(3",3"*), *i.e.* 2 x CH: **A** and **B** – 2 x 129.1; correlates with H(3"*,3"), H(4")

C(4"), *i.e.* CH: **A** – 123.7, **B** – 124.6; correlates with H(2",2"*)

COMPOUND 2d ($R^1 = R^2$ = 2-pyridyl)

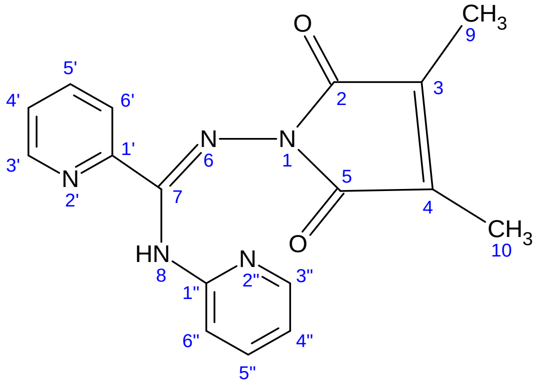

^{1}H NMR

Aliphatic chain+3,4-dimethyl-1H-pyrrole-2,5-dione ring

H(8), *i.e.* NH: **A** – 9.95, **B** – 9.84 (20% : 80%); correlates with C(7), C(1'), C(6")

3 x H(9,10), *i.e.* 2 x CH_3: **A** – 1.78, **B** – 1.88 (22% : 78%); correlates with C(2,5), C(3,4)

2-pyridyl ring (C(7)-bonded)

H(3'): **A** – 8.58, **B** – 8.41; correlates with C(1'), C(4'), C(5')

H(4'): **A** – 7.38, **B** – 7.45; correlates with C(3'), C(6')

H(5'): **A** – 7.95, **B** – 7.85; correlates with C(1'), C(3')

H(6'): **A** – 8.12, **B** – 7.89; correlates with C(7), C(1'), C(4')

2-pyridyl ring (N(8)-bonded)

H(3"): **A** – 8.26, **B** – 7.91; correlates with C(1"), C(4"), C(5")

H(4"): **A** – 7.09, **B** – 6.87; correlates with C(3"), C(6")

H(5"): **A** – 7.56, **B** – 7.57; correlates with C(1"), C(3")

H(6"): **A** – 8.17, **B** – 6.80; correlates with C(1"), C(4")

^{13}C NMR

Aliphatic chain+3,4-dimethyl-1H-pyrrole-2,5-dione ring

C(2,5), *i.e.* 2 x CO: **A** – 168.9, **B** – 168.4; correlates with H(9,10)

C(3,4), *i.e.* 2 x >C=: **A** – 136.2, **B** – 136.4; correlates with H(9,10)

C(7), *i.e.* >C=: **A** – 164.8, **B** – 160.3; correlates with H(8), H(2')

C(9,10), *i.e.* 2 x CH_3: **A** and **B** – 2 x 9.0; no correlation

2-pyridyl ring (C(7)-bonded)

C(1'), *i.e.* C: **A** – 150.7, **B** – 152.4; correlates with H(8), H(3'), H(5'), H(6')

C(3'), *i.e.* CH: **A** – 149.7, **B** – 148.6; correlates with H(4'), H(5')

C(4'), *i.e.* CH: **A** – 125.0, **B** – 125.5; correlates with H(3'), H(6')

C(5'), *i.e.* CH: **A** and **B** – 2 x 137.4; correlates with H(3')

C(6'), *i.e.* CH: **A** and **B** – 2 x 124.3; correlates with H(4')

2-pyridyl ring (N(8)-bonded)

C(1"), *i.e.* C: **A** – 152.7, **B** – 154.0; correlates with H(3"), H(5"), H(6")

C(3"), *i.e.* CH: **A** – 148.6, **B** – 147.6; correlates with H(4"), H(5")

C(4"), *i.e.* CH: **A** – 119.4, **B** – 118.6; correlates with H(3"), H(6")

C(5"), *i.e.* CH: **A** and **B** – 2 x 137.8; correlates with H(3")

C(6"), *i.e.* CH: **A** – 115.2, **B** – 115.3; correlates with H(8), H(4")

COMPOUND 2e (R^1 = 2-pyridyl, R^2 = 4-methylphenyl)

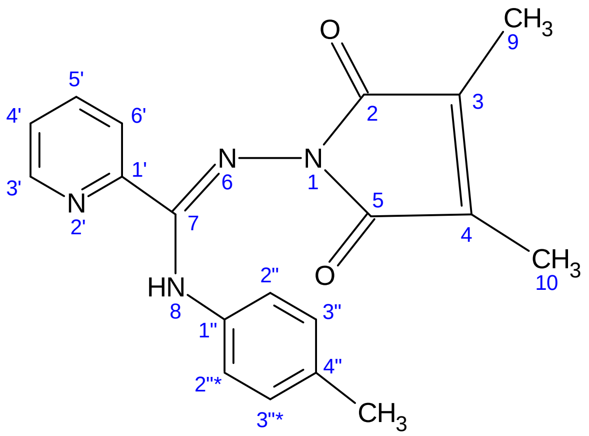

^{1}H NMR

Aliphatic chain+3,4-dimethyl-1H-pyrrole-2,5-dione ring

H(8), *i.e.* NH: **A** – 9.47, **B** – 9.42 (20% : 80%); correlates with C(7), C(1'), C(2",2"*)
3 x H(9,10), *i.e.* 2 x CH_3: **A** – 1.76, **B** – 1.72 (23% : 77%); correlates with C(2,5), C(3,4)

2-pyridyl ring

H(3'): **A** – 8.59, **B** – 8.58; correlates with C(1'), C(4'), C(5')
H(4'): **A** – 7.44, **B** – 7.54; correlates with C(3'), C(6')
H(5'): **A** – 7.86, **B** – 7.94; correlates with C(1'), C(3')
H(6'): **A** – 7.37, **B** – 8.01; correlates with C(7), C(1'), C(4')

4-methylphenyl ring

H(2",2"*): **A** – 7.67, **B** – 6.65; correlates with C(2"*,2"), C(4")
H(3",3"*): **A** – 7.13, **B** – 6.94; correlates with C(1"), C(3"*,3"), CH_3
CH_3: **A** – 2.26, **B** – 2.18; correlates with C(3",3"*), C(4")

^{13}C NMR

Aliphatic chain+3,4-dimethyl-1H-pyrrole-2,5-dione ring

C(2,5), *i.e.* 2 x CO: **A** – 169.2, **B** – 168.6; correlates with H(9,10)
C(3,4), *i.e.* 2 x >C=: **A** – 135.9, **B** – 135.6; correlates with H(9,10)
C(7), *i.e.* >C=: **A** – 165.4, **B** – 158.6; correlates with H(8), H(2')
C(9,10), *i.e.* 2 x CH_3: **A** – 8.9, **B** – 8.7; no correlation

2-pyridyl ring

C(1'), *i.e.* C: **A** – 151.7, **B** – 151.1; correlates with H(8), H(3'), H(5'), H(6')
C(3'), *i.e.* CH: **A** – 149.6, **B** – 148.9; correlates with H(4'), H(5')
C(4'), *i.e.* CH: **A** – 125.2, **B** – 126.1; correlates with H(3'), H(6')
C(5'), *i.e.* CH: **A** – 137.2, **B** – 137.8; correlates with H(3')
C(6'), *i.e.* CH: **A** – 123.4, **B** – 123.9; correlates with H(4')

4-methylphenyl ring

C(1"), *i.e.* C: **A** – 137.9, **B** – 136.4; correlates with H(3",3"*)
C(2",2"*), *i.e.* 2 x CH: **A** – 120.9, **B** – 124.7; correlates with H(8), H(2"*,2")
C(3",3"*), *i.e.* 2 x CH: **A** – 129.4, **B** – 128.9; correlates with H(3"*,3"), CH_3
C(4"), *i.e.* C: **A** – 132.4, **B** – 134.3; correlates with H(2",2"*), CH_3
CH_3: **A** – 20.9, **B** – 20.8; correlates with H(3",3"*)

COMPOUND 2f (R^1 = 2-pyridyl, R^2 = 4-nitrophenyl)

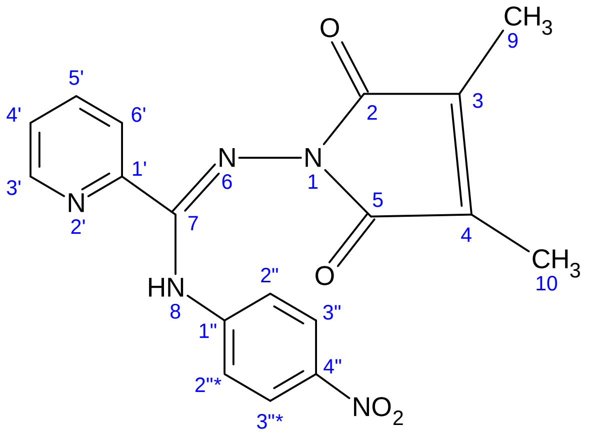

¹H NMR

Aliphatic chain+3,4-dimethyl-1H-pyrrole-2,5-dione ring

H(8), *i.e.* NH: **A** – 10.26, **B** – 9.94 (61% : 39%); correlates with C(7), C(1’), C(2”,2”*)

3 x H(9,10), *i.e.* 2 x CH_3: **A** – 1.80, **B** – 1.89 (61% : 39%); correlates with C(2,5), C(3,4)

2-pyridyl ring

H(3’): **A** – 8.66, **B** – 8.53; correlates with C(1’), C(4’), C(5’)

H(4’): **A** – 7.50, **B** – 7.56; correlates with C(3’), C(6’)

H(5’): **A** – 7.91, **B** – 7.98; correlates with C(1’), C(3’)

H(6’): **A** – 7.48, **B** – 7.51; correlates with C(7), C(1’), C(4’)

4-nitrophenyl ring

H(2”,2”*): **A** – 8.06, **B** – 6.79; correlates with C(2”*,2”), C(4”)

H(3”,3”*): **A** – 8.26, **B** – 8.01; correlates with C(1”), C(3”*,3”)

¹³C NMR

Aliphatic chain+3,4-dimethyl-1H-pyrrole-2,5-dione ring

C(2,5), *i.e.* 2 x CO: **A** – 168.8, **B** – 168.2; correlates with H(9,10)

C(3,4), *i.e.* 2 x >C=: **A** – 136.3, **B** – 136.7; correlates with H(9,10)

C(7), *i.e.* >C=: **A** – 165.2, **B** – 159.4; correlates with H(8), H(2’)

C(9,10), *i.e.* 2 x CH_3: **A** – 9.0, **B** – 8.6; no correlation

2-pyridyl ring

C(1’), *i.e.* C: **A** – 150.8, **B** – 150.7; correlates with H(8), H(3’), H(5’), H(6’)

C(3’), *i.e.* CH: **A** – 149.9, **B** – 149.4; correlates with H(4’), H(5’)

C(4’), *i.e.* CH: **A** – 125.1, **B** – 126.5; correlates with H(3’), H(6’)

C(5’), *i.e.* CH: **A** – 137.4, **B** – 138.1; correlates with H(3’)

C(6’), *i.e.* CH: **A** – 123.7, **B** – 125.7; correlates with H(4’)

4-nitrophenyl ring

C(1”), *i.e.* C: **A** – 146.5, **B** – 147.0; correlates with H(3”,3”*)

C(2”,2”*), *i.e.* 2 x CH: **A** – 120.4, **B** – 121.4; correlates with H(8), H(2”*,2”)

C(3”,3”*), *i.e.* 2 x CH: **A** – 125.3, **B** – 124.7; correlates with H(3”*,3”)

C(4”), *i.e.* C: **A** – 142.3, **B** – 142.2; correlates with H(2”,2”*)

PART E. SUMMARY AND DISCUSSION OF ^{1}H AND ^{13}C NMR CHEMICAL SHIFTS FOR 2a-2f (A AND B ISOMERS), COMPARED TO THOSE OF 1a-1f

Table S8. ^{1}H NMR chemical shifts for **A** and **B** forms of **2a-2f**, and **1a-1f** (in italics), in DMSO-d_6 (δ^{1H}, ppm), at 298 K

Species/ hydrogen	H(8)[a] (NH)	H(9,10) (both CH_3)	H(2') (CH) *i.e.* H(12)	H(3') (CH) *i.e.* H(13)	H(4') (CH) *i.e.* H(14)	H(5') (CH) *i.e.* H(15)	H(6') (CH) *i.e.* H(16)	H(2'') (CH) *i.e.* H(22)	H(3'') (CH) *i.e.* H(23)	H(4'') (CH) *i.e.* H(24)	H(5'') (CH) *i.e.* H(25)	H(6'') (CH) *i.e.* H(26)
**2a A**$^{\text{major (61\%)}}$	9.54	1.76	7.53	7.44	7.43	*	**	7.80	7.30	7.04	*	**
**2a B**$^{\text{minor (39\%)}}$	9.23	1.87	7.32	7.37	7.28	*	**	6.74	7.14	6.96	*	**
1a	*7.74*		*7.52*	*7.30*	*7.25*	*	**	*6.56*	*7.10*	*6.70*	*	**
**2b A**$^{\text{minor (43\%)}}$	9.57	1.77	no H	8.61	7.45	7.87	7.40	7.80	7.32	7.05	*	**
**2b B**$^{\text{major (57\%)}}$	9.50	1.73	no H	8.58	7.55	7.95	8.02	6.76	7.14	7.00	*	**
1b	*7.82*		*no H*	*8.46*	*7.27*	*7.77*	*7.91*	*6.62*	*7.14*	*6.74*	*	**
**2c A**$^{\text{major (62\%)}}$	9.71	1.77	7.28	8.65	no H	*	**	7.76	7.33	7.07	*	**
**2c B**$^{\text{minor (38\%)}}$	9.46	1.87	7.46	8.60	no H	*	**	6.76	7.17	7.01	*	**
1c	*7.74*		*7.42*	*8.46*	*no H*	*	**	*6.53*	*7.12*	*6.71*	*	**
**2d A**$^{\text{minor (21\%)}}$	9.95	1.78	no H	8.58	7.38	7.95	8.12	no H	8.26	7.09	7.56	8.17
**2d B**$^{\text{major (79\%)}}$	9.84	1.88	no H	8.41	7.45	7.85	7.89	no H	7.91	6.87	7.57	6.80
1d	*8.42*		*no H*	*8.43*	*7.26*	*7.76*	*7.90*	*no H*	*8.03*	*6.71*	*7.53*	*6.70*
**2e A**$^{\text{minor (22\%)}}$	9.47	1.76	no H	8.59	7.44	7.86	7.37	7.67	7.13	no H[b]	*	**
**2e B**$^{\text{major (78\%)}}$	9.42	1.72	no H	8.58	7.54	7.94	8.01	6.65	6.94	no H[c]	*	**
1e	*7.72*		*no H*	*8.45*	*7.27*	*7.77*	*7.90*	*6.53*	*6.95*	*no H*[d]	*	**
**2f A**$^{\text{major (61\%)}}$	10.26	1.80	no H	8.66	7.50	7.91	7.48	8.06	8.26	no H	*	**
**2f B**$^{\text{minor (39\%)}}$	9.94	1.89	no H	8.53	7.56	7.98	7.51	6.79	8.01	no H	*	**
1f	*8.86*		*no H*	*8.45*	*7.27*	*7.79*	*7.90*	*6.63*	*8.05*	*no H*	*	**

* the H(5') and/or H(5'') signals are identical to the H(3') and/or H(3'') ones, respectively

** the H(6') and/or H(6'') signals are identical to the H(2') and/or H(2'') ones, respectively

[a] in parent amidrazones this hydrogen is numbered as H(3)

[b] CH_3 at 2.26 ppm

[c] CH_3 at 2.18 ppm

[d] *CH_3 at 2.18 ppm*

Table S9. ^{13}C NMR chemical shifts for **A** and **B** forms of **2a-2f**, and **1a-1f** (in italics), in DMSO-d_6 (δ^{1H}, ppm), at 298 K

Species	C(2,5) (both CO)	C(3,4) (both >C=)	C(7)[a] (>C=)	C(9,10) (both CH_3)	C(1') (C) *i.e.* C(11)	C(2') (CH) *i.e.* C(12)	C(3') (CH) *i.e.* C(13)	C(4') (CH) *i.e.* C(14)	C(5') (CH) *i.e.* C(15)	C(6') (CH) *i.e.* C(16)	C(1'') (C) *i.e.* C(21)	C(2'') (CH) *i.e.* C(22)	C(3'') (CH) *i.e.* C(23)	C(4'') (CH) *i.e.* C(24)	C(5'') (CH) *i.e.* C(25)	C(6'') (CH) *i.e.* C(26)
**2a A**^{major}	169.4	135.8	168.5	8.9	133.8	128.9	128.7	130.3	*	**	140.6	121.0	128.9	123.4	*	**
**2a B**^{minor}	168.9	136.2	162.8	9.0	133.5	129.6	127.5	131.2	*	**	140.4	123.5	128.9	124.0	*	**
1a	no C	no C	*138.9*	no C	*136.3*	*126.5*	*128.6*	*128.1*	*	**	*143.7*	*116.2*	*129.2*	*119.0*	*	**
**2b A**^{minor}	169.2	135.9	165.5	8.9	151.6	no C	149.7	125.3	137.2	123.5	140.4	120.9	129.0	123.4	*	**
**2b B**^{major}	168.2	135.7	158.5	8.8	151.1	no C	149.0	126.2	137.8	124.0	139.2	124.4	128.4	124.7	*	**
1b	no C	no C	*138.1*	no C	*153.9*	*no C*	*148.6*	*123.0*	*136.9*	*120.4*	*142.5*	*117.0*	*128.9*	*119.5*	*	**
**2c A**^{major}	169.3	136.2	166.2	9.0	141.3	122.2	150.2	no C	*	**	140.2	121.0	129.1	123.7	*	**
**2c B**^{minor}	168.6	136.4	160.9	8.9	141.3	123.7	150.4	no C	*	**	139.6	123.9	129.1	124.6	*	**
1c	no C	no C	*135.3*	no C	*143.8*	*120.3*	*150.0*	*no C*	*	**	*143.5*	*115.7*	*129.4*	*119.1*	*	**
**2d A**^{minor}	168.9	136.2	164.8	9.0	150.7	no C	149.7	125.0	137.4	124.3	152.7	no C	148.6	119.4	137.8	115.2
**2d B**^{major}	168.4	136.4	160.3	9.0	152.4	no C	148.6	125.5	137.4	124.3	154.0	no C	147.6	118.6	137.8	115.3
1d	no C	no C	*136.9*	no C	*154.1*	*no C*	*148.4*	*122.9*	*136.8*	*120.5*	*155.7*	*no C*	*147.8*	*114.9*	*137.8*	*110.9*
**2e A**^{minor}	169.2	135.9	165.4	8.9	151.7	no C	149.6	125.2	137.2	123.4	137.9	120.9	129.4	132.4[b]	*	**
**2e B**^{major}	168.6	135.6	158.6	8.7	151.1	no C	148.9	126.1	137.8	123.9	136.4	124.7	128.9	134.3[c]	*	**
1e	no C	no C	*138.4*	no C	*153.8*	*no C*	*148.5*	*123.0*	*136.9*	*120.4*	*139.8*	*117.3*	*129.3*	*128.3*[d]	*	**
**2f A**^{major}	168.8	136.3	165.2	9.0	150.8	no C	149.9	125.1	137.4	123.7	146.5	120.4	125.3	142.3	*	**
**2f B**^{minor}	168.2	136.7	159.4	8.6	150.7	no C	149.4	126.5	138.1	125.7	147.0	121.4	124.7	142.2	*	**
1f	no C	no C	*135.0*	no C	*153.5*	*no C*	*148.8*	*123.0*	*137.0*	*120.3*	*150.4*	*115.1*	*125.8*	*138.6*	*	**

[a] in parent amidrazones this carbon is numbered as C(2a)

[b] CH_3 20.9 ppm

[c] CH_3 20.8 ppm

[d] *CH_3 20.6 ppm*

* the C(5') and/or C(5'') signals are identical to the C(3') and/or C(3'') ones, respectively

** the C(6') and/or C(6'') signals are identical to the C(2') and/or C(2'') ones, respectively

COMMENTS:

1. The assignment of ^{1}H and ^{13}C NMR signals, based on the ^{1}H-^{13}C HMQC and HMBC correlation analysis (for details see part D), generally refers to the atom numbering scheme shown in Figure 2 and Scheme 1 of the main text; the notation 1'>>6' (for R^1) and 1">>6" (for R^2) concerns the NMR signals, while the analogous 11>>16 and 21>>26 one – the respective atoms. Then, during comparisons of each two analogous molecules (belonging to the **2a**-**2f** and **1a**-**1f** type, respectively) containing the same set of R^1, R^2 substituents (*i.e.* corresponding to the same letter from the range "**a-f**"), the generalized symbols **2x** and **1x** (where **x** = **a**, **b**, **c**, **d**, **e**, **f**) are used.

2. The ^{1}H-^{13}C HMQC and HMBC correlation schemes are similar for all **2a**-**2f** compounds, including both **A** and **B** species (for details see part D). Particularly, in the ^{1}H-^{13}C HMBC spectra the mostly deshielded proton signal of **A** or **B** (each of NH type) always correlates (*via* two or three bonds) with the C(7), C(1') and C(2'') (in **2d** with C(6'')) resonances (although not with the C(1") one) – which confirms this is N(8)H (and not N(6)H). It excludes the opportunity that **A** and **B** differ in the position of a proton at both concerned nitrogens, which could be possible upon the hypothetical –N(6)=C(7)–N(8)H– ⇌ –N(6)H–C(7)=N(8)– equilibrium. The presence of N(8)H moiety in **2a**-**2f** molecules (in both **A** and **B** forms) in the solution is consistent with the results of single crystal X-ray studies for **2a** and **2d**. Moreover, the appearance of ^{1}H-^{13}C correlations over two or three bonds for this amine (N(8)H) proton indicates its immobility (in contrast, for **1a**-**1f** no such correlations with participation of the analogous N(3)H atom appear, suggesting it is highly mobile). Hence, the observed **A**–**B** isomerism cannot be caused by any proton transfer, especially between N(6) and N(8). Thus, **2a**-**2f** molecules seem to be exactly as shown in Figure 2 and Scheme 1 of the main text (taking into account the order of atoms and bonds, not geometry).

3. For **2a** the variable temperature ^{1}H NMR measurements were performed. However, heating of the DMSO-d_6 solution from 298 K to 323 K did not affect the relative content of **A** and **B** species (298 K – 61% : 39%, 303 K – 60% : 40%, 313 K – 61% : 39%, 323 K – 61% : 39%; determined as averages of the respective $N(8)H^A$: $N(8)H^B$ and CH_3^A : CH_3^B integration areas). Moreover, all ^{1}H NMR signals remained sharp and their δ^{1H} parameters nearly unchanged. The lack of variations upon the temperature increase by as much as 25 K confirms that **A** and **B** are rather geometric isomers than any tautomers related by the proton exchange.

4. The $\delta^{H(8)}$ values for **2a**-**2f** (*ca.* 9.2-10.3 ppm) are by *ca.* 1-2 ppm larger than for **1a**-**1f** (*ca.* 7.7-8.9 ppm). Similar differences of *ca.* 2 ppm can be concluded for the known analogues of

2a-**2c** containing 1,2-cyclohexanedicarboximide (*1H*-pyrrolidine-2,5-dione moiety) instead of 3,4-dimethyl-*1H*-pyrrole-2,5-dione ($\delta^{H(8)}$ = 9.6-9.8 ppm, all in DMSO-d_6 [EE,FF]).

5. A strong ^{1}H deshielding phenomenon in **2a**-**2f** is most likely caused by an anisotropic effect of the *1H*-pyrrole-2,5-dione system, being the sum of two components: the one of a heteroaromatic pyrrole ring (6 π electrons) and that of both carbonyl side groups (2 * 2 = 4 π electrons). However, the latter contribution seems to be even more efficient than the former one because in the already mentioned **2a**-**2c** analogues containing a saturated *1H*-pyrrolidine-2,5-dione moiety (*i.e.* having no aromatic π electrons) the observed $\delta^{H(8)}$ increases, in respect to **1a**-**1c**, were large as well (by *ca.* 2 ppm) [EE,FF]. Nevertheless, one can assume that both effects play an important role in determining the $\delta^{H(8)}$ values in **2a**-**2f**.

6. For each given **2x** molecule the observed difference between H(8) signals in **A** and **B** (varying from 0.05 ppm in **2e** to 0.32 ppm in **2f**) probably results from various magnitude of the total anisotropic effect at this hydrogen. It suggests that H(8) atom in **A** and **B**, for a given **2x** molecule, has a different geometric position in respect to the *1H*-pyrrole-2,5-dione system, being a source of this anisotropic effect.

7. A and **B** are most likely geometric isomers differing in the position of R^1 and N(8)H-R^2 substituents at the C(7) carbon. The lack of rotation around the N(6)=C(7) double bond results in *cis*-/*trans*- isomerism: in one stereomer R^1 is *trans* to N(1) and N(8)H-R^2 is *cis* to N(1), whereas in the other one R^1 is *cis* to N(1) and N(8)H-R^2 is *trans* to N(1). Taking into account the spatial orientation of N(1) and N(8) atoms, these are *Z* and *E* stereomers, as shown at the scheme below (see also Figure 3 of the main text).

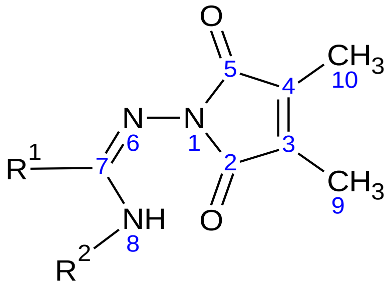

Z (R^1 *trans* to N(1), N(8)H-R^2 *cis* to N(1))

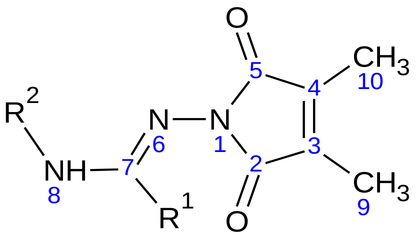

E (R^1 *cis* to N(1), N(8)H-R^2 *trans* to N(1))

8. Such *Z*/*E* isomerism was already suggested for two other amidrazones, analogous to **1a**-**1f** (however, substituted both at N(1) and N(3)), *i.e.* 1-(4-methylphenylsulfonyl)-3-(4-hydroxyphenyl-(2-ethyl))-acetamidrazone and 1-(4-methylphenylsulfonyl)-3-(4-hydroxyphenyl-(2-ethyl))-propionamidrazone, for which a similar doubling of ^{1}H and ^{13}C NMR signals was observed in DMSO-d_6 solutions [GG].

9. The hypothesis of *Z*/*E* isomerism is supported by the fact that, in the solid phase of **2a** or **2d**, where only one stereomer is observed, the crystal structures correspond just to such distinct isomeric species: **2a** to *Z* and **2d** to *E*.

10. The further analysis of ^{1}H NMR spectra of **2a-2f** allows to propose attribution of *Z* and *E* geometry to the **A** and **B** species, respectively:

a) First of all, when to compare both geometric isomers, one can conclude that in each given **2x** molecule the anisotropic effect of the *1H*-pyrrole-2,5-dione system, appearing at the H(8) atom, should be more expressed for *Z* than *E* stereomer. The reason is the shortening of the spatial distance between H(8) and the *1H*-pyrrole-2,5-dione moiety upon the virtual $E \rightarrow Z$ transition. It must result in a stronger deshielding of H(8), *i.e.* in a higher $\delta^{H(8)}$ value, which indicates that *E* geometry corresponds to **B**, while the *Z* one to **A**.

b) Such a geometric relation between *Z* and *E* stereomers is well-exemplified by comparison of the single crystal X-ray structures of **2a** and **2d**. In solid **2a** (*Z* stereomer) the distance between H(8) and the centroid of the pyrrole ring (N(1)<<C(5)) is only 4.256 Å, whereas in solid **2d** (*E* stereomer) it is as much as 5.419 Å. Similarly, the distances between H(8) and the centre of C(2)=O or C(5)=O double bonds are 3.461 Å or 4.896 Å in solid **2a** (geometry; mean 4.179 Å) and 5.054 Å or 5.214 Å in solid **2d** (*E* geometry, mean 5.134 Å). Obviously, the distance from a π-electron system is not the only factor determining the magnitude of the anisotropic effect, nevertheless, its increase by as much as *ca.* 1 Å must have an observable impact.

c) A strong anisotropic effect of the *1H*-pyrrole-2,5-dione system is observed also for H(2") and/or H(6") signals of the R^2 substituent in the **A** forms of **2a-2f**, as exhibited by the $\delta^{H(2")}$ and/or $\delta^{H(6")}$ chemical shifts, increased by *ca.* 1.1-1.5 ppm (**2a-A** +1.24 ppm, **2b-A** +1.18 ppm, **2c-A** +1.23 ppm, **2d-A** +1.47 ppm, **2e-A** +1.14 ppm, **2f-A** +1.43 ppm), comparing to **1a-1f**. This phenomenon is probably caused by the fact that in *Z* stereomers (corresponding to the **A** species) there is a relatively parallel orientation of the R^2 ring in respect to the *1H*-pyrrole-2,5-dione system (as exhibited by solid **2a** of *Z* geometry, where the dihedral angle between the N(1)<<C(5) and C(21)<<C(26) best planes is 26.9^{o}), while the average distances between H(22) and/or H(26) atoms and the π-electron systems of the *1H*-pyrrole-2,5-dione moiety are relatively short, being less than 4 Å (in solid **2a:** H(22)/H(26)...'pyrrole ring centroid' 3.525 Å/3.986 Å – mean 3.756 Å; H(22)...'middle point of C(2)=O bond' / H(22)...'middle point of C(5)=O bond' / H(26)...'middle point of C(2)=O bond' / H(26)...'middle point of C(5)=O bond' 4.298 Å/2.965 Å/3.265 Å/5.174 Å – mean 3.925 Å).

d) The same anisotropic effect is nearly absent for the other signals of the R^2 substituent in the **A** forms of **2a**-**2f**, most likely due to the fact that in *Z* stereomers the respective protons are much more far-distant from the *1H*-pyrrole-2,5-dione moiety; in fact, the respective average distances noticeably exceed 4 Å (in solid **2a**: H(23)/H(25)...'pyrrole ring centroid' 4.143 Å/4.604 Å – mean 4.374 Å; H(24)...'pyrrole ring centroid' 4.622 Å; H(23)...'middle point of C(2)=O bond' / H(23)...'middle point of C(5)=O bond' / H(25)...'middle point of C(2)=O bond' / H(25)...'middle point of C(5)=O bond' 5.335 Å/3.654 Å/4.610 Å/5.638 Å – mean 4.809 Å; H(24)...'middle point of C(2)=O bond' / 'middle point of C(5)=O bond' 5.449 Å/4.967 Å – mean 5.208 Å). As already mentioned, the impact of both carbonyl groups is probably even more important than that of the *1H*-pyrrole ring, thus the increase of the above mean distances between various protons in R^2 and the middle points of the C=O double bonds (3.925 Å for H(22)/H(26) → 4.809 Å for H(23)/H(25) → 5.208 Å for H(24)) well explains the observed dependency of their ^{1}H chemical shifts.

e) An analogous anisotropic effect does not appear for H(2") and/or H(6") signals of the R^2 substituent in the **B** forms of **2a**-**2f**, as the respective increases of $\delta^{H(2")}$ and/or $\delta^{H(6")}$ chemical shifts, comparing to **1a**-**1f**, are very small (by *ca.* 0.1-0.2 ppm). It is consistent with the *E* geometry of these species, upon which the R^2 ring is more perpendicular to the *1H*-pyrrole-2,5-dione system (as exhibited by solid **2d** being *E* stereomer, where the dihedral angle between the N(1)<<C(5) and C(21)<<C(26) best planes is 76.8°), while the distances between H(22) and/or H(26) atoms and the π-electron systems of the *1H*-pyrrole-2,5-dione moiety are much longer (in **2d**: H(26)...'pyrrole ring centroid' 4.390 Å; H(26)...'middle point of C(2)=O bond' / 'middle point of C(5)=O bond' 3.834 Å/4.551 Å – mean 4.192 Å). In our opinion, again the increase of the average distance between H(22)/H(26) or H(26) and the center of the C=O double bond(s), noted upon the *Z* (in **2a**) → *E* (in **2d**) transition (3.925 Å → 4.192 Å) is probably the most significant.

f) There is a question, however, why nearly no anisotropic effect is observed for the H(2') and H(6') signals of the R^1 substituent in the **B** forms of **2a**-**2f**, *i.e.* in *E* stereomers – although the R^1 substituents are similarly positioned *vis-á-vis* this complex π-electron system like the R^2 ones in *Z* isomers? Seemingly, one could expect an analogous deshielding of H(12) and H(16) protons in the **B** species like for H(22) and H(26) in the **A** forms, but such a phenomenon does not occur at all; in reality, the respective changes of $\delta^{H(2')}$ and/or $\delta^{H(6')}$ chemical shifts, comparing to **1a**-**1f**, are of variable sign and moderate or small absolute magnitude (max. to *ca.* ±0.4 ppm, sometimes even *ca.* 0.0 ppm – like H(6') in **2d**-**B**). Most likely, the lack of such an effect is caused by the fact that the R^1 ring is nearly perpendicular to the *1H*-pyrrole-2,5-dione

system (in solid **2d** the dihedral angle between the N(1)<<C(5) and C(11)<<C(16) best planes is 78.1^{o}) and more far-distant (through space) than the R^2 one in Z stereomers. The latter circumstance is well-exemplified by observation that in solid **2d** the ‘centroid of the pyrrole ring’...’centroid of the C(2a)-bonded 2-pyridyl ring’ distance is 4.320 Å, *i.e.* as much as *ca.* 1.0 Å more than the ‘centroid of the pyrrole ring’...’centroid of the N(3)-bonded phenyl ring’ one in solid **2a**, being 3.340 Å. Moreover, in solid **2d** the mean distance H(16)...’middle point of C(2)=O bond’ / ‘middle point of C(5)=O bond’ (5.217 Å/2.818 Å) is 4.018 Å, *i.e. ca.* 0.1 Å more than that of H(22)...’middle point of C(2)=O bond’ / H(22)...’middle point of C(5)=O bond’ / H(26)...’middle point of C(2)=O bond’ / H(26)...’middle point of C(5)=O bond’, already determined as 3.925 Å. All these factors reduce the anisotropic effect of the *1H*-pyrrole-2,5-dione system, resulting in only slight $\delta^{H(2')}$ and/or $\delta^{H(6')}$ variations in the **B** forms being *E* stereomers.

g) Attribution of *Z* geometry to the **A** species, and that of *E* to the **B** one, corresponds well to the fact the in the DMSO-d_6 solutions of **2a** and **2d**, the respective major forms (**2a**: 61% **A**, **2d**: 79% **B**) are identical to those exclusively appearing in the solid phase (**2a**: *Z*, **2d**: *E*).

11. In solid **2d** the intramolecular C(26)−H(26)···N(6) hydrogen bond is observed due to the *E* geometry. However, it must be relatively weak, as its influence at the H(6”) signal in **2d-B** is rather small ($\delta^{H(6'')}$ = 6.80 ppm, while 6.70 ppm for **1d**), especially when to compare with a much larger impact of the *1H*-pyrrole-2,5-dione anisotropic effect in **2d-A** ($\delta^{H(6'')}$ = 8.17 ppm).

12. In the ^{13}C NMR spectra the most expressed result of each **1x** → **2x** transition is observed for the aliphatic C(7) atom of **2a-2f** (denoted as C(2a) in **1a-1f**), which is deshielded by *ca.* 20-30 ppm ($\delta^{C(2a)}$ = *ca.* 135-139 ppm → $\delta^{C(7)}$ = *ca.* 158-169 ppm). This phenomenon again reveals importance of the anisotropic effect of the *1H*-pyrrole-2,5-dione moiety. Then, for each given **2x** molecule the C(7) deshielding is slightly stronger for **A** than **B** species (by *ca.* 4-7 ppm), again confirming that **A** has *Z* geometry and **B** has *E* geometry – as in *Z* stereomers the distances between C(7) and the π-electron systems of the *1H*-pyrrole-2,5-dione system are shorter than in the *E* ones. Such geometric relations are exemplified in the solid phase by comparison of the respective distances in **2a** (*Z* isomer) and **2d** (*E* isomer): C(7)…’pyrrole ring centroid’ – 3.387 Å in **2a** *vs* 3.456 Å in **2d**; C(7)… ‘middle point of C(2)=O bond’ / ‘middle point of C(5)=O bond’ – 2.976 Å / 3.607 Å (mean 3.292 Å) in **2a** *vs* 3.285 Å / 3.408 Å (mean 3.347 Å in **2d**).

13. The changes of $\delta^{C(1')-C(6')}$ and $\delta^{C(1'')-C(6'')}$ parameters upon the **1x** → **2x** transitions are variable; these chemical shifts in **2a-2f** are typical for the respective aromatic rings in R^1 and R^2 substituents. The $\delta^{C(2,5)}$ = 168.2-169.4 ppm, $\delta^{C(3,4)}$ = 135.6-136.7 ppm and $\delta^{C(9,10)}$ = 8.6-9.0 ppm values correspond well to those reported for analogous carbons in variously substituted

N(1)-amino, N(1)-amido and N(1)-imino derivatives of *1H*-pyrrole-2,5-diones, *i.e.* CO 154-174 ppm, **C**-CH_3 135-150 ppm and CH_3 8-17 ppm, which are presented in Table S10:

Table S10. ^{13}C NMR chemical shifts for selected N(1)-amino, N(1)-amido and N(1)-imino derivatives of 1*H*-pyrrole-2,5-diones

**Class of chemicals: 1*H*-pyrrole-2,5-diones (L)**	Compound	Solvent	^{13}C NMR chemical shift for the 1*H*-pyrrole-2,5-dione ring		
			C(2,5)^{CO}	C(3,4)$^{C\ a}$	CH_3
N(1)-amino derivative of 1H-pyrrole-2,5-dione (L^1)	1,1-dimethylamino-L^1	$CDCl_3$ [G]	174.1	absent	absent
	1-phenylamino-L^1	$CDCl_3$ [G]<br>DMSO-d_6 [H]	172.3<br>167.0	absent	absent
	1-(4-methylphenyl)amino-L^1	$CDCl_3$ [G]	172.4	absent	absent
	1-(4-methoxyphenyl)amino-L^1	$CDCl_3$ [G]	171.3	absent	absent
	1-(4-bromophenylamino)-L^1	DMSO-d_6 [I]	169.1	absent	absent
	1-(piperidyn-1-yl)-L^1	$CDCl_3$ [G]	170.3	absent	absent
	1-(morpholin-4-yl)-L^1	$CDCl_3$ [G]	171.2	absent	absent
	1-(4-methylpiperazin-1-yl)-L^1	$CDCl_3$ [G]	171.2	absent	absent
	1,1-diphenylamino-L^1	$CDCl_3$ [G]	171.2	absent	absent
N(1)-imino derivative of 1H-pyrrole-2,5-dione (L^1)	1-(*E*-4-phenylbut-3-en-2-ylideneimino)-L^1	$CDCl_3$ [J]	154.1[b,c]<br>154.0[b,c]	absent	absent
	1-(*E*-4-(2-fluorophenyl)but-3-en-2-ylideneimino)-L^1	$CDCl_3$ [J]	both<br>154.0[c]	absent	absent
	1-(*E*-4-(3,5-bis(trifluoromethyl)phenyl)but-3-en-2-ylideneimino)-L^1	$CDCl_3$ [J]	154.3[b,c]<br>154.0[b,c]	absent	absent
	1-(*E*-4-(2,4-dichlorophenyl)but-3-en-2-ylideneimino)-L^1	$CDCl_3$ [J]	both<br>153.9[c]	absent	absent
	1-(*E*-4-(4-nitrophenyl)but-3-en-2-ylideneimino)-L^1	$CDCl_3$ [J]	154.1[b,c]<br>153.9[b,c]	absent	absent
N(1)-amino derivative of 3-methyl-1H-pyrrole-2,5-dione (L^2)	1-phenylamino-L^2	$CDCl_3$ [Q]	165.4[b]<br>168.7[b]	unknown	11.2
	L^2-$L^{2\ d}$	$CDCl_3$ [Q]	163.9[b]<br>166.3[b]	150.2[e]	unknown
N(1)-imino derivative of 3-methyl-1H-pyrrole-2,5-dione (L^2)	1-(*E*-4-phenylbut-3-en-2-ylideneimino)-L^2	$CDCl_3$ [J]	155.1[b]<br>154.4[b]	146.0[e,f]<br>144.4[e,f]	16.9[f]<br>16.4[f]
	1-(*E*-4-(3,5-bis(trifluoromethyl)phenyl)but-3-en-2-ylidene)imino)-L^2	$CDCl_3$ [J]	155.4[b,f]<br>155.0[b,f]<br>154.7[b,f]<br>154.3[b,f]	146.9[e,f]<br>144.3[e,f]	17.0[f]<br>16.4[f]

	1-(*E*-4-(2,4-dichlorophenyl)but-3-en-2-ylideneimino)-L^2	$CDCl_3$ [J]	155.0[b]<br>154.3[b]	146.6[e,f]<br>144.1[e,f]	17.0[f]<br>16.4[f]
N(1)-amino derivative of 3,4-dimethyl-1H-pyrrole-2,5-dione (L^3)	1-phenylamino-L^3	$CDCl_3$ [Q]	165.5	unknown	10.0
	L^3-L^3 [g]	$CDCl_3$ [Q]	166.3	138.9	unknown
	1,1-dimethylamino-L^3	$CDCl_3$ [T]	170.4	135.7	8.6
N(1)-amido derivative of 3,4-diphenyl-1H-pyrrole-2,5-dione (L^5)	1-benzamido-L^5	$CDCl_3$ [BB]	168.1 or 165.9[h]	unknown	absent
	1-(4-methoxybenzamido)-L^5	$CDCl_3$ [BB]	168.2 or 165.4[h]	unknown	absent
	1-(4-bromobenzamido)-L^5	$CDCl_3$ [BB]	168.3 or 165.2[h]	unknown	absent
	1-(4-nitrobenzamido)-L^5	$CDCl_3$ [BB]	168.0 or 164.2[h]	unknown	absent

[a] only methyl-substituted C(3) or C(4) atoms are taken into account

[b] exact assignment to C(2) and C(5) remains unknown

[c] >C(2)=O and >C(5)=O moieties are inequivalent, due to the N(1) substitution by the unsymmetrical –N=CR^1R^2 moiety

[d] systematic name of this dimeric species is 3,3'-dimethyl-1,1'-bipyrrole-2,2',5,5'-tetraone

[e] assigned to C(3)–CH_3

[f] optional doubling of some C(2)=O / C(5)=O, C(3)–CH_3 and CH_3 signals probably reflects the presence of two geometric isomers differing in orientation of the unsymmetrical –N=CR^1R^2 moiety in respect to the 3-substituted pyrrole ring

[g] systematic name of this dimeric species is 3,3',4,4'-tetramethyl-1,1'-bipyrrole-2,2',5,5'-tetraone

[h] it is unclear whether this is >C(2,5)=O or –NH–CO– carbon

PART F. X-RAY STRUCTURES OF 2a AND 2d

Table S11. Selected bond lengths (Å), bond angles (°) and torsion angles (°) in the molecules **2a** and **2d,** and the closely related, CSD-reported X-ray structure LUZGUJ [FF]

bond / angle	**2a**	**2d**	LUZGUJ[a]
geometry	*Z*	*E*	*E*
N(1)–N(6)	1.409(1)	1.419(2)	1.406(4)
N(6)–C(7)	1.307(2)	1.301(2)	1.306(4)
C(7)–N(8)	1.363(2)	1.361(2)	1.353(4)
N(1)–C(2)	1.397(2)	1.401(2)	1.401(5)
C(2)–C(3)	1.503(2)	1.497(2)	1.508(5)
C(3)–C(4)	1.334(2)	1.340(2)	1.523(5)
C(4)–C(5)	1.498(2)	1.501(2)	1.490(5)
C(5)–N(1)	1.379(2)	1.401(2)	1.369(5)
C(2)–O(1)	1.209(2)	1.208(2)	1.192(4)
C(5)–O(2)	1.213(2)	1.212(2)	1.204(4)
C(7)–C(11)	1.488(2)	1.508(2)	1.482(5)
N(8)–C(21)	1.422(2)	1.410(2)	1.404(4)
C(2)–N(1)–N(6)	125.0(1)	119.8(1)	121.7(3)
C(5)–N(1)–N(6)	123.9(1)	124.1(1)	122.7(3)
C(5)–N(1)–C(2)	110.3(1)	109.5(1)	113.8(3)
N(1)–N(6)–C(7)	113.5(1)	112.6(1)	111.9(3)
N(6)–C(7)–N(8)	128.8(1)	120.8(1)	120.4(3)
N(6)–C(7)–C(11)	114.9(1)	124.3(1)	125.7(3)
C(11)–C(7)–N(8)	116.3(1)	114.9(1)	113.8(3)
C(7)–N(8)–C(21)	125.7(1)	128.4(1)	130.4(4)
N(1)–C(2)–C(3)–C(4)	2.3(1)	4.1(2)	16.6(4)
C(2)–C(3)–C(4)–C(5)	1.3(1)	0.8(2)	−16.2(4)
C(3)–C(4)–C(5)–N(1)	−4.5(1)	−5.5(2)	10.3(4)
C(4)–C(5)–N(1)–C(2)	6.0(1)	8.1(2)	0.4(4)
C(5)–N(1)–C(2)–C(3)	−5.2(1)	−7.7(2)	−11.1(5)
O(1)–C(2)–N(1)–N(6)	4.0(2)	21.4(2)	6.5(6)
O(2)–C(5)–N(1)–N(6)	−3.6(2)	−22.3(2)	−12.6(5)
C(2)–N(1)–N(6)–C(7)	−63.4(2)	−103.4(2)	−76.2(4)
N(1)–N(6)–C(7)–C(11)	166.3(1)	−11.0(2)	−8.4(5)
N(1)–N(6)–C(7)–N(8)	−13.0(2)	171.5(1)	173.1(3)
N(6)–C(7)–C(11)–C(16)	−29.6(2)	−73.3(2)	−66.5(5)
N(6)–C(7)–N(8)–C(21)	−22.3(2)	−7.5(2)	−6.7(6)
C(7)–N(8)–C(21)–C(26)	133.7(1)	−4.0(2)	−3.5(7)

[a] in this compound the N(1)<<C(5) ring is not aromatic but saturated (*1H*-pyrrolidine-2,5-dione moiety being a part of the 1,2-cyclohexanedicarboximide system)

Table S12. Selected bond lengths in the aliphatic chain of **2a**, **2d** and of the X-ray reported N^1-acylamidrazones

Compound	CSD reference code	Geometry, N(1)-N(6)-C(7)-N(8) or N(1)-N(2)-C(2a)-N(3) torsion angle [º]	N(1)-N(6) or N(1)-N(2) bond length [Å]	N(6)-C(7) or N(2)-C(2a) bond length [Å]	C(7)-N(8) or C(2a)-N(3) bond length [Å]	N(1)-N(6)-C(7) or N(1)-N(2)-C(2a) bond angle [º]	N(6)-C(7)-N(8) or N(2)-C(2a)-N(3) bond angle [º]
**2a**	**This work**	*Z*, -13.0(2)	1.409(1)	1.307(2)	1.363(2)	113.5(1)	128.8(1)
**2d**	**This work**	*E*, 171.5(1)	1.419(2)	1.301(2)	1.361(2)	112.6(1)	120.8(1)
**1d**$^{N(1)}$-acyl [a]	PAZDIF [HH]*	*Z*, 0.4(4)	1.386(2)	1.290(3)	1.383(3)	119.2(2)	136.9(2)
**1d**$^{N(1)}$-acyl [b]	RIBVEG [II]*	*Z*, -0.4(5)	1.371(3)	1.301(5)	1.356(5)	118.7(3)	134.1(3)
**1d**$^{N(1)}$-acyl [c1]	RICGUI [II]*	*Z*, 2.5(4)	1.388(3)	1.284(3)	1.391(3)	118.8(2)	132.7(2)
**1d**$^{N(1)}$-acyl [c2]	RICHAP [II]*	*Z*, 2.9(5)/1.9(6)	1.386(3)/ 1.387(5)	1.288(4)/ 1.295(4)	1.388(4)/ 1.384(4)	119.3(2) 120.3(3)	132.4(3) 136.2(3)
**1d**$^{N(1)}$-acyl **1d** [d]	RICHET [II]*	*Z*, 0.8(3)	1.383(2)	1.289(2)	1.381(2)	120.1(1)	134.4(1)
**1d**$^{N(1)}$-acyl **1d** [e]	RICHIX [II]*	*Z*, -0.2(3)	1.384(2)	1.293(2)	1.381(2)	118.1(1)	131.7(2)
**1d**$^{N(1)}$-acyl **1d** [f]	RICHOD [II]*	*Z*, 3.2(4)/-1.6(4)	1.374(3)/ 1.374(3)	1.291(4)/ 1.284(3)	1.398(4)/ 1.411(4)	117.9(2) 118.6(2)	126.3(3) 125.1(2)
**1d**$^{N(1)}$-acyl · CH_3OH [g]	RICHUJ [II]*	*Z*, 0.5(3)	1.379(2)	1.298(3)	1.381(3)	118.5(2)	134.5(2)

[a] acyl = 6-carboxycyclohex-3-enylcarbonyl
[b] acyl = 2-carboxybenzoyl
[c1, c2] acyl = 2-carboxycyclohexylcarbonyl
[d] acyl = 3-carboxypyrid-2-ylcarbonyl
[e] acyl = 3-carboxypyrid-4-ylcarbonyl
[f] acyl = 3-carboxypropionyl
[g] acyl = 4-carboxybutyryl
* In these molecules the numbering of the aliphatic chain is different from **2a** and **2d**, i.e. N(1), N(2), C(2a), N(3) instead of N(1), N(6), C(7), N(8).

Table S13. Selected bond lengths and angles in the 1*H*-pyrrole-2,5-dione moiety of **2a**, **2d** and some other X-ray reported 1*H*-pyrrole-2,5-dione derivatives

**Class of chemicals: 1*H*-pyrrole-2,5-diones (L)**	Compound	CSD reference code	Mean N(1)-C(2)/C(5) bond length [Å]	Mean C(2)-O(1) / C(5)-O(2) bond length [Å]	Mean C(2)-N(1)-C(5) bond angle [°]
1H-pyrrole-2,5-dione (L^1) derivatives	L^1	TEKQAB [C]	1.387(8)	1.215(9)	110.0(5)
	1-(4-bromophenylamino)-L^1	CARHAF [I]	1.390(5)	1.198(5)	111.0(3)
3-methyl-1H-pyrrole-2,5-dione (L^2) derivatives	1-phenylamino-L^2	COXYEV [Q]	1.386(2)	1.198(2)	110.7(1)
	L^2-$L^{2\,c}$	TUBBID [Q]	1.395(4)	1.200(4)	111.6(2)
3,4-dimethyl-1H-pyrrole-2,5-dione (L^3) derivatives	1-phenylamino-L^3	COXYUL [Q]	1.386(2)	1.210(2)	110.3(1)
	L^3-$L^{3\,e}$	COXYAR [Q]<br>COXYAR 01 [Q]	1.404(5)<br>1.397(7)	1.201(6)<br>1.195(7)	111.2(6)
	**2a**	**This work**	**1.388(2)**	**1.211(2)**	**110.3(1)**
	**2d**	**This work**	**1.401(2)**	**1.210(2)**	**109.5(1)**
3,4-diphenyl-1H-pyrrole-2,5-dione (L^5) derivatives	L^5 · 1-methylpyrrolidyn-2-one	NIXRUJ [W]	1.382(2)	1.212(2)	110.4(1)
	1-(4-bromobenzamido)-L^5	KUQRIZ [BB]	1.394(4)	1.194(4)	111.9(2)
	1-methoxycarbonylamino-3,4-bis(4-nitrophenyl)-*1H*-pyrrole-2,5-dione	TAJSIG [CC]	1.388(4)	1.198(4)	111.8(2)

Table S14. Geometries of hydrogen bonds and selected short contacts in the crystals of **2a** and **2d**

Interaction	$d_{D\text{-}H}$ /Å	$d_{H\cdots A}$ / Å	$d_{D\cdots A}$ / Å	<$d_{D\text{-}H\ldots A}$/°	Symmetry code
**2a**					
N(8)–H(8)···O(2)	0.85(2)	2.07(2)	2.901(2)	167(1)	-*x*+1, *y*-1/2, -*z*+1/2
C(26)–H(26)···N(6)	0.93	2.70	3.606(2)	165	
C(9)–H(9a)···C(13)	0.96	2.78	3.492(2)	132	
C(22)–H(22)···O(1)	0.93	2.68	3.514(2)	149	-*x*+1, *y*+1/2, -*z*+1/2
C(10)–H(10b)···N(6)	0.96	2.62	3.581(2)	175	-*x*+1, -*y*+1, -*z*
C(9)–H(9b)···O(1)	0.96	2.60	3.527(2)	163	-*x*+1, -*y*, -*z*
**2d**					
N(8)–H(8)···N(22)	0.90(2)	2.24(2)	3.137(2)	177(1)	-*x*+1, -*y*, -*z*
C(23)–H(23)···π$_{(C(11)\text{-}C(16)\ cent)}$	0.93	2.39	3.311(2)	169	
C(14)–H(14)···O(1)	0.93	2.72	3.284(2)	120	*x*, *y*, *z*+1
C(15)–H(15)···O(1)	0.93	2.66	3.259(2)	122	
C(13)–H(13)···O(1)	0.93	2.69	3.499(2)	146	*x*, -*y*-1/2, *z*+1/2
C(14)–H(14)···N(12)	0.93	2.75	3.444(2)	133	
C(15)–H(15)···O(2)	0.93	2.54	3.287(2)	138	*x*, -*y*+1/2, *z*+1/2
C(10)–H(10b)···C(3)	0.96	2.87	3.785(2)	159	
C(10)–H(10a)···O(1)	0.96	2.55	3.450(2)	156	-*x*, *y*+1/2, -*z*-1/2
C(9)–H(9a)···C(15)	0.96	2.80	3.374(2)	119	-*x*, -*y*, -*z*
C(25)–H(25)···C(21)	0.96	2.75	3.458(2)	134	*x*, -*y*+1/2, *z*-1/2
C(26)–H(26)···N(6)	0.96	2.23	2.829(2)	121	*x*, *y*, *z*

PART G. TOXICITY OF 2a-2f IN PBMC CULTURE

Figure S49. The effect of ibuprofen (IBU) and **2a-2f** at 100 μg/mL dose on the cell viability in PBMCs culture. Dot plots representative of one experiment of apoptosis measurement by Annexin-V-APC/PI staining PBMC.

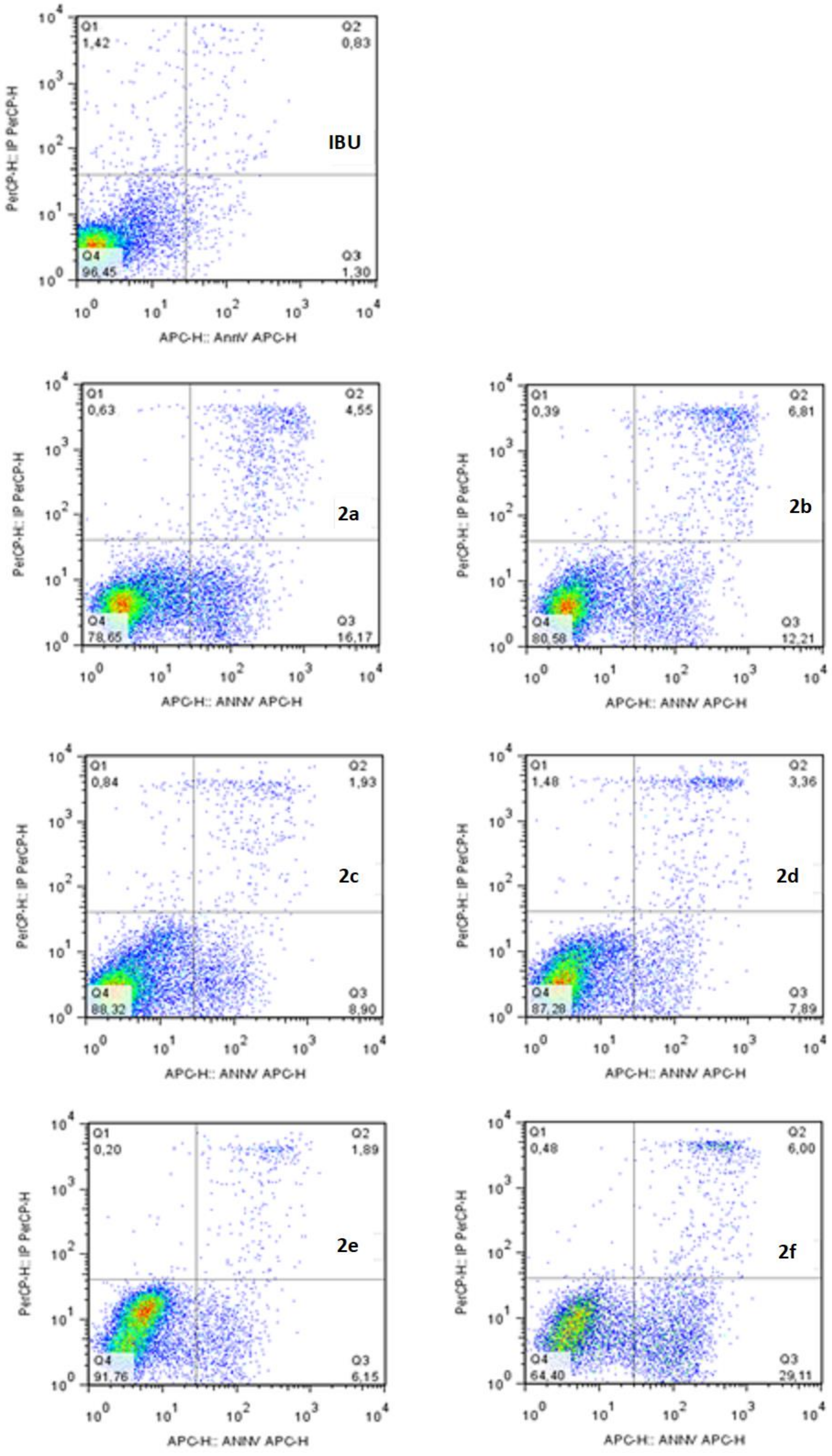

PART H. ANTIBACTERIAL ACTIVITY OF 2a-2f

Table S15. MIC values of **2a-2f**, ampicillin and tetracycline against the tested bacterial strains

	MIC [µg/ml]							
	**2a**	**2b**	**2c**	**2d**	**2e**	**2f**	**ampicillin**	**tetracycline**
E. coli ATCC 25922	>512	>512	>512	256	>512	512	8	2
Y. enterocolitica O3	512	512	256	128	>512	>512	16	4
P. aeruginosa ATCC 27853	512	>512	512	512	>512	>512	>256	16
S. aureus ATCC 25923	128	256	128	256	512	>512	0.5	1
M. luteus	>512	512	512	512	>512	>512	0.5	1
E. faecalis ATCC 29212	>512	>512	>512	512	>512	512	1	16
M. smegmatis	>512	512	256	512	>512	>512	16	2
N. corralina (*Rhodococcus* sp.)	>512	512	512	512	>512	512	8	16

[A] SDBS – Spectral Database for Organic Compounds, National Institute of Advanced Industrial Science and Technology, Japan; http://sdbs.db.aist.go.jp (record 3678 – maleimide), accessed 2021/01/25.

[B] Spectra Base – Free Spectral Database, Bio-Rad Laboratories, Hercules, California (USA), https://spectrabase.com/ (records for maleimide, 3,4-dimethylpyrrol), accessed 2021/01/25.

[C] Cox P.J., Parker S.F. Maleimide. *Acta Crystallogr. C* **1996**, *52*, 2578-2580. https://doi.org/10.1107/S0108270196006841.

[D] Padie C., Zeitler K. A novel reaction-based, chromogenic and "turn-on" fluorescent chemodosimeter for fluoride detection†. *New J. Chem.* **2011**, *35*, 994-997. https://doi.org/10.1039/C0NJ00937G.

[E] Ali M.A., Siddiki S.M.A.H., Kon K., Hasegawa J., Shimizu K. Versatile and Sustainable Synthesis of Cyclic Imides from Dicarboxylic Acids and Amines by Nb_2O_5 as a Base-Tolerant Heterogeneous Lewis Acid Catalyst. *Chem. Eur. J.* **2014**, *20*, 14256-14260. https://doi.org/10.1002/chem.201404538.

[F] Vera-Hidalgo M., Giovanelli E., Navio C., Perez E.M. Mild Covalent Functionalization of Transition Metal Dichalcogenides with Maleimides: A "Click" Reaction for 2H-MoS_2 and WS_2 *J. Am. Chem. Soc.* **2019**, *141*, 3767-3771. https://doi.org/10.1021/jacs.8b10930.

[G] Sadiq A., Mahnashi M.H., Alyami B.A., Alqahtani Y.S., Alqarni A., Rashid U. Tailoring the substitution pattern of Pyrrolidine-2,5-dione for discovery of new structural template for dual COX/LOX inhibition. *Bioorg. Chem.* **2021**, *112*, 104969. https://doi.org/10.1016/j.bioorg.2021.104969.

[H] Cheng S., Comer D.D. An alumina-catalyzed Michael addition of mercaptans to N-anilinomaleimides and its application to the solution-phase parallel synthesis of libraries. *Tetrahedron Lett.* **2002**, *43*, 1179-1181. https://doi.org/10.1016/S0040-4039(01)02401-7.

[I] Conley N.R., Hung R.J., Willson C.G. A New Synthetic Route to Authentic N-Substituted Aminomaleimides. *J. Org. Chem.* **2005**, *70*, 4553-4555. https://doi.org/10.1021/jo048031q.

[J] Song X., Liu C., Chen P., Zhang H., Sun R. Natural Product-Based Pesticide Discovery: Design, Synthesis and Bioactivity Studies of *N*-Amino-Maleimide Derivatives. *Molecules* **2018**, *23*, 1521. https://doi.org/10.3390/molecules23071521.

[K] Gill G.B., James G.D., Oates K.V., Pattenden G. The synthesis of 5-ylidenepyrrol-2(5H)-ones from maleimides and from pyrrol-2-(5H)-ones. *J. Chem. Soc. Perkin Trans. 1* **1993**, 2567-2579. https://doi.org/10.1039/P19930002567.

[L] Kuehne P., Hesse M. Simple synthesis of (±)-(E)-3-(4-hydroxyphenyl)-N-[4-(3-methyl-2,5-dioxo-1-pyrrolidinyl)butyl]-2-propenamide, a novel phenolic amide derivative from the bulbs of Lilium regale WILSON. *Tetrahedron*, **1993**, *49*, 4575-4580. https://doi.org/10.1016/S0040-4020(01)81286-2.

[M] Haddon W.F., Binder R.G., Wong R.Y., Harden L.A., Wilson R.E., Benson M., Stevens K.L. Potent Bacterial Mutagens Produced by Chlorination of Simulated Poultry Chiller Water. *J. Agric. Food Chem.* **1996,** *44*, 256-263. https://doi.org/10.1021/jf950076s.

[N] Zou C., Zeng C., Liu Z., Lu M., Sun X., Ye J. γ'-Selective Functionalization of Cyclic Enones: Construction of a Chiral Quaternary Carbon Center by [4+2] Cycloaddition/Retro-Mannich Reaction with 3-Substituted Maleimides. *Angew. Chem. Int. Ed.* **2016**, *55*, 14257-14261. https://doi.org/10.1002/anie.201605790.

[O] Nagy S., Szigetvari A., Ilkei V., Kramos B., Beni Z., Szantay C. Jr., Hazai L. Synthesis of aminal-type Lilium candidum alkaloids and lilaline; determination of their relative configuration by the concerted use of NMR spectroscopy and DFT conformational analysis. *Tetrahedron* **2021**, *81*, 131827. https://doi.org/10.1016/j.tet.2020.131827.

[P] Yogo M., Hirota K., Maki Y. Synthesis of 5-iminopyrrol-2-one derivatives from 1,3-oxazines. Ring transformations via attack on the 2- or 6-position of 1,3-oxazines *J. Chem. Soc. Perkin Trans. I,* **1984**, 2097-2102. https://doi.org/10.1039/P19840002097.

[Q] Katrusiak A., Katrusiak A. One-step ring condensation of hydrazine derivatives and cyclic anhydrides. *J. Mol. Struct.* **2015**, *1085*, 28-36. https://doi.org/10.1016/j.molstruc.2014.12.050.

[R] Watson D.J., Dowdy E.D., Li W.S., Wang J., Polniaszek R. Electronic effects in the acid-promoted deprotection of N-2,4-dimethoxybenzyl maleimides. *Tetrahedron Lett.* **2001**, *42*, 1827-1830. https://doi.org/10.1016/S0040-4039(01)00031-4.

[S] Rix K., Kelsall G.H., Hellgardt K., Hii K.K.M. Chemo- and Diastereoselectivities in the Electrochemical Reduction of Maleimides. *ChemSusChem* **2015**, *8*, 665-671. https://dx.doi.org/10.1002%2Fcssc.201403184.

[T] Nguyen H.N., Cee V.J., Deak H.L., Du B., Faber K.P., Gunaydin H., Hodous B.L., Hollis S.L., Krolikowski P.H., Olivieri P.R., Patel V.F., Romero K., Schenkel L.B., Geuns-Meyer S.D. Synthesis of 4-substituted chlorophthalazines, dihydrobenzoazepinediones, 2-pyrazolylbenzoic acid, and 2-pyrazolylbenzohydrazide via 3-substituted 3-hydroxyisoindolin-1-ones. *J. Org. Chem*. **2012**, *77*, 3887-3906. https://doi.org/10.1021/jo3000628.

[U] Schilling W., Zhang Y., Riemer D., Das S. Visible-Light-Mediated Dearomatisation of Indoles and Pyrroles to Pharmaceuticals and Pesticides. *Chem. Eur. J.* **2020**, *26*, 390-395. https://doi.org/10.1002/chem.201904168.

[V] Hu W., Zheng J., Li J., Liu B., Wu W., Liu H., Jiang H. Assembly of Polysubstituted Maleimides via Palladium-Catalyzed Cyclization Reaction of Alkynes with Isocyanides. *J. Org. Chem.* **2016**, *81*, 12451-12458. https://doi.org/10.1021/acs.joc.6b02227.

[W] Bulatov E., Boyarskaya D., Chulkova T., Haukka M. 2,3-Diphenylmaleimide 1-methylpyrrolidin-2-one monosolvate. *Acta Crystallogr. E* **2014**, *70*, o260. https://doi.org/10.1107/S1600536814002372.

[X] Yeh H.C., Wu W.C., Wen Y.S., Dai D.C., Wang J.K., Chen C.T. Derivative of α,β-Dicyanostilbene: Convenient Precursor for the Synthesis of Diphenylmaleimide Compounds, *E-Z* Isomerization, Crystal Structure, and Solid-State Fluorescence. *J. Org. Chem.* **2004**, *69*, 6455-6462. https://doi.org/10.1021/jo049512c.

[Y] Jafarpour F., Shamsianpour M., Issazadeh S., Dorrani M., Hazrati H. Palladium-catalyzed direct arylation of maleimides: A simple route to bisaryl-substituted maleimides. *Tetrahedron* **2017**, *73*, 1668-1672. https://doi.org/10.1016/j.tet.2017.01.069.

[Z] Mendoza-Macias C.L., Solorio-Alvarado C.R., Alonso-Castro A.J., Alba-Betancourt C., Deveze-Alvarez M.A., Padilla-Vaca F., Reyes-Gualito A. Discovery of new effective N-alkyl-3,4-diarylmaleimides-based drugs for reversing the bacterial resistance to rhodamine 6G in *Bacillus subtilis*. *Chem. Pap.* **2020**, *74*, 1429-1438. https://doi.org/10.1007/s11696-019-00992-7.

[AA] Chen P., Cao W., Li X., Shi D. A Unified Approach for Divergent Synthesis of Heterocycles via TMSOTf-Catalyzed Formal [3+2] Cycloaddition of Electron-Rich Alkynes. *Adv. Synth. Catal.* **2021**, *363*, 4789-4794. https://doi.org/10.1002/adsc.202100769.

[BB] Zheng R., Mei X., Lin Z., Zhao Y., Yao H., Lva W., Ling Q. Strong CIE activity, multi-stimuli-responsive fluorescence and data storage application of new diphenyl maleimide derivatives. *J. Mater. Chem. C* **2015**, *3*, 10242-10248. https://doi.org/10.1039/C5TC02374B.

[CC] Boubekeur K., Grandjean D., Florac C., Robert A. Structure of N-methoxycarbonylamino-3,4-bis(4-nitrophenyl)maleimide at 140 K. *Acta Crystallogr. C* **1991**, *C47*, 1107-1108. https://doi.org/10.1107/S0108270190010204.

[DD] Allen F.H. The Cambridge Structural Database: a quarter of a million crystal structures and rising. *Acta Crystallogr. B* **2002**, *58*, 380-388. https://doi.org/10.1107/S0108768102003890.

[EE] Modzelewska-Banachiewicz B., Ucherek M., Zimecki M., Kutkowska J., Kaminska T., Morak-Młodawska B., Paprocka R., Szulc M., Lewandowski G., Marciniak J., Bobkiewicz-Kozlowska T. Reactions of N^3-substituted

amidrazones with *cis*-1,2-cyclohexanedicarboxylic anhydride and biological activities of the products. *Arch. Pharm. Chem. Life Sci.* **2012**, *345*, 486-494. https://doi.org/10.1002/ardp.201100333.

[FF] Ziegler-Borowska M., Ucherek M., Kutkowska J., Mazur L. Modzelewska-Banachiewicz B., Kedziera D., Kaczmarek-Kedziera A. Reaction of N^3-phenylbenzamidrazone with *cis*-1,2-cyclohexanedicarboxylic anhydride. *Tetrahedron Lett*. **2010**, *51*, 2951-2955. https://doi.org/10.1016/j.tetlet.2010.03.116.

[GG] Salem A.B., Salah B.B., Mhalla D., Trigui M., Mourer M., Regnouf-de-Vains J.B. Kossentini M. Synthesis, crystal structure and biological studies of novel amidrazones, triazoles, Thiatriazole and Triazine compounds. *J. Mol. Struct.* **2020**, *1214*, 128209. https://doi.org/10.1016/j.molstruc.2020.128209.

[HH] Mazur L., Modzelewska-Banachiewicz B., Paprocka R., Zimecki M., Wawrzyniak U.E., Kutkowska J., Ziolkowska G. Synthesis, crystal structure and biological activities of a novel amidrazone derivative and its copper(II) complex — A potential antitumor drug. *J. Inorg. Biochem*. **2012**, *114*, 55-64. https://doi.org/10.1016/j.jinorgbio.2012.04.021.

[II] Mazur L., Saczewski J., Jarzembska K.N., Szwarc-Karabyka K., Paprocka R., Modzelewska-Banachiewicz B. Synthesis, structural characterization and reactivity of new trisubstituted N1-acylamidrazones: solid state and solution studies. C*rystEngComm* **2018**, *20*, 4179-4193. https://doi.org/10.1039/C8CE00701B.
